# Supplementary material for: Two-step synthesis of chiral fused tricyclic scaffolds from phenols via desymmetrization on nickel
Source: Nat Commun. 2017 Jun 26;8:32. doi: 10.1038/s41467-017-00068-8 (PMC5484674; doi:10.1038/s41467-017-00068-8)
Supplement: Supplementary file 1 — Supplementary Information [file 41467_2017_68_MOESM1_ESM.pdf]

File Name: Supplementary Information
Description: Supplementary Figures, Supplementary Table, Supplementary Methods and Supplementary References.

File Name: Peer Review File
Description:

Supplementary Methods

General considerations and starting materials. All manipulations were conducted under a nitrogen atmosphere by using standard Schlenk or dry box techniques unless otherwise noted. ^{1}H and ^{13}C spectra were recorded on Bruker AVANCE III 400 spectrometers at 25 °C. The chemical shifts in ^{1}H NMR and $^{13}C\{^{1}H\}$ NMR spectra are reported in parts per million (ppm) and are referenced to the residual solvent signal as the internal standard: $CDCl_3$ δ = 7.26 (^{1}H) and δ = 77.0 (^{13}C) ppm; C_6D_6: δ = 7.16 (^{1}H) and δ = 128.1 (^{13}C) ppm. Splitting patterns are denoted as "s" for singlet; "d" for doublet; "t" for triplet; "q" for quartet; "sext" for sextet; "sept" for septet; "m" for multiplet, "br" for broad; "dt" for doublet of triplets; "td" for triplet of doublets, and "app" for apparent. Mass spectra were obtained by using a Shimadzu GCMS-QP 2010 instrument with an ionization voltage of 70 eV. Medium-pressure column chromatography was carried out on a Biotage Flash Purification System Isolera, equipped with a 250 nm UV detector. Analytical gas chromatography (GC) was carried out on a Shimadzu GC-2014 gas chromatograph, equipped with a flame ionization detector. High resolution mass spectrometry (HRMS) and elemental analysis were performed at Instrumental Analysis Center, Faculty of Engineering, Osaka University. Enantioselectivities were recorded by means of either JASCO-Supercritical Fluid chromatography (SFC) equipped with PU-2080-CO_2 plus CO_2 delivery pump and MD-2018 plus as a photodiode array detector using chiral stationary phase columns of Diacel Chiralpak (IA). Optical rotations were measured either in JASCO-DIP-1000 or JASCO-P-2200 polarimeter with a path length of 1 dm using the sodium D line, 589 nm. THF, toluene, and benzene-d_6 were distilled from sodium benzophenone ketyl and other solvents were distilled and degassed prior to use. All commercially available reagents were distilled over CaH_2 under reduced pressure prior to use. All synthesized starting materials were purified either by distillation over CaH_2 or recrystallized prior to use. Chiral *N*-heterocyclic carbene (NHC) salts were synthesized according to the reported procedures.[1–4]

Preparation of alkynyl-cyclohexadienones (1)

The general experimental procedures for the preparation of alkynyl-cyclohexadienone were followed as reported previously (Supplementary Figure 1).[5–7] (Diacetoxyiodo)benzene (1.1 equiv) (PIDA) was added portion wise to a stirred solution of 4-substituted phenol (1.0–15 mmol) in propargyl alcohol (1.0 mL/mmol of phenol) at 0 °C. The solution was allowed to warm to room temperature for overnight. Reaction was quenched with saturated aqueous $NaHCO_3$ solution to neutralize the acidic reaction mixture and extracted with EtOAc for three times. The combined organic phases

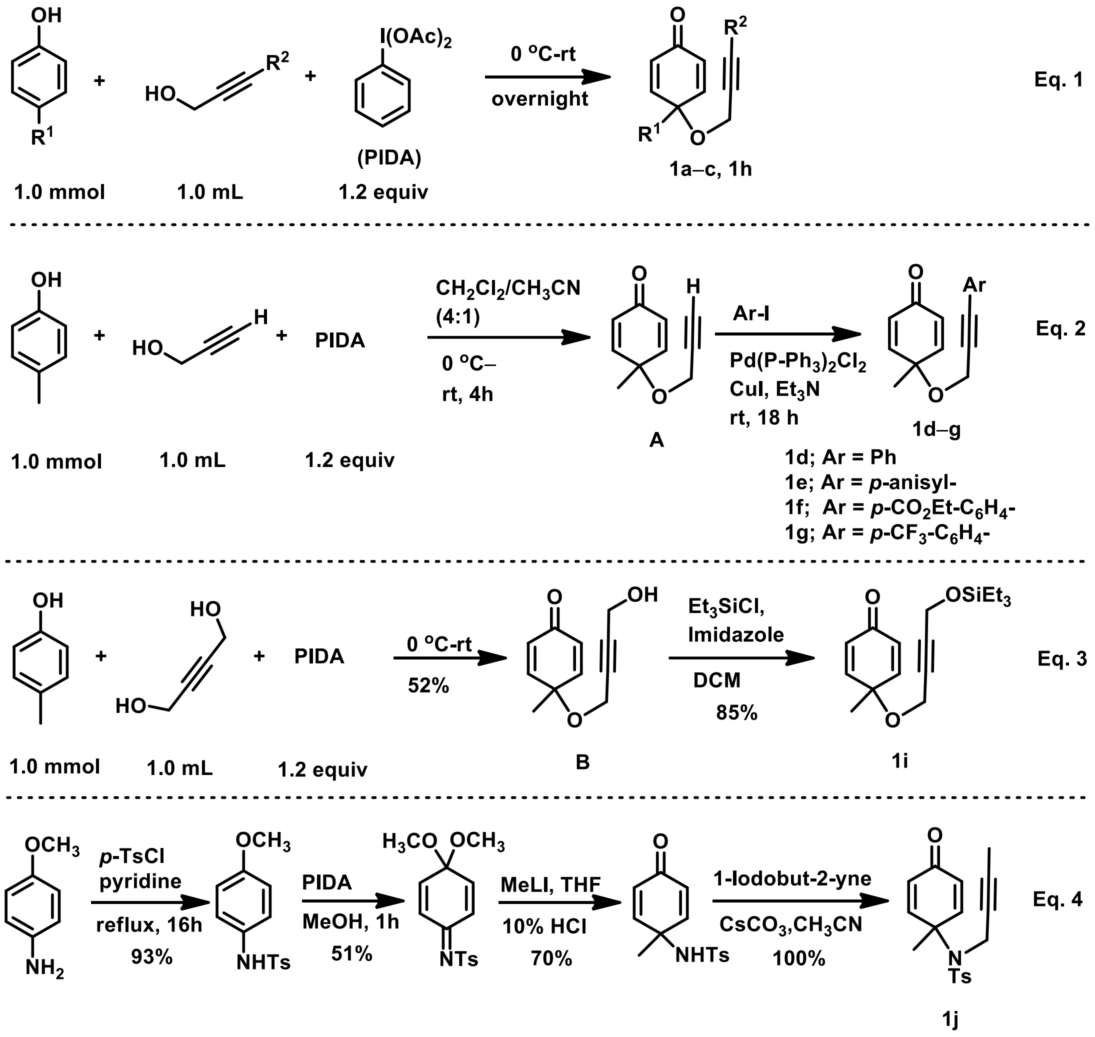

Supplementary Figure 1. Synthesis of Alkynyl-cyclohexadienone

were washed with brine, dried over Na_2SO_4, filtered and concentrated under reduced pressure. The residue was purified by column chromatography (5–10% EtOAc in hexane) to give pure alkynyl-cyclohexadienone **1**. Alkynyl-cyclohexadienones **1a**–**1e**, **1h**, and **1j** were synthesized following the reported procedures.[S2] Compounds **1f**, **1g**, and **1i** were prepared according to experimental procedures, shown below.

Ethyl 4-(3-((1-methyl-4-oxocyclohexa-2,5-dien-1-yl)oxy)prop-1-yn-1-yl)benzoate (1f): Alkynyl-cyclohexadienone **1f** was synthesized in two steps following the equation 2 in Supplementary Figure 1. **(**Diacetoxyiodo)benzene (5.8 g, 18.0 mmol) was added portion wise to a stirred solution of *p*-cresol (1.6 g, 15.0 mmol) in propargyl alcohol (15.0 mL) at 0 °C, the solution

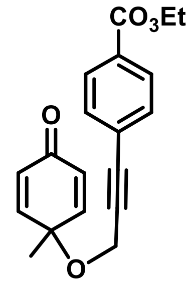

was warmed to room temperature and stirred for overnight. The reaction was quenched with saturated aqueous $NaHCO_3$ solution (40 mL) and extracted with EtOAc (3 × 30 mL). The combined organic phases were washed with brine (40 mL). The combined organic layers were dried over Na_2SO_4, filtered, and concentrated under reduced pressure. The residue was purified by column chromatography (10% EtOAc in hexane) to give 4-methyl-4-(prop-2-yn-1-yloxy)cyclohexa-2,5-dien-1-one (**A**; 0.97 g, 5.99 mmol, 40%) as a pale yellow solid. The spectral data of **A** is matched with that of reported previously.[8] An oven-dried Schlenk flask containing stirrer bar was charged with $Pd(PPh_3)_2Cl_2$ (39.1 mg, 0.06 mmol) and CuI (5.6 mg, 0.04 mmol) under N_2. A solution of alkyne-cyclohexadienone **A** (300.0 mg, 1.85 mmol) and ethyl 4-iodobenzoate (543.0 mg, 2.07 mmol) in Et_3N (10 mL) was added and the mixture was stirred at room temperature for 18 h. The mixture was diluted with EtOAc (25 mL) and washed with 10% aqueous HCl solution (2 × 25 mL). The combined organic layers were dried over Na_2SO_4, filtered and concentrated under reduced pressure. The residue was purified by silica gel flash chromatography (5–10% EtOAc in hexane) to give cyclohexadienone **1f** as a yellow amorphous solid (0.40 g, 1.3 mmol, 70%). R_f = 0.3 (20% EtOAc in hexane). **^{1}H NMR** (400 MHz, $CDCl_3$) δ 7.96 (2H, d, J = 8.0 Hz, Ar*H*), 7.45 (2H, d, J = 8.0 Hz, Ar*H*), 6.87 (2H, d, J = 9.2 Hz, CH=C*H*C), 6.34 (2H, d, J = 9.2 Hz, C*H*=CHC), 4.35 (2H, q, J = 7.6 Hz, OCH_2CH$_3$), 4.22 (2H, s, OCH_2C), 1.50 (3H, s, CCH_3), 1.37 (3H, t, J = 7.6 Hz, CH$_2$CH_3). **^{13}C{^{1}H} NMR** (100 MHz, $CDCl_3$) δ 185.0, 165.9, 150.7, 131.5, 130.5, 130.2, 129.4, 126.8, 88.5, 86.0, 73.3, 61.1, 54.4, 26.3, 14.2. **HRMS** (CI+): *m/z* Calcd for $C_{19}H_{19}O_4$: (M+H$^+$) 311.1283, found 311.1282.

4-Methyl-4-((3-(4-(trifluoromethyl)phenyl)prop-2-yn-1-yl)oxy)cyclohexa-2,5-dien-1-one (1g):

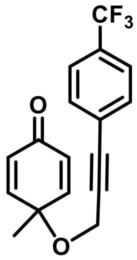

Alkynyl-cyclohexadienone **1g** was synthesized in two steps following the equation 2 in Supplementary Figure 1. First 4-methyl-4-(prop-2-yn-1-yloxy)cyclohexa-2,5-dien-1-one (**A**) was synthesized by following the above experimental procedure. An oven-dried Schlenk flask containing stirrer bar was charged with $Pd(PPh_3)_2Cl_2$ (25.1 mg, 0.03 mmol) and CuI (3.0 mg, 0.02 mmol) under N_2. A solution of alkyne-cyclohexadienone **A** (200.0 mg, 1.23 mmol) and 1-iodo-4-(trifluoromethyl)benzene (367.6 mg, 1.35 mmol) in Et_3N (10 mL) was added and the mixture was stirred at room temperature for 18 h. The mixture was diluted with EtOAc (20 mL) and washed with 10% aqueous HCl solution (2 × 20 mL). The combined organic layers were dried over Na_2SO_4, filtered and concentrated under reduced pressure. The residue was purified by silica gel

flash chromatography (5% EtOAc in hexane) to give **1g** as a yellow amorphous solid (250 mg, 0.82 mmol, 67%). R_f = 0.4 (20% EtOAc in hexane). **^{1}H NMR** (400 MHz, $CDCl_3$) δ 7.57 (2H, d, *J* = 8.4 Hz, Ar*H*), 7.51 (2H, d, *J* = 8.4 Hz, Ar*H*), 6.88 (2H, d, *J* = 9.2 Hz, CH=C*H*C), 6.35 (2H, d, *J* = 9.2 Hz, C*H*=CHC), 4.24 (2H, s, OCH_2C), 1.52 (3H, s, CCH_3). **^{13}C{^{1}H} NMR** (100 MHz, $CDCl_3$) δ 185.0, 150.7, 131.9, 130.6, 130.2, 125.1 (d, J_{CF} = 275.0 Hz), 125.2 (q, J_{CF} = 4.0 Hz), 88.1, 85.4, 73.3, 61.1, 54.4, 26.3. **HRMS** (CI+): *m/z* Calcd for $C_{17}H_{14}F_3O_2$: (M+H$^+$) 307.0946, found 307.0947.

4-Methyl-4-((4-((triethylsilyl)oxy)but-2-yn-1-yl)oxy)cyclohexa-2,5-dien-1-one (1i): Alkynyl-cyclohexadienone **1i** was synthesized in two steps following the equation 3 in Supplementary Figure 1. A flame-dried flask was charged with *p*-cresol (1.10 g, 10.0 mmol), CH_3CN (10 mL) and 1,4-butynediol (4.30 g, 50.0 mmol). A solution of $PhI(OAc)_2$ (4.80 g, 15.0 mmol, dissolved in 40 mL CH_2Cl_2) was added dropwise to the reaction pot over 2 h. The reaction mixture was allowed to stir at room temperature for 1 h. The solution was concentrated under vacuo and the residue was purified by column silica gel flash chromatography (50% EtOAc in hexane) to give pure alkynol-cyclohexadienone **B** as a yellow amorphous solid (1.0 g, 5.2 mmol, 52%). R_f = 0.2 (50% EtOAc in hexane). **^{1}H NMR** (400 MHz, $CDCl_3$) δ 6.82 (2H, d, *J* = 10.2 Hz, CH=C*H*C), 6.31 (2H, d, *J* = 10.2 Hz, C*H*=CHC), 4.29 (2H, br s, CH_2OH), 4.03 (2H, t, *J* = 1.9 Hz, OCH_2C), 1.47 (3H, s, CCH_3). The primary alcohol **B** (280.0 mg, 1.2 mmol) and imidazole (0.24 g, 3.5 mmol) were taken to two neck flask and dissolved in DCM (10 mL). Chlorotriethylsilane (0.39 g, 0.24 mmol) was then added dropwise to the stirring solution at 0 °C. The reaction mixture was allowed to warm to room temperature for overnight. The mixture was quenched with water (20 mL) and partioned by separating funnel. Aqueous layer was washed with DCM (2 x 20 mL). The combined organic layers were dried over Na_2SO_4, filtered and concentrated under reduced pressure. The residue was purified by silica gel flash chromatography (5% EtOAc in hexane) to give cyclohexadienone **1i** as a yellow amorphous solid (310.0 g, 1.1 mmol, 84%). R_f = 0.4 (10% EtOAc in hexane); **^{1}H NMR** (400 MHz, $CDCl_3$) δ 6.82 (2H, d, *J* = 10.2 Hz, CH=C*H*C), 6.29 (2H, d, *J* = 10.2 Hz, C*H*=CHC), 4.31 (2H, d, *J* = 1.8 Hz, CCH_2OSi), 4.01 (2H, d, *J* = 1.8 Hz, OCH_2C), 1.46 (3H, s, CCH_3), 0.96 (9H, t, *J* = 8.0 Hz, SiCH$_2$CH_3), 0.61 (6H, q, *J* = 8.0 Hz, SiCH_2CH$_3$). **^{13}C{^{1}H} NMR** (100 MHz, $CDCl_3$) δ 184.8, 150.7, 130.3, 85.3, 81.1, 73.0, 53.9, 51.2, 26.2, 6.5, 4.3. **HRMS** (EI): *m/z* Calcd for $C_{17}H_{26}O_3Si$: (M$^+$) 307.1729, found 307.1728.

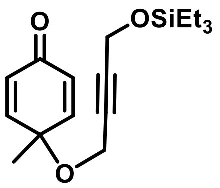

Evaluation of catalytic reaction conditions (Supplementary Table 1)

General method for the catalytic racemic reactions (Entries 1–4): In glove box, a vial was charged with phosphine (20 mol%) or IPr (10 mol%) and Ni(cod)$_2$ (2.75 mg, 0.01 mmol, 10 mol%) in C_6D_6 (0.5 mL). After 10 min of stirring, a mixture of 4-(but-2-yn-1-yloxy)-4-methylcyclohexa-2,5-dien-1-one (**1a**, 17.6 mg, 0.1 mmol) and (*E*)-3-(4-methoxyphenyl)-1-phenylprop-2-en-1-one (**2a**, 23.8 mg, 0.12 mmol) in C_6D_6 (0.5 mL) was

Supplementary Table 1. Evaluation of catalytic reaction conditions

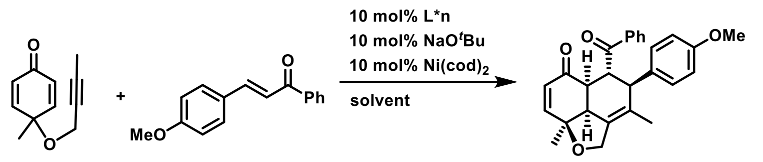

1a (1.2 equiv) + 2a → *Rac.* or (*S*,*S*,*R*,*R*,*S*)-3aa

entry	ligand	special conditions	sovent	base	temp (°C)	time (h)	yield (%)	ee (%)
1[a]	PCy_3	20 mol% PCy_3	C_6D_6	–	rt	72	0	–
2[a]	IPr	–	C_6D_6	–	rt	11	58	–
3	IPr	–	C_6D_6	–	rt	20	96	–
4	IPr	no Ni(cod)$_2$	C_6D_6	–	rt	24	0	–
5	L*1	–	C_6D_6	NaO^{t}Bu	rt	72	0	–
6	L*2	–	C_6D_6	NaO^{t}Bu	rt	72	0	–
7[b]	L*3	–	C_6D_6	NaO^{t}Bu	rt	72	13	94
8	L*4	–	C_6D_6	NaO^{t}Bu	rt	72	0	–
9	L*5	–	C_6D_6	NaO^{t}Bu	rt	72	0	–
10	L*6	–	C_6D_6	NaO^{t}Bu	rt	36	36	98
11	L*7	–	C_6D_6	NaO^{t}Bu	rt	36	48	89
12	L*6	–	benzene	KO^{t}Bu	rt	36	29	98
13	L*6	–	benzene	LiO^{t}Bu	rt	36	29	98
14	L*6	–	benzene	NaHMDS	rt	36	40	96
15	L*6	–	toluene	NaO^{t}Bu	rt	36	41	97
16	L*6	–	DMSO	NaO^{t}Bu	rt	36	7	96
17	L*6	–	CF_3-toluene	NaO^{t}Bu	rt	36	24	98
18	L*6	–	THF	NaO^{t}Bu	rt	36	30	97
19	L*6	–	DMF	NaO^{t}Bu	rt	36	8	97
20	L*6	–	o-Xylene	NaO^{t}Bu	rt	36	36	97
21	L*6	–	CPME	NaO^{t}Bu	rt	36	19	97
22	L*6	0.5 mL solvent	toluene	NaO^{t}Bu	rt	36	32	96
23	L*6	3.0 mL solvent	toluene	NaO^{t}Bu	60	36	51	98
24	L*6	5.0 mL solvent	toluene	NaO^{t}Bu	rt	36	68	99
25	L*6	10.0 mL solvent	toluene	NaO^{t}Bu	rt	36	67	99
26	L*6	5.0 mL solvent	toluene	NaO^{t}Bu	60	36	74	98
27	L*6	5.0 mL solvent, 5 mol% cat.	toluene	NaO^{t}Bu	60	36	61	98

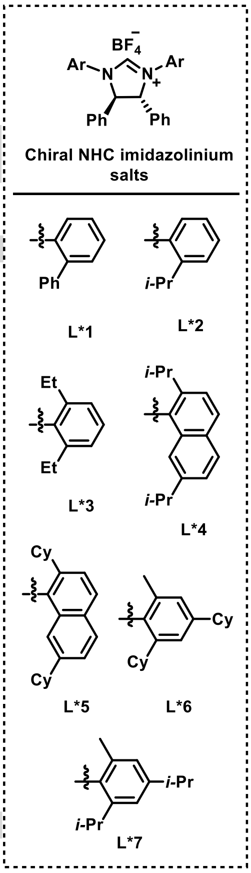

General conditions. Reaction was conducted with **1a** (0.12 mmol), **2a** (0.10 mmol), and solvent (1.0 mL). Isolated yields are given and enantioselectivity was determined by SFC equipped with a chiral stationary phase. [a]1.2 Equiv of **2a** was employed. [b]Yield and ee were measured at 27% conversion of **1a**. Cy = Cyclohexyl, CPME = Cyclopentylmethyl ether.

added and transferred to a J. Young NMR tube. The progress of reaction was monitored by ^{1}H NMR. After consumption of **1a**, reaction mixture was filtered through a small pad of silica, washed with diethylether, concentrated under vacuum. The residue was purified by silica gel flash chromatography (10% EtOAc in hexane) to get pure *rac*-**3aa** as a white amorphous solid. R_f = 0.3 (20% EtOAc in hexane). The structure of **3aa** was confirmed by 1D and 2D spectral analyses. IPr was effective ligand to afford *rac*-**3aa** in 58% yield (entry 2). However, 96% yield was obtained, when 1.2 equiv. of **1a** was used (entry 3).

Controlled Experiment: Reaction didn't proceed in the absence of Ni(cod)$_2$ (entry 4).

General method for the catalytic enantioselective reactions: A screw cap vial in the globe box was charged with **L*n**·HBF_4 (0.01 mmol, 10 mol%) and NaO^{t}Bu (0.01 mmol, 10 mol%) in toluene or C_6D_6 (0.5 mL). After 10 min of stirring, Ni(cod)$_2$ (0.01 mmol, 10 mol%) was added. To the stirring solution was added a mixture of 4-(but-2-yn-1-yloxy)-4-methylcyclohexa-2,5-dien-1-one (**1a**, 0.12 mmol) and (*E*)-3-(4-methoxyphenyl)-1-phenylprop-2-en-1-one (**2a**, 0.10 mmol) in toluene or C_6D_6 (0.5 mL) and stirred at room temperature for 36–72 h. The progress of reaction was monitored by TLC or NMR. After the consumption of **1a**, reaction mixture was filtered through a small pad of silica, washed with diethylether, and concentrated under vacuum. The residue was purified by silica gel flash chromatography (10% EtOAc in hexane) to get pure *enantioenriched*-**3aa** as a white amorphous solid. The absolute configuration of all five chiral centers in **3aa** was assigned by according to an analogy to **3ij**, determined unambiguously by X-ray crystallography (*vide infra*).

Entries 5–11: Screening of C_2-chiral NHC imidazolinium salts (**L*1–L*7**·HBF_4)
(**L*6**·HBF_4 gave the best enantioselectivity among them; entry 10).

Entries 12–14: Different bases were examined using **L*6**·HBF_4.

Entries 15–21: Different solvents were examined using **L*6**·HBF_4.

Entries 22–25: Reactions were studied at dilute conditions using **L*6**·HBF_4 to retard oligomerization of **1a**, which was a major side reaction in this transformation.

Optimized conditions for the study of scope and limitations (entry 26): To a screw cap vial in a glove box was added **L*6**·HBF_4 (16.3 mg, 0.02 mmol, 10 mol%) and NaO^{t}Bu (1.9 mg, 0.02 mmol, 10 mol%) in toluene (5 mL) and the suspension was allowed to stir at room temperature for 10 minutes and then Ni(cod)$_2$ (5.5 mg, 0.02 mmol, 10 mol%) was added. After further stirring for 10 minutes at room temperature was added a solution of alkynyl-cyclohexadienone (**1**, 0.24 mmol, 1.2 eq) and enone (**2**, 0.20 mmol) in toluene (10 mL). The reaction mixture was taken out of glove box

and heated at 60 ºC for 36 h with stirring. The reaction mixture was cooled to room temperature and filtered after the consumption of **1** and washed with Et_2O. The filtrate was concentrated in vacuo, and the residue was purified by silica gel flash chromatography (SiO_2, eluted with 5 to 20% ethyl acetate in hexane) to get pure *enantioenriched*-**3**.

Entry 27: Reaction was carried out with 5 mol% of **L*6**·HBF_4, NaO^{t}Bu, and Ni(cod)$_2$.

Spectral data of chiral tricyclic products 3

(2a^1*S*,4*S*,5*R*,5a*R*,8a*S*)-5-Benzoyl-4-(4-methoxyphenyl)-3,8a-dimethyl-2,2a^1,4,5,5a,8a-hexahydro-6*H*-naphtho[1,8-*bc*]furan-6-one (3aa): Following the general procedure, (*R*,*R*)-**L*6**·HBF_4 (16.0 mg, 0.02 mmol), NaO^{t}Bu (2.0 mg, 0.02 mmol), Ni(cod)$_2$ (5.5 mg, 0.02 mmol), 4-(but-2-yn-1-yloxy)-4-methylcyclohexa-2,5-dien-1-one (**1a**, 42.2 mg, 0.24 mmol) and (*E*)-3-(4-methoxyphenyl)-1-phenylprop-2-en-1-one (**2a**, 47.6 mg, 0.2 mmol) were used. Purification by silica gel flash column chromatography (10% EtOAc in hexane) gave **3aa** (61.0 mg, 0.157 mmol, 74% yield) as a white solid. R_f = 0.3 (20% EtOAc in hexane). **^{1}H NMR** (400 MHz, $CDCl_3$) δ 7.99 (2H, d, *J* = 7.6 Hz, benzoyl-*ortho*-Ar-*H*), 7.56 (1H, dd, *J* = 7.6, 7.6 Hz, benzoyl-*para*-Ar-*H*), 7.44 (2H, dd, *J* = 7.6, 7.6 Hz, benzoyl-*meta*-Ar-*H*), 6.88 (2H, d, *J* = 8.6 Hz, Ar-*H*), 6.79 (2H, d, *J* = 8.6 Hz, Ar-*H*), 6.58 (1H, dd, *J* = 10.2, 2.0 Hz, C*H*=CHCO), 5.98 (1H, d, *J* = 10.2 Hz, CH=C*H*CO), 4.88 (1H, br s, C*H*COPh), 4.57 (1H, d, *J* = 13.5 Hz, OCH_2C), 4.33 (1H, d, *J* = 13.5 Hz, OCH_2C), 3.80 (1H, br s, CC*H*-anisyl), 3.78 (3H, s, OCH_3), 2.93 (1H, dd, *J* = 5.8, 1.2 Hz, CH=CHCOC*H*CH), 2.76 (1H, s, CC*H*C=CH), 1.45 (3H, s, CCH_3), 1.39 (3H, s, C=CCH_3). **^{13}C{^{1}H} NMR** (100 MHz, $CDCl_3$) δ 201.5, 195.4, 158.2, 151.8, 135.6, 135.2, 133.7, 133.3, 130.1, 128.9, 128.8, 128.2, 126.6, 113.6, 80.1, 68.6, 55.1, 49.6, 44.2, 44.0, 42.9, 24.1, 18.1. **HRMS** (EI): *m/z* Calcd for $C_{27}H_{26}O_4$: (M^+) 414.1831, found 414.1835. $[\alpha]_D^{23}$ = (+) 177.3 (*c* = 0.11, in $CHCl_3$). **Chiral separation** (98% ee): The enantioselectivity was determined by SFC using Chiralpak IA (Back pressure = 15 MPa, Flow (CO_2) = 2.4 mL/min, Flow (isopropanol) = 0.6 mL/min, 25 ºC, λ = 250 nm). Retention time: t_R = 2.6 min (minor enantiomer) and 4.4 min (major enantiomer).

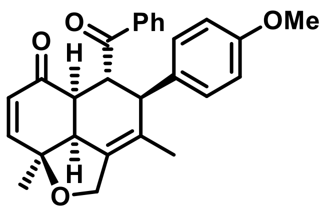

A half-gram scale reaction of **1a** (0.53 g, 3.0 mmol) was carried out with **2a** (0.6 g, 2.5 mmol), giving almost same results (0.76 g, 1.83 mmol, 73% yield, 98% ee).

(2a¹*S*,4*S*,5*R*,5a*R*,8a*S*)-5-Benzoyl-3,8a-dimethyl-4-phenyl-2,2a¹,4,5,5a,8a-hexahydro-6*H*-naphtho[1,8-*bc*]furan-6-one (3ab): Following the general procedure, (*R*,*R*)-**L*6**·HBF_4 (16.2 mg, 0.02 mmol), NaO*ᵗ*Bu (2.0 mg, 0.02 mmol), Ni(cod)$_2$ (5.4 mg, 0.02 mmol), 4-(but-2-yn-1-yloxy)-4-methyl- cyclohexa-2,5-dien- 1-one (**1a**, 42.0 mg, 0.24 mmol) and chalcone (**2b**, 41.6 mg, 0.2 mmol) were used. Purification by silica gel flash column chromatography (5% EtOAc in hexane) gave **3ab** (56.0 mg, 0.145 mmol, 73% yield) as a white amorphous solid. R_f = 0.4 (20% EtOAc in hexane). **^{1}H NMR** (400 MHz, $CDCl_3$) δ 7.99 (2H, d, *J* = 7.6 Hz, benzoyl-*ortho*-Ar-*H*), 7.55 (1H, dd, *J* = 7.6, 7.6 Hz, benzoyl-*para*-Ar-*H*), 7.43 (2H, dd, *J* = 7.6, 7.6 Hz, benzoyl-*meta*-Ar-*H*), 7.17–7.27 (3H, m, Ar-*H*), 6.97 (2H, d, *J* = 7.6 Hz, Ar-*H*), 6.58 (1H, dd, *J* = 10.2, 2.0 Hz, C*H*=CHCO), 5.98 (1H, d, *J* = 10.2 Hz, CH=C*H*CO), 4.90 (1H, s, C*H*COPh), 4.57 (1H, d, *J* = 13.2 Hz, OC*H*$_2$C), 4.34 (1H, d, *J* = 13.2 Hz, OC*H*$_2$C), 3.86 (1H, s, CC*H*-Ph), 2.95 (1H, d, *J* = 5.2 Hz, CH=CHCOC*H*CH), 2.76 (1H, s, CC*H*C=CH), 1.44 (3H, s, CC*H*$_3$), 1.41 (3H, s, C=CC*H*$_3$). **^{13}C{^{1}H} NMR** (100 MHz, $CDCl_3$) δ 201.3, 195.2, 151.7, 143.5, 135.2, 133.9, 133.3, 129.1, 128.9, 128.8, 128.3, 128.2, 126.6, 126.3, 80.1, 68.5, 49.5, 44.9, 44.1, 42.9, 24.1, 18.1. **HRMS** (EI): *m/z* Calcd for $C_{26}H_{24}O_3$: (M^+) 384.1725, found 384.1726. $[\alpha]_D^{23}$ = (+) 204.6 (*c* = 0.17, in $CHCl_3$). **Chiral separation** (98% ee): The enantioselectivity was determined by SFC using Chiralpak IA (Back pressure = 15 MPa, Flow (CO_2) = 3.0 mL/min, Flow (isopropanol) = 0.3 mL/min, 25 °C, λ = 250 nm). Retention time: t_R = 3.8 min (minor enantiomer) and 6.1 min (major enantiomer).

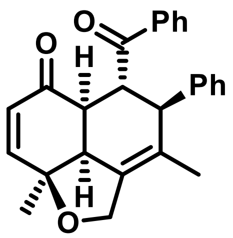

(2a¹*S*,4*S*,5*R*,5a*R*,8a*S*)-5-Benzoyl-4-(4-fluorophenyl)-3,8a-dimethyl-2,2a¹,4,5,5a,8a-hexahydro-6*H*-naphtho[1,8-*bc*]furan-6-one (3ac): Following the general procedure, (*R*,*R*)-**L*6**·HBF_4 (16.4 mg, 0.02 mmol), NaO*ᵗ*Bu (2.0 mg, 0.02 mmol), Ni(cod)$_2$ (5.5 mg, 0.02 mmol), 4-(but-2-yn-1-yloxy)-4-methylcyclohexa-2,5-dien- 1-one (**1a**, 42.2 mg, 0.24 mmol) and (*E*)-3-(4-fluorophenyl)-1-phenylprop-2-en-1-one (**2c**, 45.2 mg, 0.2 mmol) were used. Purification by silica gel flash column chromatography (5% EtOAc in hexane) gave **3ac** (58.0 mg, 0.144 mmol, 72% yield) as thick oil. R_f = 0.4 (20% EtOAc in hexane).**^{1}H NMR** (400 MHz, $CDCl_3$) δ 7.98 (2H, d, *J* = 7.6 Hz, benzoyl-*ortho*-Ar-*H*), 7.57 (1H, dd, *J* = 7.6, 7.6 Hz, benzoyl-*para*-Ar-*H*), 7.45 (2H, dd, *J* = 7.6, 7.6 Hz, benzoyl-*meta*-Ar-*H*), 6.94 (4H, d, *J* = 7.6 Hz, Ar-*H*), 6.59 (1H, d, *J* = 10.2 Hz, C*H*=CHCO), 5.98 (1H, d, *J* = 10.2 Hz, CH=C*H*CO), 4.87 (1H, s, C*H*COPh), 4.58 (1H, d, *J* = 13.2 Hz, OC*H*$_2$C), 4.34 (1H, d, *J* = 13.2 Hz, OC*H*$_2$C), 3.86 (1H, s, CC*H*Ar), 2.95 (1H, d, *J* = 5.2 Hz,

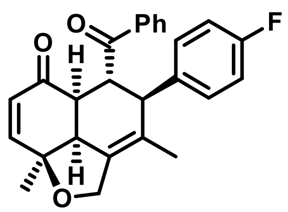

CH=CHCOC*H*CH), 2.74 (1H, s, CC*H*C=CH), 1.45 (3H, s, CCH_3), 1.39 (3H, s, C=CCH_3). **^{13}C{^{1}H} NMR** (100 MHz, $CDCl_3$) δ 201.1, 195.3, 151.9, 135.1, 134.2, 133.5, 130.6, 130.5, 128.9, 128.8, 128.2, 126.1, 115.3, 115.1, 80.1, 68.5, 49.5, 44.12, 44.08, 43.0, 24.0, 18.1. **HRMS** (EI): *m/z* Calcd for $C_{26}H_{23}FO_3$: (M^+) 402.1631, found 402.1628. $[\alpha]_D^{20}$ = (+) 135.3 (*c* = 0.13, in $CHCl_3$). **Chiral separation** (98% ee): The enantioselectivity was determined by SFC using Chiralpak IA (Back pressure = 15 MPa, Flow (CO_2) = 3.0 mL/min, Flow (isopropanol) = 0.3 mL/min, 25 °C, λ = 250 nm). Retention time: t_R = 3.3 min (minor enantiomer) and 4.7 min (major enantiomer).

(2a^1*S*,4*S*,5*R*,5a*R*,8a*S*)-5-benzoyl-4-(4-fluorophenyl)-3,8a-dimethyl-2,2a1,4,5,5a,8a-hexahydro-6H-naphtho[1,8-bc]furan-6-one (3ad): Following the general procedure, (*R*,*R*)-**L*6**·HBF_4 (8.1 mg, 0.01 mmol), and NaO^{t}Bu (1.0 mg, 0.01 mmol), Ni(cod)$_2$ (2.76 mg, 0.01 mmol), 4-(but-2-yn-1-yloxy)-4-methylcyclohexa-2,5-dien- 1-one (**1a**, 21.0 mg, 0.12 mmol), and (*E*)-1-(furan-2-yl)-3-(p-tolyl)prop-2-en-1-one (**2d**, 21.0 mg, 0.1 mmol), were used. Purification by column chromatography (15% EtOAc in hexane) gave **3ad** (28.0 mg, 0.072 mmol, 72%) as colorless oil. R_f = 0.3 (20% EtOAc in hexane). **^{1}H NMR** (400 MHz, $CDCl_3$): δ 7.59 (1H, d, 1.0 Hz, Furyl-*H*), 7.23 (1H, d, *J* = 3.5 Hz, Furyl-Ar-*H*), 7.06 (2H, d, *J* = 7.9 Hz, Ar-*H*), 6.87 (2H, d, *J* = 7.9 Hz, Ar-*H*), 6.58 (1H, dd, *J* = 10.2, 1.7 Hz, C*H*=CHCO), 6.50 (1H, dd, *J* = 3.5, 1.7 Hz, Furyl-Ar-*H*), 5.97 (1H, d, *J* = 10.2 Hz, CH=C*H*CO), 4.61 (1H, dd, *J* = 2.5, 1.7 Hz, C*H*COPh), 4.57 (1H, d, *J* = 13.1 Hz, OCH_2C), 4.33 (1H, d, *J* = 13.1 Hz, OCH_2C), 3.75 (1H, br s, CC*H*Ar), 2.94 (1H, dd, *J* = 5.9, 1.4 Hz, CH=CHCOC*H*CH), 2.88 (1H, br s, CC*H*C=CH), 2.30 (3H, s, Ar-CH_3), 1.49 (3H, s, CCH_3), 1.38 (3H, s, C=CCH_3). **^{13}C NMR** (100 MHz, $CDCl_3$): δ 195.1, 190.2, 151.5, 151.2, 147.0, 140.2, 136.0, 134.1, 128.9, 128.2, 126.2, 118.9, 112.3, 80.1, 68.5, 49.9, 44.2, 44.1, 43.2, 24.1, 21.0, 18.1. **HRMS** (EI+): *m/z* Calcd for $C_{25}H_{24}O_4$: (M^+) 388.1675, found 388.1678. $[\alpha]_D^{20}$ = (+) 160.3 (*c* = 0.24, in $CHCl_3$). **Chiral separation** (99% ee): The enantioselectivity was determined by SFC using Chiralpak IA (Back pressure = 15 MPa, Flow (CO_2) = 3.0 mL/min, Flow (isopropanol) = 0.3 mL/min, 40 °C, λ = 250 nm). Retention time: t_R = 4.6 min (minor enantiomer) and 5.0 min (major enantiomer).

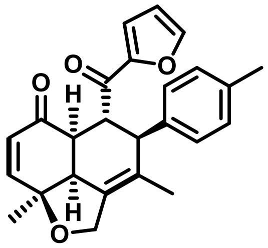

(2a^1*S*,4*S*,5*R*,5a*R*,8a*S*)-5-Benzoyl-3,4,8a-trimethyl-2,2a1,4,5,5a,8a-hexahydro-6*H*-naphtho[1,8-*b c*]furan-6-one (3ae): Following the general procedure, (*R*,*R*)-**L*6**·HBF_4 (8.2 mg, 0.01 mmol), and NaO^{t}Bu (1.0 mg, 0.01 mmol), Ni(cod)$_2$ (2.75 mg, 0.01 mmol), 4-(but-2-yn-1-yloxy)-4-methylcyclohexa-2,5-dien- 1-one (**1a**, 21.0 mg, 0.12 mmol), and

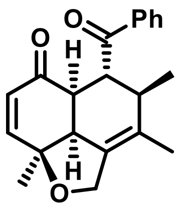

(E)-1-phenylbut-2-en-1-one (**2e**, 14.8 mg, 0.1 mmol), were used. Purification by column chromatography (5% EtOAc in hexane) gave **3ad** (20.0 mg, 0.062 mmol, 62%) as colorless oil. R_f = 0.3 (20% EtOAc in hexane). **^{1}H NMR** (400 MHz, $CDCl_3$): δ 8.04 (2H, d, 7.6 Hz, benzoyl-*ortho*-Ar-*H*), 7.59 (1H, dd, *J* = 7.2, 7.2 Hz, benzoyl-*para*-Ar-*H*), 7.49 (2H, dd, *J* = 8.0, 8.0 Hz, benzoyl-*meta*-Ar-*H*), 6.52 (1H, dd, *J* = 10.0, 2.0 Hz, C*H*=CHCO), 5.91 (1H, d, *J* = 10.0 Hz, CH=C*H*CO), 4.59 (1H, dd, *J* = 1.6, 1.6 Hz, C*H*COPh), 4.49 (1H, d, *J* = 13.2 Hz, OCH_2C), 4.22 (1H, d, *J* = 13.2 Hz, OCH_2C), 2.94 (1H, dd, *J* = 6.0, 1.8 Hz, CH=CHCOC*H*CH), 2.72 (1H, br s, CC*H*C=CH), 2.60 (1H, br s, CC*H*CH_3), 1.58 (3H, br s, C=CCH_3), 1.41 (3H, s, CCH_3), 1.12 (3H, d, *J* = 7.6 Hz, CHCH_3). **^{13}C NMR** (100 MHz, $CDCl_3$): δ 202.0. 196.3, 151.9, 135.6, 133.2, 131.2, 128.9, 128.5, 128.3, 128.0, 80.0, 68.6, 48.2, 43.9, 42.2, 33.4, 24.0, 20.6, 17.2. **HRMS** (EI+): *m/z* Calcd for $C_{21}H_{22}O_3$: (M^+) 322.1569, found 322.1571. $[\alpha]_D^{20}$ = (+) 73.0 (*c* = 0.34, in $CHCl_3$). **Chiral separation** (97% ee): The enantioselectivity was determined by SFC using Chiralpak IA (Back pressure = 15 MPa, Flow (CO_2) = 2.4 mL/min, Flow (isopropanol) = 0.6 mL/min, 25 °C, λ = 250 nm). Retention time: t_R = 2.0 min (minor enantiomer) and 2.5 min (major enantiomer).

The fully-intermolecular [2+2+2] cycloaddition product (**3ae'**) was eluted with 15% EtOAc in hexane (3.8 mg). R_f = 0.2 (20% EtOAc in hexane). **^{1}H NMR** (400 MHz, $CDCl_3$): δ 7.99 (2H, d, *J* = 8.2 Hz, benzoyl-*ortho*-Ar-*H*), 7.86 (2H, d, *J* = 8.2 Hz, benzoyl-*ortho*-Ar-*H*), 7.42–7.59 (6H, m, Ph-*H*), 6.86 (2H, td, *J* = 2.5, 10.2 Hz, C*H*=CHCO), 6.30 (2H, d, *J* = 10.2 Hz, CH=C*H*CO), 4.12 (1H, br s, C=CC*H*COPh), 3.94 (2H, br s, OCH_2C), 3.86 (1H, dd, *J* = 3.4, 6.0 Hz, CHC*H*COPh), 2.98 (1H, t, *J* = 6.8 Hz, C=CC*H*CH_3), 2.51–2.54 (1H, m, CHC*H*CH_3), 1.60 (3H, d, *J* = 1.6 Hz, C=CCH_3), 1.42 (3H, s, CCH_3), 1.19 (3H, d, *J* = 7.3 Hz, CHCHCH_3), 0.96 (3H, d, *J* = 7.3 Hz, C=CCHCH_3). **^{13}C NMR** (100 MHz, $CDCl_3$): δ 203.7. 202.5, 185.3, 152.4, 152.3, 138.6, 137.2, 133.5, 133.3, 132.7, 130.0, 129.8, 129.3, 128.8, 128.6, 128.4, 128.0, 72.5, 63.5, 56.0, 46.3, 34.4, 33.1, 26.5, 19.3, 18.7, 15.7. **HRMS** (EI+): *m/z* Calcd for $C_{31}H_{32}O_4$: (M^+) 468.2301, found 468.2306.

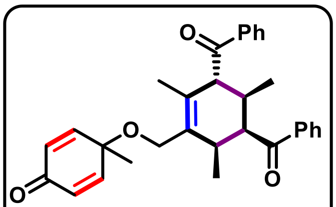

3ae'

(2a^1*S*,4*S*,5*R*,5a*R*,8a*S*)-(4-Methoxybenzoyl)-3,4,8a-trimethyl-2,2a^1,4,5,5a,8a-hexahydro-6*H*-naphtho[1,8-*bc*]furan-6-one (3af): Following the general procedure, (*R*,*R*)-**L*6**·HBF_4 (8.1 mg, 0.01 mmol), NaO^{t}Bu (1.0 mg, 0.01 mmol), Ni(cod)$_2$ (2.75 mg, 0.01 mmol), 4-(but-2-yn-1-yloxy)-4-methylcyclohexa-2,5-dien- 1-one (**1a**, 21.0 mg, 0.12 mmol) and(E)-1-(4-methoxyphenyl)but-2-en-1-one (**2f**, 17.6 mg, 0.1 mmol) were used. Purification by

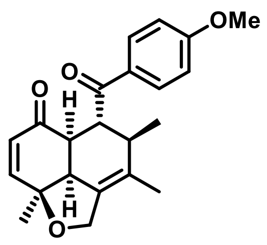

silica gel flash column chromatography (5–10% EtOAc in hexane) gave **3af** (22.9 mg, 0.065 mmol, 65% yield) as thick oil. R_f = 0.3 (20% EtOAc in hexane). **^{1}H NMR** (400 MHz, $CDCl_3$): δ 8.03 (2H, d, 8.3 Hz, benzoyl-*ortho*-Ar-*H*), 6.96 (2H, d, *J* = 8.3 Hz, benzoyl-*meta*-Ar-*H*), 6.52 (1H, d, *J* = 10.4 Hz, C*H*=CHCO), 5.90 (1H, d, *J* = 10.4 Hz, CH=C*H*CO), 4.54 (1H, s, C*H*COPh), 4.51 (1H, d, *J* = 12.8 Hz, OCH_2C), 4.21 (1H, d, *J* = 12.8 Hz, OCH_2C), 3.87 (3H, s, OCH_3), 2.90 (1H, d, *J* = 6.1 Hz, CH=CHCOC*H*CH), 2.73 (1H, br s, CC*H*C=CH), 2.59 (1H, br s, CC*H*CH_3), 1.57 (3H, br s, CCH_3), 1.41 (3H, s, C=CCH_3), 1.10 (3H, d, *J* = 7.8 Hz, CHCH_3). **^{13}C NMR** (100 MHz, $CDCl_3$): δ 200.5. 196.5, 163.6, 151.9, 131.2, 130.9 (two carbons), 128.4, 128.0, 114.0, 80.0, 68.6, 55.5, 47.8, 43.9, 42.6, 33.5, 24.0, 20.6, 17.2. **HRMS** (EI+): *m/z* Calcd for $C_{22}H_{24}O_4$: (M^+) 352.1675, found 352.1672. $[\alpha]_D^{20}$ = (+) 91.6 (*c* = 0.14, in $CHCl_3$). **Chiral separation** (94% ee): The enantioselectivity was determined by SFC using Chiralpak IA (Back pressure = 15 MPa, Flow (CO_2) = 2.4 mL/min, Flow (isopropanol) = 0.6 mL/min, 25 °C, λ = 250 nm). Retention time: t_R = 2.4 min (minor enantiomer) and 3.6 min (major enantiomer).

(2a^1*S*,4*S*,5*R*,5a*R*,8a*S*)-3,4,8a-Trimethyl-5-(4-methylbenzoyl)-2,2a^1,4,5,5a,8a-hexahydro-6*H*-naphtho[1,8-*bc*]furan-6-one (3ag): Following the general procedure, (*R*,*R*)-**L*6**·HBF_4 (16.2 mg, 0.02 mmol), NaO^{t}Bu (1.9 mg, 0.02 mmol), Ni(cod)$_2$ (5.5 mg, 0.02 mmol), 4-(but-2-yn-1-yloxy)-4-methylcyclohexa-2,5-dien- 1-one (**1a**, 42.2 mg, 0.24 mmol) and (*E*)-1-(*p*-tolyl)but-2-en-1-one (**2g**, 32.0 mg, 0.2 mmol) were used. Purification by silica gel flash column chromatography (5% EtOAc in hexane) gave **3ag** (45.0 mg, 0.134 mmol, 67% yield) as thick oil. R_f = 0.3 (20% EtOAc in hexane). **^{1}H NMR** (400 MHz, $CDCl_3$): δ 7.94 (2H, d, *J* = 7.6 Hz, benzoyl-*ortho*-Ar-*H*), 7.28 (2H, d, *J* = 7.6 Hz, benzoyl-*meta*-Ar-*H*), 6.51 (1H, d, *J* = 10.0 Hz, C*H*=CHCO), 5.90 (1H, d, *J* = 10.4 Hz, CH=C*H*CO), 4.56 (1H, s, C*H*COPh), 4.48 (1H, dd, *J* = 12.6 Hz, OCH_2C), 4.21 (1H, d, *J* = 12.6 Hz, OCH_2C), 2.92 (1H, d, *J* = 5.6 Hz, CH=CHCOC*H*CH), 2.72 (1H, br s, CC*H*C=CH), 2.59 (1H, br s, CC*H*CH_3), 2.42 (3H, s, Ar-CH_3), 1.57 (3H, br s, CCH_3), 1.40 (3H, s, C=CCH_3), 1.11 (3H, d, *J* = 7.0 Hz, CHCH_3). **^{13}C NMR** (100 MHz, $CDCl_3$): δ 201.7, 196.5, 151.9, 144.0, 133.0, 131.2, 129.5, 128.7, 128.4, 128.0, 80.0, 68.6, 48.1, 43.9, 42.3, 33.5, 24.1, 21.6, 20.6, 17.2. **HRMS** (EI+): *m/z* Calcd for $C_{22}H_{24}O_3$: (M^+) 336.1725, found 336.1720. $[\alpha]_D^{20}$ = (+) 96.3 (*c* = 0.13, in $CHCl_3$). **Chiral separation** (95% ee): The enantioselectivity was determined by SFC using Chiralpak IA (Back pressure = 15 MPa, Flow (CO_2) = 3.0 mL/min, Flow (isopropanol) = 0.3 mL/min, 25 °C, λ = 250 nm). Retention time: t_R = 3.6 min (minor enantiomer) and 5.4 min (major

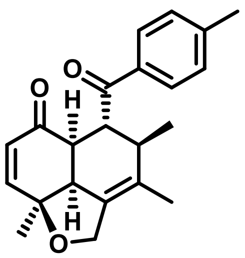

enantiomer).

(2a^1*S*,4*S*,5*R*,5a*R*,8a*S*)-(4-Fluorobenzoyl)-3,4,8a-trimethyl-2,2a^1,4,5,5a,8a-hexahydro-6*H*-naphtho[1,8-*bc*]furan-6-one (3ah): Following the general procedure, (*R*,*R*)-**L*6**·HBF_4 (16.4 mg, 0.02 mmol), NaO^{t}Bu (2.0 mg, 0.02 mmol), Ni(cod)$_2$ (5.5 mg, 0.02 mmol), 4-(but-2-yn-1-yloxy)-4-methylcyclohexa-2,5-dien- 1-one (**1a**, 42.2 mg, 0.24 mmol) and (*E*)-1-(4-fluorophenyl)but-2-en-1-one (**2h**, 32.8 mg, 0.2 mmol) were used. Purification by silica gel flash column chromatography (5% EtOAc in hexane) gave **3ah** (46.0 mg, 0.135 mmol, 68% yield) as thick oil. R_f = 0.3 (20% EtOAc in hexane). **^{1}H NMR** (400 MHz, $CDCl_3$): δ 8.05–8.09 (2H, m, benzoyl-*ortho*-Ar-*H*), 7.12–7.18 (2H, m, benzoyl-*meta*-Ar-*H*), 6.53 (1H, dd *J* = 10.5, 1.5 Hz, C*H*=CHCO), 5.91 (1H, d, *J* = 10.5 Hz, CH=C*H*CO), 4.54 (1H, s, C*H*COPh), 4.51 (1H, dd, *J* = 13.4 Hz, OC*H*$_2$C), 4.21 (1H, d, *J* = 13.4 Hz, OC*H*$_2$C), 2.89 (1H, d, *J* = 5.7 Hz, CH=CHCOC*H*CH), 2.71 (1H, br s, CC*H*C=CH), 2.59 (1H, d, *J* = 3.4 Hz, CC*H*CH$_3$), 1.58 (3H, br s, CC*H*$_3$), 1.42 (3H, s, C=CC*H*$_3$), 1.11 (3H, d, *J* = 6.7 Hz, CHC*H*$_3$). **^{13}C NMR** (100 MHz, $CDCl_3$): δ 200.4, 196.2, 165.8 (d, J_{CF} = 255.6 Hz), 152.1, 132.0, 131.3, 128.3, 128.0, 116.1, 115.7 (d, J_{CF} = 20.1 Hz), 80.0, 68.6, 48.2, 43.9, 42.3, 33.5, 24.1, 20.6, 17.2. **HRMS** (EI+): *m/z* Calcd for $C_{21}H_{21}FO_3$: (M^+) 340.1475, found 340.1474. $[\alpha]_D^{20}$ = (+) 86.4 (*c* = 0.15, in $CHCl_3$). **Chiral separation** (95% ee): The enantioselectivity was determined by SFC using Chiralpak IA (Back pressure = 15 MPa, Flow (CO_2) = 3.0 mL/min, Flow (isopropanol) = 0.3 mL/min, 25 °C, λ = 250 nm). Retention time: t_R = 2.7 min (minor enantiomer) and 3.3 min (major enantiomer).

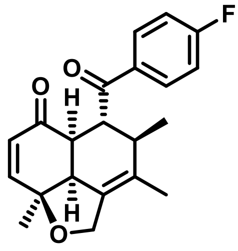

(2a^1*S*,4*S*,5*R*,5a*R*,8a*S*)-(3-Fluorobenzoyl)-3,4,8a-trimethyl-2,2a^1,4,5,5a,8a-hexahydro-6*H*-naphtho[1,8-*bc*]furan-6-one (3ai): Following the general procedure, (*R*,*R*)-**L*6**·HBF_4 (16.0 mg, 0.02 mmol), NaO^{t}Bu (2.0 mg, 0.02 mmol), Ni(cod)$_2$ (5.5 mg, 0.02 mmol), 4-(but-2-yn-1-yloxy)-4-methylcyclohexa-2,5-dien- 1-one (**1a**, 42.0 mg, 0.24 mmol) and (*E*)-1-(3-fluorophenyl)but-2-en-1-one (**2i**, 32.5 mg, 0.2 mmol) were used. Purification by silica gel flash column chromatography (5% EtOAc in hexane) gave **3ai** (47.0 mg, 0.14 mmol, 70% yield) as thick oil. R_f = 0.3 (20% EtOAc in hexane). **^{1}H NMR** (400 MHz, $CDCl_3$): δ 7.82 (1H, d, *J* = 8.0 Hz,, benzoyl-*ortho*-Ar*H*), 7.70 (1H, d, *J* = 8.0 Hz,, benzoyl-*ortho*-Ar*H*), 7.47 (1H, dd, *J* = 8.0, 8.0 Hz,, benzoyl-*meta*-Ar*H*), 7.29 (1H, dd, *J* = 8.0, 8.0 Hz, benzoyl-*para*-Ar*H*), 6.52 (1H, dd *J* = 10.0, 1.8 Hz, C*H*=CHCO), 5.91 (1H, d, *J* = 10.0 Hz, CH=C*H*CO), 4.52 (1H, s, C*H*COPh), 4.50 (1H,

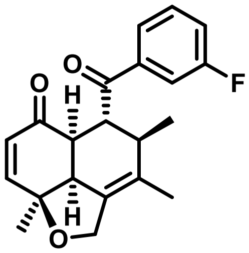

d, J = 12.7 Hz, OCH_2C), 4.21 (1H, d, J = 12.7 Hz, OCH_2C), 2.91 (1H, d, J = 5.7 Hz, CH=CHCOC*H*CH), 2.71 (1H, br s, CC*H*C=CH), 2.58 (1H, d, J = 5.7 Hz, CC*H*CH_3), 1.57 (3H, br s, CCH_3), 1.42 (3H, s, C=CCH_3), 1.11 (3H, d, J = 7.2 Hz, CHCH_3). **^{13}C NMR** (100 MHz, $CDCl_3$): δ 200.8, 196.1, 163.1 (d, J_{CF} = 248.6 Hz), 152.0, 137.8 (d, J_{CF} = 6.7 Hz), 131.3, 130.6 (d, J_{CF} = 8.7 Hz), 128.2, 128.0, 124.2 (d, J_{CF} = 2.8 Hz), 120.3 (d, J_{CF} = 22.2 Hz), 115.3 (d, J_{CF} = 22.2 Hz), 80.0, 68.6, 48.5, 43.9, 42.1, 33.4, 24.1, 20.6, 17.2. **HRMS** (EI+): *m/z* Calcd for $C_{21}H_{21}FO_3$: (M^+) 340.1475, found 340.1478. $[\alpha]_D^{20}$ = (+) 84.4 (c = 0.14, in $CHCl_3$). **Chiral separation** (97% ee): The enantioselectivity was determined by SFC using Chiralpak IA (Back pressure = 15 MPa, Flow (CO_2) = 4.5 mL/min, Flow (isopropanol) = 0.2 mL/min, 25 °C, λ = 250 nm). Retention time: t_R = 3.2 min (minor enantiomer) and 3.6 min (major enantiomer).

(2a^1*S*,4*S*,5*R*,5a*R*,8a*S*)-5-Benzoyl-8a-ethyl-3-methyl-4-phenyl-2,2a^1,4,5,5a,8a-hexahydro-6*H*-naphtho[1,8-*bc*]furan-6-one (3bb): Following the general procedure, (*R*,*R*)-**L*6**·HBF_4 (16.4 mg, 0.02 mmol), NaO^{t}Bu (2.0 mg, 0.02 mmol), Ni(cod)$_2$ (5.5 mg, 0.02 mmol), 4-(but-2-yn-1-yloxy)-4-ethylcyclohexa-2,5-dien-1-one (**1b**, 45.2 mg, 0.24 mmol) and chalcone (**2b**, 41.6 mg, 0.2 mmol) were used. Purification by silica gel flash column chromatography (5% EtOAc in hexane) gave **3bb** (58.0 mg, 0.146 mmol, 73% yield) as thick oil. R_f = 0.3 (20% EtOAc in hexane). **^{1}H NMR** (400 MHz, $CDCl_3$) δ 7.96 (2H, d, J = 7.6 Hz, benzoyl-*ortho*-Ar-*H*), 7.55 (1H, dd, J = 7.6, 7.6 Hz, benzoyl-*para*-Ar-*H*), 7.43 (2H, dd, J = 7.6, 7.6 Hz, benzoyl-*meta*-Ar-*H*), 7.20–7.28 (3H, m, Ar-*H*), 6.98 (2H, d, J = 7.6 Hz, Ar-*H*), 6.59 (1H, dd, J = 10.2, 2.0 Hz, C*H*=CHCO), 6.05 (1H, d, J = 10.2 Hz, CH=C*H*CO), 4.87 (1H, s, C*H*COPh), 4.57 (1H, d, J = 12.6 Hz, OCH_2C), 4.34 (1H, d, J = 12.6 Hz, OCH_2C), 3.85 (1H, s, CC*H*-Ph), 2.97 (1H, d, J = 5.2 Hz, CH=CHCOC*H*CH), 2.83 (1H, br s, CC*H*C=CH), 1.74–1.84 (2H, m, C$H_2$$CH_3$), 1.41 (3H, s, C=C$CH_3$), 0.92 (3H, t, J = 7.7 Hz, CH_2CH_3). **^{13}C{^{1}H} NMR** (100 MHz, $CDCl_3$) δ 201.5, 195.6, 150.4, 143.4, 135.3, 134.1, 133.3, 129.2, 129.1, 128.8, 128.7, 128.3, 126.6, 126.1, 82.7, 68.4, 49.6, 44.9, 43.4, 42.0, 31.1, 18.1, 8.0. **HRMS** (EI): *m/z* Calcd for $C_{27}H_{26}O_3$: (M^+) 384.1725, found 384.1726. $[\alpha]_D^{23}$ = (+) 170.8 (c = 0.17, in $CHCl_3$). **Chiral separation** (99% ee): The enantioselectivity was determined by SFC using Chiralpak IA (Back pressure = 15 MPa, Flow (CO_2) = 3.0 mL/min, Flow (isopropanol) = 0.3 mL/min, 25 °C, λ = 250 nm). Retention time: t_R = 3.8 min (minor enantiomer) and 5.6 min (major enantiomer).

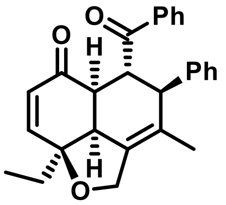

(2a^1*S*,4*S*,5*R*,5a*R*,8a*S*)-5-Benzoyl-8a-ethyl-3-methyl-4-propyl-2,2a^1,4,5,5a,8a-hexahydro-6*H*-nap

htho[1,8-*bc*]furan-6-one (3bj): Following the general procedure, (*R*,*R*)-**L*6**·HBF_4 (16.0 mg, 0.02 mmol), NaO*t*Bu (2.0 mg, 0.02 mmol), Ni(cod)$_2$ (5.5 mg, 0.02 mmol), 4-(but-2-yn-1-yloxy)-4-ethylcyclohexa-2,5-dien-1-one (**1b**, 45.6 mg, 0.24 mmol) and (*E*)-1-phenylhex-2-en-1-one (**2j**, 34.2 mg, 0.2 mmol) were used. Purification by silica gel flash column chromatography (5% EtOAc in hexane) gave **3bj** (48.0 mg, 0.132 mmol, 65% yield) as thick oil. R_f = 0.4 (20% EtOAc in hexane). **^{1}H NMR** (400 MHz, $CDCl_3$): δ 8.03 (2H, d, *J* = 7.6 Hz, benzoyl-*ortho*-Ar-*H*), 7.57 (1H, dd, *J* = 7.6, 7.6 Hz, benzoyl-*para*-Ar-*H*), 7.50 (2H, dd, *J* = 7.6, 7.6 Hz, benzoyl-*meta*-Ar-*H*), 6.51 (1H, dd, *J* = 10.1, 2.0 Hz, C*H*=CHCO), 5.96 (1H, d, *J* = 10.1 Hz, CH=C*H*CO), 4.74 (1H, br s, C*H*COPh), 4.49 (1H, d, *J* = 12.6 Hz, OCH_2C), 4.22 (1H, d, *J* = 12.6 Hz, OCH_2C), 2.87 (1H, dd, *J* = 5.2, 1.7 Hz, CH=CHCOC*H*CH), 2.64 (1H, br s, CC*H*-Ph), 2.51 (1H, d, *J* = 11.8 Hz, CH=CHCOC*H*CH), 1.57–1.76 (7H, m, $(CH_2)_2CH_3$, C=CCH_3), 1.23–1.31 (1H, m, CH_2CH$_3$), 1.07–1.15 (1H, m, CH_2CH$_3$), 0.89 (3H, t, *J* = 7.6 Hz, CH$_2$CH_3), 0.84 (3H, t, *J* = 7.6 Hz, CH$_2$CH_3). **^{13}C{^{1}H} NMR** (100 MHz, $CDCl_3$) δ 202.3, 196.3, 151.2, 135.4, 133.2, 131.5, 128.9, 128.8, 128.5, 127.9, 82.5, 68.6, 44.4, 42.9, 41.5, 38.2, 34.6, 31.0, 21.3, 17.6, 14.1, 8.0. **HRMS** (EI): *m/z* Calcd for $C_{24}H_{28}O_3$: (M^+) 364.2038, found 364.2039. $[\alpha]_D^{20}$ = (+) 52.7 (*c* = 0.14, in $CHCl_3$). **Chiral separation** (96% ee): The enantioselectivity was determined by SFC using Chiralpak IA (Back pressure = 15 MPa, Flow (CO_2) = 3.0 mL/min, Flow (isopropanol) = 0.3 mL/min, 25 °C, λ = 250 nm). Retention time: t_R = 2.4 min (minor enantiomer) and 4.2 min (major enantiomer).

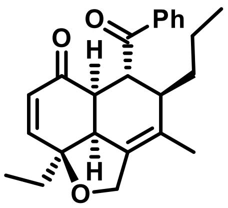

(2a^1*S*,4*S*,5*R*,5a*R*,8a*S*)-5-Benzoyl-8a-(2-methoxyethyl)-4-(4-methoxyphenyl)-3-methyl-2,2a^1,4,5,5a,8a-hexahydro-6*H*-naphtho[1,8-*bc*]furan-6-one (3ca): Following the general procedure, (*R*,*R*)-**L*6**·HBF_4 (16.0 mg, 0.02 mmol), NaO*t*Bu (2.0 mg, 0.02 mmol), Ni(cod)$_2$ (5.5 mg, 0.02 mmol), 4-(but-2-yn-1-yloxy)-4-(2-methoxyethyl)cyclohexa-2,5-dien-1-one (**1c**, 48.4 mg, 0.24 mmol) and (*E*)-3-(4-methoxyphenyl)-1-phenylprop-2-en-1-one (**2a**, 47.0 mg, 0.197 mmol) were used. Purification by silica gel flash column chromatography (20% EtOAc in hexane) gave **3ca** (55.0 mg, 0.12 mmol, 60% yield) as a white amorphous solid. R_f = 0.2 (30% EtOAc in hexane). **^{1}H NMR** (400 MHz, $CDCl_3$): δ 8.00 (2H, d, *J* = 7.9 Hz, benzoyl-*ortho*-Ar-*H*), 7.52 (1H, dd, *J* = 7.9, 7.9 Hz, benzoyl-*para*-Ar-*H*), 7.44 (2H, dd, *J* = 7.9, 7.9 Hz, benzoyl-*meta*-Ar-*H*), 6.90 (2H, d, *J* = 8.8 Hz, anisyl-*ortho*-Ar-*H*), 6.79 (2H, d, *J* = 8.8 Hz, anisyl-*meta*-Ar-*H*), 6.52 (1H, dd, *J* = 10.3, 2.0 Hz, C*H*=CHCO), 6.04 (1H, d, *J* = 10.3 Hz,

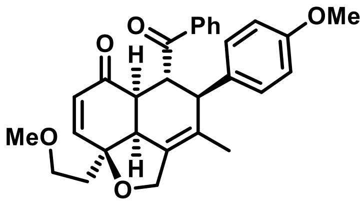

CH=C*H*CO), 4.85 (1H, dd, *J* = 2.2, 1.7 Hz, C*H*COPh), 4.54 (1H, d, *J* = 13.3 Hz, OCH_2C), 4.33 (1H, d, *J* = 13.3 Hz, OCH_2C), 3.88 (1H, br s, CH=CHCOC*H*CH), 3.78 (3H, s, OCH_3), 3.33–3.40 (2H, m, CH_2OCH_3), 3.19 (1H, dd, *J* = 5.6, 1.3 Hz, CC*H*-Ph), 2.86 (3H, s, $CH_2OC$$H_3$), 2.80 (1H, br s, C=CC*H*), 2.03–2.10 (1H, m, C$H_2$$CH_2OCH_3$), 1.91–1.97 (1H, m, C$H_2$$CH_2OCH_3$), 1.41 (3H, br s, C=CC$H_3$). **^{13}C{^{1}H} NMR** (100 MHz, $CDCl_3$) δ 201.3, 195.9, 158.1, 150.3, 135.8, 135.2, 133.8, 133.2, 130.1, 129.3, 128.8, 128.7, 126.5, 113.6, 82.1, 68.2, 67.8, 58.0, 55.1, 49.9, 43.8, 43.1, 42.5, 38.1, 18.1. **HRMS** (EI): *m/z* Calcd for $C_{29}H_{30}O_5$: (M^+) 458.2093, found 458.2088. $[\alpha]_D^{23}$ = (+) 215.9 (*c* = 0.10, in $CHCl_3$). **Chiral separation** (98% ee): The enantioselectivity was determined by SFC using Chiralpak IA (Back pressure = 15 MPa, Flow (CO_2) = 2.4 mL/min, Flow (isopropanol) = 0.6 mL/min, 25 °C, λ = 250 nm). Retention time: t_R = 3.1 min (minor enantiomer) and 4.5 min (major enantiomer).

(2a^1*S*,4*S*,5*R*,5a*R*,8a*S*)-5-Benzoyl-4-(4-chlorophenyl)-8a-(2-methoxyethyl)-3-methyl-2,2a^1,4,5,5a,8a-hexahydro-6*H*-naphtho[1,8-*bc*]furan-6-one (3ck): Following the general procedure, (*R*,*R*)-**L*6**·HBF_4 (16.0 mg, 0.02 mmol), NaO^{t}Bu (2.0 mg, 0.02 mmol), Ni(cod)$_2$ (5.5 mg, 0.02 mmol), 4-(but-2-yn-1-yloxy)-4-(2-methoxyethyl)cyclohexa-2,5-dien-1-one (**1c**, 48.0 mg, 0.24 mmol) and (*E*)-3-(4-chlorophenyl)-1-phenylprop-2-en-1-one (**2k**, 49.0 mg, 0.2 mmol) were used. Purification by silica gel flash column chromatography (15% EtOAc in hexane) gave **3ck** (63.0 mg, 0.136 mmol, 68% yield) as a white amorpous solid. R_f = 0.2 (20% EtOAc in hexane). **^{1}H NMR** (400 MHz, $CDCl_3$): δ 7.97 (2H, d, *J* = 7.6 Hz, benzoyl-*ortho*-Ar-*H*), 7.54 (1H, dd, *J* = 7.6, 7.6 Hz, benzoyl-*para*-Ar-*H*), 7.43 (2H, dd, *J* = 7.6, 7.6 Hz, benzoyl-*meta*-Ar-*H*), 7.22 (2H, d, *J* = 8.4 Hz, anisyl-*ortho*-Ar-*H*), 6.92 (2H, d, *J* = 8.4 Hz, anisyl-*meta*-Ar-*H*), 6.52 (1H, dd, *J* = 10.2, 1.8 Hz, C*H*=CHCO), 6.03 (1H, d, *J* = 10.2 Hz, CH=C*H*CO), 4.81 (1H, dd, *J* = 2.0, 1.8 Hz, C*H*COPh), 4.55 (1H, d, *J* = 13.1 Hz, OCH_2C), 4.32 (1H, d, *J* = 13.1 Hz, OCH_2C), 3.93 (1H, br s, CH=CHCOC*H*CH), 3.31–3.93 (2H, m, CH_2OCH_3), 3.22 (1H, dd, *J* = 5.8, 1.5 Hz, CC*H*-Ph), 2.81 (3H, s, $CH_2OC$$H_3$), 2.78 (1H, br s, C=CC*H*), 2.02–2.09 (1H, m, C$H_2$$CH_2OCH_3$), 1.89–1.96 (1H, m, C$H_2$$CH_2OCH_3$), 1.40 (3H, br s, C=CC$H_3$). **^{13}C{^{1}H} NMR** (100 MHz, $CDCl_3$) δ 200.8, 195.8, 150.4, 142.3, 135.0, 134.6, 133.3, 132.3, 130.5, 129.2, 128.8, 128.7, 128.5, 125.6, 82.2, 68.2, 67.8, 58.0, 49.6, 43.8, 43.2, 42.5, 38.0, 18.1. **HRMS** (EI): *m/z* Calcd for $C_{28}H_{27}ClO_4$: (M^+) 462.1798, found 462.1594. $[\alpha]_D^{20}$ = (+) 132.8 (*c* = 0.27, in $CHCl_3$). **Chiral separation** (98% ee): The enantioselectivity was determined by SFC using Chiralpak IA (Back

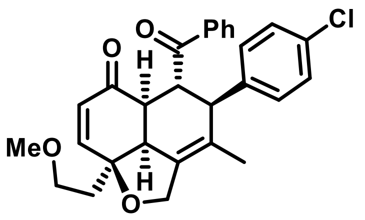

pressure = 15 MPa, Flow (CO_2) = 2.4 mL/min, Flow (isopropanol) = 0.6 mL/min, 40 °C, λ = 250 nm). Retention time: t_R = 3.0 min (minor enantiomer) and 3.8 min (major enantiomer).

(2a^1*S*,4*S*,5*R*,5a*R*,8a*S*)-5-Benzoyl-4,8a-dimethyl-3-phenyl-2,2a1,4,5,5a,8a-hexahydro-6*H*-naphtho[1,8-*bc*]furan-6-one (3de): Following the general procedure, (*R,R*)-**L*6**·HBF$_4$ (16.0 mg, 0.02 mmol), NaO^{t}Bu (2.0 mg, 0.02 mmol), Ni(cod)$_2$ (5.5 mg, 0.02 mmol), 4-methyl-4-((3-phenylprop-2-yn-1-yl)oxy)cyclohexa-2,5-dien-1-one (**1d**, 57.0 mg, 0.24 mmol) and (*E*)-1-phenylbut-2-en-1-one (**2e**, 29.8 mg, 0.02 mmol), were used. Purification by silica gel flash column chromatography gave **3de** (54.0 mg, mmol, 0.14 mmol, 70% yield) as thick oil. R_f = 0.3 (20% EtOAc in hexane). **^{1}H NMR** (400 MHz, $CDCl_3$): δ 8.08 (2H, d, *J* = 7.8 Hz, benzoyl-*ortho*-Ar-*H*), 7.61 (1H, dd, *J* = 7.8, 7.8 Hz, benzoyl-*para*-Ar-*H*), 7.52 (2H, dd, *J* = 7.8, 7.8 Hz, benzoyl-*meta*-Ar-*H*), 7.30 (2H, dd, *J* = 7.6, 7.6 Hz, Ph-*ortho*-Ar-*H*), 7.22 (1H, dd, *J* = 7.6, 7.6 Hz, Ph-*para*-Ar-*H*), 7.05 (2H, d, *J* = 7.6 Hz, Ph-*meta*-Ar-*H*), 6.59 (1H, dd, *J* = 10.1, 2.0 Hz, C*H*=CHCO), 6.03 (1H, d, *J* = 10.2 Hz, CH=C*H*CO), 4.73 (1H, br s, C*H*COPh), 4.26 (1H, d, *J* = 13.5 Hz, OC*H*$_2$C), 4.19 (1H, d, *J* = 13.5 Hz, OC*H*$_2$C), 2.93 (2H, dd, *J* = 1.6, 5.6 Hz, CH=CHCOC*H*CH, CHC*H*$_3$), 2.85 (1H, br s, CC*H*C=C), 1.46 (3H, s, CC*H*$_3$), 0.92 (3H, d, *J* = 7.6 Hz, CHC*H*$_3$). **^{13}C{^{1}H} NMR** (100 MHz, $CDCl_3$) δ 202.0, 196.0, 151.9, 140.1, 135.5, 134.8, 134.2, 133.3, 128.9, 128.5, 128.33, 128.25, 127.6, 126.8, 80.0, 68.5, 47.8, 44.1, 42.1, 33.2, 23.9, 21.3. **HRMS** (EI+): *m/z* Calcd for $C_{26}H_{24}O_3$: (M^+) 384.1725, found 384.1727. $[\alpha]_D^{23}$ = (+) 211.4 (*c* = 0.11, in $CHCl_3$). **Chiral separation** (99% ee): The enantioselectivity was determined by SFC using Chiralpak IA (Back pressure = 15 MPa, Flow (CO_2) = 3.0 mL/min, Flow (isopropanol) = 0.3 mL/min, 25 °C, λ = 250 nm). Retention time: t_R = 3.4 min (minor enantiomer) and 5.7 min (major enantiomer).

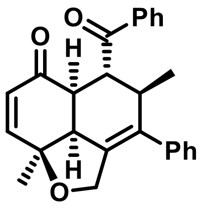

(2a^1*S*,4*S*,5*R*,5a*R*,8a*S*)-5-Benzoyl-4-(4-fluorophenyl)-3-(4-methoxyphenyl)-8a-methyl-2,2a^1,4,5,5a,8a-hexahydro-6*H*-naphtho[1,8-*bc*]furan-6-one (3ec): Following the general procedure, (*R,R*)-**L*6**·HBF$_4$ (16.0 mg, 0.02 mmol), NaO^{t}Bu (2.0 mg, 0.02 mmol), Ni(cod)$_2$ (5.5 mg, 0.02 mmol), 4-((3-(4-methoxyphenyl)prop-2-yn-1-yl)oxy)-4- methylcyclohexa-2,5-dien-1-one (**1e**, 64.3 mg, 0.24 mmol) and (*E*)-3-(4-fluorophenyl)-1-phenylprop-2-en-1-one (**2c**, 45.0 mg, 0.2 mmol) were used. Purification by silica gel flash column chromatography (20% EtOAc in hexane) gave **3ec** (70.0 mg, 0.14 mmol, 71% yield) as a white amorphous solid. R_f = 0.2 (20% EtOAc in

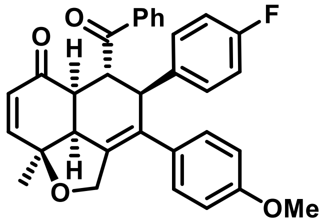

hexane). **^{1}H NMR** (400 MHz, $CDCl_3$) δ 7.99 (2H, d, *J* = 7.6 Hz, benzoyl-*ortho*-Ar-*H*), 7.56 (1H, dd, *J* = 7.6, 7.6 Hz, benzoyl-*para*-Ar-*H*), 7.44 (2H, dd, *J* = 7.6, 8.6 Hz, benzoyl-*ortho*-Ar-*H*), 6.88 (2H, dd, *J* = 8.6, 8.6 Hz, Ar-*H*), 6.83 (2H, d, *J* = 8.6 Hz, Ar-*H*), 6.76 (2H, dd, *J* = 8.6, 8.6 Hz, Ar-*H*), 6.63 (1H, dd, *J* = 1.8, 10.1 Hz, C*H*=CHCO), 6.62 (2H, d, *J* = 8.6 Hz, Ar-*H*), 6.09 (1H, d, *J* = 10.1 Hz, CH=C*H*CO), 4.99 (1H, dd, *J* =1.4, 3.5 Hz, C*H*COPh), 4.58 (1H, d, *J* = 13.2 Hz, OCH_2C), 4.50 (1H, quintet, *J* = 3.3 Hz, C*H*Ar), 4.34 (1H, dq, *J* = 1.5, 13.2 Hz, OCH_2C), 3.69 (3H, s, OCH_3), 3.04 (1H, dd, *J* = 1.4, 5.7 Hz, CH=CHCOC*H*CH), 2.91 (1H, br s, CC*H*C=CH), 1.50 (3H, s, CCH_3). **^{13}C{^{1}H} NMR** (100 MHz, $CDCl_3$) δ 201.2, 195.4, 161.2 (d, J_{CF} = 250.4 Hz), 158.0, 151.4, 139.1 (d, J_{CF} = 3.1 Hz), 137.2, 135.3, 133.5, 131.7 (d, J_{CF} = 15.7 Hz), 130.9 (d, J_{CF} = 7.4 Hz), 129.0, 128.9, 128.8, 128.6, 115.0, 114.7, 113.3, 80.1, 68.8, 55.0, 50.2, 45.3, 43.9, 43.8, 23.8. **HRMS** (EI+): *m/z* Calcd for $C_{32}H_{27}FO_4$: (M^+) 494.1893, found 494.1895. $[\alpha]_D^{20}$ = (+) 217.2 (*c* = 0.30, in $CHCl_3$). **Chiral separation** (98% ee): The enantioselectivity was determined by SFC using Chiralpak IA (Back pressure = 15 MPa, Flow (CO_2) = 2.4 mL/min, Flow (isopropanol) = 0.6 mL/min, 40 °C, λ = 250 nm). Retention time: t_R = 3.0 min (minor enantiomer) and 4.3 min (major enantiomer).

(2a^1*S*,4*S*,5*R*,5a*R*,8a*S*)-5-Benzoyl-4-(4-chlorophenyl)-3-(4-methoxyphenyl)-8a-methyl-2,2a^1,4,5,5a,8a-hexahydro-6*H*-naphtho[1,8-*bc*]furan-6-one (3ek): Following the general procedure,

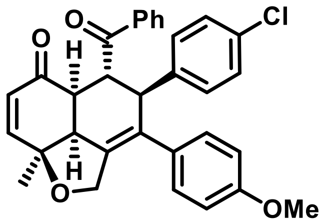

(*R*,*R*)-**L*6**·HBF_4 (32.2 mg, 0.04 mmol), NaO^{t}Bu (3.9 mg, 0.04 mmol), Ni(cod)$_2$ (10.9 mg, 0.04 mmol), 4-((3-(4-methoxyphenyl)prop-2-yn-1-yl)oxy)-4-methylcyclohexa-2,5-dien-1-one (**1e**, 128.0 mg, 0.48 mmol) and (*E*)-3-(4-chlorophenyl)-1-phenylprop-2-en-1-one (**2k**, 98.0 mg, 0.4 mmol) were used. Purification by silica gel flash column chromatography (20% EtOAc in hexane) gave **3ek** (138.0 mg, 0.27 mmol, 68% yield) as a white amorphous solid. R_f = 0.2 (20% EtOAc in hexane). **^{1}H NMR** (400 MHz, $CDCl_3$) δ 7.99 (2H, d, *J* = 8.0 Hz, benzoyl-*ortho*-Ar-*H*), 7.56 (1H, dd, *J* = 8.0, 8.0 Hz, benzoyl-*para*-Ar-*H*), 7.44 (2H, dd, *J* = 8.0, 8.0 Hz, Ar-*H*), 7.04 (2H, d, *J* = 8.3 Hz, Ar-*H*), 6.86 (2H, d, *J* = 8.3 Hz, Ar-*H*), 6.84 (2H, d, *J* = 8.3 Hz, Ar-*H*), 6.63 (1H, d, *J* = 10.3 Hz, C*H*=CHCO), 6.62 (2H, d, *J* = 8.3 Hz, Ar-*H*), 6.10 (1H, d, *J* = 10.3 Hz, CH=C*H*CO), 4.97 (1H, dd, *J* =1.1, 3.3 Hz, C*H*COPh), 4.48 (1H, dt, *J* = 2.6, 14.0 Hz, OCH_2C), 4.42 (1H, quintet, *J* = 3.1 Hz, C*H*Ar), 4.33 (1H, d, *J* = 14.0 Hz, OCH_2C), 3.69 (3H, s, OCH_3), 3.04 (1H, d, *J* = 5.9 Hz, CH=CHCOC*H*CH), 2.88 (1H, br s, CC*H*C=CH), 1.49 (3H, s, CCH_3). **^{13}C{^{1}H} NMR** (100 MHz, $CDCl_3$) δ 201.0, 195.2, 158.0, 151.4, 142.0, 137.3, 135.1, 133.5, 131.8, 131.5, 131.4, 130.7, 129.0, 128.9, 128.8, 128.6, 128.2, 113.3, 80.0, 68.7, 55.0, 50.0, 45.2, 43.9, 43.7, 23.7. **HRMS** (EI+): *m/z*

Calcd for $C_{32}H_{27}ClO_4$: (M^+) 510.1598, found 510.1591. $[\alpha]_D^{23}$ = (+) 201.8 (*c* = 0.46, in $CHCl_3$). **Chiral separation** (99% ee): The enantioselectivity was determined by SFC using Chiralpak IA (Back pressure = 15 MPa, Flow (CO_2) = 2.4 mL/min, Flow (isopropanol) = 0.6 mL/min, 40 °C, λ = 250 nm). Retention time: t_R = 3.9 min (minor enantiomer) and 5.8 min (major enantiomer).

Ethyl 4-((2a^1*S*,4*S*,5*R*,5a*R*,8a*S*)-5-benzoyl-4-(4-fluorophenyl)-8a-methyl-6-oxo-2a^1,4,5,5a,6,8a-hexahydro-2*H*-naphtho[1,8-*bc*]furan-3-yl)benzoate (3fc): Following the general procedure,

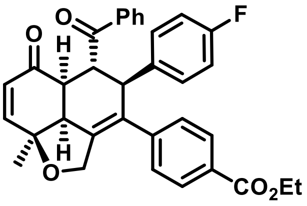

(*R*,*R*)-**L*6**·HBF_4 (8.2 mg, 0.01 mmol), NaO^{t}Bu (0.92 mg, 0.01 mmol), Ni(cod)$_2$ (2.7 mg, 0.01 mmol), ethyl 4-(3-((1-methyl-4-oxocyclohexa-2,5-dien-1-yl)oxy) prop-1-yn-1-yl)-benzoate (**1f**, 37.0 mg, 0.12 mmol) and (*E*)-3-(4-fluorophenyl)-1-phenylprop-2-en-1-one (**2c**, 22.6 mg, 0.1 mmol) were used. Purification by silica gel flash column chromatography (15% EtOAc in hexane) gave **3fc** (32.0 mg, 0.06 mmol, 60% yield) as thick oil. R_f = 0.3 (20% EtOAc in hexane). **^{1}H NMR** (400 MHz, $CDCl_3$) δ 7.99 (2H, d, *J* = 8.5 Hz, benzoyl-*ortho*-Ar-*H*), 7.77 (2H, d, *J* = 8.5 Hz, benzoyl-*meta*-Ar-*H*), 7.57 (1H, dd, *J* = 8.5, 8.5 Hz, benzoyl-*para*-Ar-*H*), 7.45 (2H, dd, *J* = 7.8, 7.8 Hz, Ar-*H*), 6.99 (2H, d, *J* = 8.3 Hz, Ar-*H*), 6.88 (2H, d, *J* = 8.7 Hz, Ar-*H*), 6.74 (2H, dd, *J* = 8.7, 8.7 Hz, Ar-*H*), 6.64 (1H, dd, *J* = 1.9, 10.2 Hz, CH=C*H*CO), 6.14 (1H, d, *J* = 10.2 Hz, CH=C*H*CO), 5.02 (1H, dd, *J* =1.4, 3.5 Hz, C*H*COPh), 4.25–4.49 (2H, m, OC*H*$_2$C), 4.25–4.44 (3H, m, C*H*Ar, OC*H*$_2$CH$_3$), 3.06 (1H, dd, *J* = 1.2, 5.9 Hz, CH=CHCOC*H*CH), 2.90 (1H, br s, CC*H*C=3CH), 1.51 (3H, s, CC*H*$_3$), 1.25 (3H, t, *J* =7.3 Hz, CH$_2$C*H*$_3$),. **^{13}C{^{1}H} NMR** (100 MHz, $CDCl_3$) δ 201.0, 195.1, 166.2 161.3 (d, J_{CF} = 237.6 Hz), 151.2, 144.1, 138.9, 138.7 (d, J_{CF} = 3.5 Hz), 135.1, 133.6, 130.8 (d, J_{CF} = 8.5 Hz), 129.2, 128.9, 128.9, 128.8, 128.7, 127.9, 115.2, 114.9, 80.2, 68.5, 60.9, 49.8, 45.4, 43.9, 43.5, 23.7, 14.3. **HRMS** (EI+): *m/z* Calcd for $C_{34}H_{29}FO_5$: (M^+) 536.1999, found 536.1992. $[\alpha]_D^{20}$ = (+) 158.4 (*c* = 0.09, in $CHCl_3$). **Chiral separation** (98% ee): The enantioselectivity was determined by SFC using Chiralpak IA (Back pressure = 15 MPa, Flow (CO_2) = 2.4 mL/min, Flow (isopropanol) = 0.6 mL/min, 40 °C, λ = 250 nm). Retention time: t_R = 3.0 min (minor enantiomer) and 4.6 min (major enantiomer).

(2a^1*S*,4*S*,5*R*,5a*R*,8a*S*)-5-Benzoyl-4,8a-dimethyl-3-(4-(trifluoromethyl)phenyl)-2,2a^1,4,5,5a,8a-hexahydro-6*H*-naphtho[1,8-*bc*]furan-6-one (3ge): Following the general procedure, (*R*,*R*)-**L*6**·HBF_4 (16.0 mg, 0.02 mmol), NaO^{t}Bu (2.0 mg, 0.02 mmol), Ni(cod)$_2$ (5.5 mg, 0.02 mmol), 4-methyl-4-((3-(4-(trifluoromethyl)phenyl)prop-2-yn-1-yl)oxy)cyclohexa-2,5-dien-1-one (**1g**, 61.5

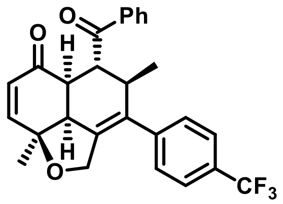

mg, 0.24 mmol) and (*E*)-1-phenylbut-2-en-1-one (**2e**, 29.2 mg, 0.2 mmol) were used. Purification by silica gel flash column chromatography (10% EtOAc in hexane) gave **3ge** (64.2 mg, 0.14 mmol, 71% yield) as a white crystalline solid. R_f = 0.3 (20% EtOAc in hexane). **^{1}H NMR** (400 MHz, $CDCl_3$) δ 8.08 (2H, d, *J* = 7.4 Hz, benzoyl-*ortho*-Ar-*H*), 7.62 (1H, dd, *J* = 7.4, 7.4 Hz, benzoyl-*para*-Ar-*H*), 7.51–7.57 (4H, m, benzoyl and CF_3-*meta*-Ar-*H*,), 7.19 (2H, d, *J* = 7.9 Hz, Ar-*H*), 6.59 (1H, dd, *J* = 1.8, 10.2 Hz, CH=C*H*CO), 6.03 (1H, d, *J* = 10.2 Hz, CH=C*H*CO), 4.74 (1H, br s, C*H*COPh), 4.18–4.24 (2H, m, OC*H*$_2$C), 3.03–3.07 (2H, m, CH=CHCOC*H*CH, CC*H*C=C), 2.82 (1H, br s, C*H*CH_3), 1.45 (3H, s, CHC*H*$_3$), 0.91 (3H, d, *J* =7.3 Hz, CHC*H*$_3$),. **^{13}C{^{1}H} NMR** (100 MHz, $CDCl_3$) δ 201.7, 195.6, 151.7, 143.8, 135.4, 135.3, 133.7, 133.4, 129.0, 128.5, 128.3, 128.0, 125.4, 125.3, 80.0, 68.2, 47.6, 44.2, 42.0, 32.9, 23.8, 21.1, one carbon overlapped in aromatic region. **HRMS** (EI+): *m/z* Calcd for $C_{27}H_{23}F_3O_3$: (M^+) 452.1599, found 452.1595. $[\alpha]_D^{23}$ = (+) 166.3 (*c* = 0.20, in $CHCl_3$). **Chiral separation** (96% ee): The enantioselectivity was determined by SFC using Chiralpak IA (Back pressure = 15 MPa, Flow (CO_2) = 3.0 mL/min, Flow (isopropanol) = 0.3 mL/min, 40 °C, λ = 250 nm). Retention time: t_R = 2.4 min (minor enantiomer) and 3.5 min (major enantiomer)

(2a^1*S*,4*S*,5*R*,5a*R*,8a*S*)-5-Benzoyl-3-ethyl-4,8a-dimethyl-2,2a1,4,5,5a,8a-hexahydro-6*H*-naphtho[1,8-*bc*]furan-6-one (3he): Following the general procedure, (*R*,*R*)-**L*6**·HBF_4 (16.0 mg, 0.02 mmol), NaO^{t}Bu (2.0 mg, 0.02 mmol), Ni(cod)$_2$ (5.5 mg, 0.02 mmol), 4-methyl-4-(pent-2-yn-1-yloxy)cyclohexa-2,5-dien-1-one (**1h**, 45.2 mg, 0.24 mmol) and (*E*)-1-phenylbut-2-en-1-one (**2e**, 29.0 mg, 0.2 mmol) were used. Purification by silica gel flash column chromatography (5% EtOAc in hexane) gave **3he** (46.4 mg, 0.138 mmol, 69% yield) as thick oil. R_f = 0.4 (20% EtOAc in hexane). **^{1}H NMR** (400 MHz, $CDCl_3$) δ 8.03 (2H, d, *J* = 7.3 Hz, benzoyl-*ortho*-Ar-*H*), 7.58 (1H, dd, *J* = 7.3, 7.3 Hz, benzoyl-*para*-Ar-*H*), 7.50 (2H, dd, *J* = 7.3, 7.3 Hz, benzoyl-*meta*-Ar-*H*), 6.51 (1H, dd, *J* = 2.0, 10.2 Hz, CH=C*H*CO), 5.90 (1H, d, *J* = 10.2 Hz, CH=C*H*CO), 4.58 (1H, br s, C*H*COPh), 4.51 (1H, d, *J* = 12.5 Hz, OC*H*$_2$C), 4.26 (1H, d, *J* = 12.5 Hz, OC*H*$_2$C), 2.92 (1H, dd, *J* = 1.7, 5.7 Hz, CH=CHCOC*H*CH), 2.73–2.77 (1H, m, CC*H*C=C), 2.70 (1H, br s, C*H*CH_3), 2.03 (1H, dq, *J* = 7.5, 15.5 Hz, C*H*$_2$$CH_3$), 1.90 (1H, dq, *J* = 7.5, 15.5 Hz, C*H*$_2$$CH_3$), 1.40 (3H, s, CC*H*$_3$), 1.10 (3H, d, *J* =7.5 Hz,, CHC*H*$_3$), 0.96 (3H, t, *J* =7.5 Hz, CH_2C*H*$_3$). **^{13}C{^{1}H} NMR** (100 MHz, $CDCl_3$) δ 202.0, 196.3, 152.0, 135.6, 134.3, 133.2, 130.7, 128.9, 128.5, 128.0, 79.7, 68.2, 48.2, 43.9, 42.2, 30.6, 24.1, 24.0, 20.5, 12.3. **HRMS** (EI+): *m/z* Calcd for $C_{22}H_{24}O_3$: (M^+) 336.1725, found 336.1723. $[\alpha]_D^{20}$ = (+)

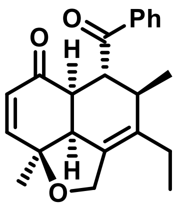

113.7 (c = 0.28, in $CHCl_3$). **Chiral separation** (98% ee): The enantioselectivity was determined by SFC using Chiralpak IA (Back pressure = 15 MPa, Flow (CO_2) = 3.0 mL/min, Flow (isopropanol) = 0.3 mL/min, 25 °C, λ = 250 nm). Retention time: t_R = 2.4 min (minor enantiomer) and 3.4 min (major enantiomer)

(2a^1*S*,4*S*,5*R*,5a*R*,8a*S*)-5-Benzoyl-4-(4-fluorophenyl)-8a-methyl-3-(((triethylsilyl)oxy)methyl)-2,2a1,4,5,5a,8a-hexahydro-6*H*-naphtho[1,8-*bc*]furan-6-one (3ic): Following the general procedure, (*R*,*R*)-**L*6**·HBF_4 (8.2 mg, 0.01 mmol), NaO^{t}Bu (1.0 mg, 0.02 mmol), Ni(cod)$_2$ (2.75 mg, 0.01 mmol), 4-methyl-4-((4-((triethylsilyl)oxy)but-2-yn-1-yl)oxy)cyclohexa-2,5-dien-1-one (**1i**, 33.6 mg, 0.12 mmol) and (*E*)-3-(4-fluorophenyl)-1-phenylprop-2-en-1-one (**2c**, 22.6 mg, 0.1 mmol) were used. Purification by silica gel flash column chromatography (5% EtOAc in hexane) gave **3ic** (39.0 mg, 0.07 mmol, 74% yield) as a white amorphous solid. R_f = 0.4 (20% EtOAc in hexane). **^{1}H NMR** (400 MHz, $CDCl_3$) δ 7.95 (2H, d, J = 7.5 Hz, benzoyl-*ortho*-Ar-*H*), 7.55 (1H, dd, J = 7.5,7.5 Hz, benzoyl-*para*-Ar-*H*), 7.43 (2H, dd, J = 7.5, 7.5 Hz, benzoyl-*para*-Ar-*H*), 6.90–6.99 (4H, m, Ar-*H*), 6.59 (1H, dd, J = 2.0, 10.2 Hz, CH=C*H*CO), 5.99 (1H, d, J = 10.2 Hz, CH=C*H*CO), 4.85 (1H, dd, J =1.5, 3.0 Hz, C*H*COPh), 4.77 (1H, dq, J = 1.5, 13.0 Hz, OCH_2C), 4.43 (1H, d, J = 13.0 Hz, OCH_2C), 4.00 (1H, t, J =2.6 Hz, C*H*Ar), 3.84 (1H, d, J = 13.2 Hz, CH_2OTES), 3.77 (1H, d, J = 13.2 Hz, CH_2OTES), 2.97 (1H, dd, J = 1.5, 5.7 Hz, CH=CHCOC*H*CH), 2.81 (1H, br s, CC*H*C=C), 1.46 (3H, s, CCH_3), 0.82 (9H, t, J =8.0 Hz, CH_2CH_3), 0.44 (6H, t, J =8.0 Hz, C$H_2$$CH_3$). **^{13}C{^{1}H} NMR** (100 MHz, $CDCl_3$) δ 200.9, 195.4, 162.6 (d, J_{CF} = 237.6 Hz), 151.9, 138.7 (d, J_{CF} = 3.1 Hz), 135.4 (d, J_{CF} = 32.2 Hz), 133.4, 130.5 (d, J_{CF} = 7.3 Hz), 129.4, 129.1, 128.8, 128.3, 123.8, 115.1 (d, J_{CF} = 21.8 Hz), 79.1, 68.6, 62.8, 49.4, 44.9, 43.3, 41.4, 23.8, 6.6, 4.1. **HRMS** (EI+): *m/z* Calcd for $C_{32}H_{37}FO_4Si$: (M^+) 532.2445, found 532.2441. $[\alpha]_D^{20}$ = (+) 103.6 (c = 0.19, in $CHCl_3$). **Chiral separation** (95% ee): The enantioselectivity was determined by SFC using Chiralpak IA (Back pressure = 15 MPa, Flow (CO_2) = 3.0 mL/min, Flow (isopropanol) = 0.3 mL/min, 25 °C, λ = 250 nm). Retention time: t_R = 2.5 min (minor enantiomer) and 3.4 min (major enantiomer).

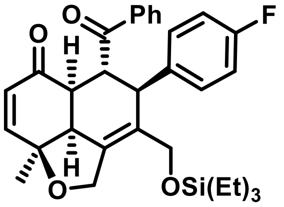

(2a^1*S*,4*S*,5*R*,5a*R*,8a*S*)-5-Benzoyl-4-(4-chlorophenyl)-8a-methyl-3-(((triethylsilyl)oxy)methyl)-2,2a1,4,5,5a,8a-hexahydro-6*H*-naphtho[1,8-*bc*]furan-6-one (3ik): Following the general procedure, (*R*,*R*)-**L*6**·HBF_4 (8.1 mg, 0.02 mmol), NaO^{t}Bu (1.0 mg, 0.01 mmol), Ni(cod)$_2$ (2.76 mg, 0.01 mmol), 4-methyl-4-((4-((triethylsilyl)oxy)but-2-yn-1-yl)oxy)cyclohexa-2,5-dien-1-one (**1i**, 33.6 mg, 0.12

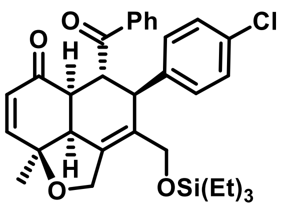

mmol) and (*E*)-3-(4-chlorophenyl)-1-phenylprop-2-en-1-one (**2k**, 24.0 mg, 0.1 mmol) were used. Purification by silica gel flash column chromatography (10% EtOAc in hexane) gave **3ik** (42.0 mg, 0.08 mmol, 77% yield) as a white amorphous solid. R_f = 0.4 (20% EtOAc in hexane). **^{1}H NMR** (400 MHz, $CDCl_3$) δ 7.95 (2H, d, *J* = 7.6 Hz, benzoyl-*ortho*-Ar-*H*), 7.55 (1H, dd, *J* = 7.6,7.6 Hz, benzoyl-*para*-Ar-*H*), 7.43 (2H, dd, *J* = 7.6, 7.6 Hz, benzoyl-*para*-Ar-*H*), 7.20 (2H, *J* = 8.3 Hz, Ar-*H*), 6.94 (2H, *J* = 8.3 Hz, Ar-*H*), 6.59 (1H, dd, *J* = 1.8, 10.2 Hz, CH=C*H*CO), 5.99 (1H, d, *J* = 10.2 Hz, CH=C*H*CO), 4.83 (1H, dd, *J* =1.5, 3.1 Hz, C*H*COPh), 4.77 (1H, dq, *J* = 1.8, 13.9 Hz, OCH_2C), 4.43 (1H, d, *J* = 13.9 Hz, OCH_2C), 4.00 (1H, t, *J* = 2.7 Hz, C*H*Ar), 3.85 (1H, d, *J* = 13.2 Hz, CH_2OTES), 3.77 (1H, d, *J* = 13.2 Hz, CH_2OTES), 2.97 (1H, dd, *J* = 1.3, 5.9 Hz, CH=CHCOC*H*CH), 2.80 (1H, br s, CC*H*C=C), 1.46 (3H, s, CCH_3), 0.83 (9H, t, *J* =8.0 Hz, CH_2CH_3), 0.44 (6H, t, *J* =8.0 Hz, CH_2CH$_3$). **^{13}C{^{1}H} NMR** (100 MHz, $CDCl_3$) δ 200.8, 195.3, 152.0, 141.6, 135.8, 135.2, 133.4, 132.4, 130.4, 128.9, 128.8, 128.4, 128.3, 79.1, 68.6, 62.8, 49.3, 44.8, 43.3, 41.5, 23.8, 6.6, 4.1, one carbon overlapped in aromatic region. **HRMS** (EI+): *m/z* Calcd for $C_{32}H_{37}ClO_4Si$: (M^+) 548.2140, found 548.2140. $[\alpha]_D^{23}$ = (+) 149.3 (*c* = 0.15, in $CHCl_3$). **Chiral separation** (97% ee, 100% ee after single recrystallization): The enantioselectivity was determined by SFC using Chiralpak IA (Back pressure = 15 MPa, Flow (CO_2) = 3.0 mL/min, Flow (isopropanol) = 0.3 mL/min, 25 °C, λ = 250 nm). Retention time: t_R = 3.1 min (minor enantiomer) and 4.5 min (major enantiomer).

Synthetic Applications of 3aa

(2a^1*S*,4*S*,5a*R*,8a*S*)-5-Benzoyl-4-(4-methoxyphenyl)-3,8a-dimethyl-2a^1,5,5a,8a-tetrahydro-2*H*-naphtho[1,8-*bc*]furan-2,6(4H)-dione (4): To a solution of **3aa** (41 mg, 0.10 mmol) in CH_2Cl_2 (1 mL)

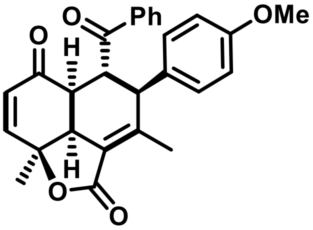

in a flask under N_2 at room temperature was added pyridine (10μL) and PCC (22.0 mg, 0.1 mmol). The resulting solution was heated with stirring at 50 °C for 3 h. The reaction mixture was cooled to room temperature and another portion of pyridine (10μL) and PCC (22.0 mg, 0.1 mmol) were added and again heated for another 3 h. The resulting brown solution was stirred at room temperature for 24 h to complete the reaction. The solid was filtered off and washed with CH_2Cl_2, concentrated under vacuo and purified by silica gel flash chromatography (25% ethyl acetate in hexanes) to get pure lactone **4** (39.0 mg, 0.09 mmol, 90%) as thick oil. R_f = 0.2 (30% EtOAc in hexane). **^{1}H NMR** (400 MHz, $CDCl_3$) δ 7.99 (2H, d, *J* = 7.3 Hz,

benzoyl-*ortho*-Ar-*H*), 7.59 (1H, dd, *J* = 7.3, 7.3 Hz, benzoyl-*para*-Ar-*H*), 7.47 (2H, dd, *J* = 7.3, 7.3 Hz, benzoyl-*meta*-Ar-*H*), 6.87 (2H, *J* = 8.4 Hz, Ar-*H*), 6.80 (2H, *J* = 8.4 Hz, Ar-*H*), 6.67 (1H, dd, *J* = 1.6, 10.0 Hz, CH=C*H*CO), 6.06 (1H, d, *J* = 10.0 Hz, CH=C*H*CO), 4.94 (1H, br s, C*H*COPh), 3.94 (1H, s, C*H*Ar), 3.78 (3H, s, OCH_3), 3.22 (1H, br s, CH=CHCOC*H*CH), 3.01 (1H, d, *J* = 6.6 Hz, CC*H*C=C), 2.04 (3H, d, *J* = 2.2 Hz, $C{=}CCH_3$), 1.63 (3H, br s, CCH_3). **$^{13}C\{^1H\}$ NMR** (100 MHz, $CDCl_3$) δ 200.5, 193.6, 167.2, 158.6, 153.4, 147.4, 134.6, 133.8, 133.5, 130.1, 129.0, 128.9, 127.9, 120.9, 114.0, 78.7, 55.2, 48.8, 46.3, 43.0, 41.6, 24.7, 17.4. **HRMS** (EI+): *m/z* Calcd for $C_{27}H_{24}O_5$: (M^+) 428.1624, found 428.1622. $[\alpha]_D^{23}$ = (+) 205.3 (*c* = 0.11, in $CHCl_3$). **Chiral separation** (98% ee): The enantioselectivity was determined by SFC using Chiralpak IA (Back pressure = 15 MPa, Flow (CO_2) = 2.4 mL/min, Flow (isopropanol) = 0.6 mL/min, 40 ºC, λ = 250 nm). Retention time: t_R = 4.3 min (minor enantiomer) and 5.3 min (major enantiomer).

(2a^1*S*,4*S*,5a*R*,6a*S*,7a*R*,7b*R*)-5-Benzoyl-4-(4-methoxyphenyl)-3,7b-dimethyl-2,2a^1,4,5,5a,6a,7a,7b-octahydro-6*H*-oxireno[2',3':2,3]naphtho[1,8-*bc*]furan-6-one (5): To a solution of **3aa** (41.0 mg, 0.10 mmol) in MeOH:CH_2Cl_2 (3:1) (0.4 mL), H_2O_2 (30 wt. % in H_2O) (0.2 mL) and NaOH (20 wt. % in H_2O) (0.1 mL) were added sequentially at 0 ºC. The reaction was allowed to stir at 0 ºC to rt for overnight. The reaction was quenched with sat. $Na_2S_2O_3$(aq.) (5 mL) to quench excess peroxides. The reaction was partitioned between CH_2Cl_2 (15 mL) and H_2O (10 mL). The layers were separated and the aqueous layer was extracted with CH_2Cl_2 (2 x 10 mL). The combined organic layers was washed with brine, dried over Na_2SO_4, filtered, and concentrated in vacuo. The crude residue was purified by silica gel flash column chromatography (10% EtOAc in hexane) to afford epoxide **5** (32.1 mg, 0.07 mmol, 75% yield) as a white solid. R_f = 0.4 (20% EtOAc in hexane). **^1H NMR** (400 MHz, $CDCl_3$) δ 7.74 (2H, d, *J* = 8.0 Hz, benzoyl-*ortho*-Ar-*H*), 7.51 (1H, dd, *J* = 7.4, 8.0 Hz, benzoyl-*para*-Ar-*H*), 7.36 (2H, dd, *J* = 7.4, 8.0 Hz, benzoyl-*meta*-Ar-*H*), 7.17 (2H, br s, Ar-*H*), 6.81 (2H, d *J* = 7.4 Hz, Ar-*H*), 4.74 (1H, dd, *J* =1.2, 6.3 Hz, C*H*COPh), 4.53 (1H, doublet, *J* = 13.3 Hz, OC*H*$_2$C), 4.44 (1H, *J* = 13.3 Hz, OC*H*$_2$C), 3.89 (1H, br s, C*H*Ar), 3.76 (3H, s, OCH_3), 3.40–3.43 (2H, m, C*H*OC*H*), 3.31 (1H, d *J* = 4.8 Hz, OCHCOC*H*CH), 2.75 (1H, br s, CC*H*C=C), 1.60 (3H, s, CCH_3), 1.31 (3H, br s, $C{=}CCH_3$). **$^{13}C\{^1H\}$ NMR** (100 MHz, $CDCl_3$) δ 204.1, 201.4, 158.3, 135.7, 134.7, 133.3, 132.8, 129.3, 128.7, 128.6, 113.7, 79.6, 69.0, 63.8, 55.7, 55.2, 49.6, 48.1, 44.4, 42.7, 23.9, 18.0, one carbon overlapped in aromatic region. **HRMS** (EI+): *m/z* Calcd for $C_{27}H_{26}O_5$: (M^+) 430.1780, found 430.1781. $[\alpha]_D^{23}$ = (+) 54.7 (*c* = 0.07, in $CHCl_3$). **Chiral separation** (98% ee): The enantioselectivity was determined

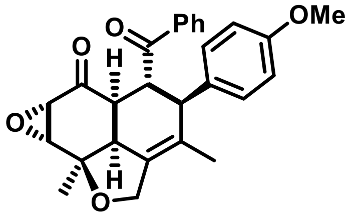

by SFC using Chiralpak IA (Back pressure = 15 MPa, Flow (CO_2) = 2.4 mL/min, Flow (isopropanol) = 0.6 mL/min, 40 °C, λ = 250 nm). Retention time: t_R = 2.6 min (minor enantiomer) and 2.9 min (major enantiomer).

Diethyl 2-((2a^1*S*,4*S*,5a*R*,8*S*,8a*S*)-5-benzoyl-4-(4-methoxyphenyl)-3,8a-dimethyl-6-oxo-2a^1,4,5,5a,6,7,8,8a-octahydro-2*H*-naphtho[1,8-*bc*]furan-8-yl)malonate (6): To a solution of **3aa** (22.0 mg, 0.053 mmol) in THF (2 mL) was added sodium diethyl malonate (11.7 mg, 0.063 mmol) at room temperature and stirred for 6 h. The reaction was diluted with ether the added water. The reaction was partitioned. The layers were separated and the aqueous layer was extracted with ether (2 x 10 mL). The combined organic extracts were dried over Na_2SO_4, filtered, and concentrated *in vacuo*. The crude residue was purified by silica gel flash column chromatography to afford epoxide **6** (26.0 mg, 0.045 mmol, 85% yield) as a white amorphous solid. **^{1}H NMR** (400 MHz, $CDCl_3$) δ 7.93 (2H, d, *J* = 7.6 Hz, benzoyl-*ortho*-Ar-*H*), 7.56 (1H, dd, *J* = 7.6, 7.6 Hz, benzoyl-*para*-Ar-*H*), 7.45 (2H, dd, *J* = 7.6, 7.6 Hz, benzoyl-*meta*-Ar-*H*), 6.94 (2H, d, *J* = 9.0 Hz, Ar-*H*), 6.84 (2H, d *J* = 9.0 Hz, Ar-*H*), 4.76 (1H, br s, C*H*COPh), 4.45 (2H, br s, OCH_2C), 4.16–4.28 (4H, CH_2CH$_3$), 3.79 (3H, s, OCH$_3$), 3.66 (1H, br s, C*H*Ar), 3.48 (1H, d, *J* = 9.5 Hz, C*H*(CO_2Et)$_2$), 2.98 (1H, ddd, *J* = 3.3, 9.5, 13.7 Hz, C*H*CH$_2$), 2.83 (1H, d, *J* = 8.1 Hz, CH$_2$COC*H*CH), 2.70–2.73 (1H, m, CC*H*C=C), 2.50 (1H, dd, *J* = 3.8, 16.1 Hz, CHCH_2), 2.15 (1H, dd, *J* = 14.7, 16.1 Hz, CHCH_2), 1.50 (3H, br s, C=CCH$_3$), 1.46 (3H, s, CCH_3), 1.31 (3H, t, *J* = 7.1, Hz, CH$_2$CH_3), 1.28 (3H, t, *J* = 7.1, Hz, C H$_2$CH_3). **^{13}C{^{1}H} NMR** (100 MHz, $CDCl_3$) δ 204.6, 201.5, 168.2, 168.1, 158.3, 135.2, 135.1, 133.3, 132.8, 129.8, 128.9, 128.7, 126.2, 113.7, 82.3, 67.0, 61.7, 61.6, 55.1, 52.3, 48.1, 47.7, 44.4, 44.2, 41.0, 37.6, 18.6, 18.2, 14.0, 13.9. **HRMS** (EI+): *m/z* Calcd for $C_{34}H_{38}O_8$: (M^+) 574.2567, found 574.2564. $[\alpha]_D^{20}$ = (+) 71.2 (*c* = 0.14, in $CHCl_3$). **Chiral separation** (98% ee): The enantioselectivity was determined by SFC using Chiralpak IA (Back pressure = 15 MPa, Flow (CO_2) = 2.4 mL/min, Flow (isopropanol) = 0.6 mL/min, 40 °C, λ = 250 nm). Retention time: t_R = 3.1 min (minor enantiomer) and 4.4 min (major enantiomer).

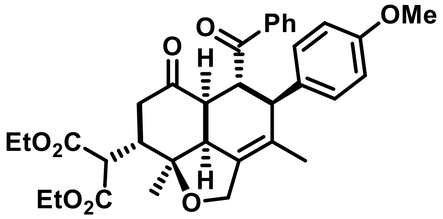

(2a^1*S*,4*S*,5*R*,5a*R*,8a*S*)-5-Benzoyl-4-(4-methoxyphenyl)-3,8a-dimethyl-2,2a1,4,5,5a,7,8,8a-octahydro-6*H*-naphtho[1,8-*bc*]furan-6-one (7): To a solution of **3aa** (25.4 mg, 0.06 mmol) in ethyl acetate (5 mL) was added Pd/C (10% w/w, 3.5 mg) and stirred for 6 hours at ambient temperature under an atmospheric pressure of hydrogen (1 atm). The solid was filtered off and filtrate was

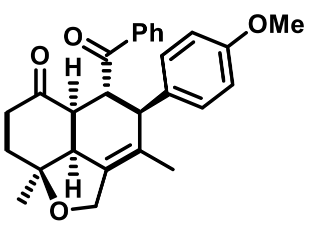

concentrated in vacuo. The residue was purified by silica gel flash column chromatography (10% EtOAc in hexane) to afford **7** (22.2 mg, 0.053 mmol, 89%) as thick colorless oil. R_f = 0.4 (20% EtOAc in hexane). **^{1}H NMR** (400 MHz, $CDCl_3$) δ 7.89 (2H, d, *J* = 7.3 Hz, benzoyl-*ortho*-Ar-*H*), 7.52 (1H, dd, *J* = 7.3,7.3 Hz, benzoyl-*para*-Ar-*H*), 7.40 (2H, dd, *J* = 7.3, 7.3 Hz, benzoyl-*meta*-Ar-*H*), 7.01 (2H, *J* = 8.7 Hz, Ar-*H*), 6.79 (2H, *J* = 8.7 Hz, Ar-*H*), 4.81 (1H, dd, *J* =1.5, 4.6 Hz, C*H*COPh), 4.55 (1H, d, *J* = 12.6 Hz, OCH_2C), 4.44 (1H, apparent quintet of doublet, *J* = 1.7, 12.6 Hz, OCH_2C), 3.80 (1H, s, C*H*Ar), 3.75 (3H, s, OCH_3), 2.83 (1H, dd, *J* = 1.5, 6.8 Hz, CH_2COC*H*CH), 2.70–2.72 (1H, m, CC*H*C=C), 2.52 (1H, ddd, *J* =4.8, 10.5, 15.6 Hz, CH_2CH_2CO), 2.31 (1H, dt, *J* =5.5, 17.8 Hz, CH_2CH_2CO), 2.02–2.09 (1H, m, CH_2CH_2CO), 1.84 (1H, ddd, *J* =4.8, 10.5, 15.6 Hz, CH_2CH_2CO), 1.41 (3H, s, CCH_3), 1.38 (3H, br s, $C{=}CCH_3$). **^{13}C{^{1}H} NMR** (100 MHz, $CDCl_3$) δ 208.6, 201.8, 158.1, 135.5, 134.9, 134.4, 133.1, 130.1, 128.6 (two carbons), 126.6, 113.6, 81.6, 67.6, 55.0, 47.5, 46.8, 45.4, 44.1, 35.4, 32.1, 26.4, 17.9. **HRMS** (EI+): *m/z* Calcd for $C_{27}H_{28}O_4$: (M^+) 416.1988, found 416.1986. $[\alpha]_D^{23}$ = (+) 177.3 (*c* = 0.11, in $CHCl_3$). $[\alpha]_D^{20}$ = (+) 110.7 (*c* = 0.06, in $CHCl_3$). **Chiral separation** (98% ee): The enantioselectivity was determined by SFC using Chiralpak IA (Back pressure = 15 MPa, Flow (CO_2) = 3.0 mL/min, Flow (isopropanol) = 0.3 mL/min, 40 °C, λ = 250 nm). Retention time: t_R = 5.5 min (minor enantiomer) and 5.7 min (major enantiomer).

Isolation of η^3-oxaallyl nickelacycle (8)

To a benzene solution of $Ni(cod)_2$ (275.0 mg, 1.0 mmol) and IPr (389.0 mg, 1.0 mmol) was added **1a** (176.0 mg, 1.0 mmol) at room temperature. Resulting dark brown mixture was stirred for 5 minutes and then volatiles were removed under vacuum to give brown solids (622.0 mg, 1.0 mmol, 99%). A single crystal suitable for X-ray diffraction analysis was obtained by recrystallization from Et_2O/hexane at –30 °C, indicated the formation of η^3-oxaallyl nickelacycle of structure **8**. The characteristic resonances of the **8** were attributed as follows;

^{1}H NMR (400 MHz, C_6D_6): δ 7.12–7.32 (6H, m, Ar*H*), 6.66 (2H, s, NC*H*=C*H*N), 5.95 (1H, d, *J* = 10.1 Hz, C*H*=CHCO), 5.49 (1H, d, *J* = 10.1 Hz, CH=C*H*CO), 4.60 (1H, dd, *J* = 6.2, 2.0 Hz, η^3 CH(C*H*=CO)Ni), 3.87 (1H, d, *J* = 9.4 Hz, OCH_2C), 3.72 (1H, d, *J* = 9.4 Hz, OCH_2C), 3.19 (2H, sept, *J* = 6.8 Hz, *iPr*-C*H*), 2.99 (2H, sept, *J* = 6.8 Hz, *iPr*-C*H*), 2.74 (1H, d, *J* = 5.9 Hz, CC*H*C=C), 1.47 (6H, d, *J* = 6.8 Hz, *iPr*-CH_3), 1.43 (6H, d, *J* = 6.8 Hz, *iPr*-CH_3), 1.20 (3H, s, $C{=}CCH_3$), 1.14 (3H, s, CCH_3), 1.03 (12H, d, *J* = 7.2 Hz, *s*). **^{13}C NMR {^{1}H} NMR** (100 MHz, C_6D_6): δ 190.6 (N*C*N), 163.1 ((CH=CH)*C*OCHNi), 147.7 (COCH=*C*HC), 146.8 (*Ar*), 146.5 (*Ar*), 142.0 (CH_2*C*=C), 141.0

($C{=}\mathit{C}CH_3$), 136.5 (*Ar*), 130.1 (*Ar*), 125.4 (CO*C*H=CH), 124.5 (*Ar*), 124.3 (*Ar*), 124.2 (*Ar*), 124.0 (N*C*H=*C*HN), 75.8 ((CH=CH)CO*C*HNi), 75.4 ($CH{=}CH\mathit{C}CH_3$), 59.9 ($O\mathit{C}H_2C$), 53.1 (C*C*HC=C), 29.0 (*iPr*-$\mathit{C}HCH_3$), 28.9 ($C\mathit{C}H_3$), 27.3 ($C{=}C\mathit{C}H_3$), 26.5 (*g*), 26.4 (*iPr*-$\mathit{C}H_3$), 26.3 (*iPr*-$\mathit{C}H_3$), 22.8 (*iPr*-$\mathit{C}H_3$), 22.6 (*iPr*-$\mathit{C}H_3$). Elemental analysis did not give perfect result probably due to extremely high sensitivity of the complex to the air and moisture. However, best result could be obtained as follows; Elemental Analysis: C, 73.20; H, 7.76; N, 4.49; Ni, 9.41; O, 5.13, found C, 72.66; H, 7.69; N, 4.69.

Supplementary Figures

Supplementary Figure 2. ^{1}H and ^{13}C-NMR spectra of product 1f

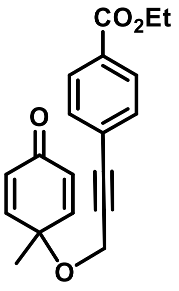

1f

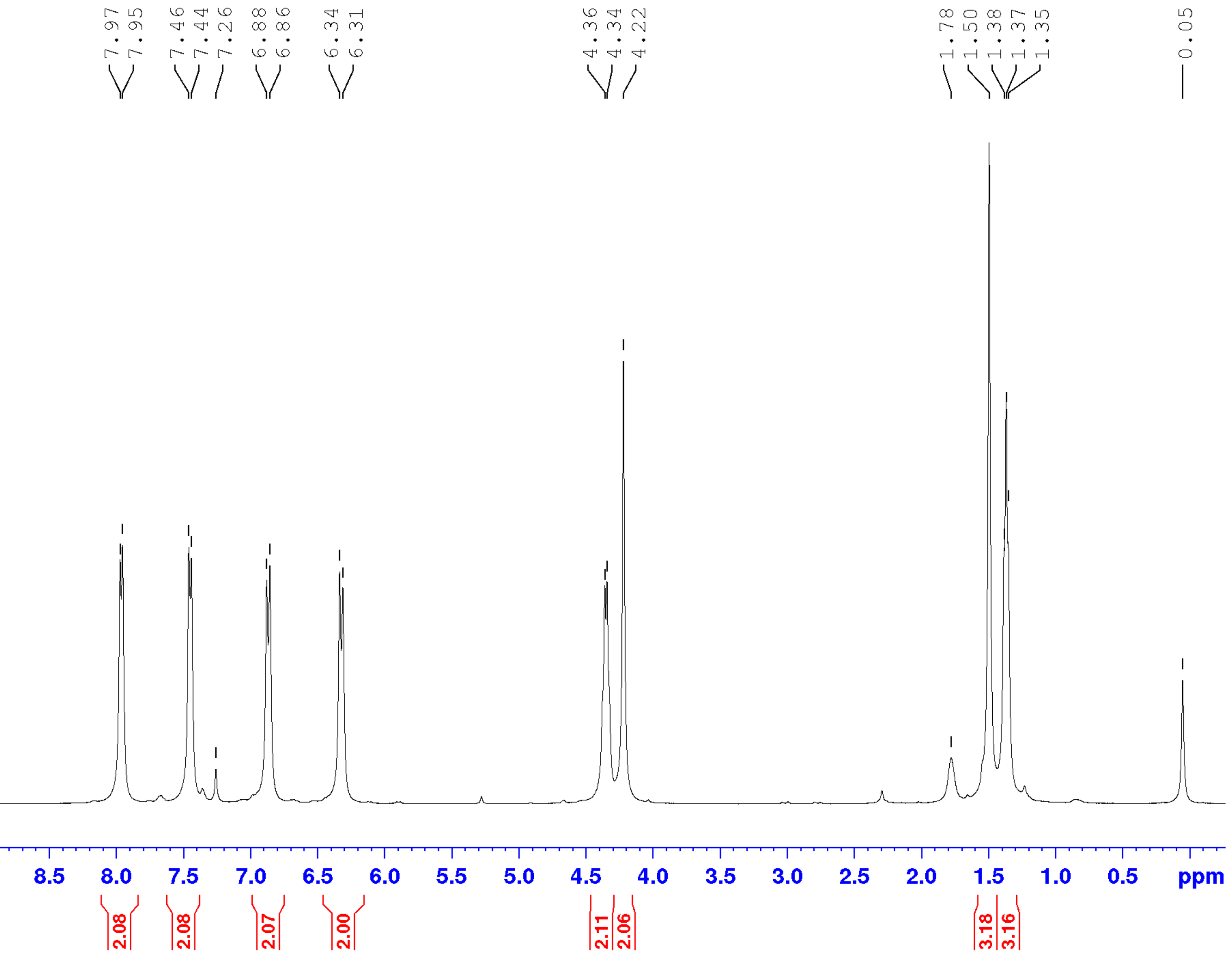

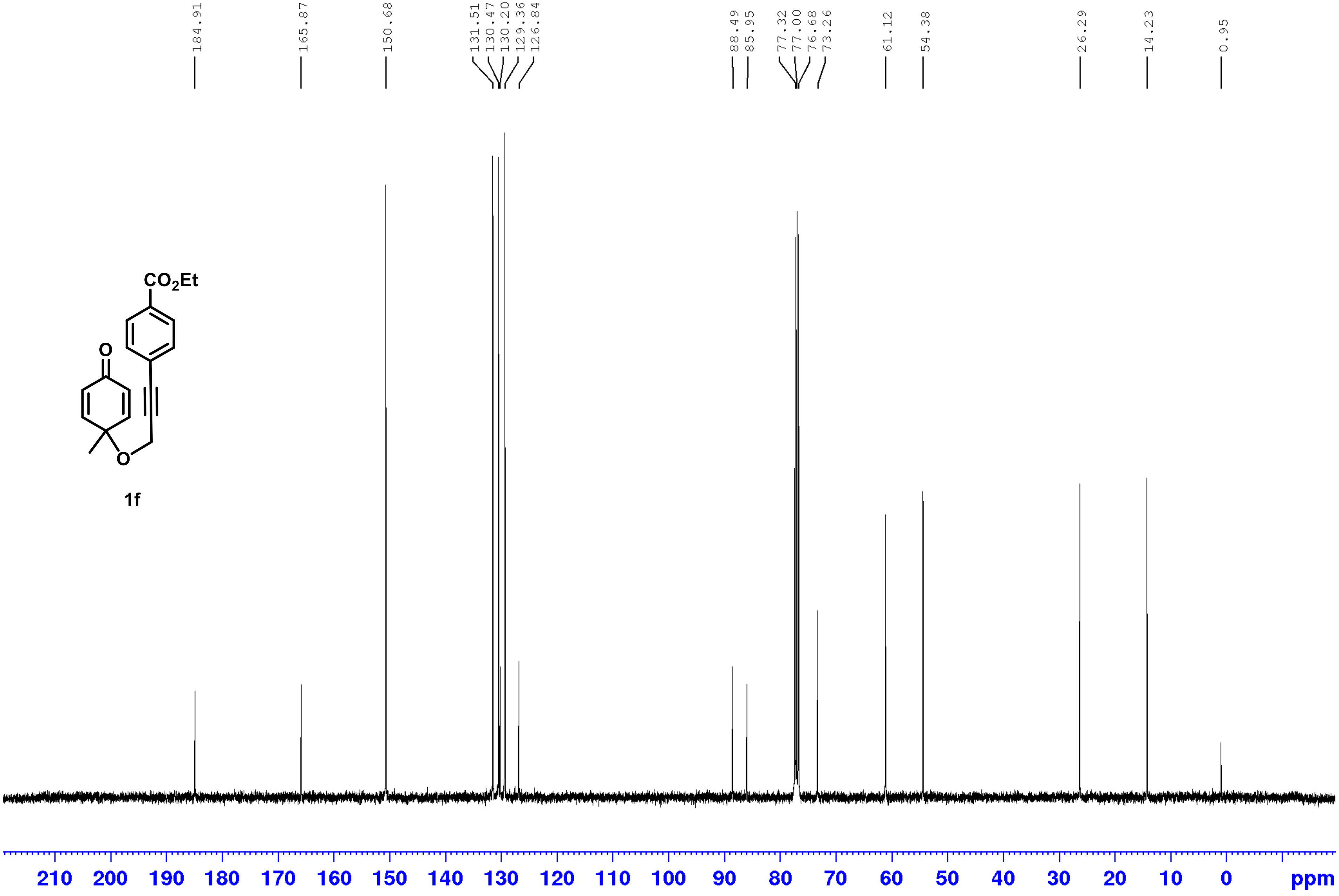
184.91
165.87
150.68
131.51
130.47
130.20
129.36
126.84
88.49
85.95
77.32
77.00
76.68
73.26
61.12
54.38
26.29
14.23
0.95
CO2Et
O
O
1f
210 200 190 180 170 160 150 140 130 120 110 100 90 80 70 60 50 40 30 20 10 0 ppm

Supplementary Figure 3. ^{1}H and ^{13}C-NMR spectra of product 1g

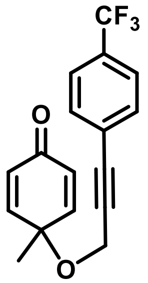

1g

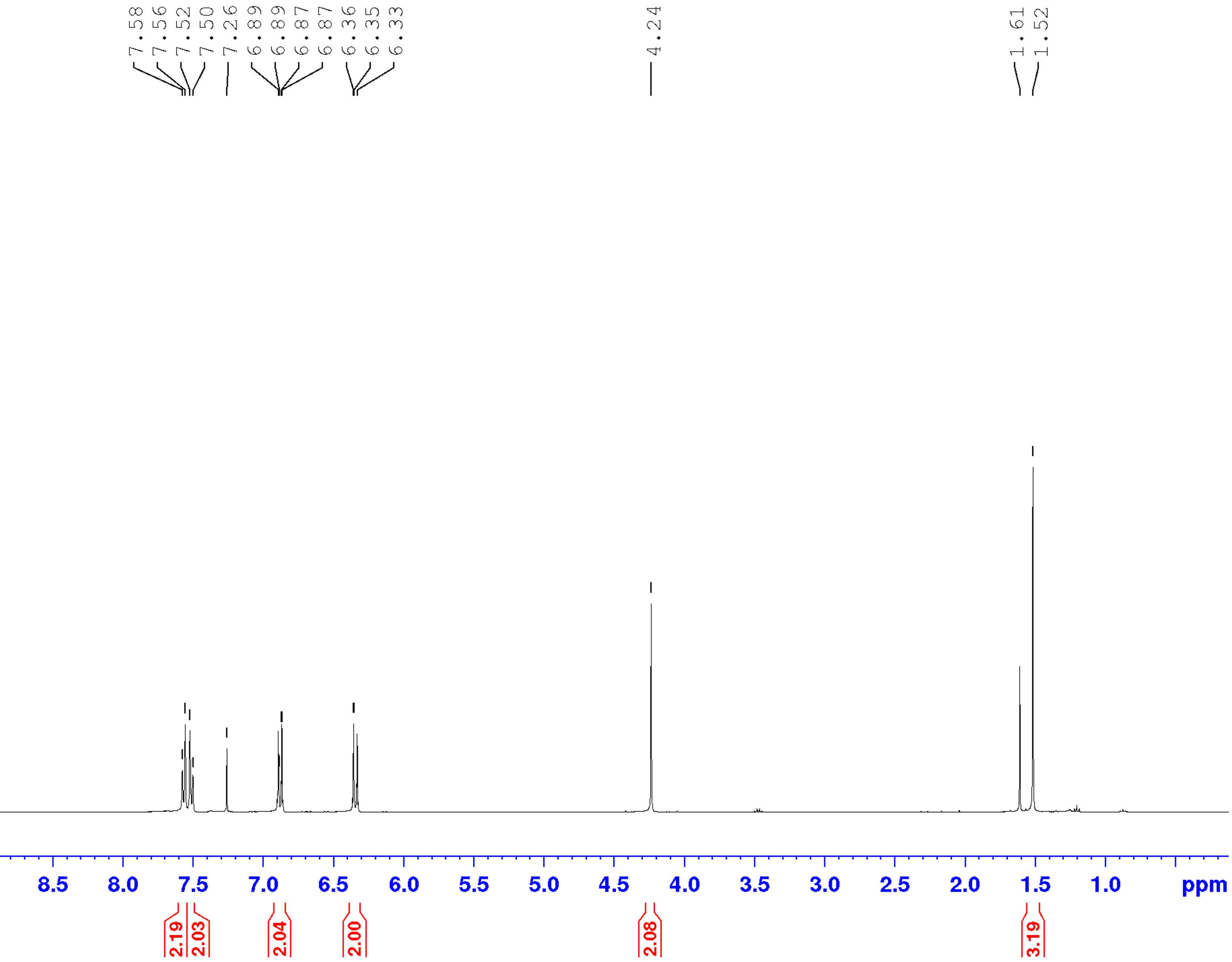

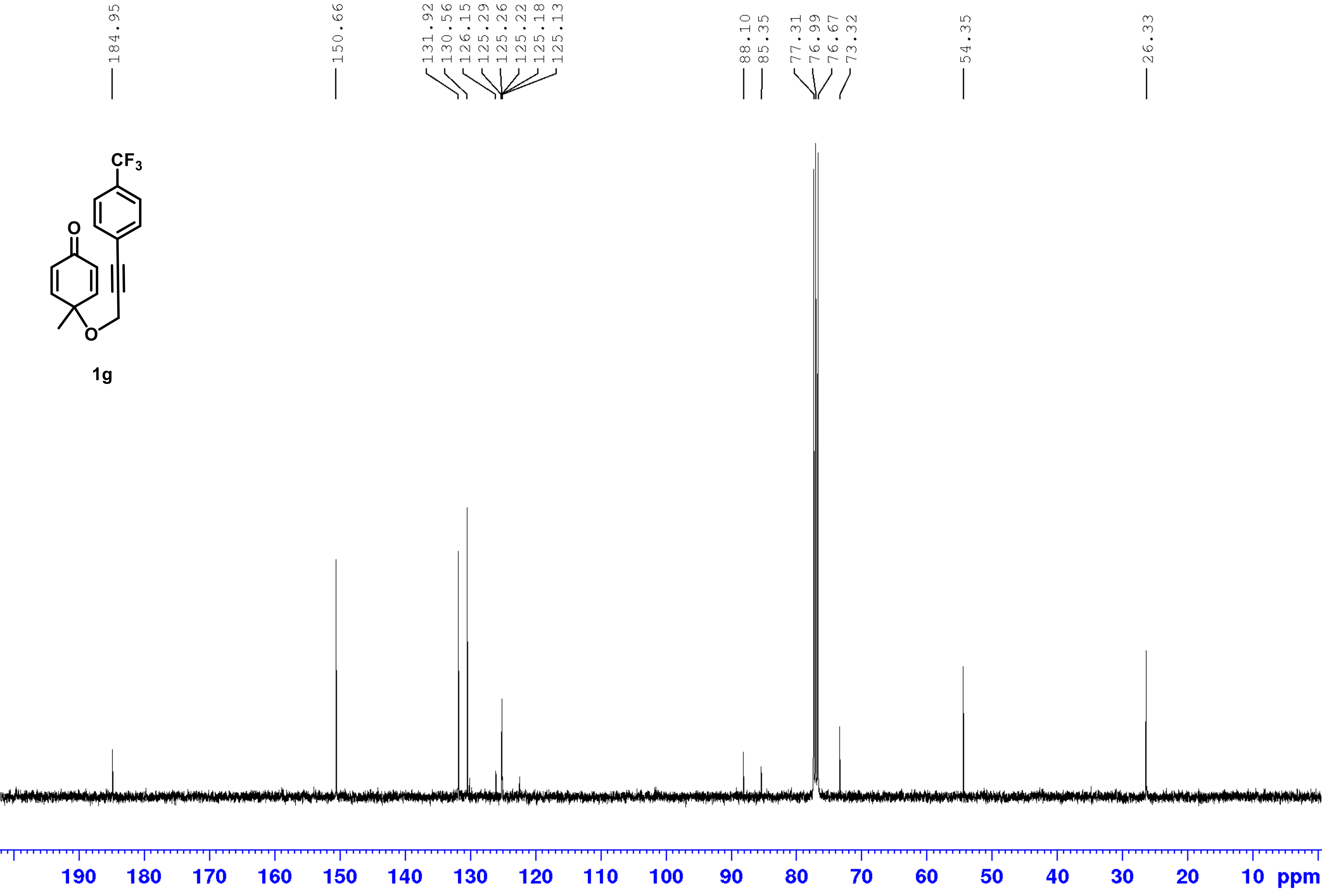
184.95
150.66
131.92
130.56
126.15
125.29
125.26
125.22
125.18
125.13
88.10
85.35
77.31
76.99
76.67
73.32
54.35
26.33
CF3
O
O
1g
190 180 170 160 150 140 130 120 110 100 90 80 70 60 50 40 30 20 10 ppm

Supplementary Figure 4. ¹H and ¹³C-NMR spectra of product 1i

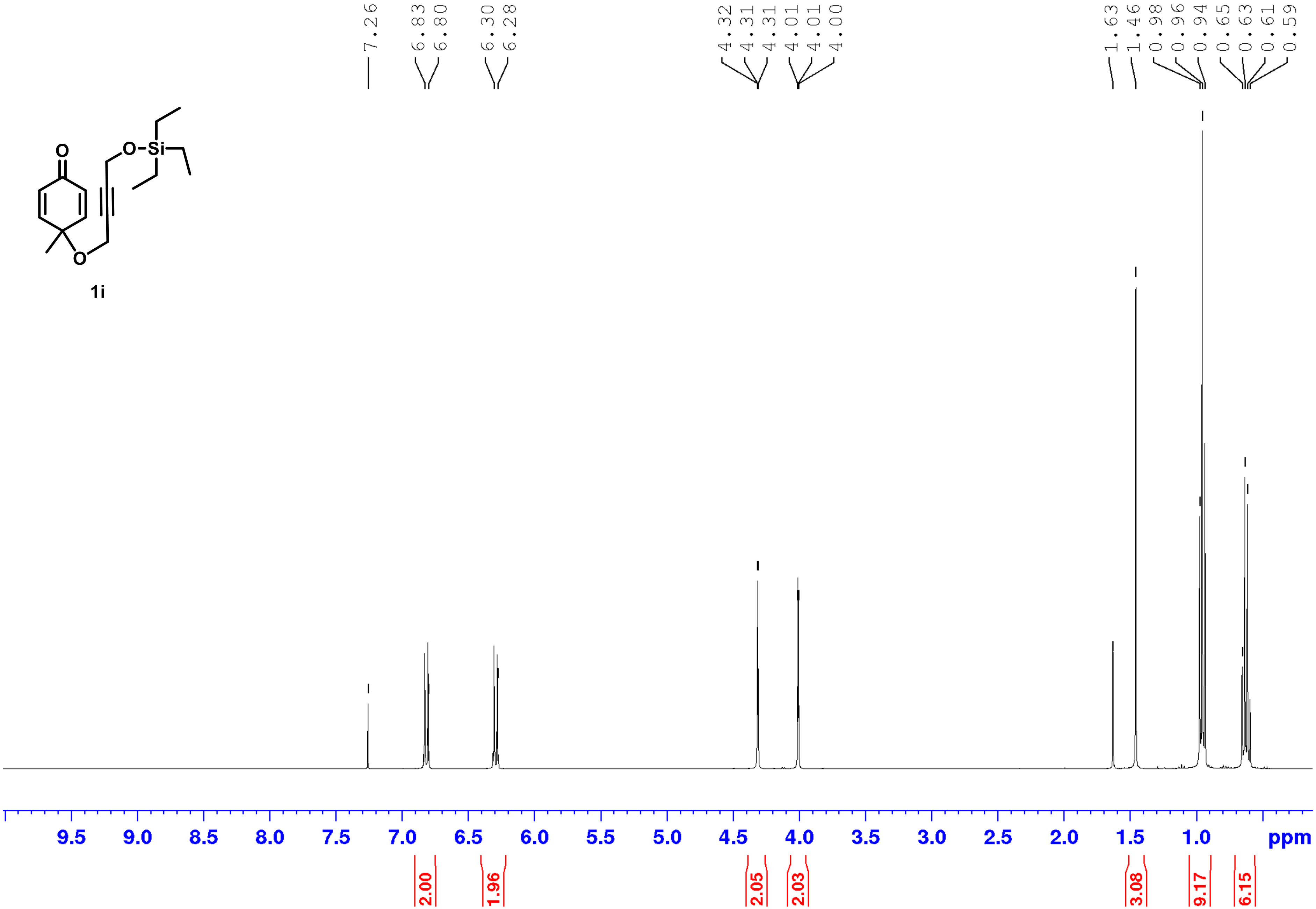

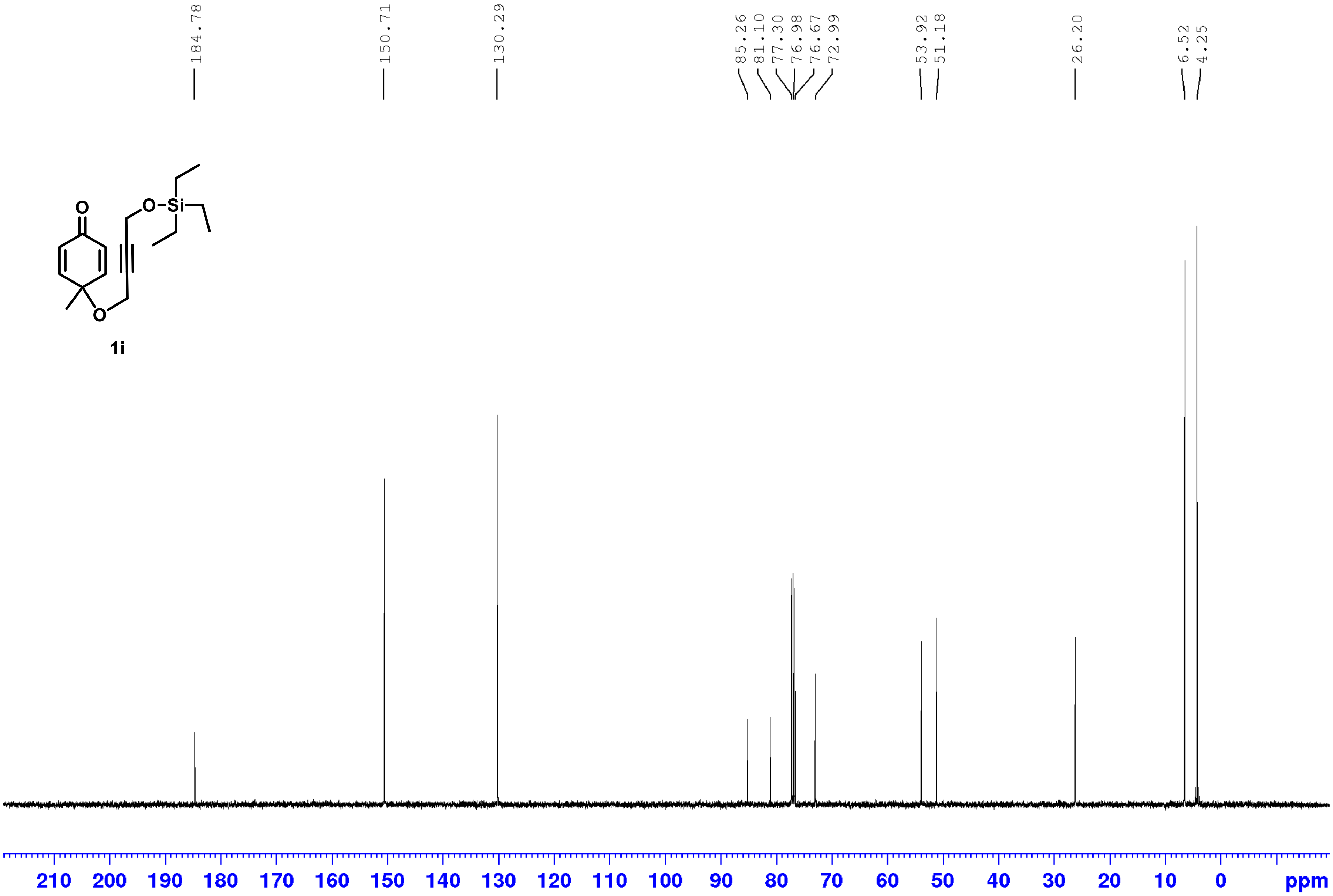
184.78
150.71
130.29
85.26
81.10
77.30
76.98
76.67
72.99
53.92
51.18
26.20
6.52
4.25
O
O
O
Si
1i
210 200 190 180 170 160 150 140 130 120 110 100 90 80 70 60 50 40 30 20 10 0 ppm

Supplementary Figure 5. ^{1}H, ^{13}C-NMR and SFC spectra of product 3aa

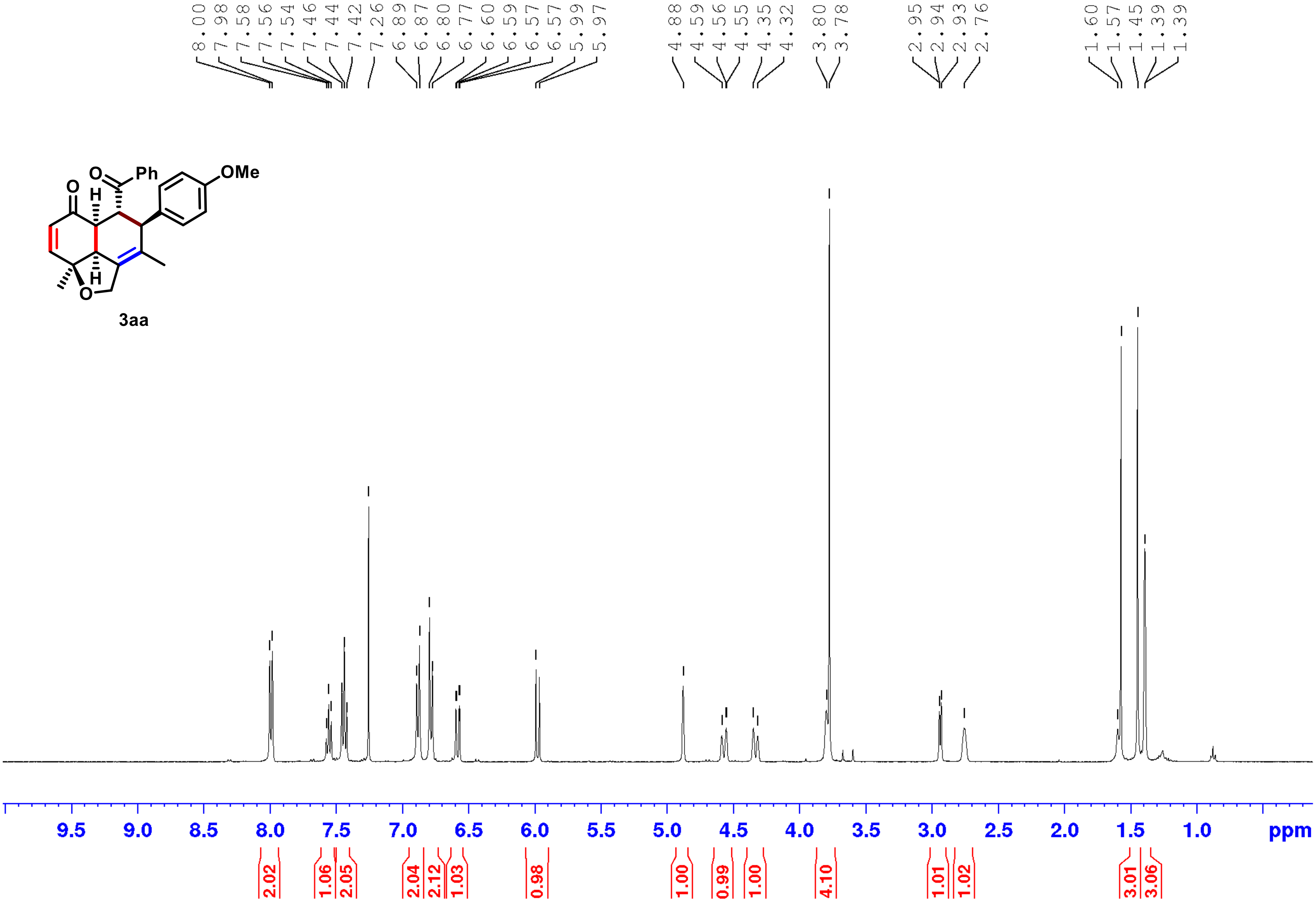

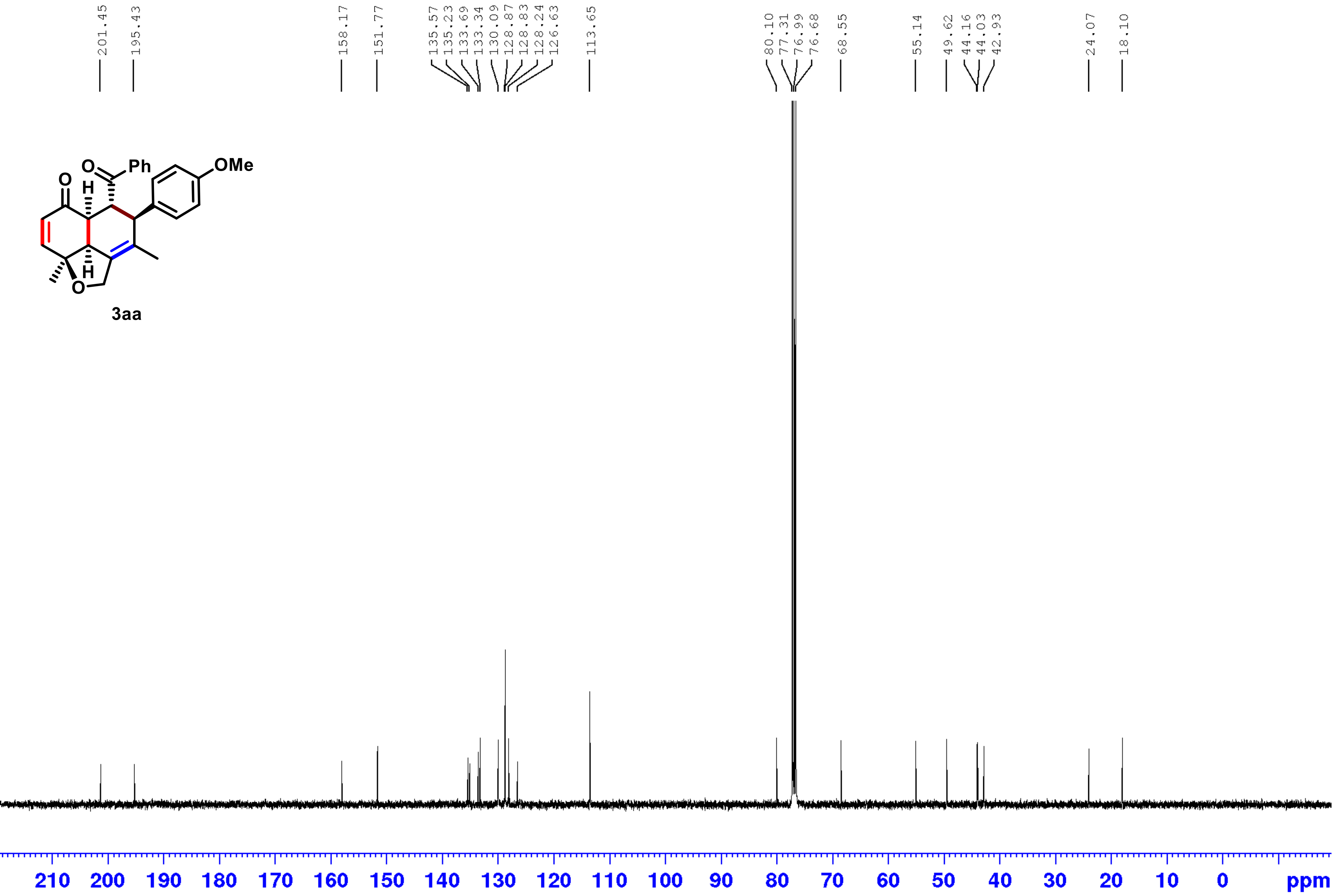
201.45
195.43
158.17
151.77
135.57
135.23
133.69
133.34
130.09
128.87
128.83
128.24
126.63
113.65
80.10
77.31
76.99
76.68
68.55
55.14
49.62
44.16
44.03
42.93
24.07
18.10
O
Ph
OMe
O
H
H
O
3aa
210
200
190
180
170
160
150
140
130
120
110
100
90
80
70
60
50
40
30
20
10
0
ppm

Chiral Separation (SFC): Chiralpak IA; Flow (CO_2) = 2.4 mL/min; Flow (isopropanol) = 0.6 mL/min; T = 25 °C; λ = 250 nm; Back pressure = 15 Mpa

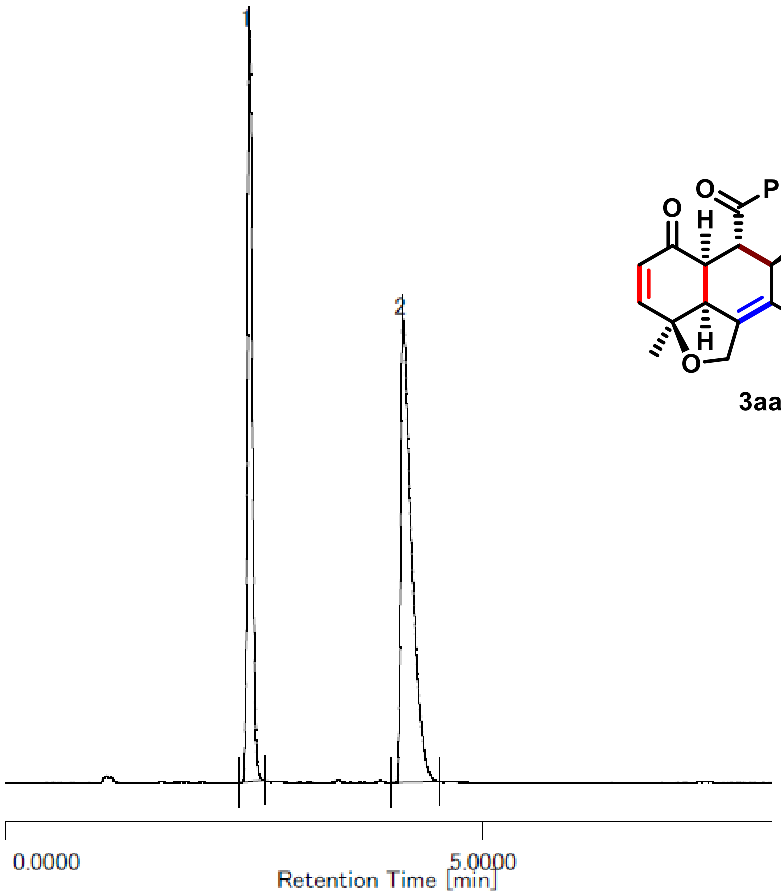

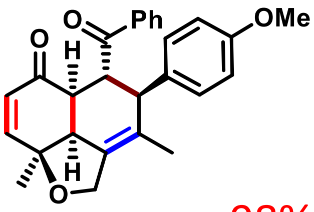

3aa

98% ee

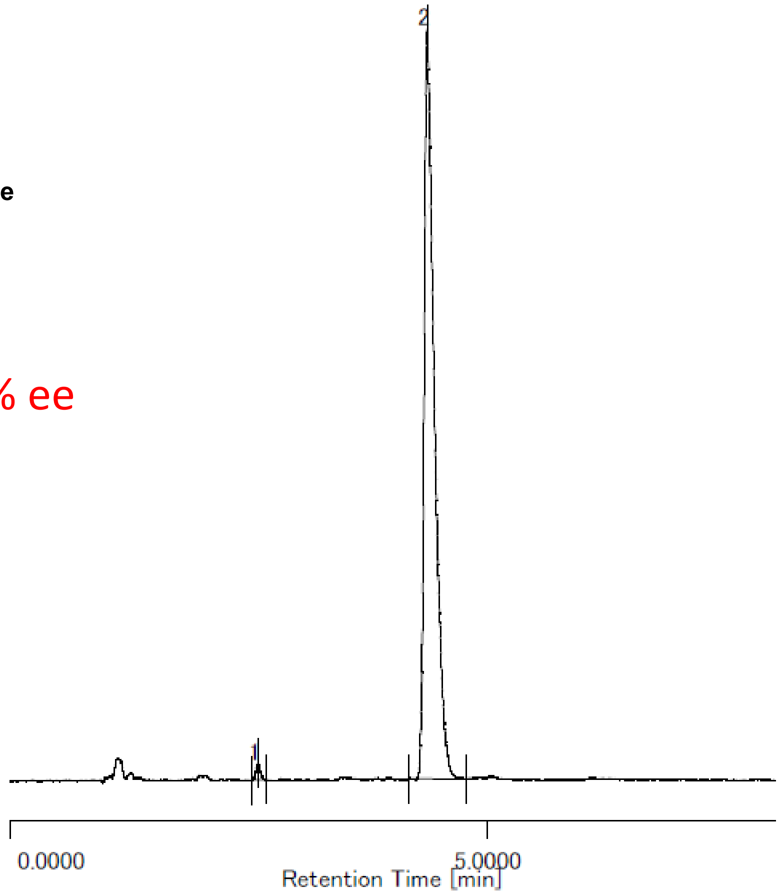

Chromatogram R-551

Name

#	CH	tR	Area	Height	Area%
1	10	2.5583	9164659	2561898	42.586
2	10	4.1600	12355542	1580788	57.414

Chromatogram R-651

Name

#	CH	tR	Area	Height	Area%
1	10	2.5967	58151	17411	0.998
2	10	4.3633	5768852	785440	99.002

Supplementary Figure 6. ^{1}H, ^{13}C-NMR and SFC spectra of product 3ab

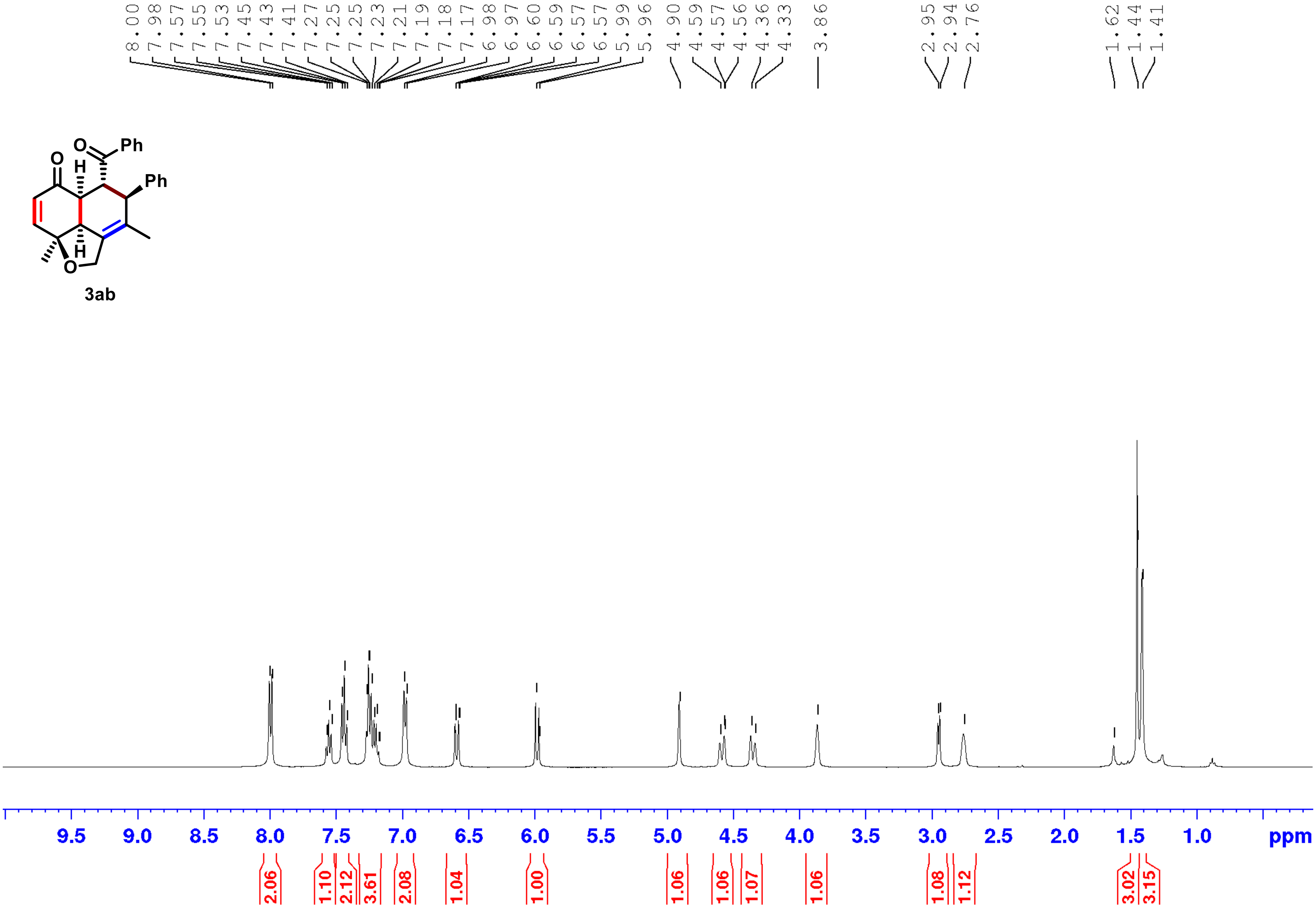

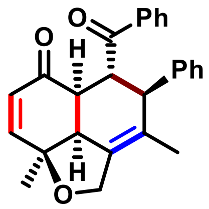

O
Ph
O
H
Ph
H
O

3ab

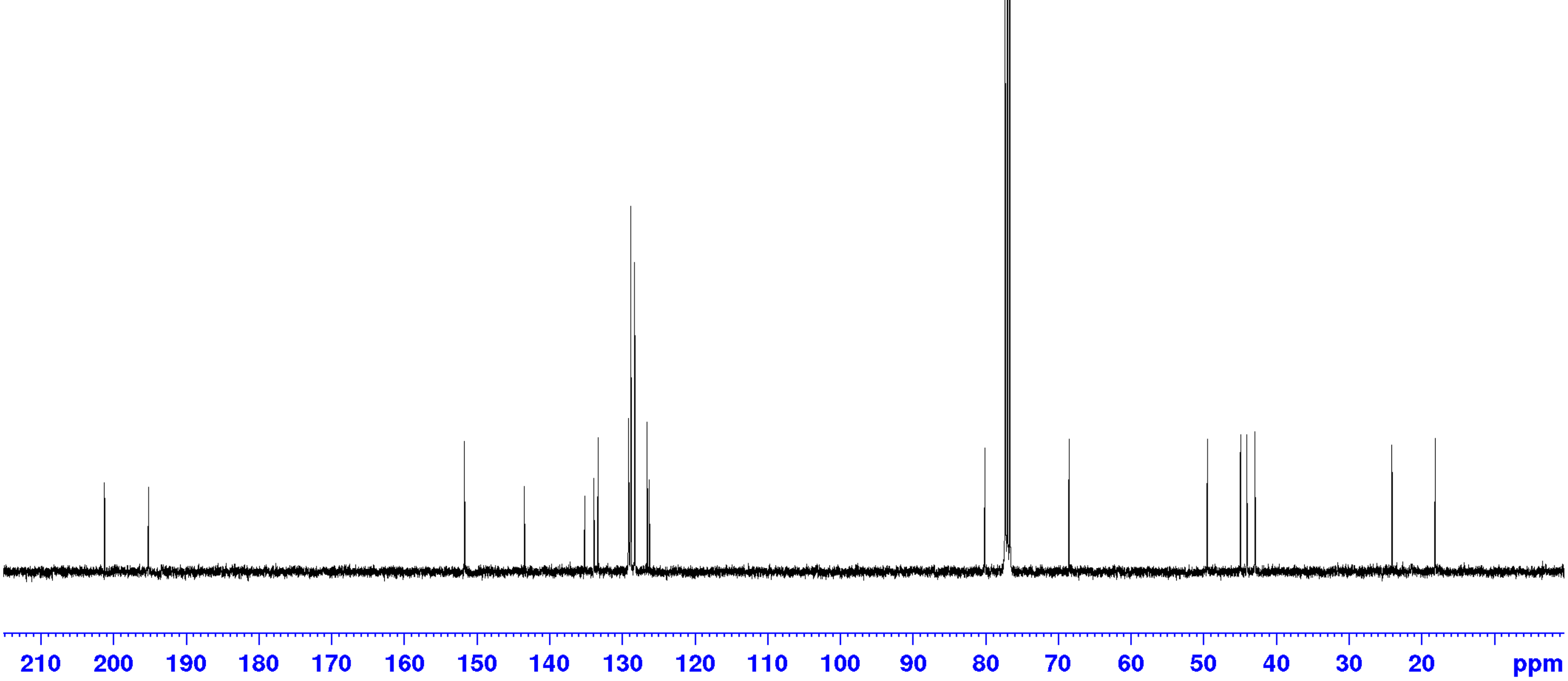

201.27
195.23
151.74
143.48
135.17
133.93
133.34
129.14
128.85
128.83
128.29
128.24
126.58
126.29
80.08
77.31
77.00
76.68
68.52
49.49
44.91
44.05
42.92
24.07
18.11
210 200 190 180 170 160 150 140 130 120 110 100 90 80 70 60 50 40 30 20 ppm

Chiral Separation (SFC): Chiralpak IA; Flow (CO_2) = 3.0 mL/min; Flow (isopropanol) = 0.3 mL/min; T = 25 ^oC; λ = 250 nm; Back pressure = 15 Mpa

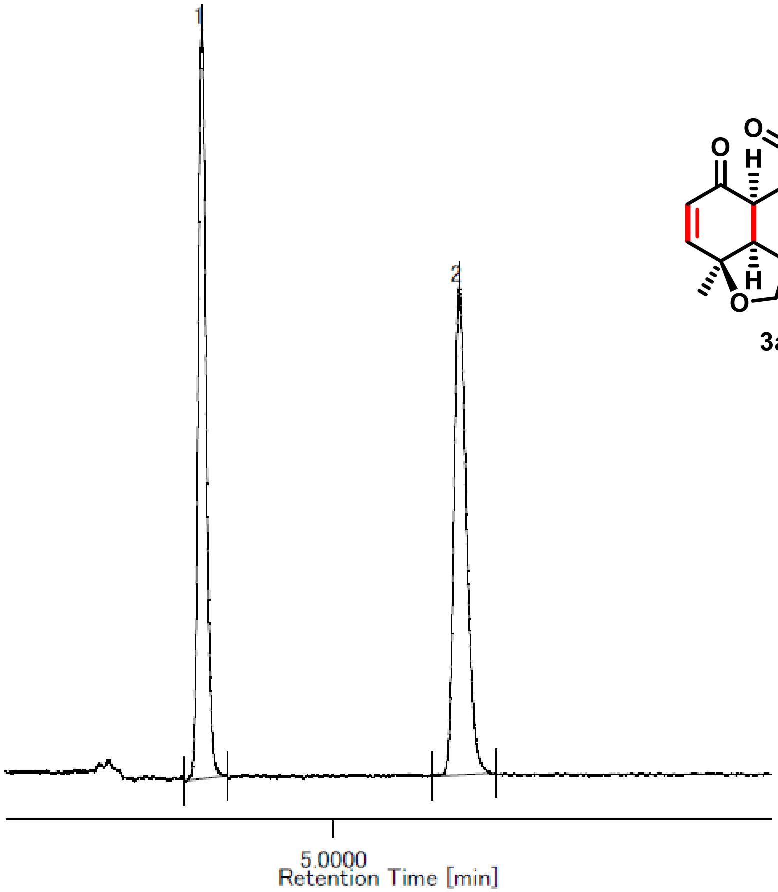

Chromatogram Name R−680

#	CH	tR	Area	Height	Area%
1	9	3.7917	1830750	361876	49.474
2	9	6.1450	1869713	235883	50.526

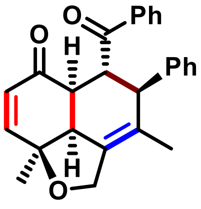

3ab

98% ee

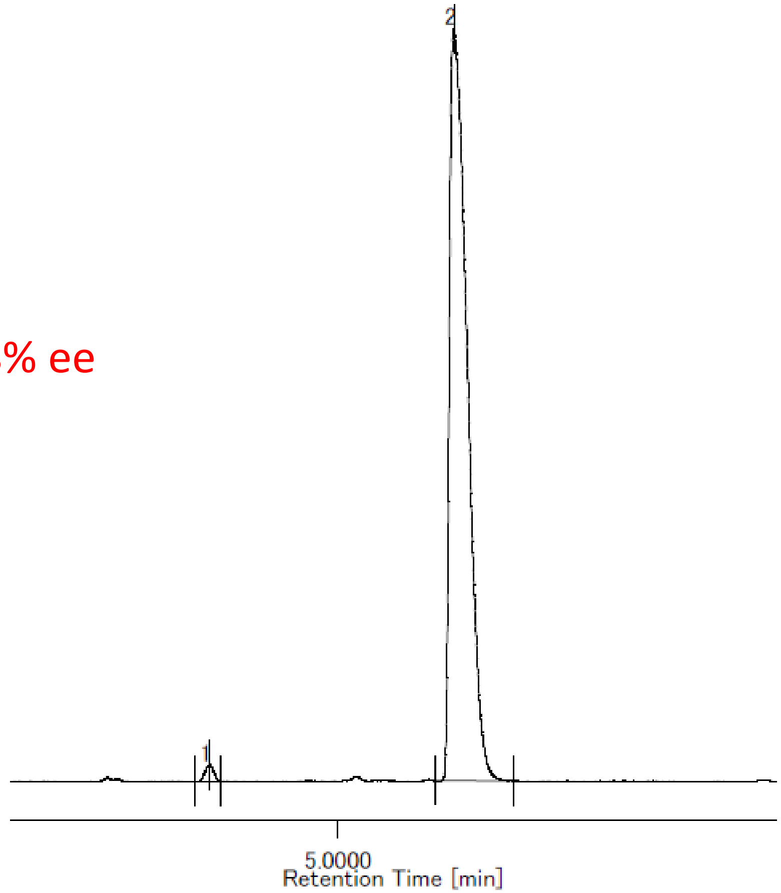

Chromatogram Name R−678−chalcone

#	CH	tR	Area	Height	Area%
1	9	3.8200	174880	31805	1.051
2	9	6.0533	16457123	1476173	98.949

Supplementary Figure 7. ^{1}H, ^{13}C-NMR and SFC spectra of product 3ac

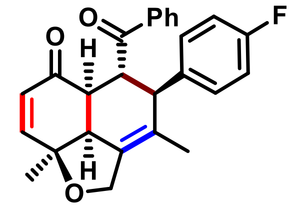

3ac

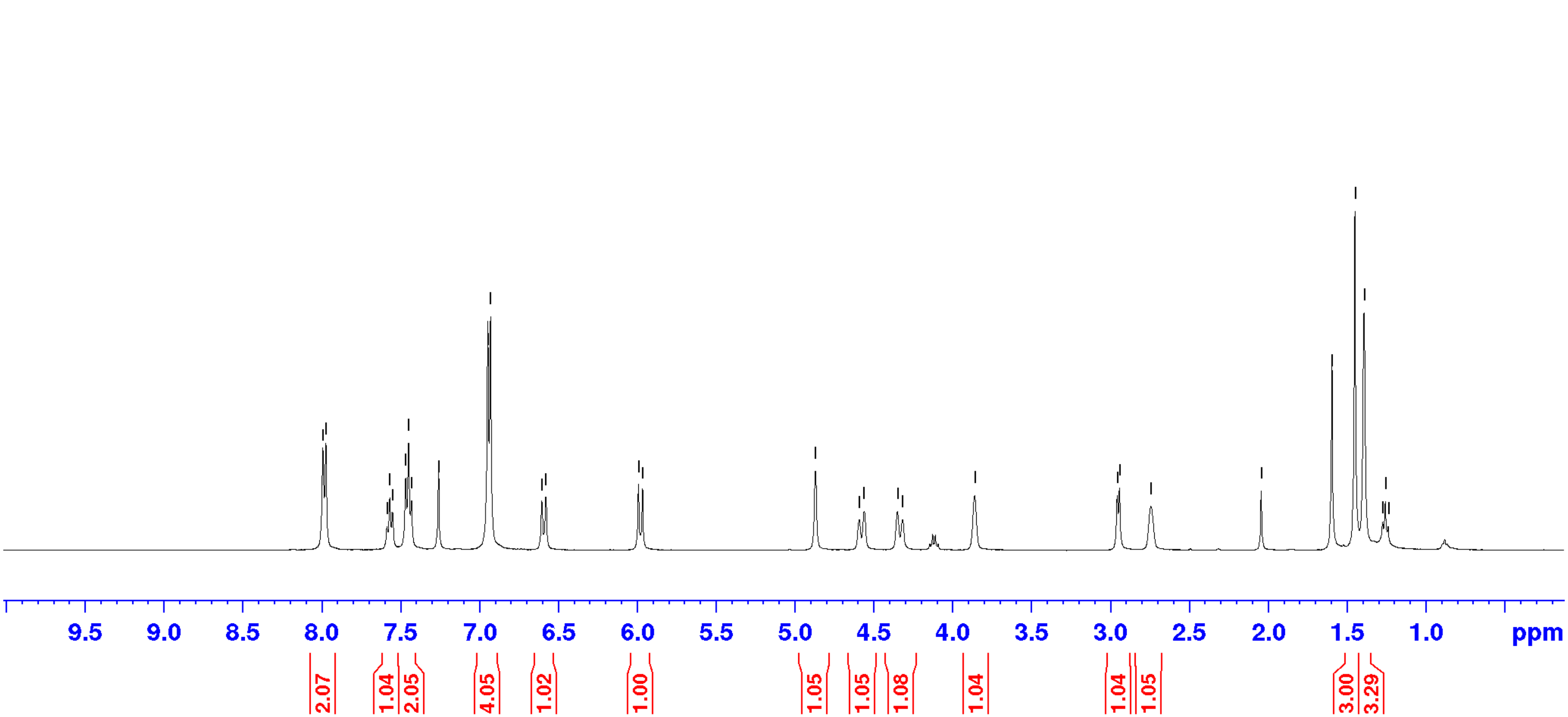

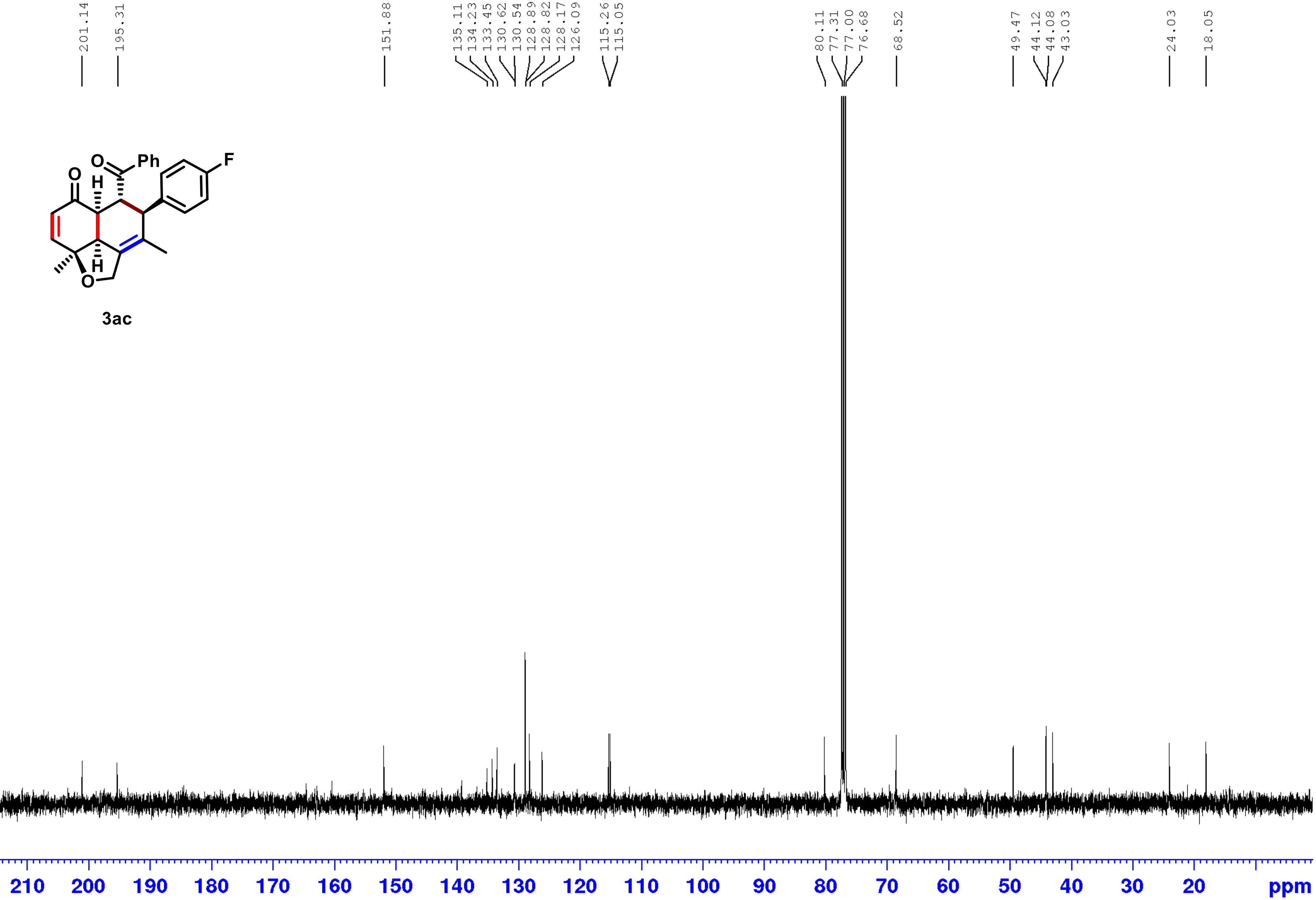
201.14
195.31
151.88
135.11
134.23
133.45
130.62
130.54
128.89
128.82
128.17
126.09
115.26
115.05
80.11
77.31
77.00
76.68
68.52
49.47
44.12
44.08
43.03
24.03
18.05
O
Ph
F
O
H
H
O
3ac
210 200 190 180 170 160 150 140 130 120 110 100 90 80 70 60 50 40 30 20 ppm

Chiral Separation (SFC): Chiralpak IA; Flow (CO_2) = 3.0 mL/min; Flow (isopropanol) = 0.3 mL/min; T = 25 ^{o}C; λ = 250 nm; Back pressure = 15 Mpa

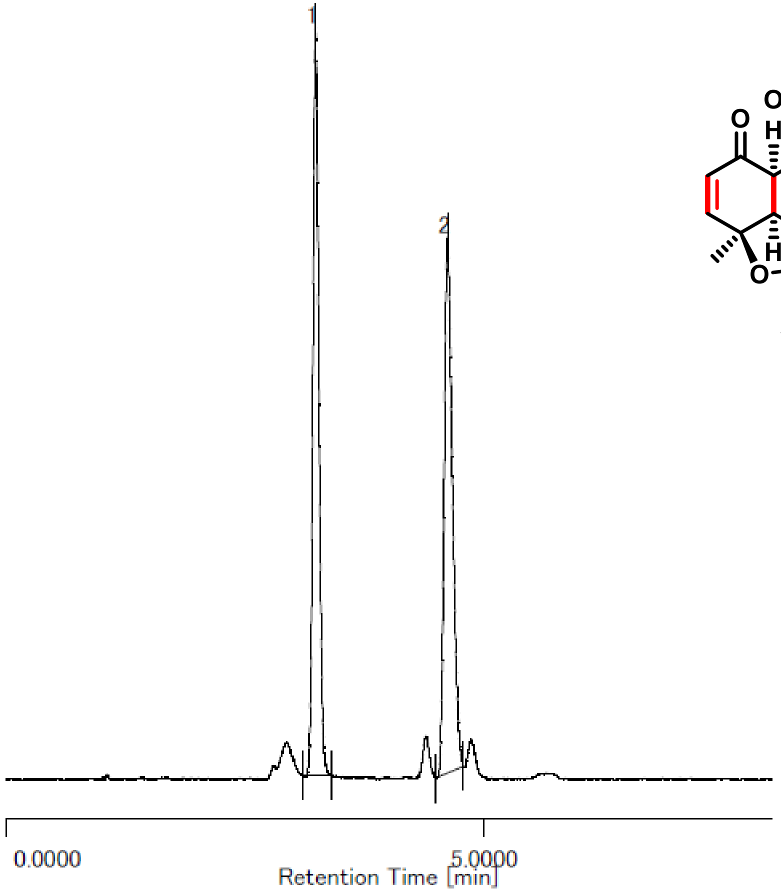

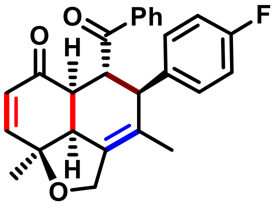

3ac

98% ee

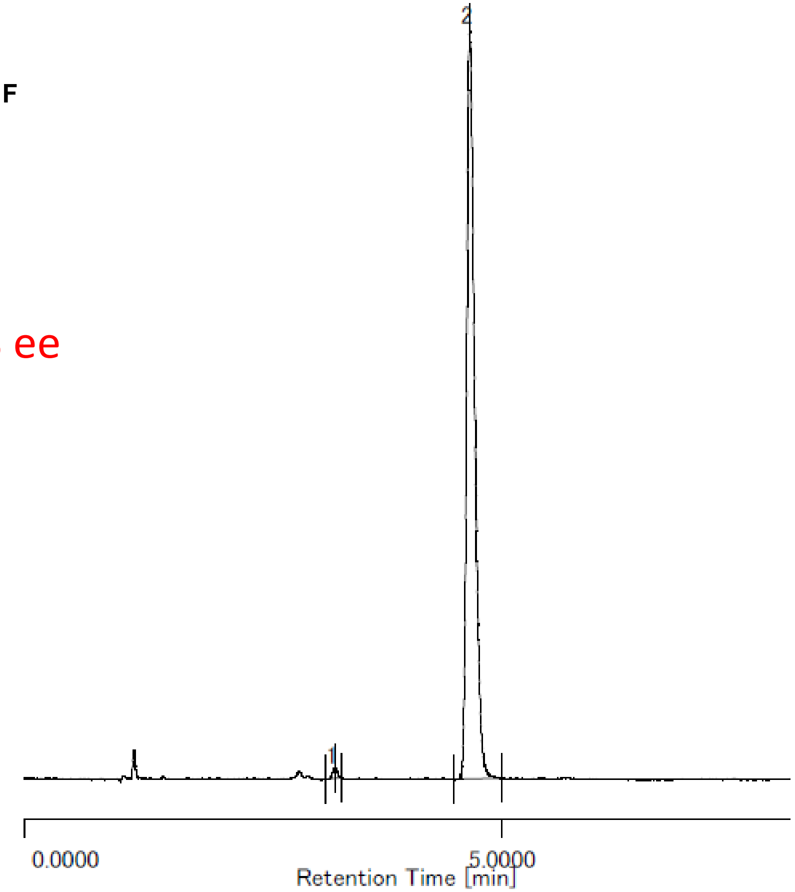

Chromatogram Name R-681

#	CH	tR	Area	Height	Area%
1	10	3.2367	3947525	915848	50.389
2	10	4.6133	3886567	655119	49.611

Chromatogram Name R-677-p-F-chalcone

#	CH	tR	Area	Height	Area%
1	10	3.2517	41349	11962	0.878
2	10	4.6583	4666885	813530	99.122

Supplementary Figure 8. ^{1}H, ^{13}C-NMR and SFC spectra of product 3ad

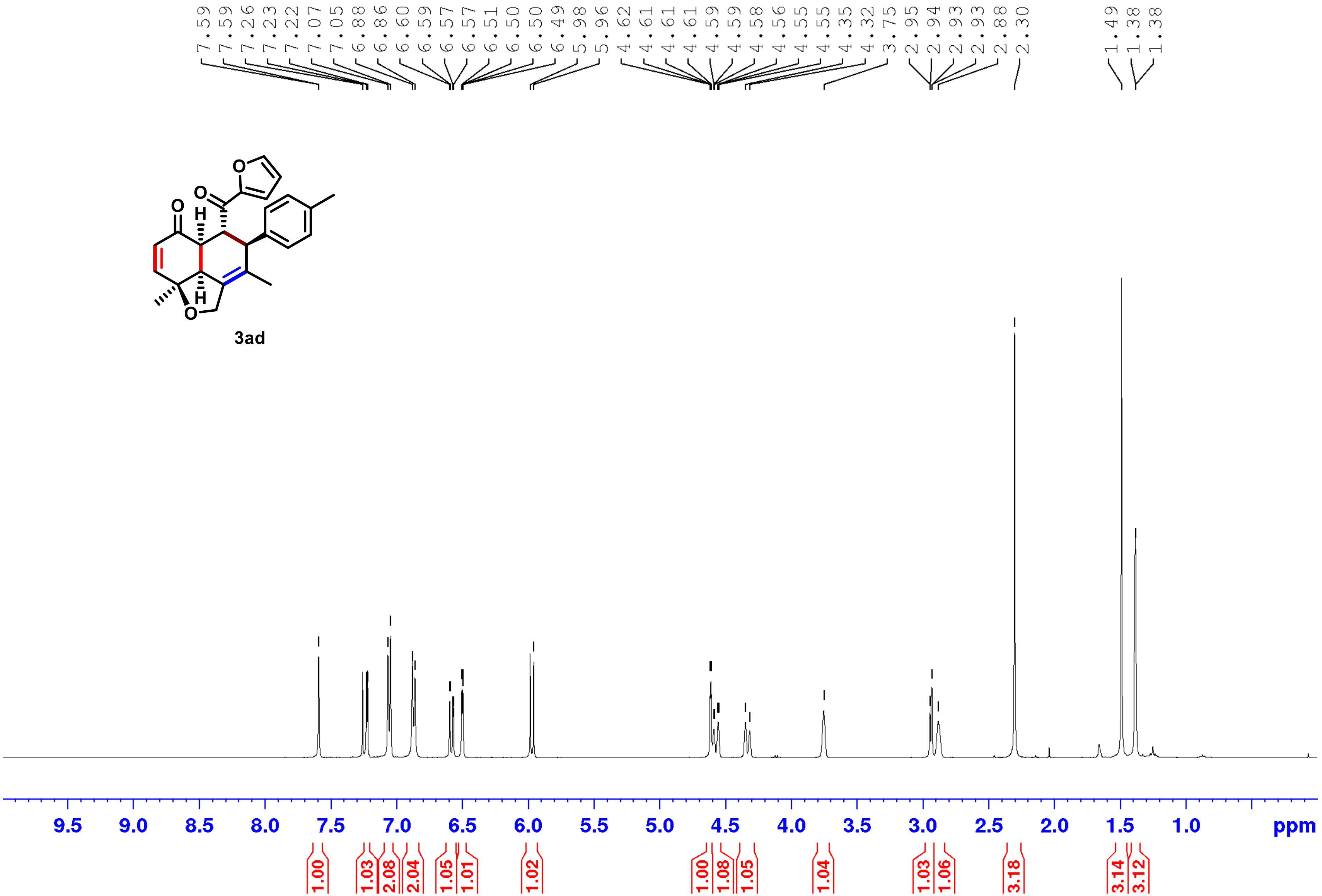

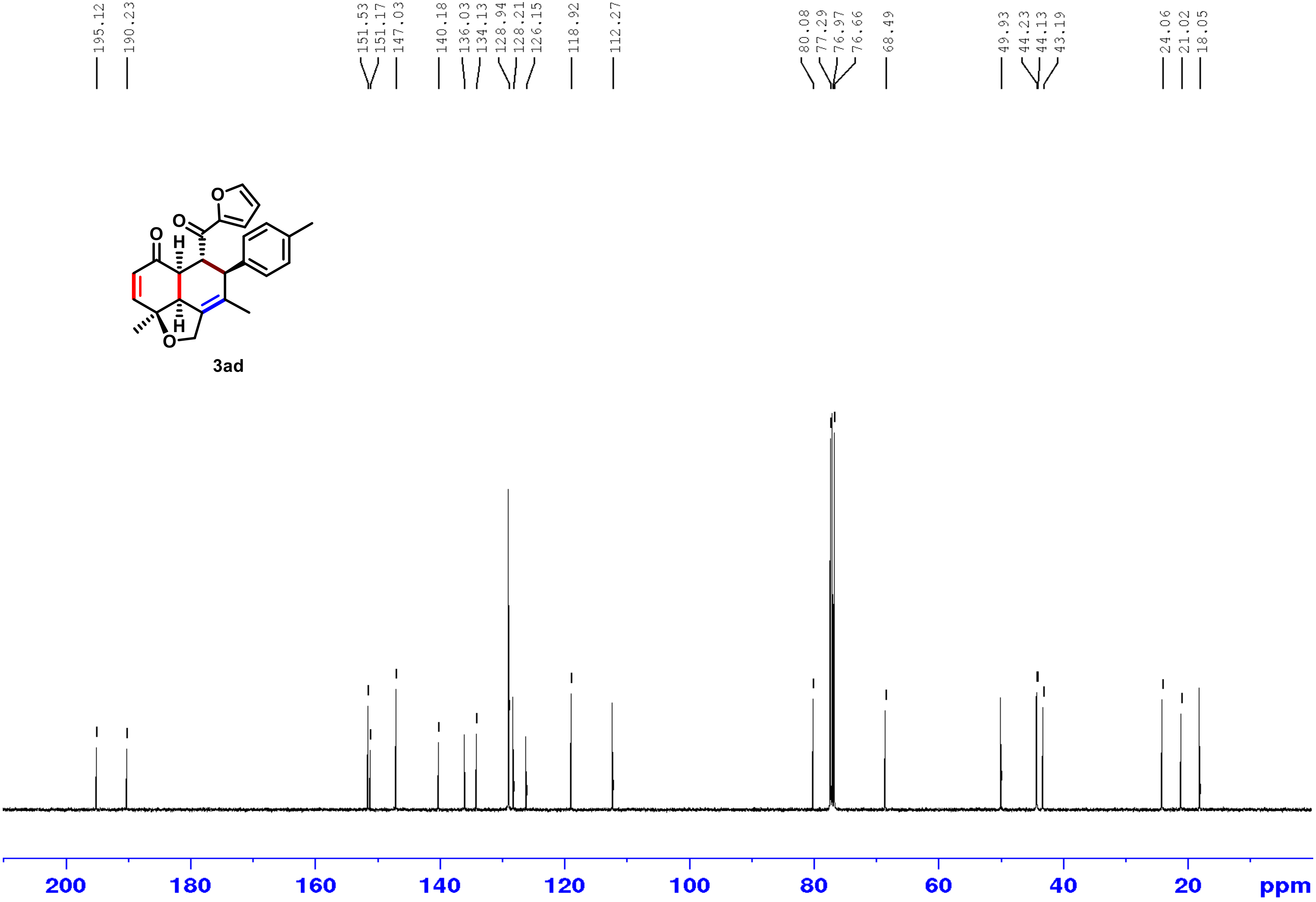
195.12
190.23
151.53
151.17
147.03
140.18
136.03
134.13
128.94
128.21
126.15
118.92
112.27
80.08
77.29
76.97
76.66
68.49
49.93
44.23
44.13
43.19
24.06
21.02
18.05
3ad
200
180
160
140
120
100
80
60
40
20
ppm

Chiral Separation (SFC): Chiralpak IA; Flow (CO_2) = 3.0 mL/min; Flow (isopropanol) = 0.3 mL/min; T = 25 ^{o}C; λ = 250 nm; Back pressure = 15 Mpa

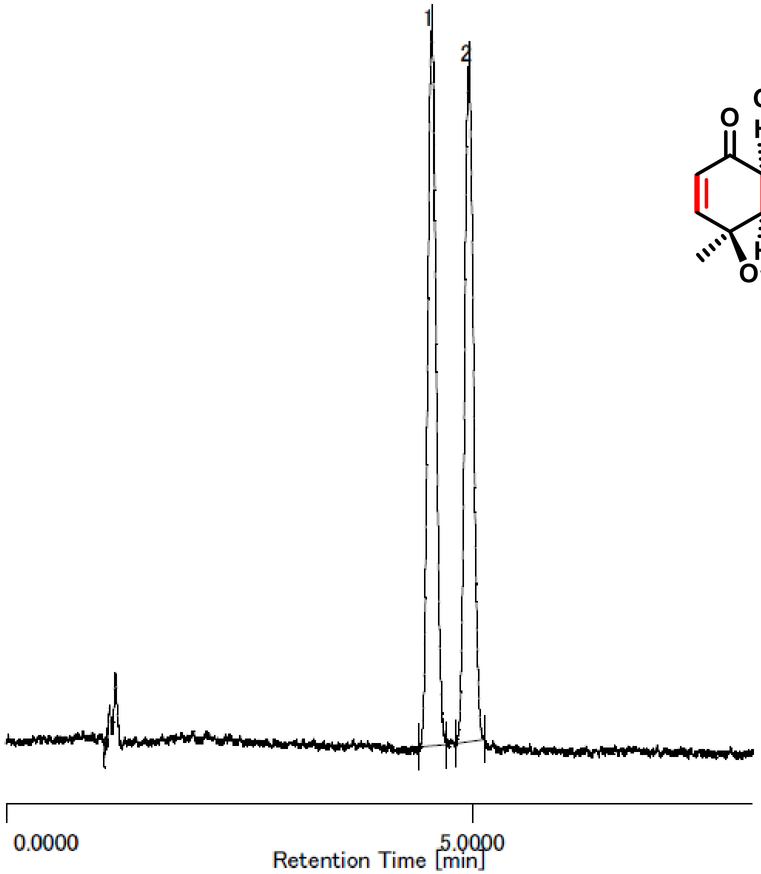

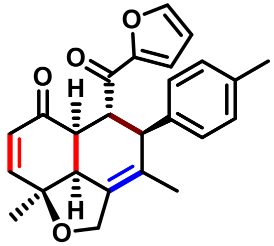

3ad

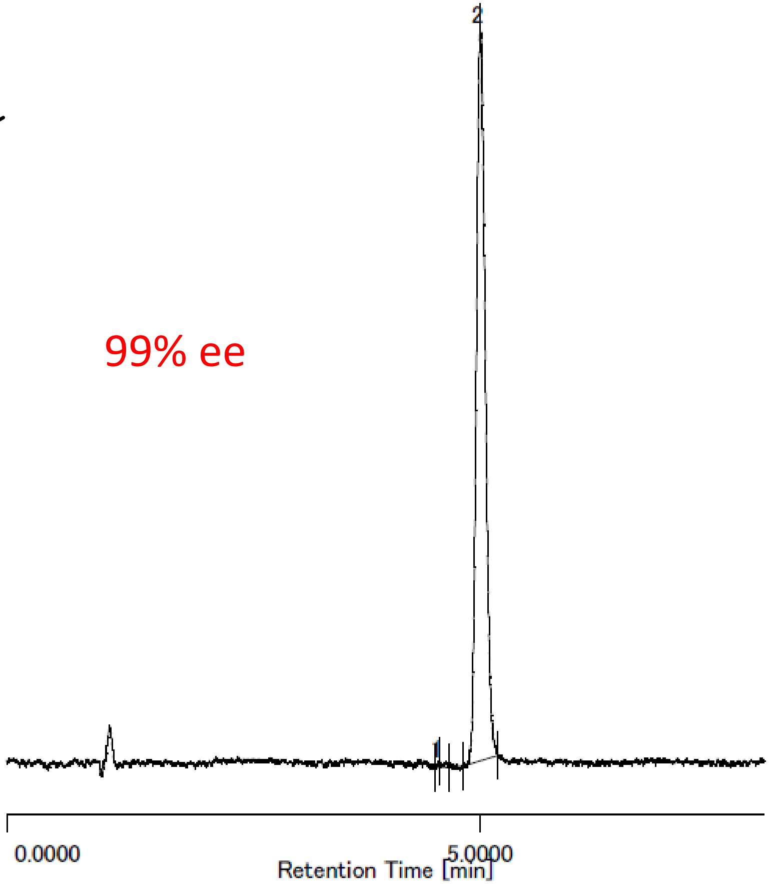

99% ee

ChromatogramName R-788-rac-model-dienone+furyl-chalcone

#	CH	tR	Area	Height	Area%
1	9	4.5633	583607	95296	50.462
2	9	4.9600	572925	90017	49.538

ChromatogramName R-789-chir-model-dienone+furyl-chalcone

#	CH	tR	Area	Height	Area%
1	9	4.5717	4751	1247	0.507
2	9	5.0000	932682	143738	99.493

Supplementary Figure 9. ^{1}H, ^{13}C-NMR and SFC spectra of product 3ae

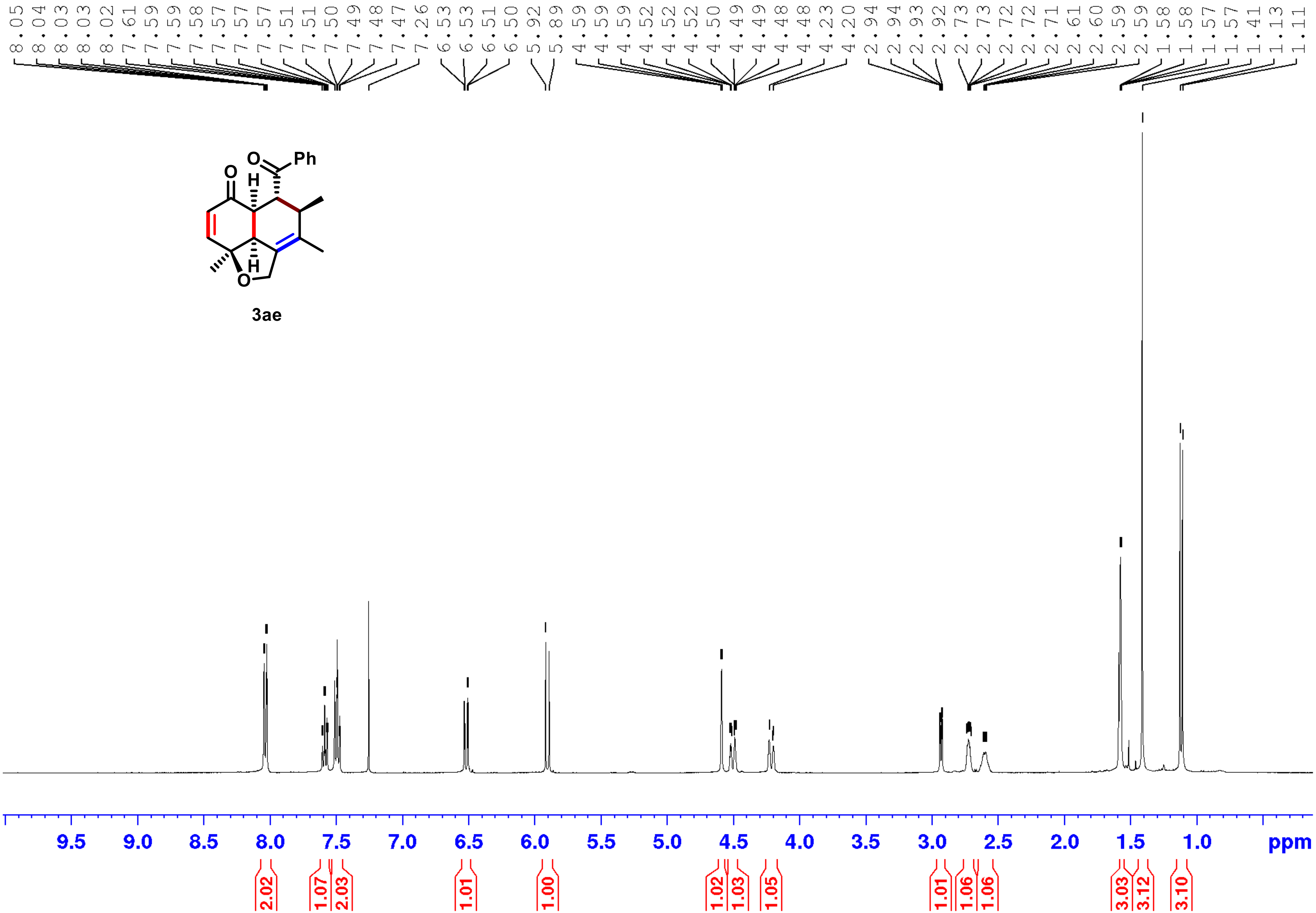

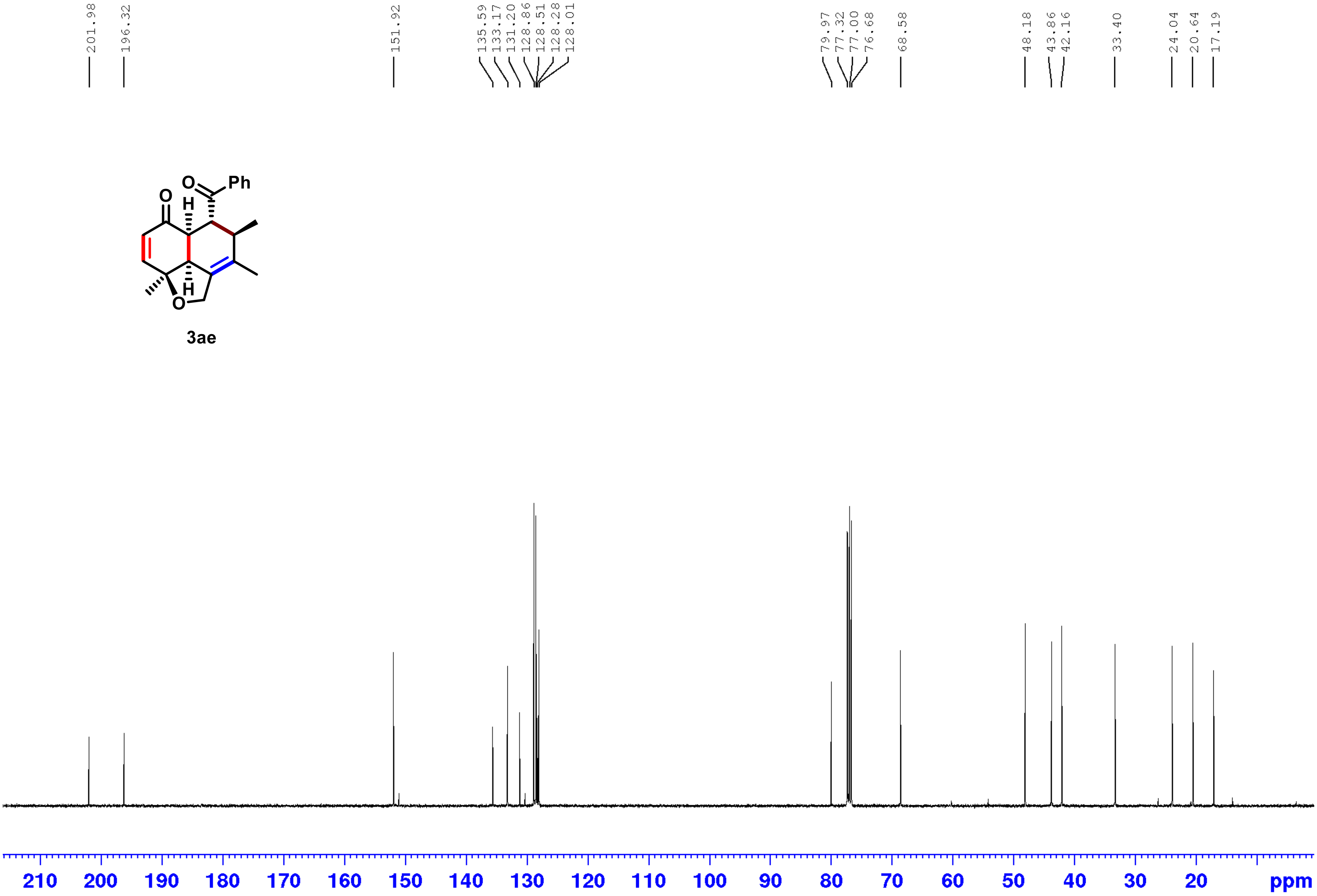
201.98
196.32
151.92
135.59
133.17
131.20
128.86
128.51
128.28
128.01
79.97
77.32
77.00
76.68
68.58
48.18
43.86
42.16
33.40
24.04
20.64
17.19
O
Ph
O
H
H
O
3ae
210
200
190
180
170
160
150
140
130
120
110
100
90
80
70
60
50
40
30
20
ppm

Chiral Separation (SFC): Chiralpak IA; Flow (CO_2) = 2.4 mL/min; Flow (isopropanol) = 0.6 mL/min; T = 25 ^oC; λ = 250 nm; Back pressure = 15 Mpa

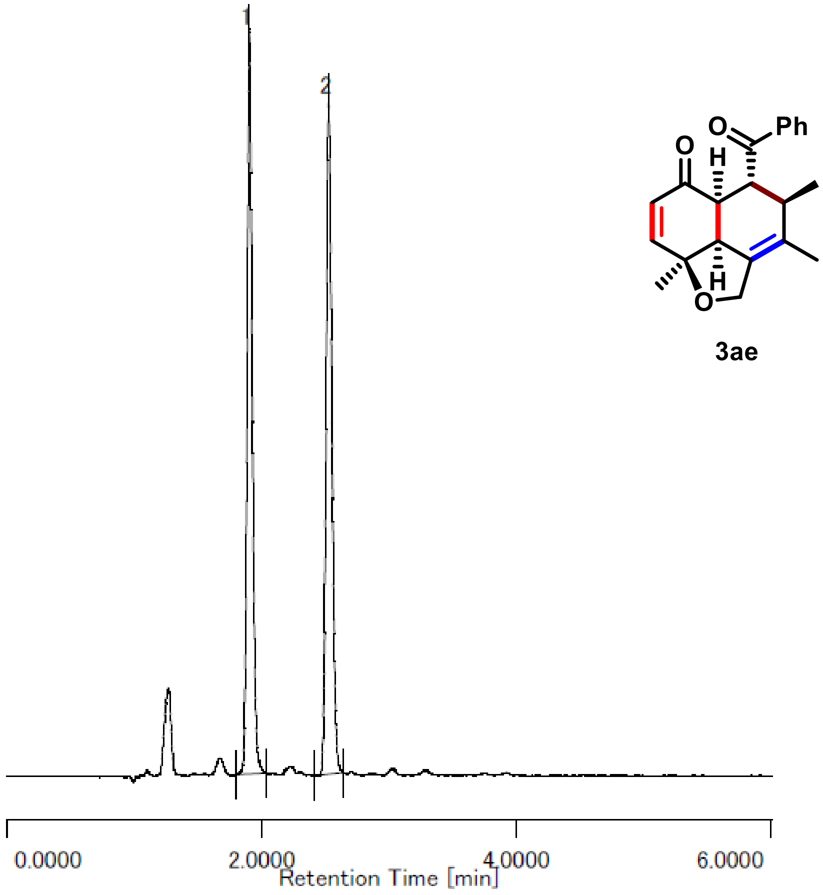

3ae

97% ee

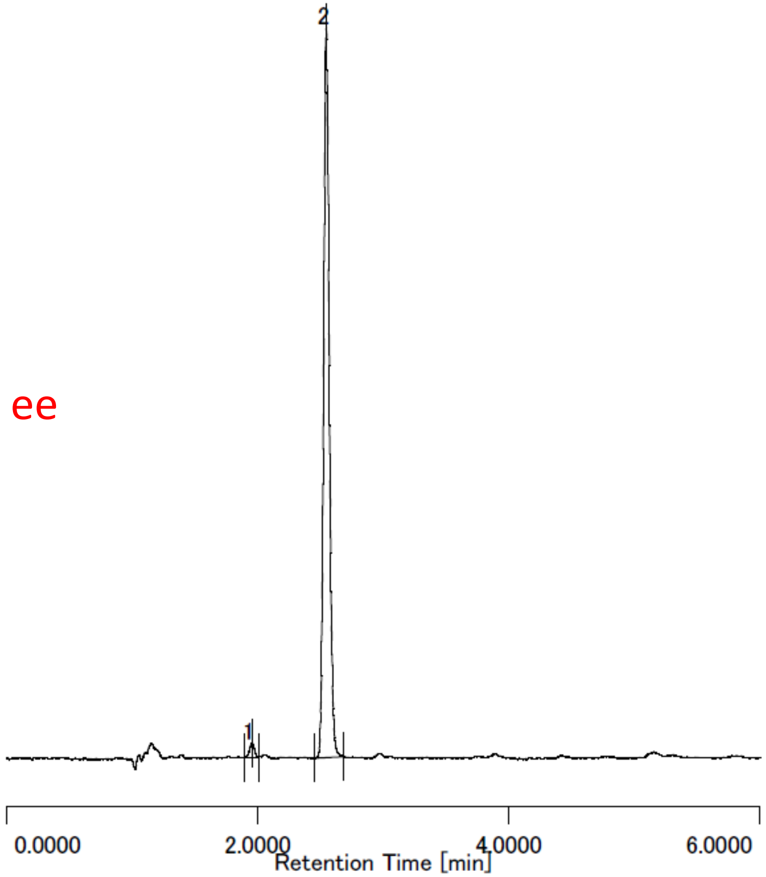

Chromatogram Name R–572–eri

#	CH	tR	Area	Height	Area%
1	9	1.9050	1858428	666890	49.443
2	9	2.5267	1900331	605206	50.557

Chromatogram Name R–637

#	CH	tR	Area	Height	Area%
1	9	1.9533	22445	8576	1.602
2	9	2.5450	1378261	444946	98.398

Supplementary Figure 10. ^{1}H and ^{13}C-NMR spectra of product 3ae'

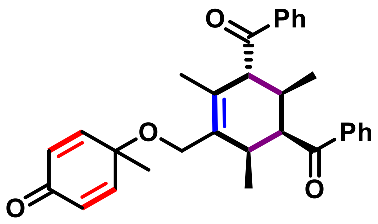

3ae'

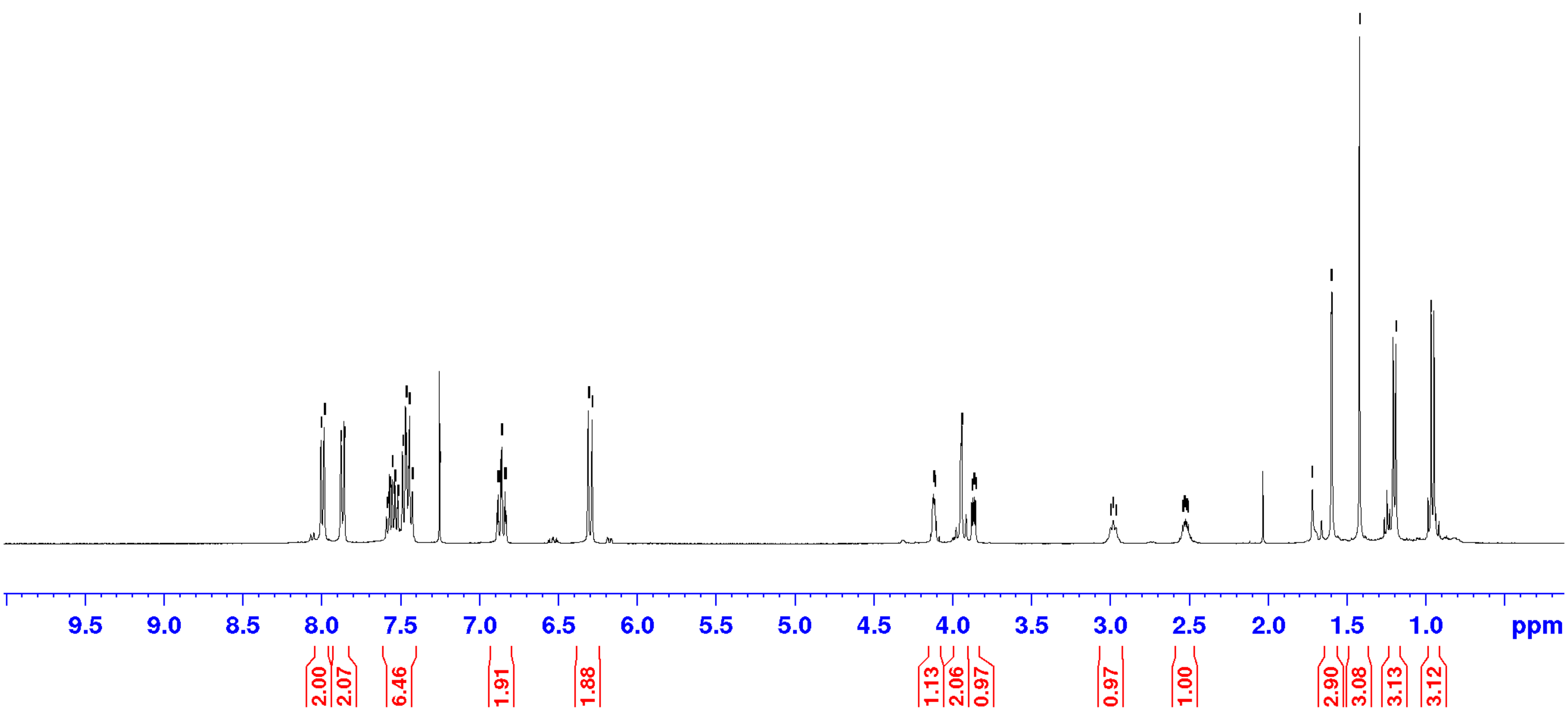

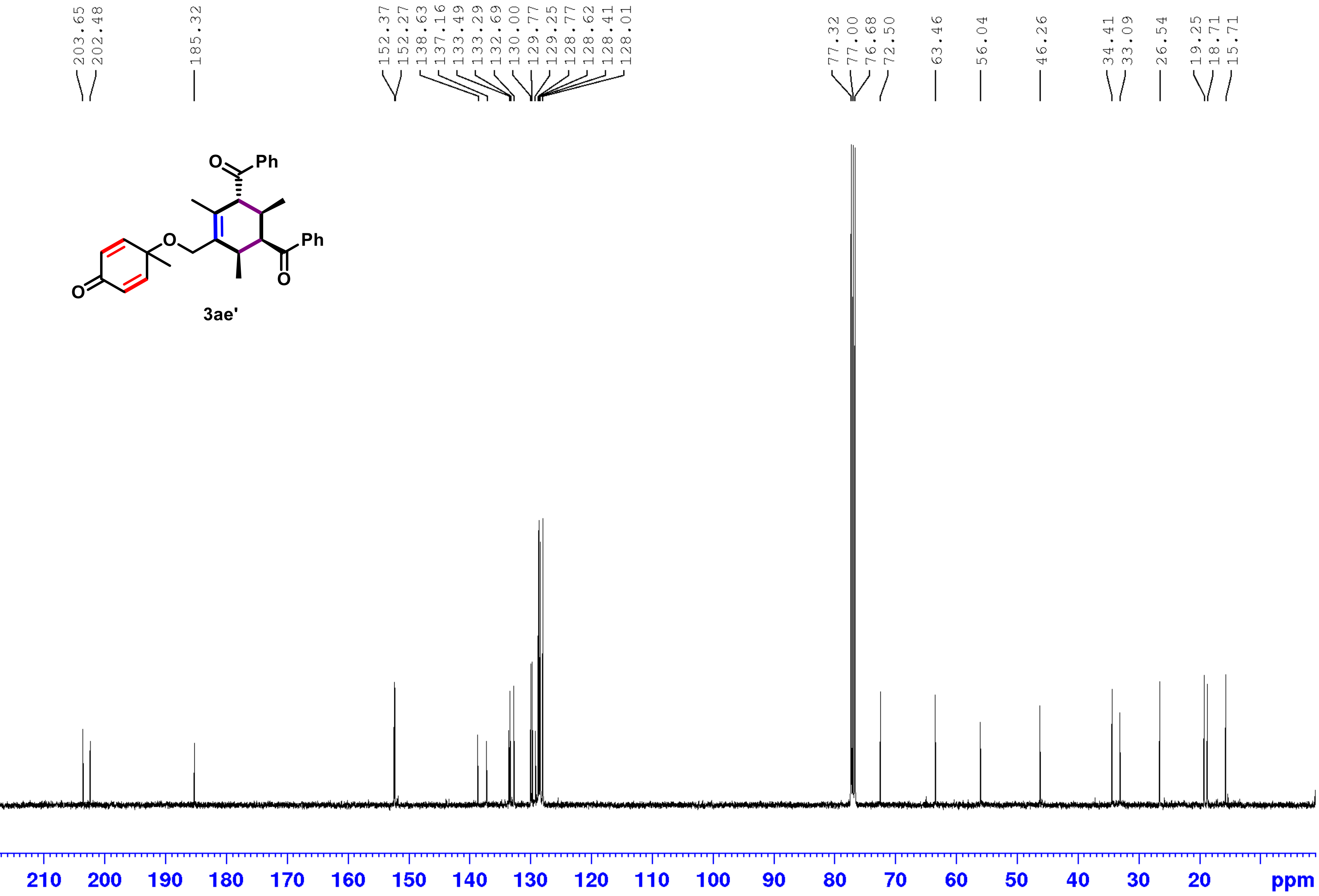

203.65
202.48
185.32
152.37
152.27
138.63
137.16
133.49
133.29
132.69
130.00
129.77
129.25
128.77
128.62
128.41
128.01
77.32
77.00
76.68
72.50
63.46
56.04
46.26
34.41
33.09
26.54
19.25
18.71
15.71
O
Ph
O
Ph
O
O
3ae'
210
200
190
180
170
160
150
140
130
120
110
100
90
80
70
60
50
40
30
20
ppm

Supplementary Figure 11. ^{1}H, ^{13}C-NMR and SFC spectra of product 3af

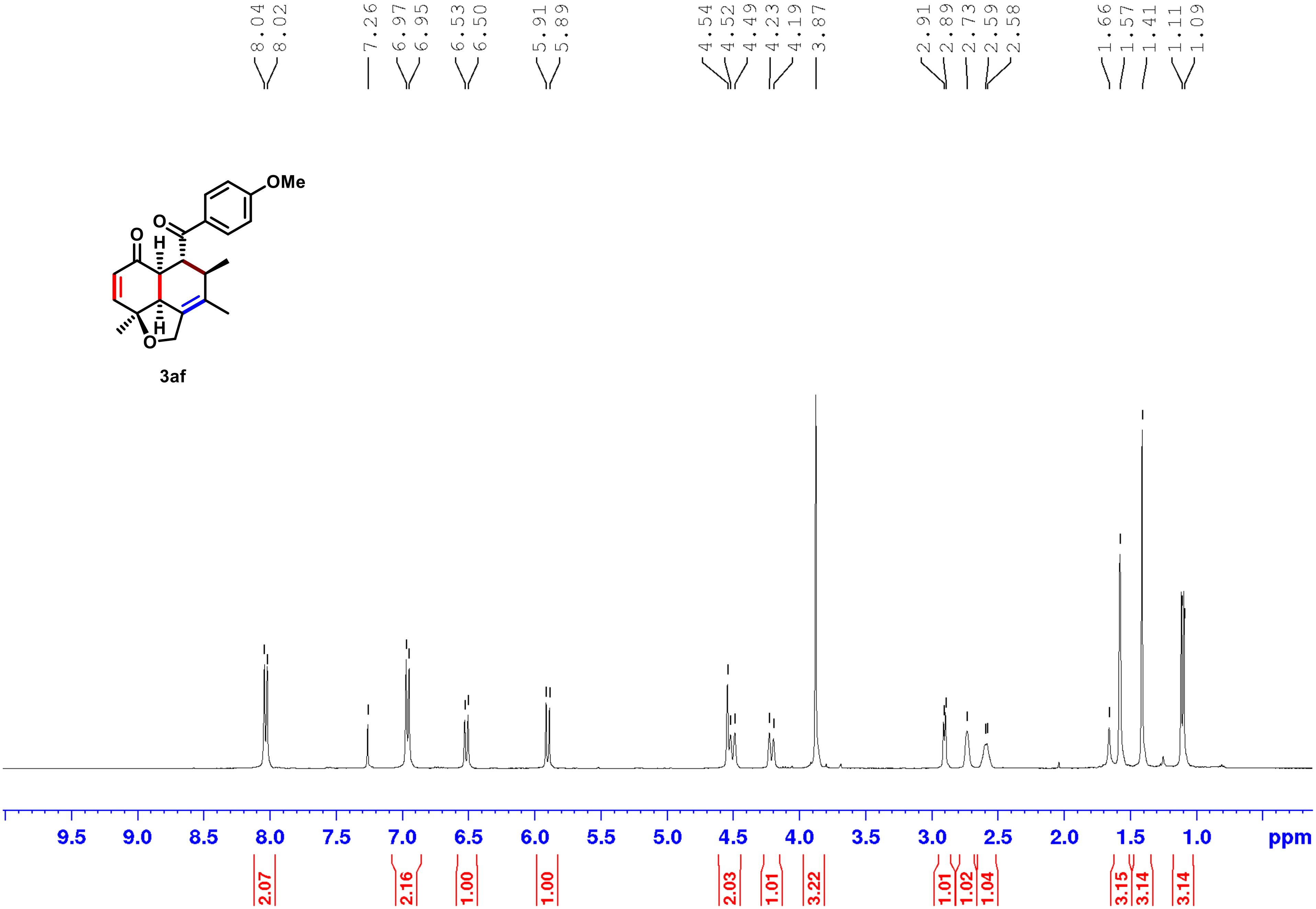

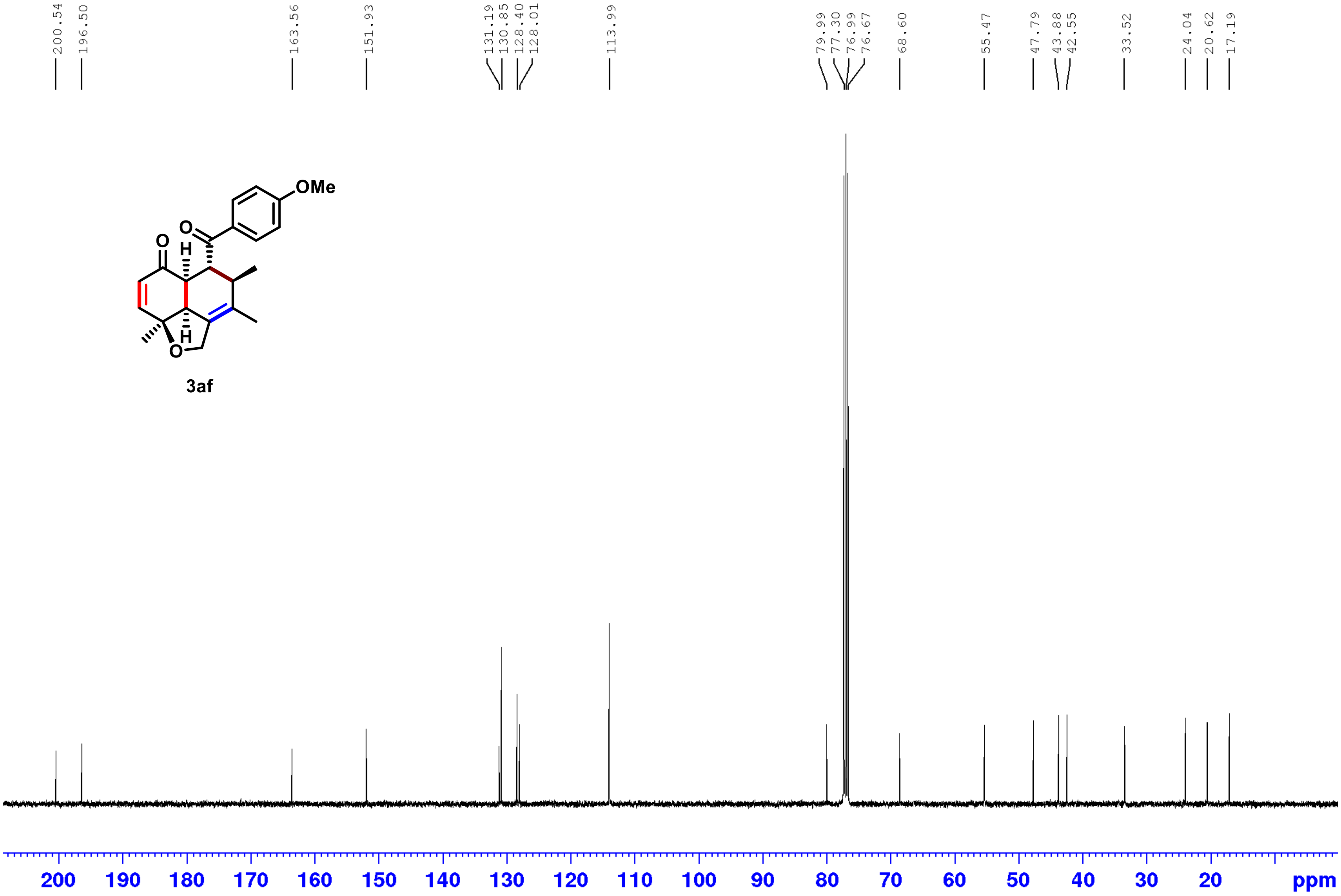
200.54
196.50
163.56
151.93
131.19
130.85
128.40
128.01
113.99
79.99
77.30
76.99
76.67
68.60
55.47
47.79
43.88
42.55
33.52
24.04
20.62
17.19
OMe
O
O
H
H
O
3af
200
190
180
170
160
150
140
130
120
110
100
90
80
70
60
50
40
30
20
ppm

Chiral Separation (SFC): Chiralpak IA; Flow (CO_2) = 2.4 mL/min; Flow (isopropanol) = 0.6 mL/min; T = 25 ºC; λ = 250 nm; Back pressure = 15 Mpa

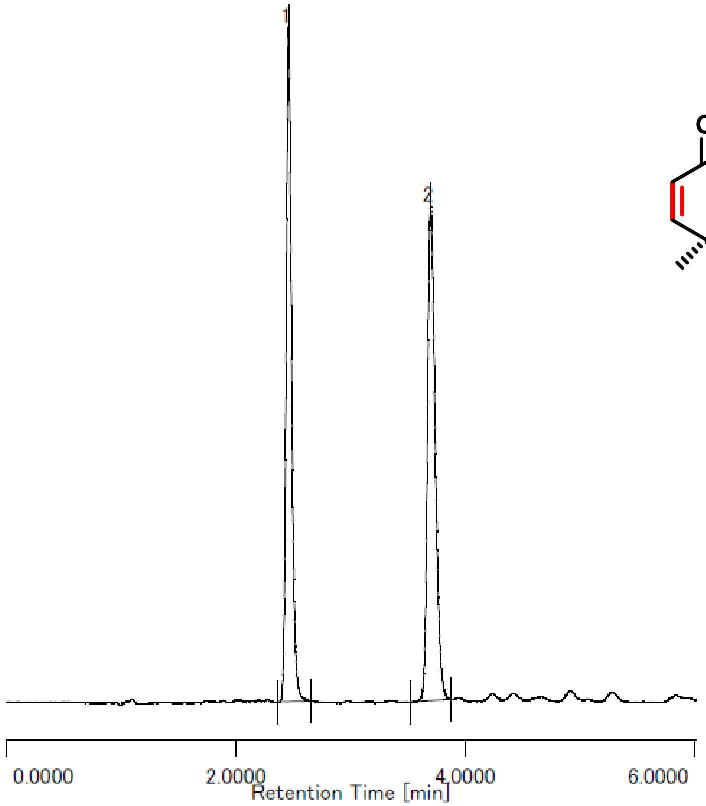

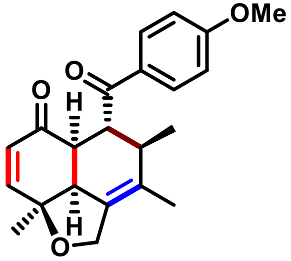

3af

94% ee

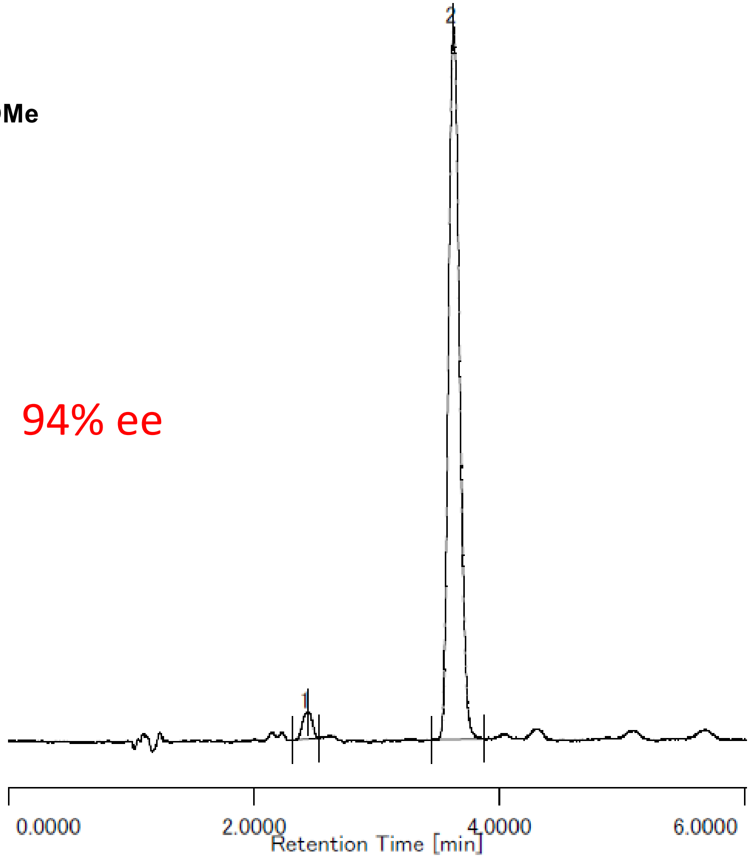

Chromatogram Name E-565

#	CH	tR	Area	Height	Area%
1	11	2.4583	2639959	806009	48.986
2	11	3.6933	2749229	591828	51.014

Chromatogram Name R-654-p-OMe-PPK

#	CH	tR	Area	Height	Area%
1	11	2.4383	66360	12213	3.217
2	11	3.6217	1996317	317255	96.783

Supplementary Figure 12. ^{1}H, ^{13}C-NMR and SFC spectra of product 3ag

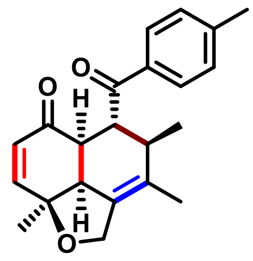

3ag

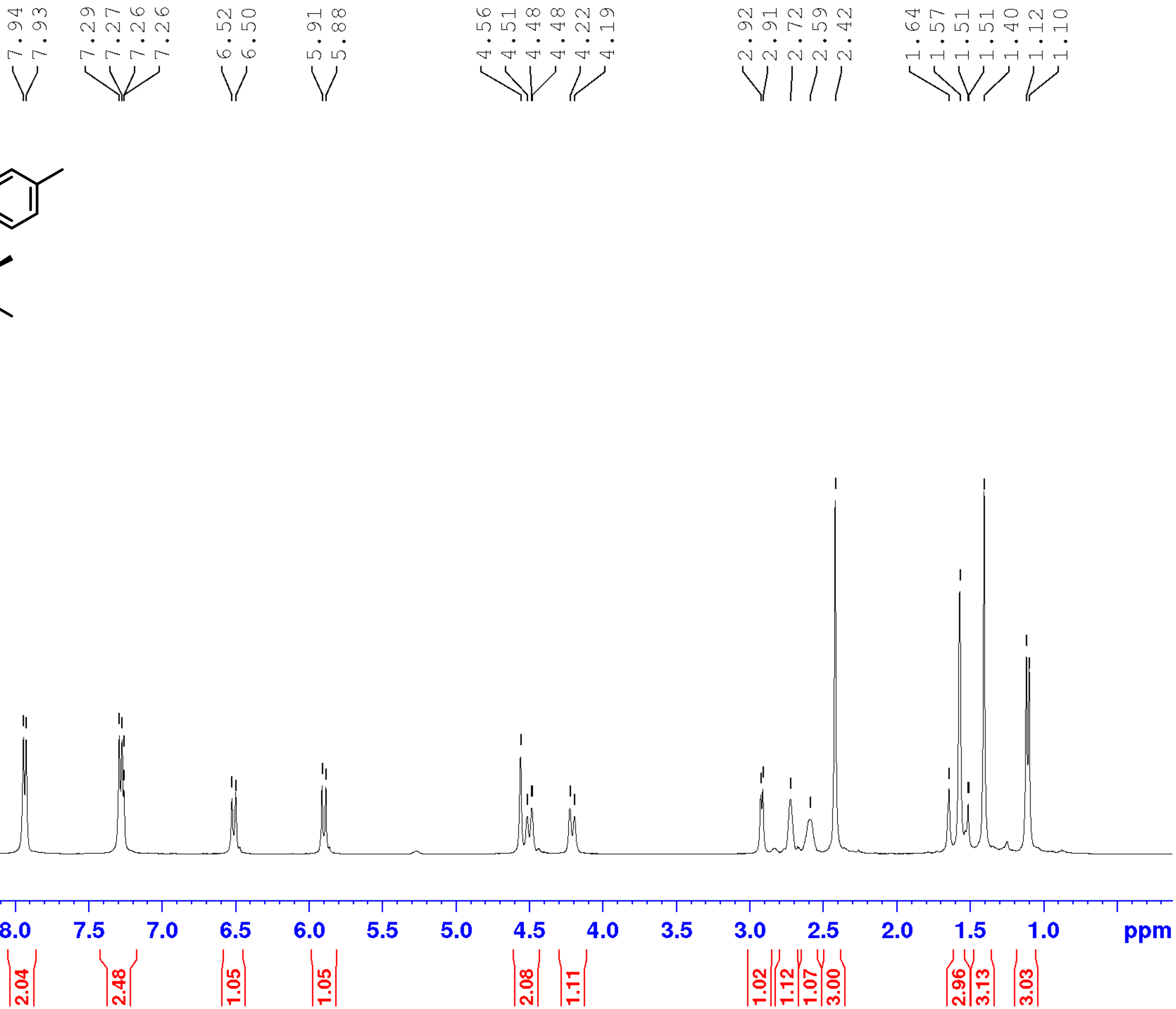

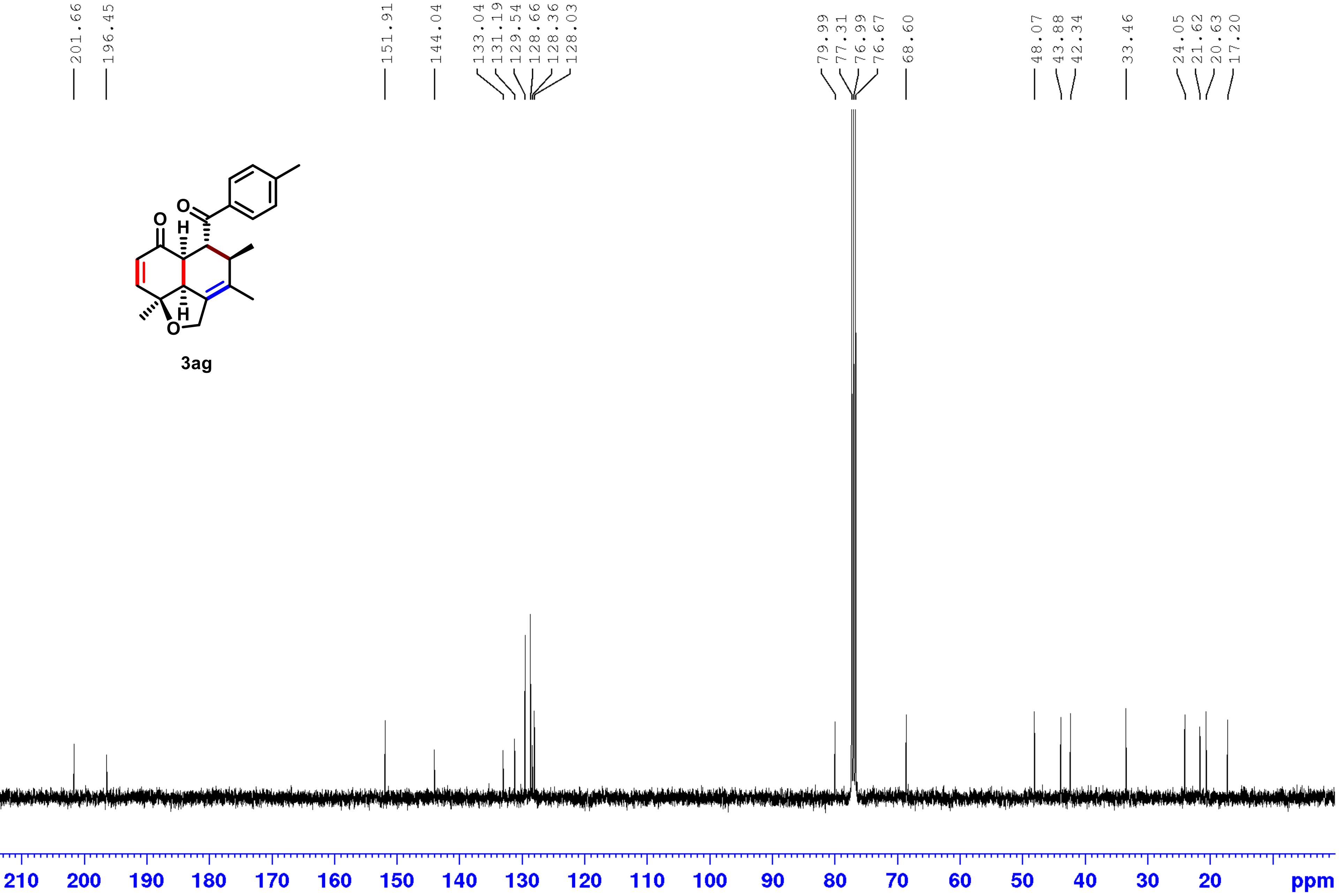
201.66
196.45
151.91
144.04
133.04
131.19
129.54
128.66
128.36
128.03
79.99
77.31
76.99
76.67
68.60
48.07
43.88
42.34
33.46
24.05
21.62
20.63
17.20
3ag
210 200 190 180 170 160 150 140 130 120 110 100 90 80 70 60 50 40 30 20 ppm

Chiral Separation (SFC): Chiralpak IA; Flow (CO_2) = 3.0 mL/min; Flow (isopropanol) = 0.3 mL/min; T = 25 ^{o}C; λ = 250 nm; Back pressure = 15 Mpa

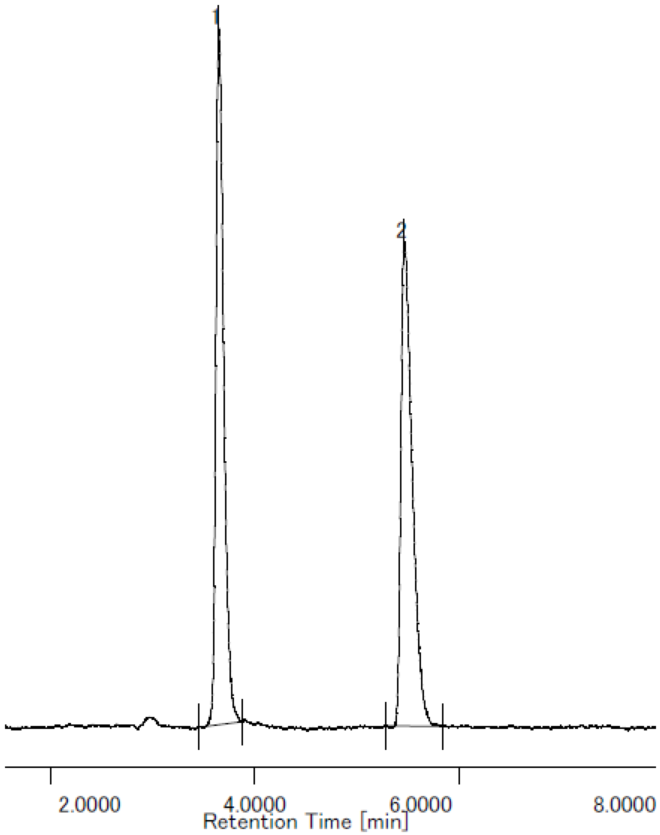

Chromatogram Name E-654-p-Me-PPK (R)

#	CH	tR	Area	Height	Area%
1	10	3.6467	1918765	362037	49.748
2	10	5.4617	1938168	251536	50.252

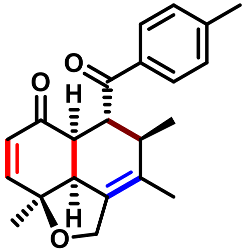

3ag

95% ee

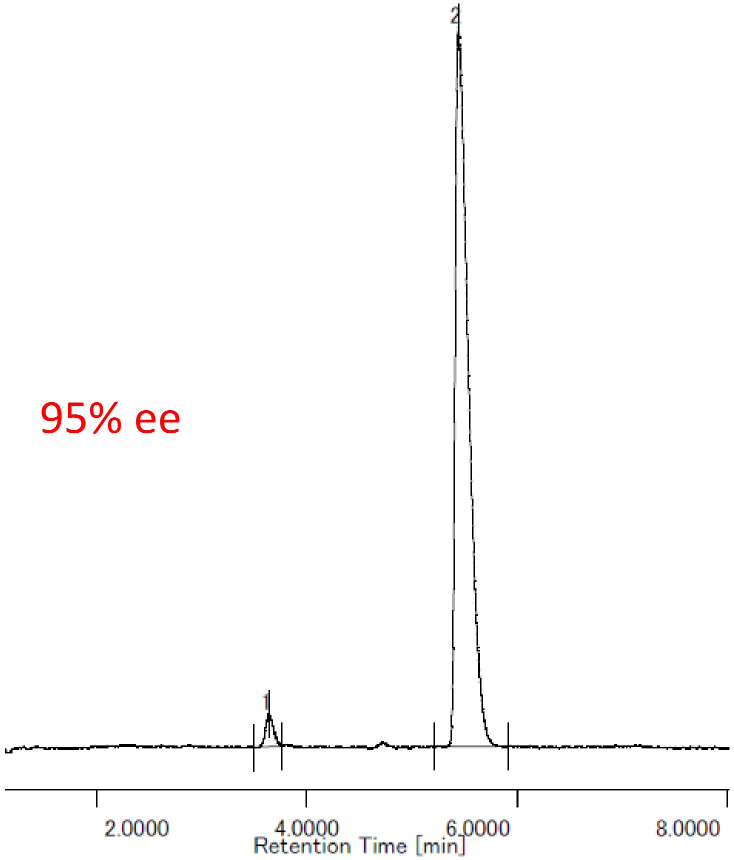

Chromatogram Name R-676-p-Me-PPK(C)

#	CH	tR	Area	Height	Area%
1	10	3.6383	112471	21713	2.699
2	10	5.4367	4054507	472044	97.301

Supplementary Figure 13. ^{1}H, ^{13}C-NMR and SFC spectra of product 3ah

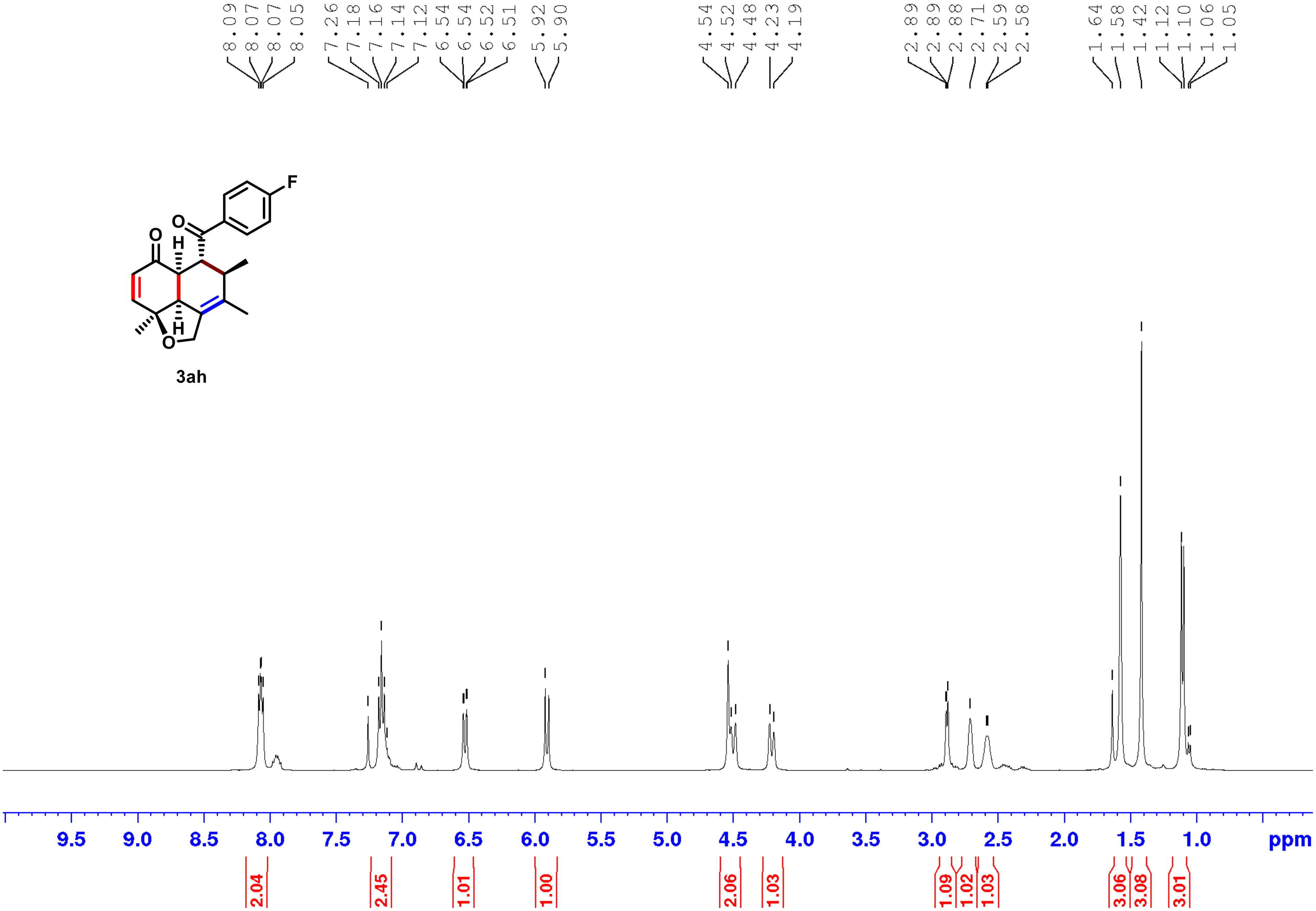

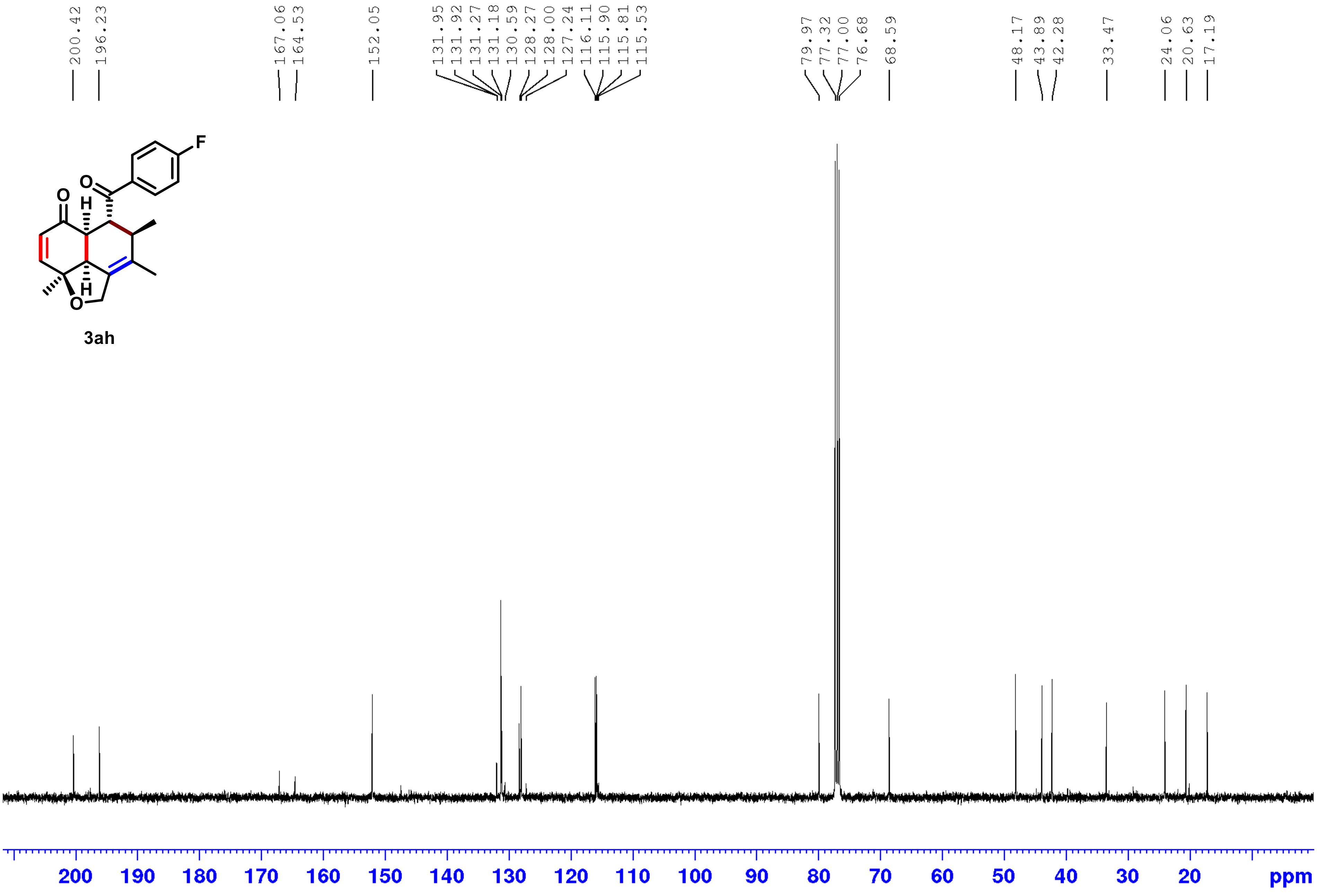

200.42
196.23
167.06
164.53
152.05
131.95
131.92
131.27
131.18
130.59
128.27
128.00
127.24
116.11
115.90
115.81
115.53
79.97
77.32
77.00
76.68
68.59
48.17
43.89
42.28
33.47
24.06
20.63
17.19
3ah
200
190
180
170
160
150
140
130
120
110
100
90
80
70
60
50
40
30
20
ppm

Chiral Separation (SFC): Chiralpak IA; Flow (CO_2) = 3.0 mL/min; Flow (isopropanol) = 0.3 mL/min; T = 25 ^{o}C; λ = 250 nm; Back pressure = 15 Mpa

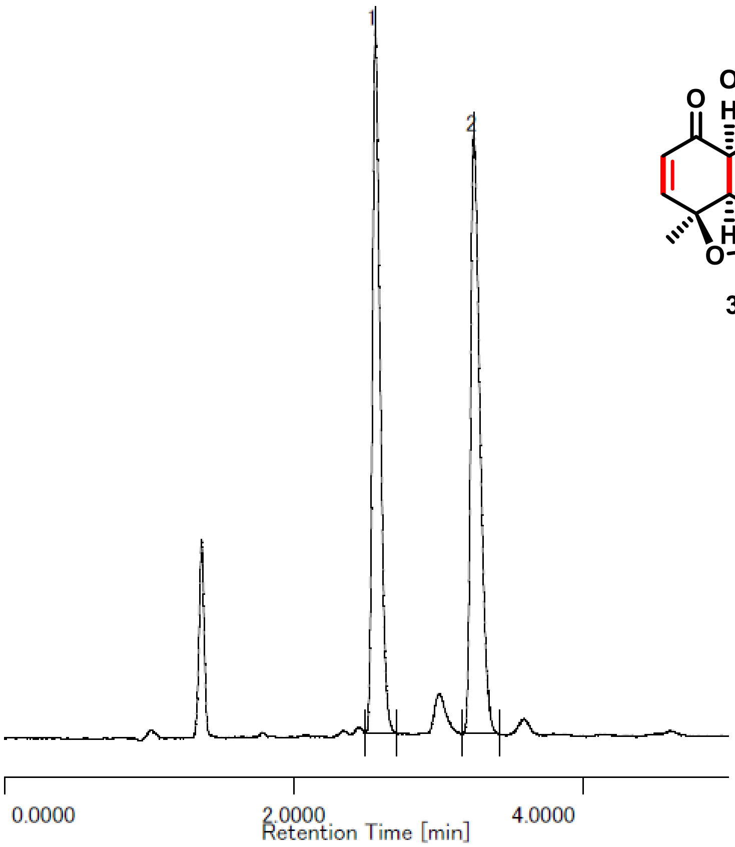

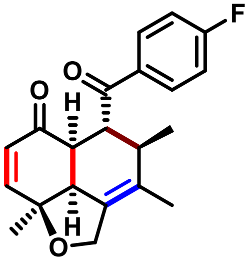

3ah

95% ee

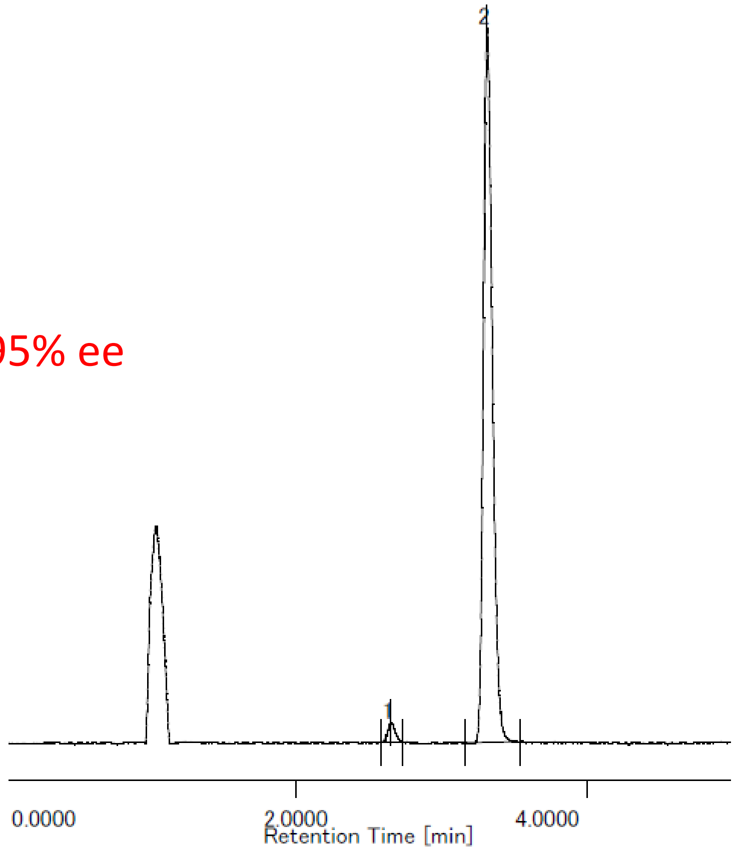

Chromatogram Name E−566

#	CH	tR	Area	Height	Area%
1	10	2.5650	2979865	805647	49.010
2	10	3.2433	3100264	685843	50.990

Chromatogram Name R−655−p−F−PPK

#	CH	tR	Area	Height	Area%
1	10	2.6533	107134	30734	2.281
2	10	3.3117	4589541	1100645	97.719

Supplementary Figure 14. ¹H, ¹³C-NMR and SFC spectra of product 3ai

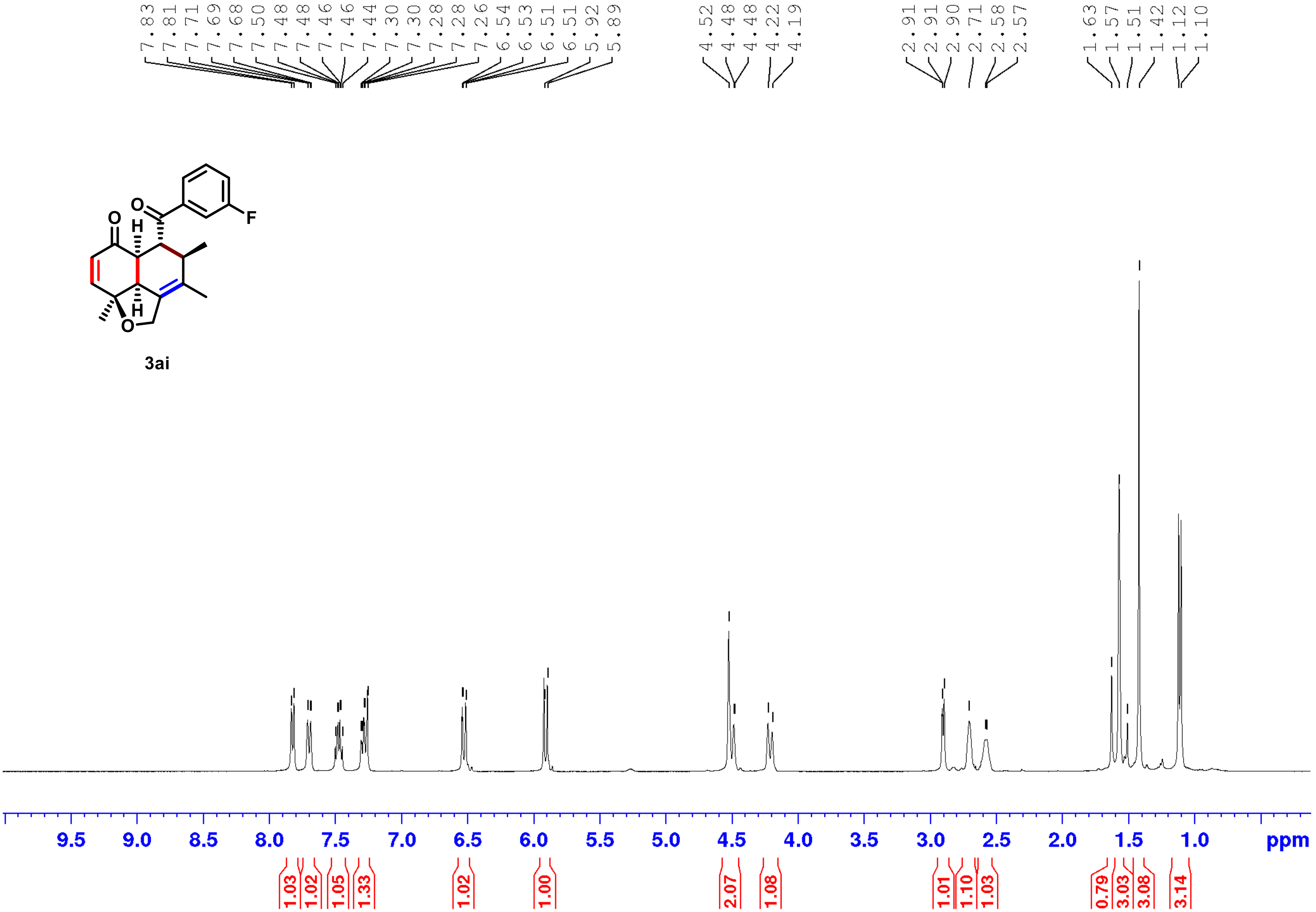

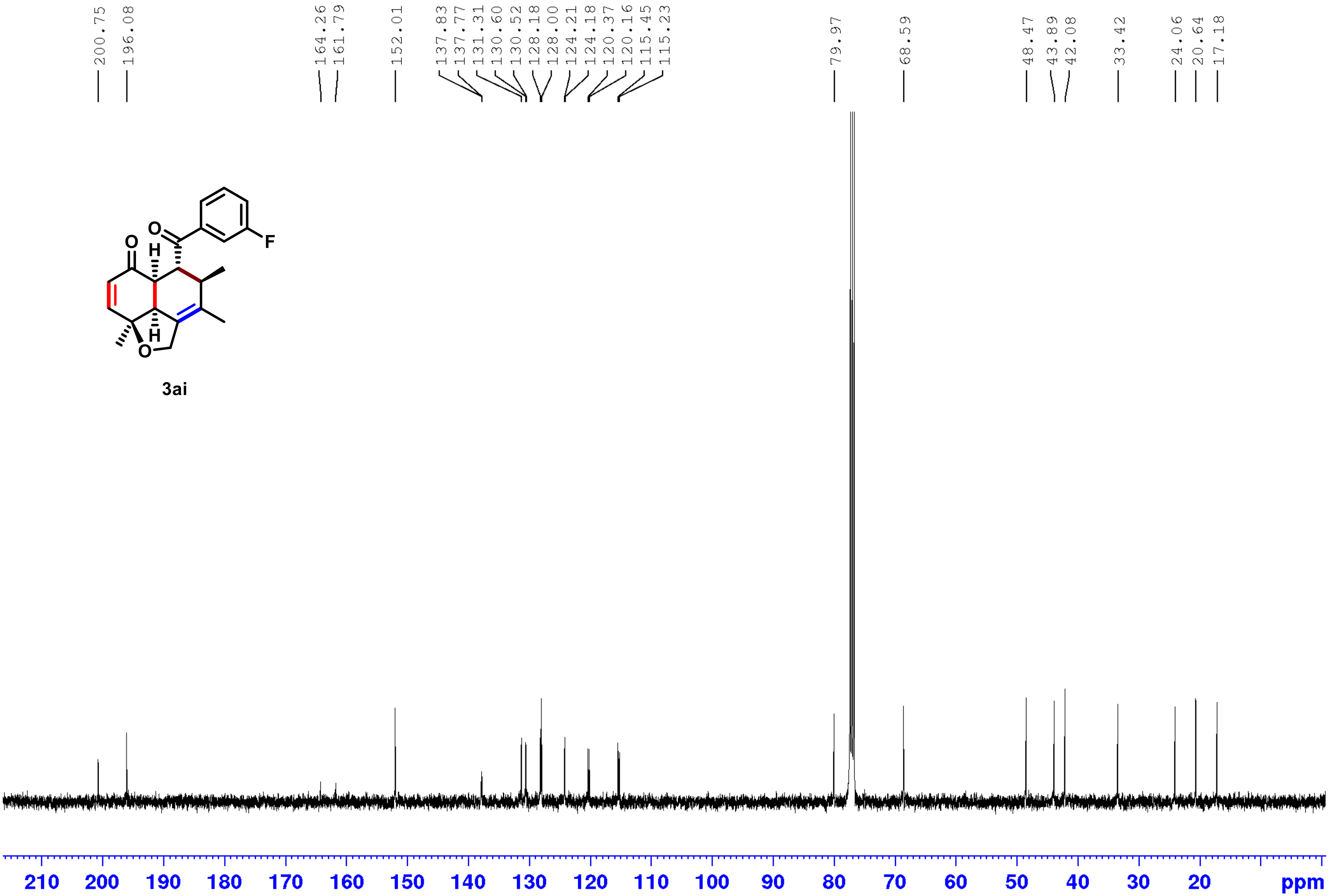
200.75
196.08
164.26
161.79
152.01
137.83
137.77
131.31
130.60
130.52
128.18
128.00
124.21
124.18
120.37
120.16
115.45
115.23
79.97
68.59
48.47
43.89
42.08
33.42
24.06
20.64
17.18
O
O
H
H
O
F
3ai
210 200 190 180 170 160 150 140 130 120 110 100 90 80 70 60 50 40 30 20 ppm

Chiral Separation (SFC): Chiralpak IA; Flow (CO_2) = 4.5 mL/min; Flow (isopropanol) = 0.2 mL/min; T = 25 ^oC; λ = 250 nm; Back pressure = 15 Mpa

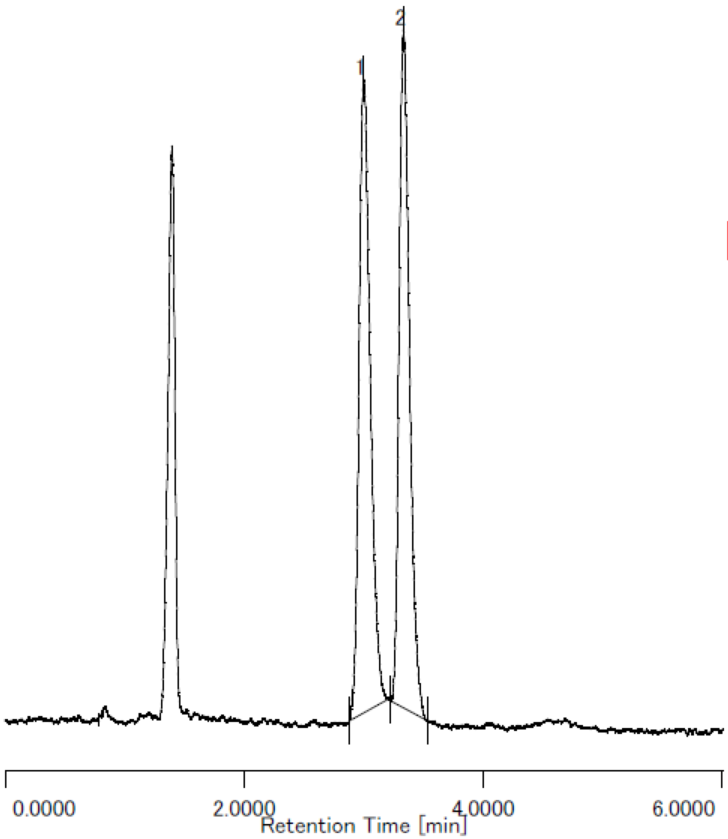

Chromatogram Name E−567

#	CH	tR	Area	Height	Area%
1	10	2.9933	653527	99269	50.448
2	10	3.3267	641924	106257	49.552

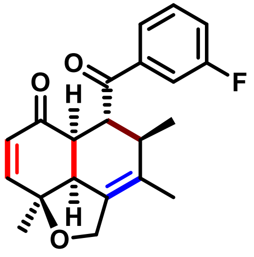

3ai

97% ee

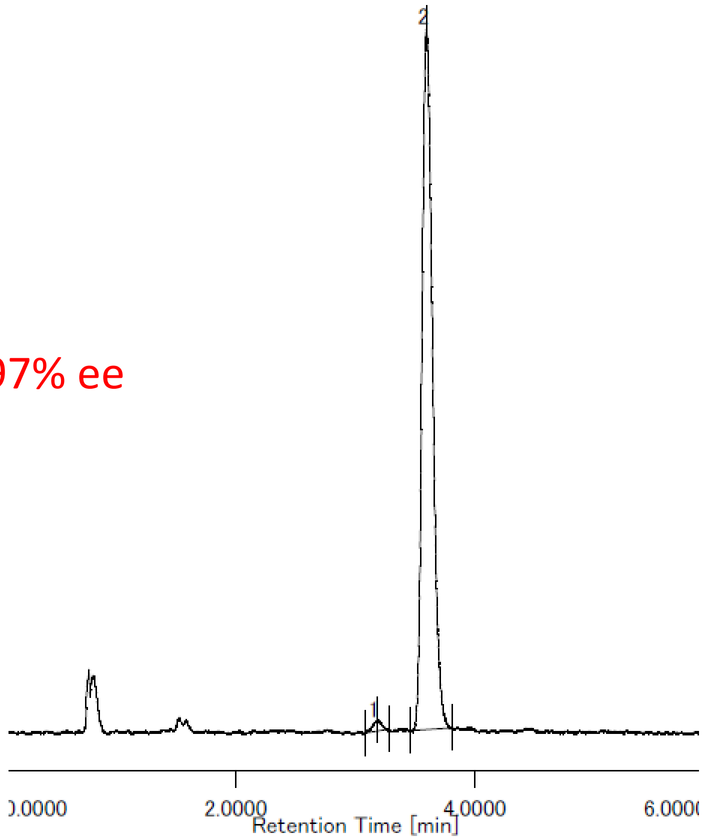

Chromatogram Name R−656−m−F−PPK

#	CH	tR	Area	Height	Area%
1	10	3.1733	23472	4668	1.305
2	10	3.5917	1775710	296078	98.695

Supplementary Figure 15. ^{1}H, ^{13}C-NMR and SFC spectra of product 3bb

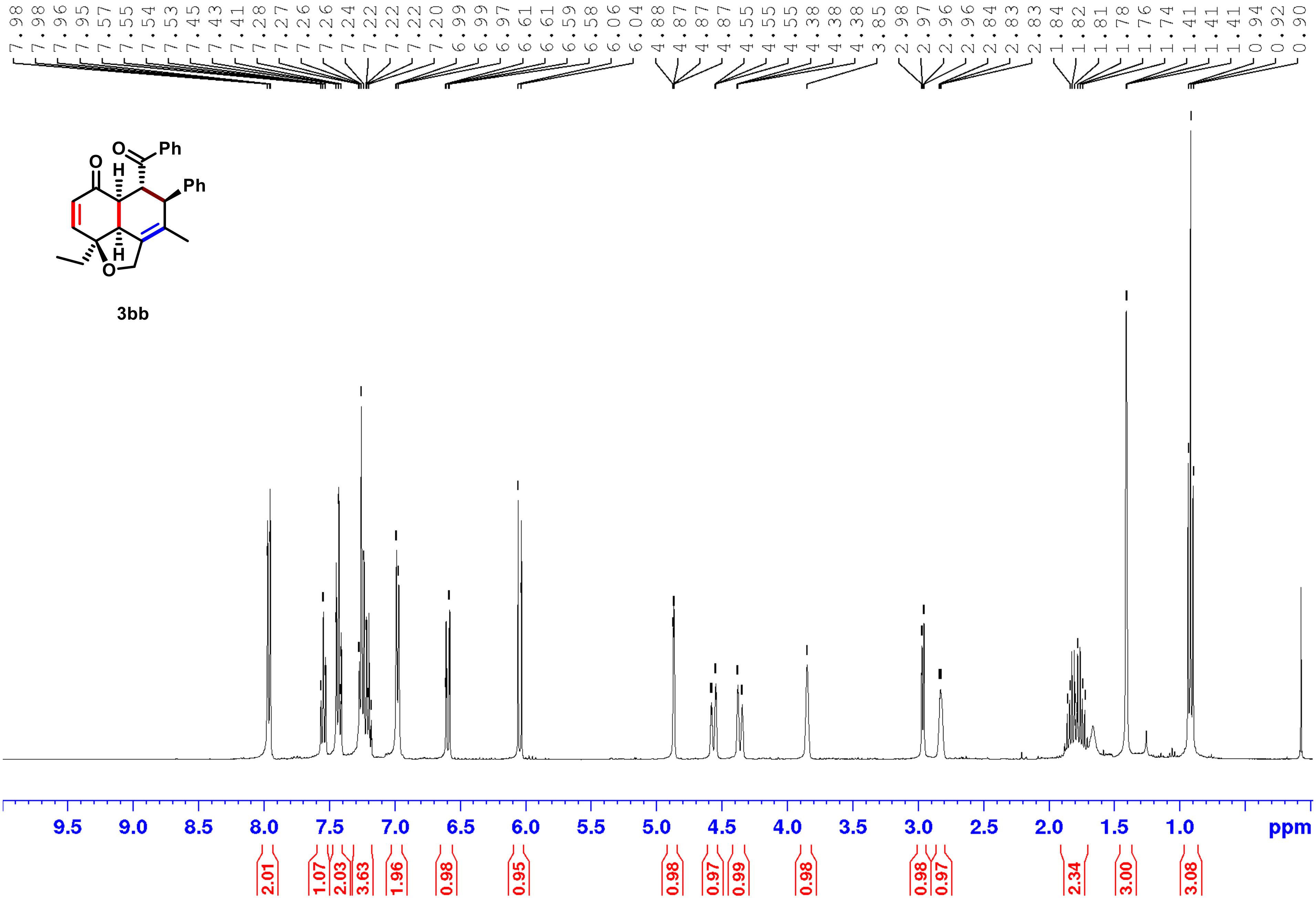

3bb

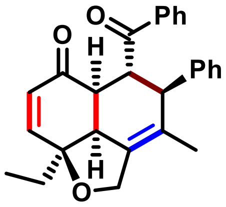

O
Ph
O
H
Ph
H
O

3bb

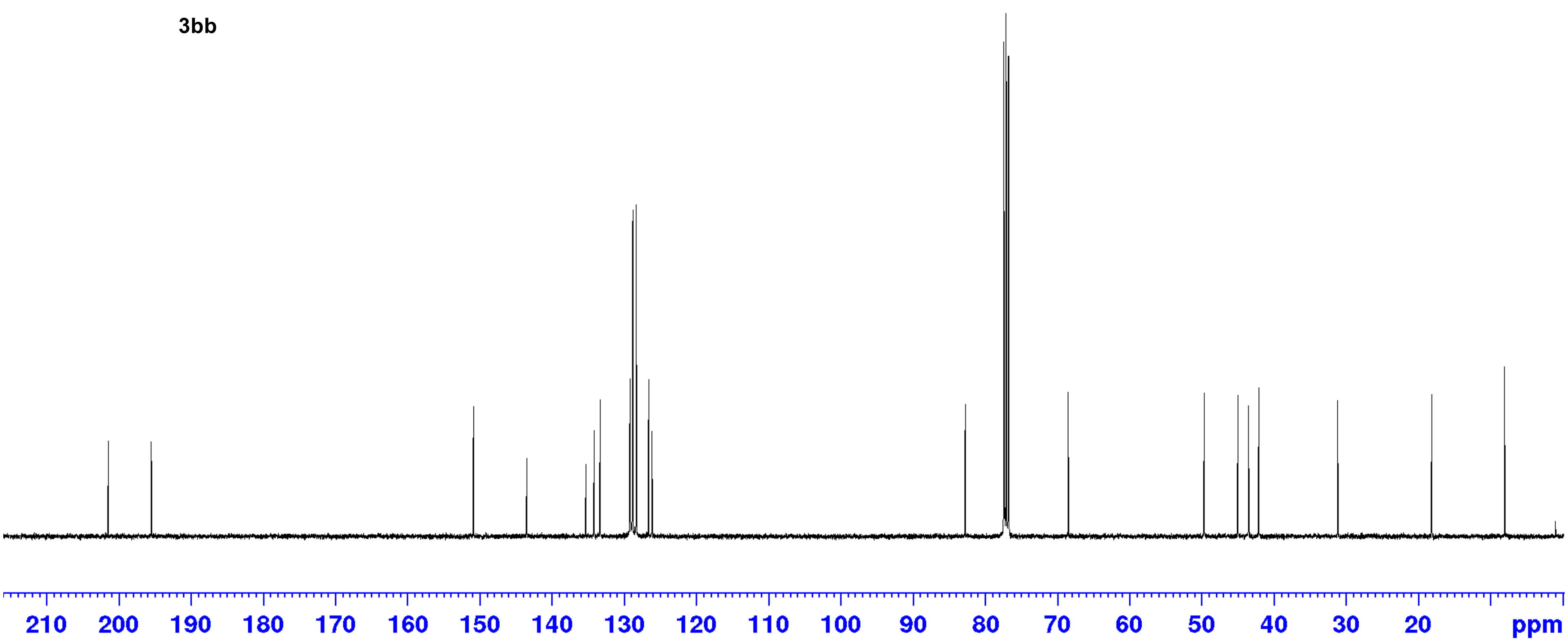

201.53
195.55
150.94
143.42
135.25
134.10
133.27
129.15
129.10
128.80
128.74
128.27
126.56
126.07
82.69
77.31
77.19
76.99
76.67
68.40
49.57
44.89
43.42
42.01
31.06
18.14
8.03
210 200 190 180 170 160 150 140 130 120 110 100 90 80 70 60 50 40 30 20 ppm

Chiral Separation (SFC): Chiralpak IA; Flow (CO_2) = 3.0 mL/min; Flow (isopropanol) = 0.3 mL/min; T = 25 ºC; λ = 250 nm; Back pressure = 15 Mpa

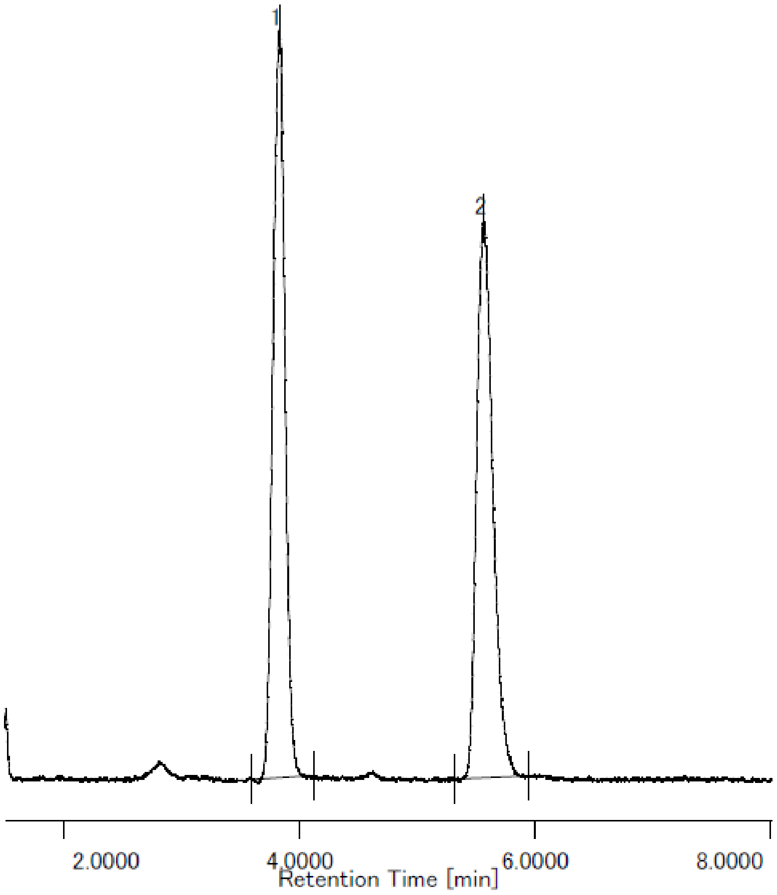

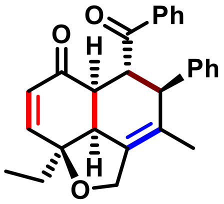

3bb

99% ee

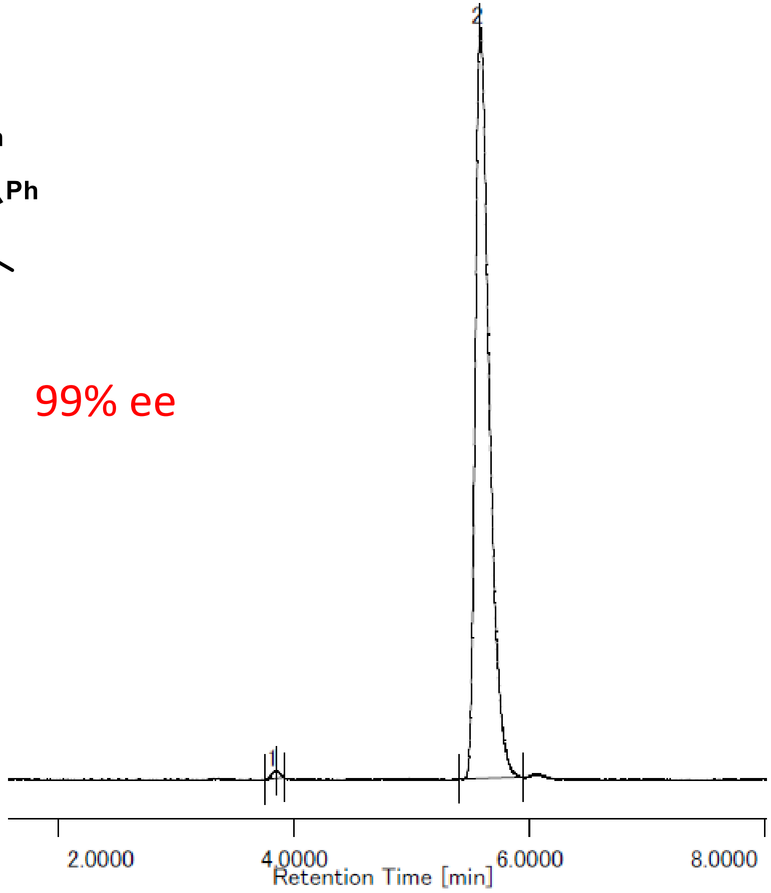

Chromatogram Name R-695

#	CH	tR	Area	Height	Area%
1	11	3.8250	843034	119024	49.959
2	11	5.5550	844424	89022	50.041

Chromatogram Name R-699

#	CH	tR	Area	Height	Area%
1	11	3.8417	22987	5210	0.551
2	11	5.5750	4149429	498958	99.449

Supplementary Figure 16. ^{1}H, ^{13}C-NMR and SFC spectra of product 3bj

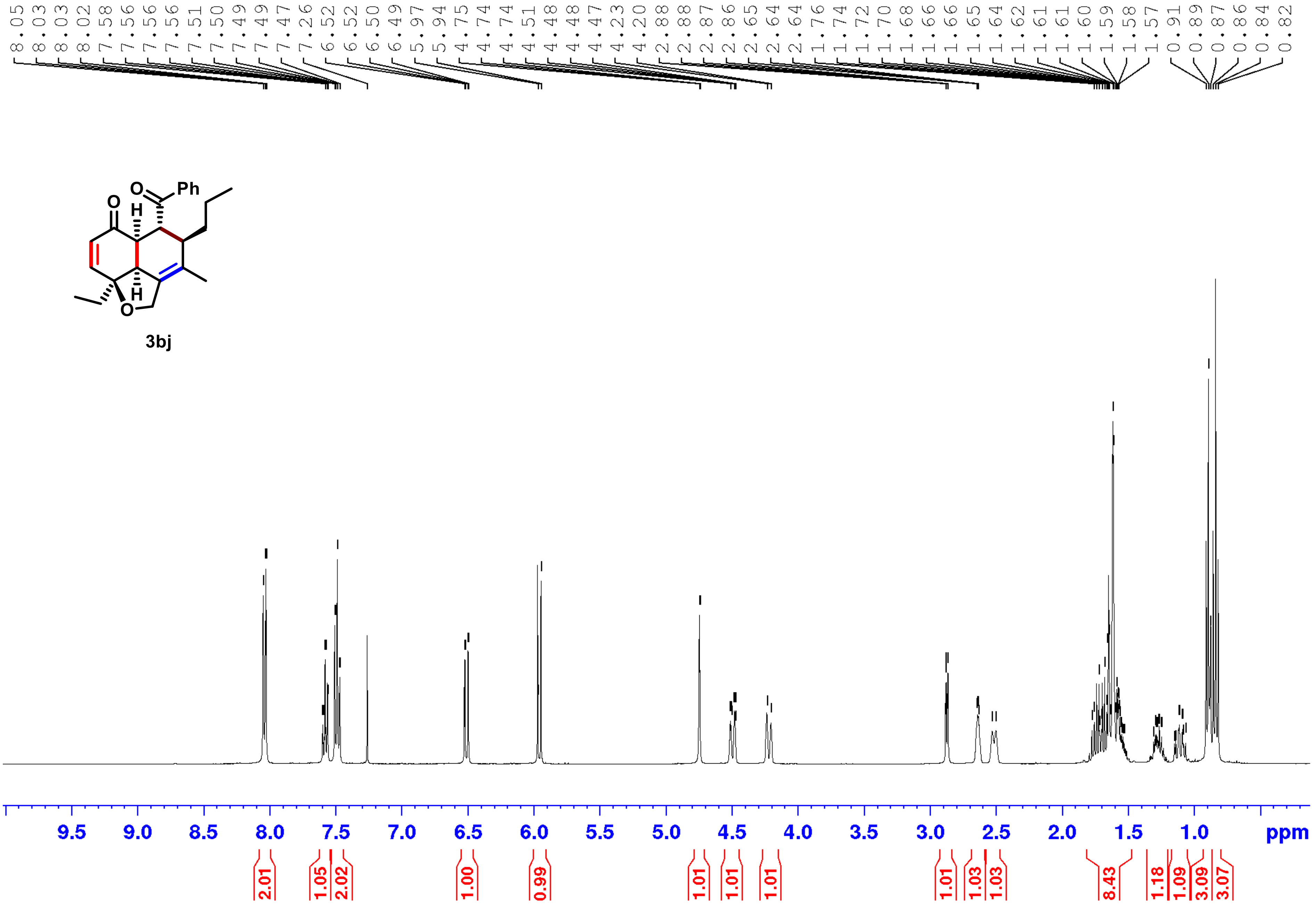

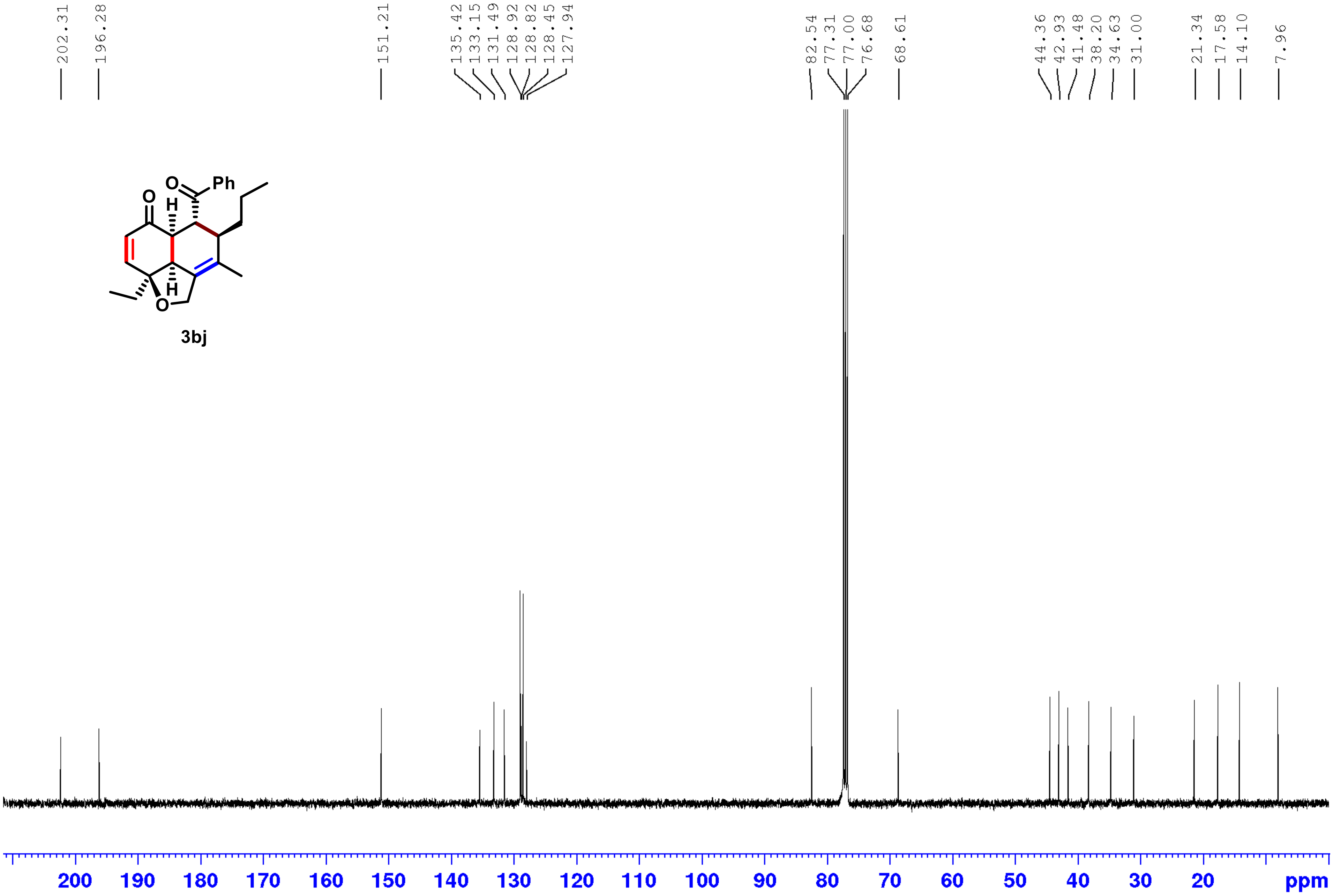

202.31
196.28
151.21
135.42
133.15
131.49
128.92
128.82
128.45
127.94
82.54
77.31
77.00
76.68
68.61
44.36
42.93
41.48
38.20
34.63
31.00
21.34
17.58
14.10
7.96
O
Ph
O
H
H
O
3bj
200
190
180
170
160
150
140
130
120
110
100
90
80
70
60
50
40
30
20
ppm

Chiral Separation (SFC): Chiralpak IA; Flow (CO_2) = 3.0 mL/min; Flow (isopropanol) = 0.3 mL/min; T = 25 ^oC; λ = 250 nm; Back pressure = 15 Mpa

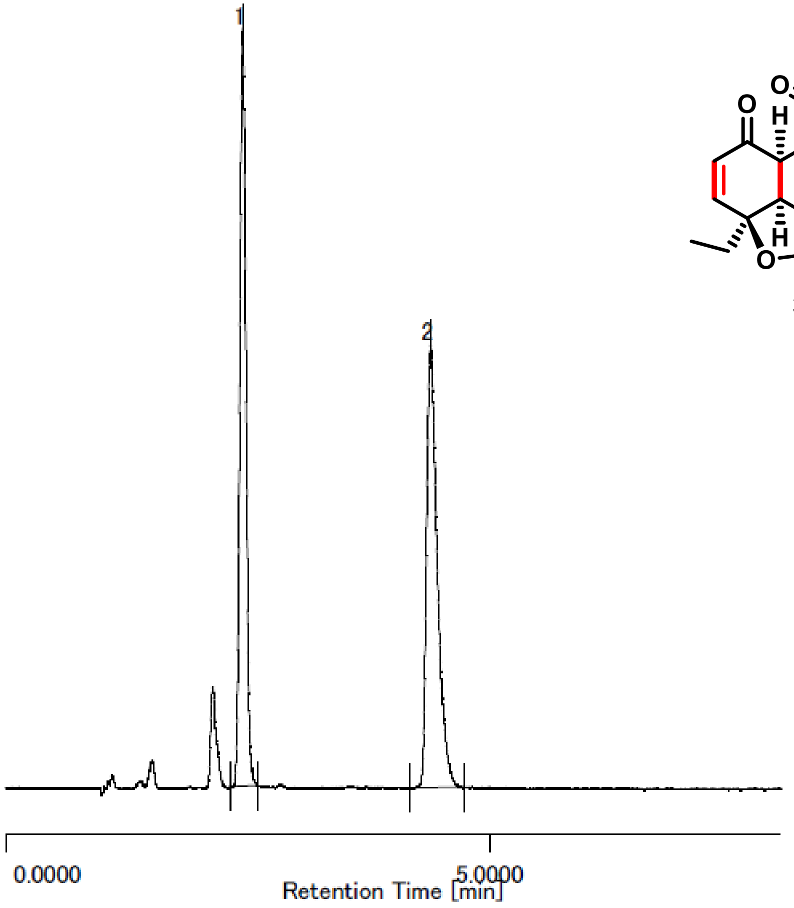

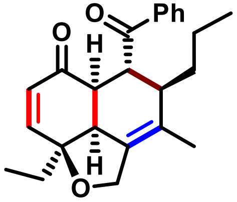

3bj

96% ee

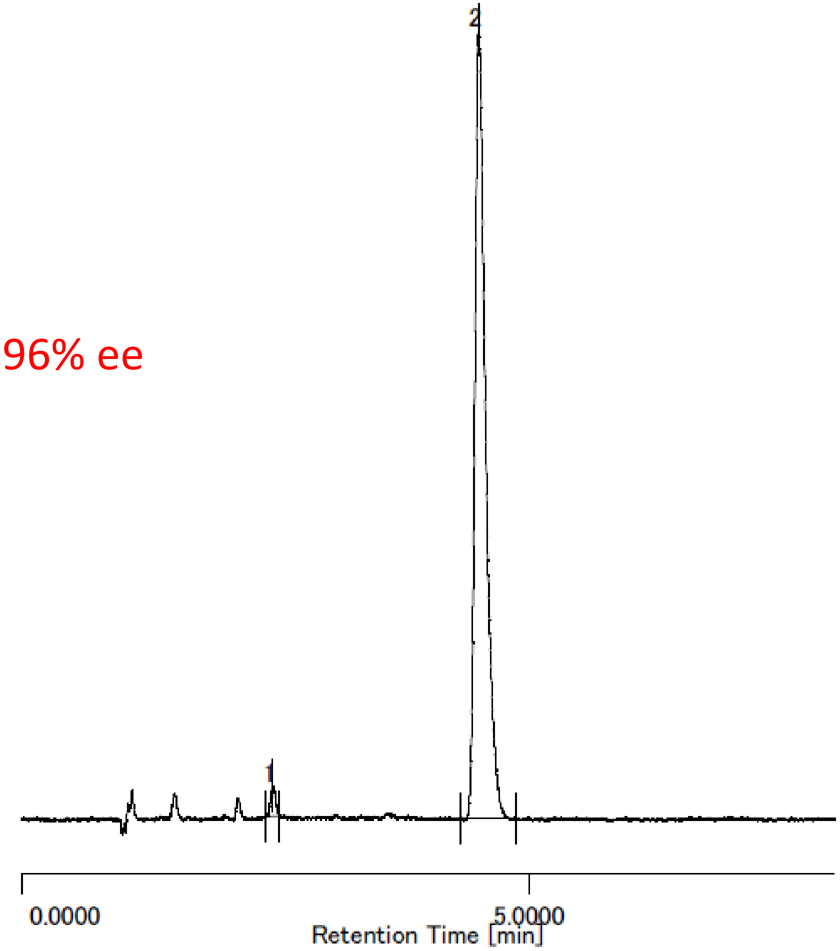

ChromatogramName R-702-rac1

#	CH	tR	Area	Height	Area%
1	9	2.4467	1630383	379574	49.024
2	9	4.3867	1695319	222357	50.976

ChromatogramName R-703-chir

#	CH	tR	Area	Height	Area%
1	9	2.4767	24759	7192	1.802
2	9	4.5000	1349495	185072	98.198

Supplementary Figure 17. ^{1}H, ^{13}C-NMR and SFC spectra of product 3ca

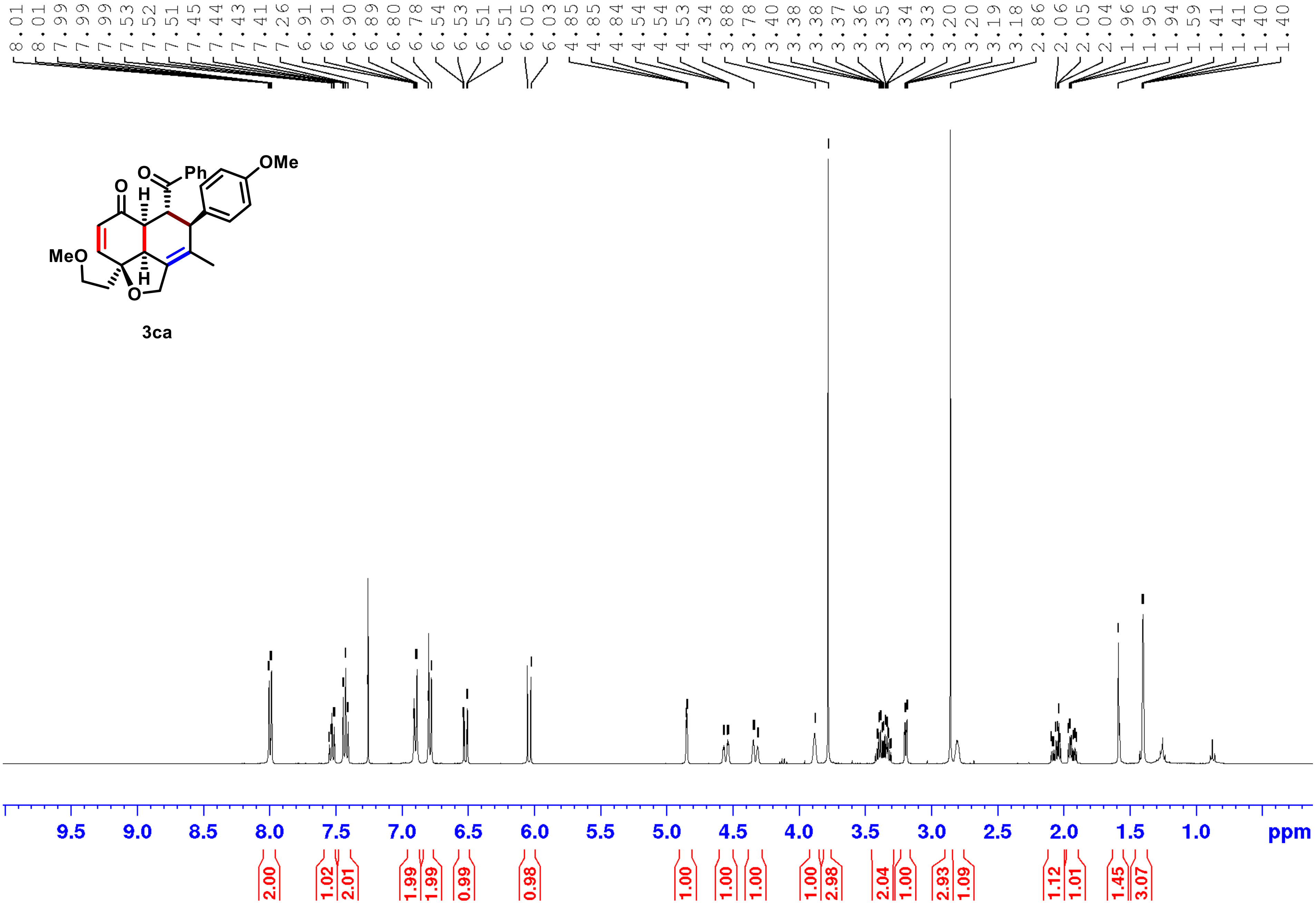

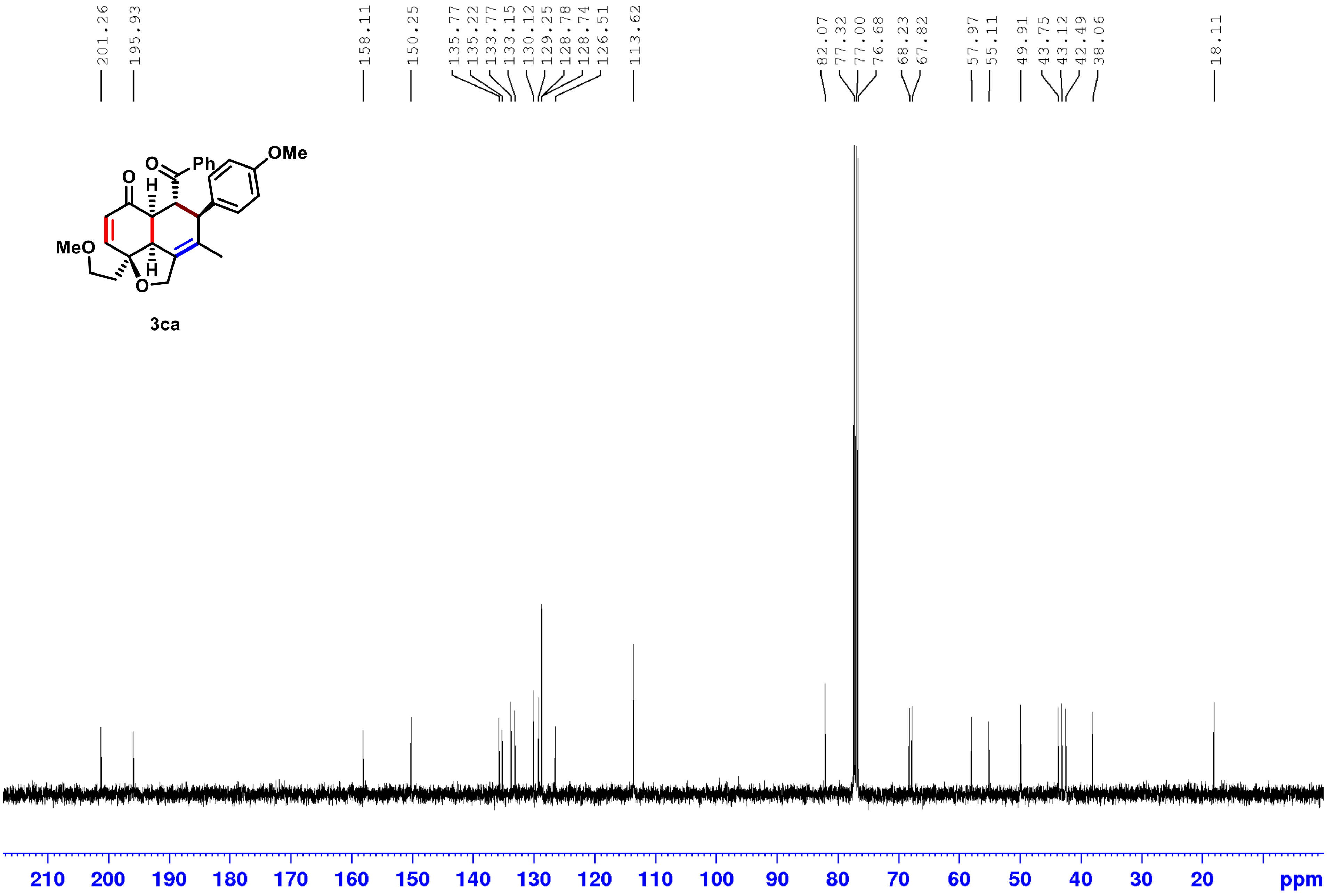
201.26
195.93
158.11
150.25
135.77
135.22
133.77
133.15
130.12
129.25
128.78
128.74
126.51
113.62
82.07
77.32
77.00
76.68
68.23
67.82
57.97
55.11
49.91
43.75
43.12
42.49
38.06
18.11
OMe
Ph
O
H
MeO
3ca
210
200
190
180
170
160
150
140
130
120
110
100
90
80
70
60
50
40
30
20
ppm

Chiral Separation (SFC): Chiralpak IA; Flow (CO_2) = 2.4 mL/min; Flow (isopropanol) = 0.6 mL/min; T = 25 ^oC; λ = 250 nm; Back pressure = 15 Mpa

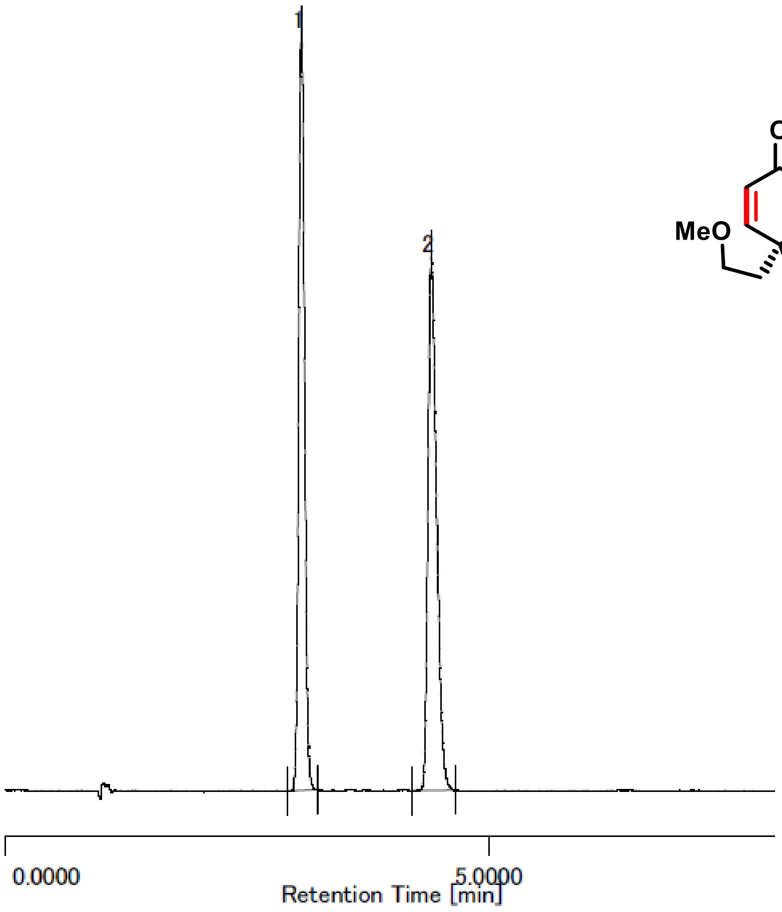

ChromatogramName R-711-rac.OMe-dienone-p-OMe-chalcone

#	CH	tR	Area	Height	Area%
1	9	3.0633	3343153	803691	48.961
2	9	4.4033	3485087	567078	51.039

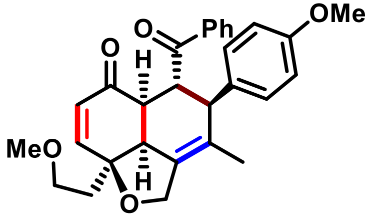

3ca

98% ee

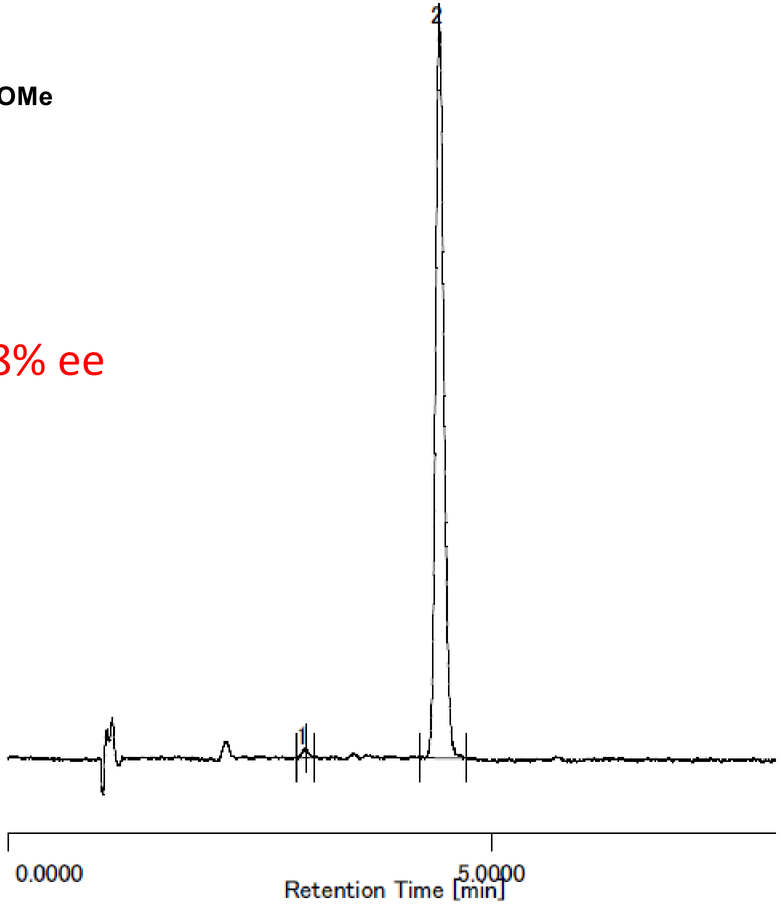

ChromatogramName R-717-Chir.OMe-dienone-p-OMe-chalcone

#	CH	tR	Area	Height	Area%
1	9	3.0767	7698	1757	0.962
2	9	4.4567	792556	131448	99.038

Supplementary Figure 18. ^{1}H, ^{13}C-NMR and SFC spectra of product 3ck

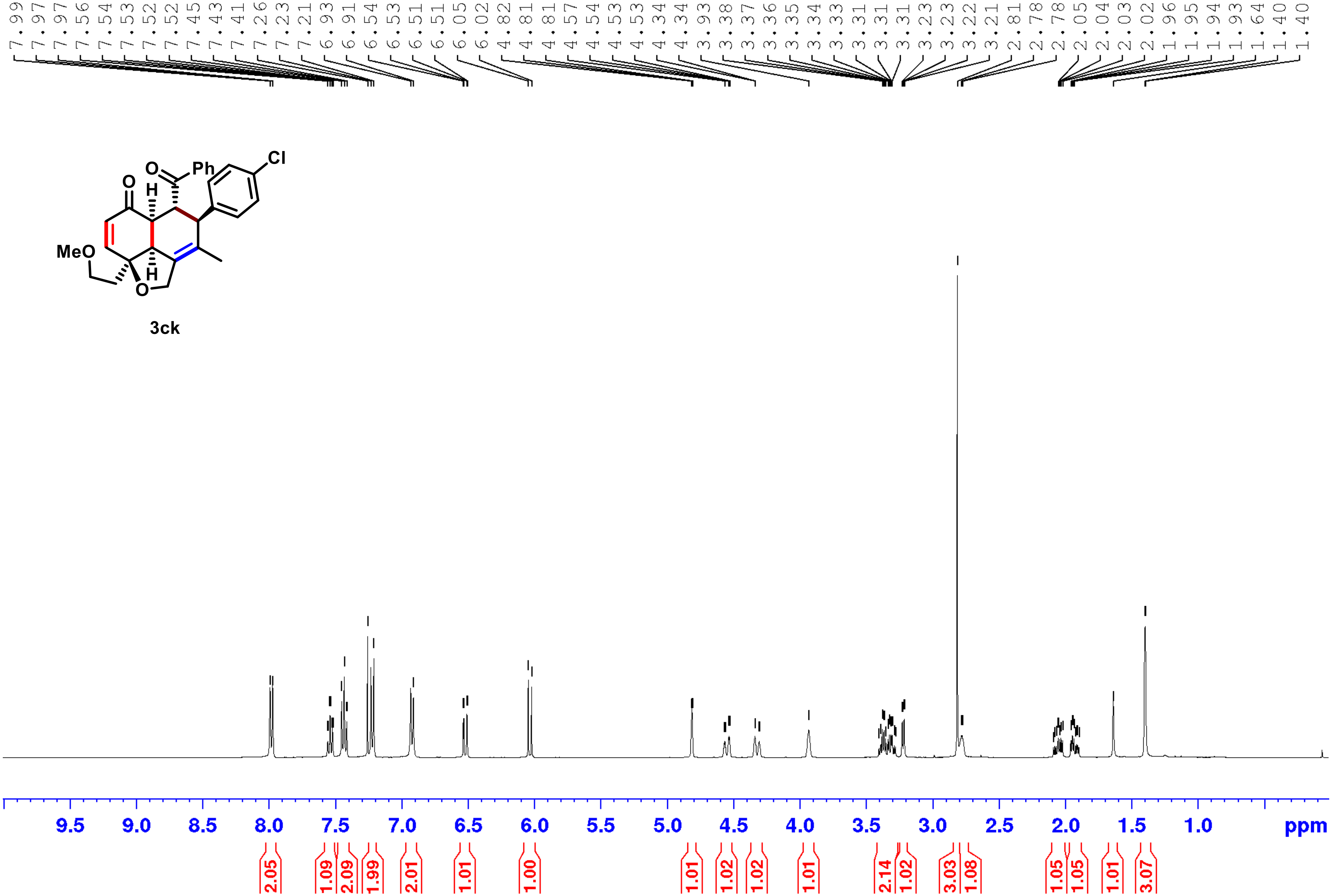

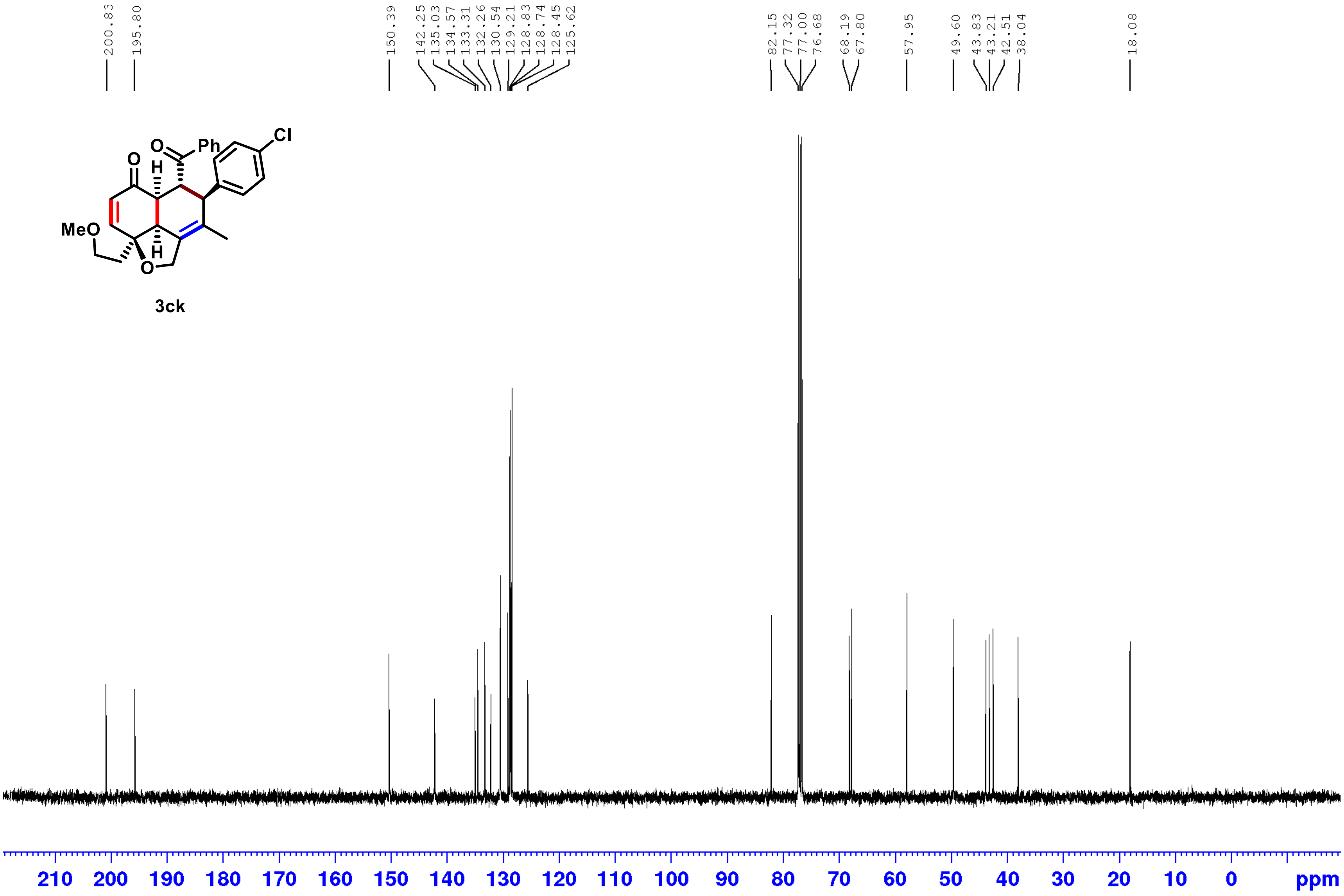
200.83
195.80
150.39
142.25
135.03
134.57
133.31
132.26
130.54
129.21
128.83
128.74
128.45
125.62
82.15
77.32
77.00
76.68
68.19
67.80
57.95
49.60
43.83
43.21
42.51
38.04
18.08
O
Ph
Cl
H
MeO
3ck
210 200 190 180 170 160 150 140 130 120 110 100 90 80 70 60 50 40 30 20 10 0 ppm

Chiral Separation (SFC): Chiralpak IA; Flow (CO_2) = 2.4 mL/min; Flow (isopropanol) = 0.6 mL/min; T = 40 ^{o}C; λ = 250 nm; Back pressure = 15 Mpa

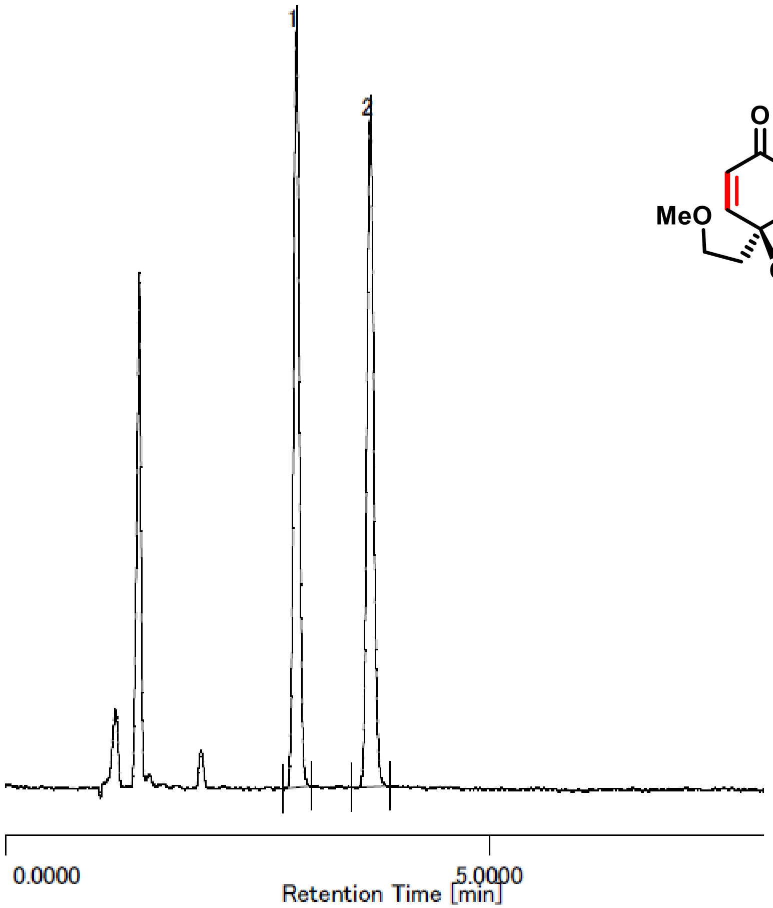

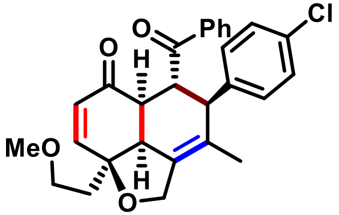

3ck

98% ee

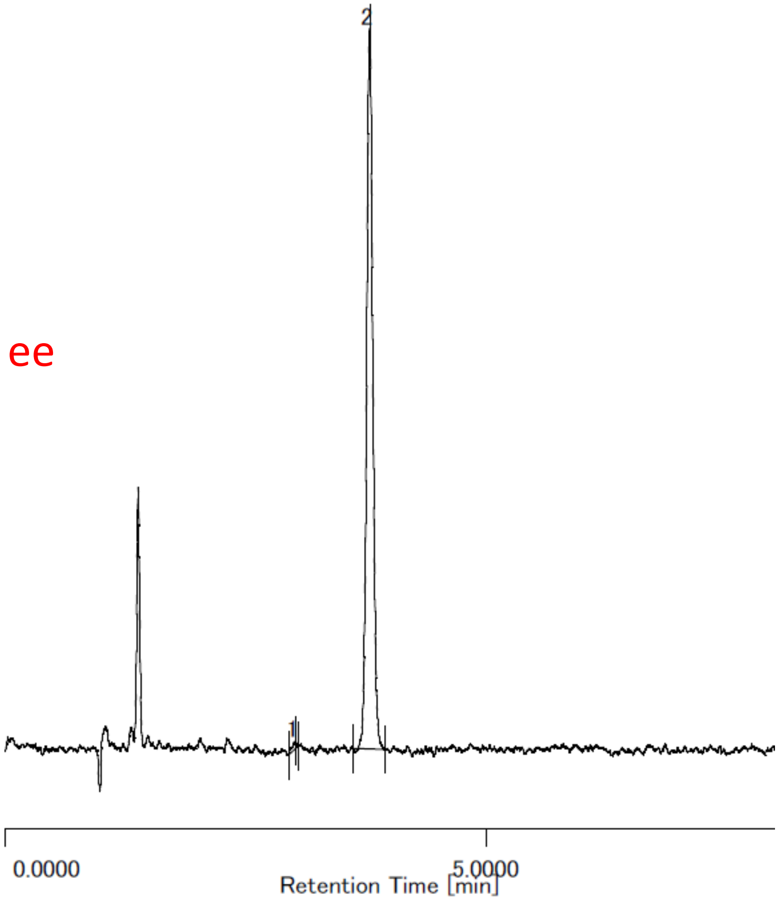

ChromatogramName R-722-rac.OMe-ethyl-dienone+p-Cl-chalcone

#	CH	tR	Area	Height	Area%
1	10	3.0067	4359775	1071314	48.999
2	10	3.7700	4537921	945607	51.001

ChromatogramName R-725-Chiral-OMe-ethyl-dienone+p-Cl-chalcone

#	CH	tR	Area	Height	Area%
1	9	3.0167	10187	2924	0.839
2	9	3.7883	1203730	272592	99.161

Supplementary Figure 19. ^{1}H, ^{13}C-NMR and SFC spectra of product 3de

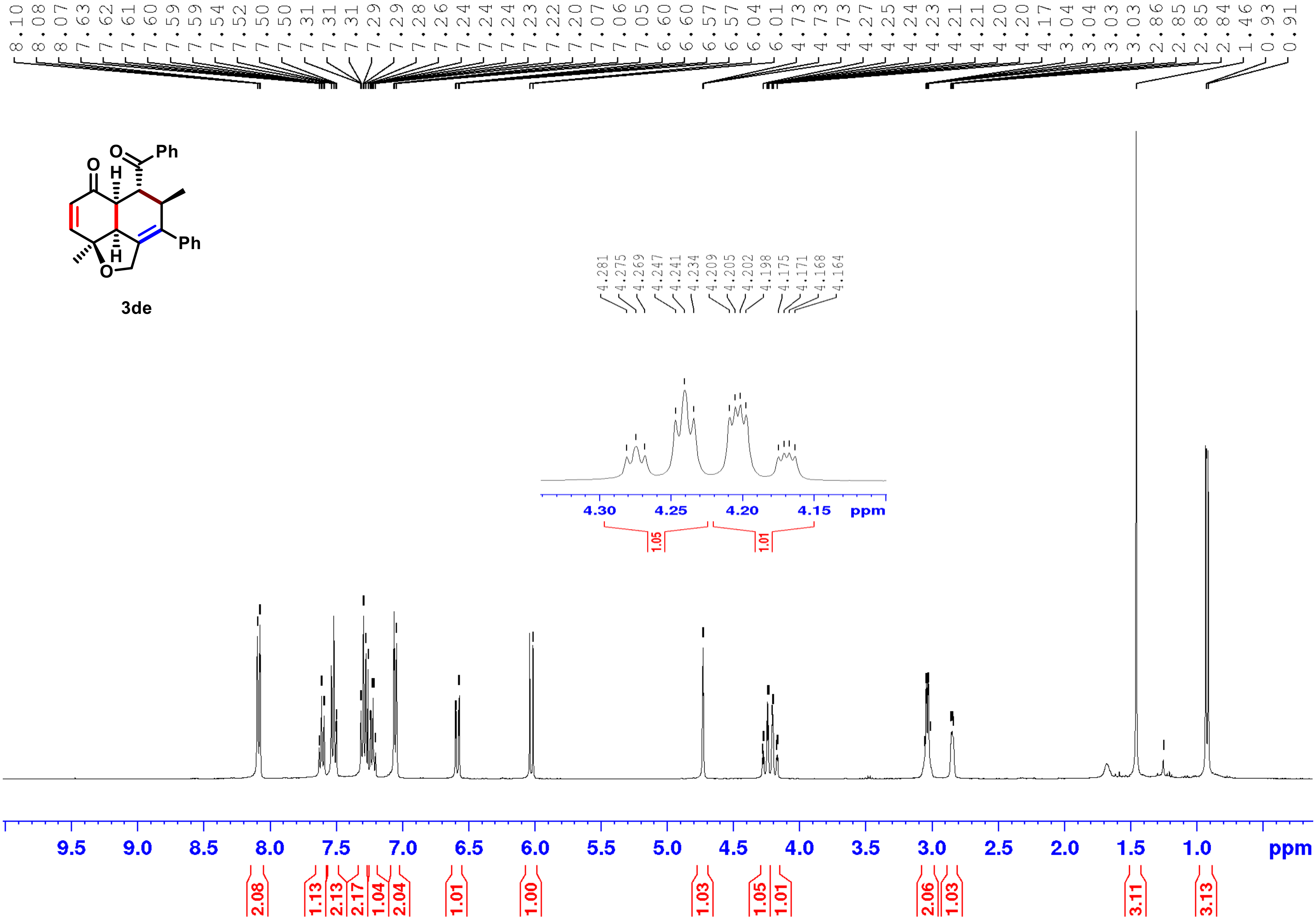

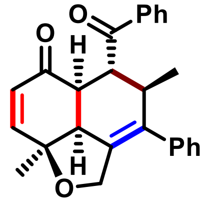
O
Ph
O
H
H
Ph
O

3de

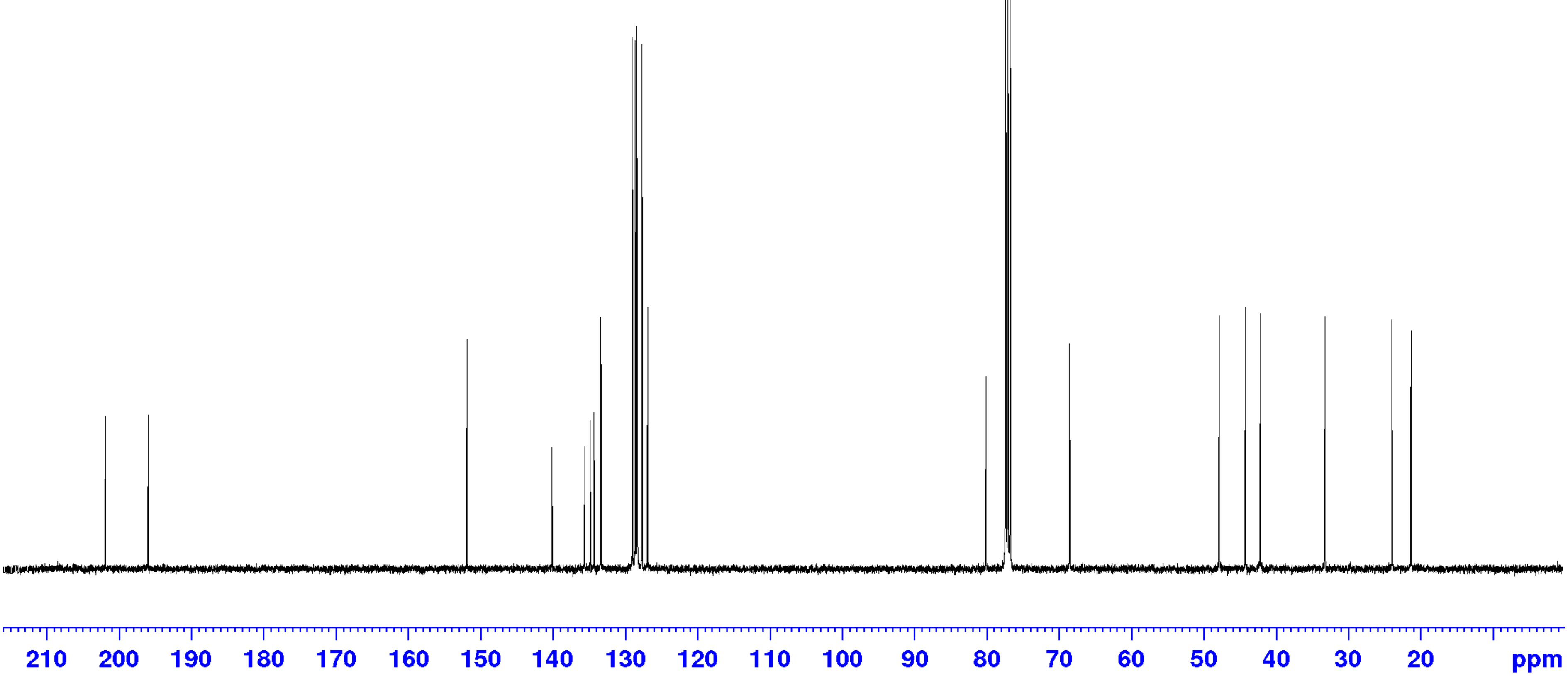
201.96
196.03
151.85
140.07
135.53
134.78
134.24
133.30
128.94
128.54
128.33
128.25
127.59
126.84
80.02
77.30
76.99
76.67
68.48
47.77
44.14
42.09
33.16
23.87
21.25
210 200 190 180 170 160 150 140 130 120 110 100 90 80 70 60 50 40 30 20 ppm

Chiral Separation (SFC): Chiralpak IA; Flow (CO_2) = 3.0 mL/min; Flow (isopropanol) = 0.3 mL/min; T = 25 ºC; λ = 250 nm; Back pressure = 15 Mpa

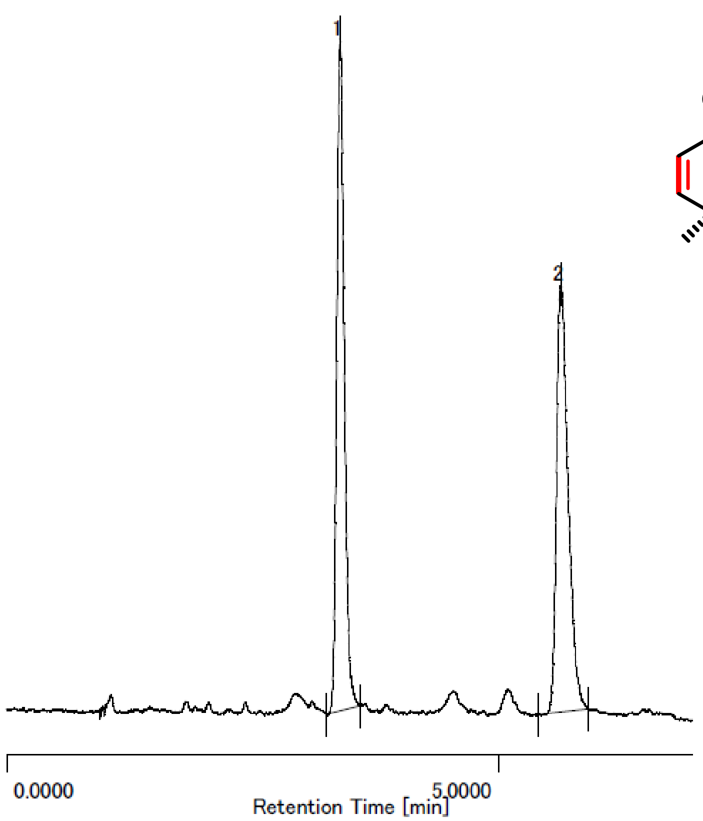

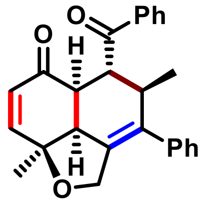

3de

99% ee

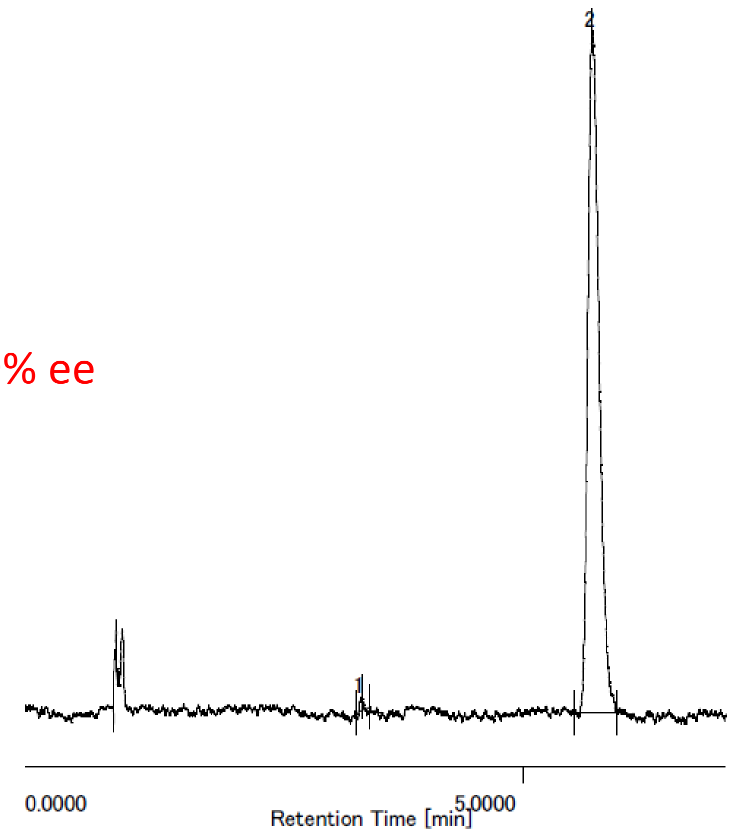

ChromatogramName E-632-rac-Ph-dienone+PPK

#	CH	tR	Area	Height	Area%
1	9	3.3883	1073578	201812	50.484
2	9	5.6283	1052997	128546	49.516

ChromatogramName R-659rr-chir-Ph-dienone+PPK-fr1

#	CH	tR	Area	Height	Area%
1	9	3.4033	2340	1119	0.543
2	9	5.6800	428426	55855	99.457

Supplementary Figure 20. ¹H, ¹³C-NMR and SFC spectra of product 3ac

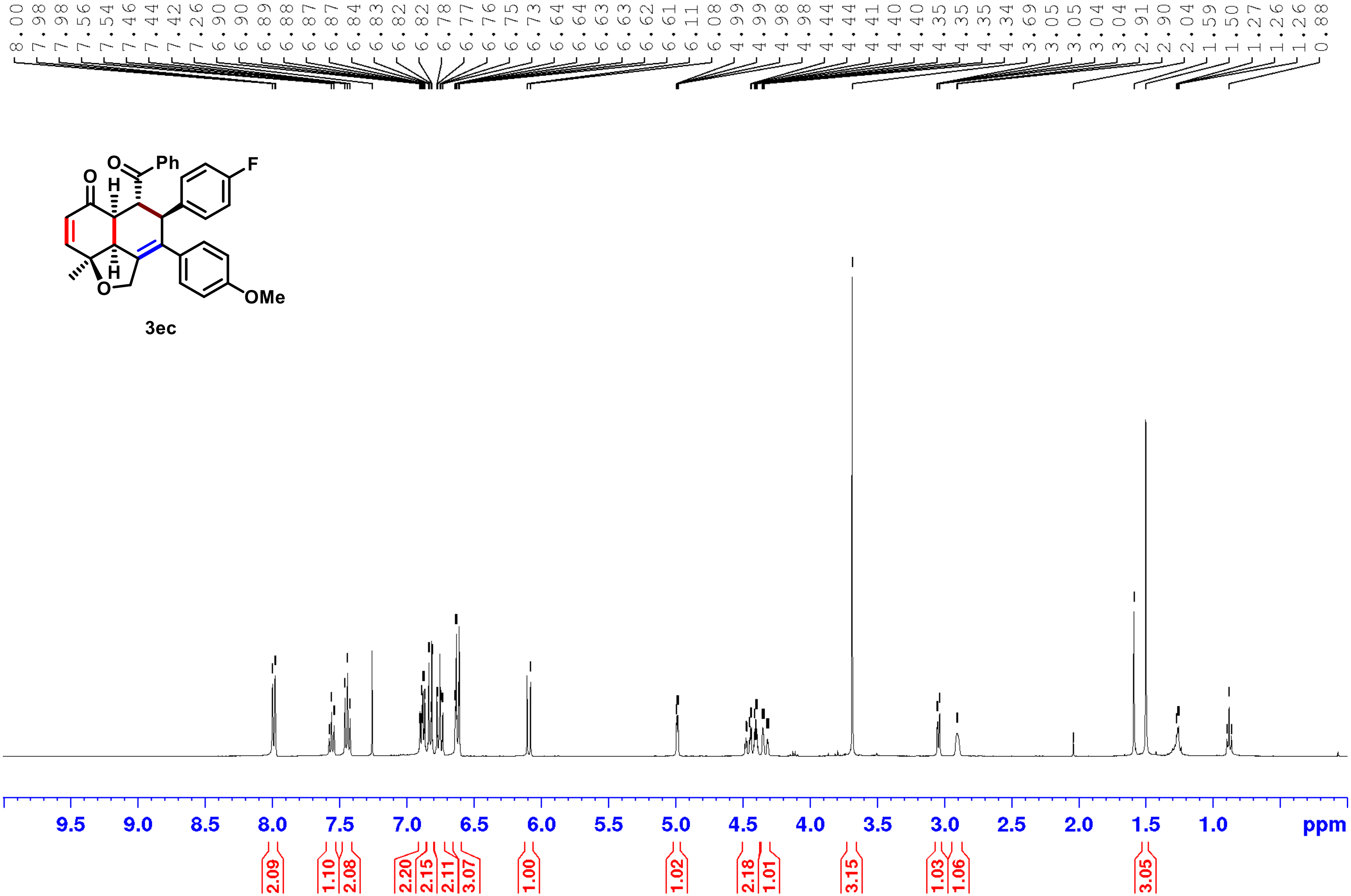

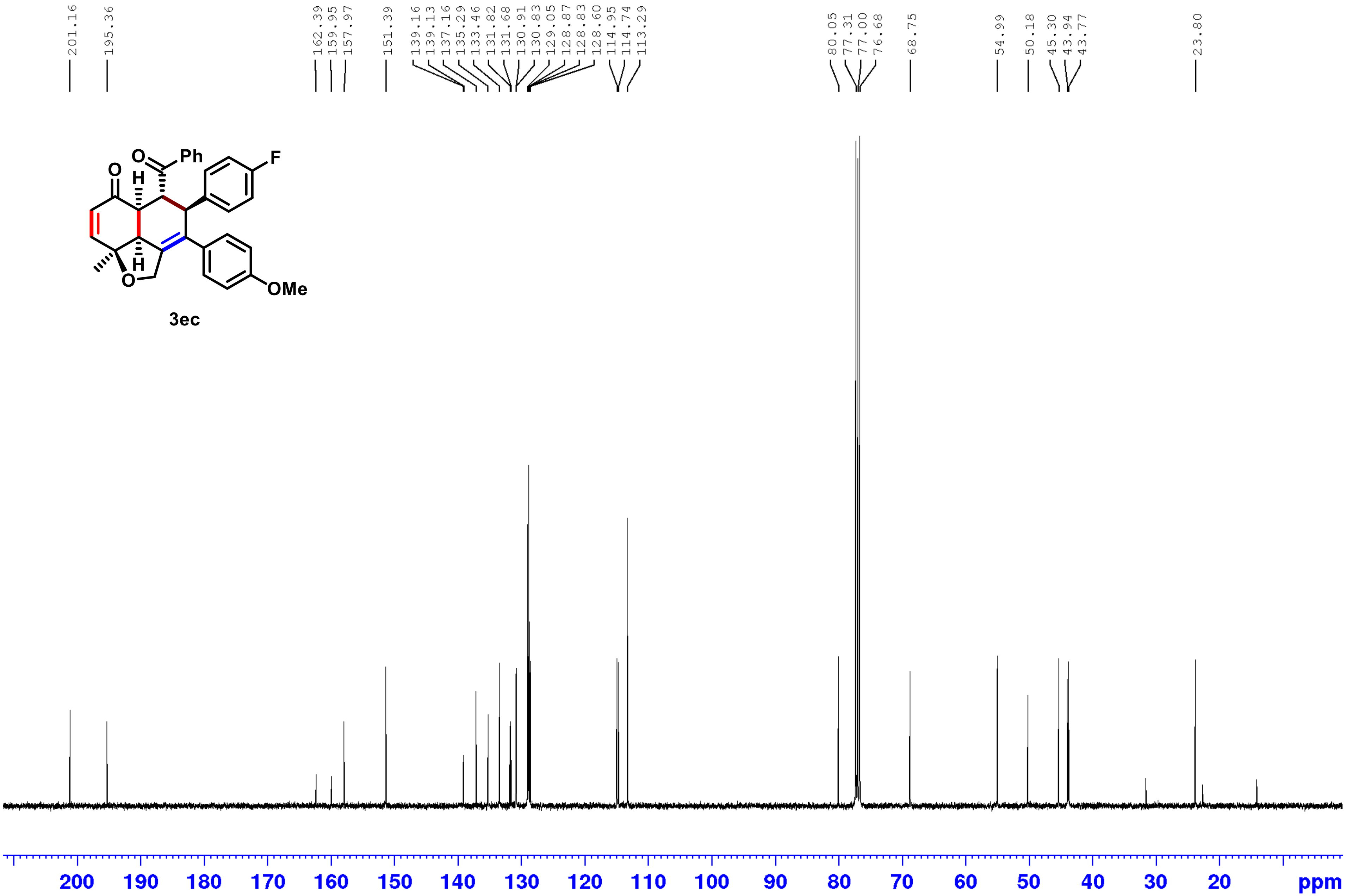
201.16
195.36
162.39
159.95
157.97
151.39
139.16
139.13
137.16
135.29
133.46
131.82
131.68
130.91
130.83
129.05
128.87
128.83
128.60
114.95
114.74
113.29
80.05
77.31
77.00
76.68
68.75
54.99
50.18
45.30
43.94
43.77
23.80
O
Ph
F
O
H
H
O
OMe
3ec
200
190
180
170
160
150
140
130
120
110
100
90
80
70
60
50
40
30
20
ppm

<u>Chiral Separation</u> (SFC): Chiralpak IA; Flow (CO_2) = 2.4 mL/min; Flow (isopropanol) = 0.6 mL/min; T = 40 °C; λ = 250 nm; Back pressure = 15 Mpa

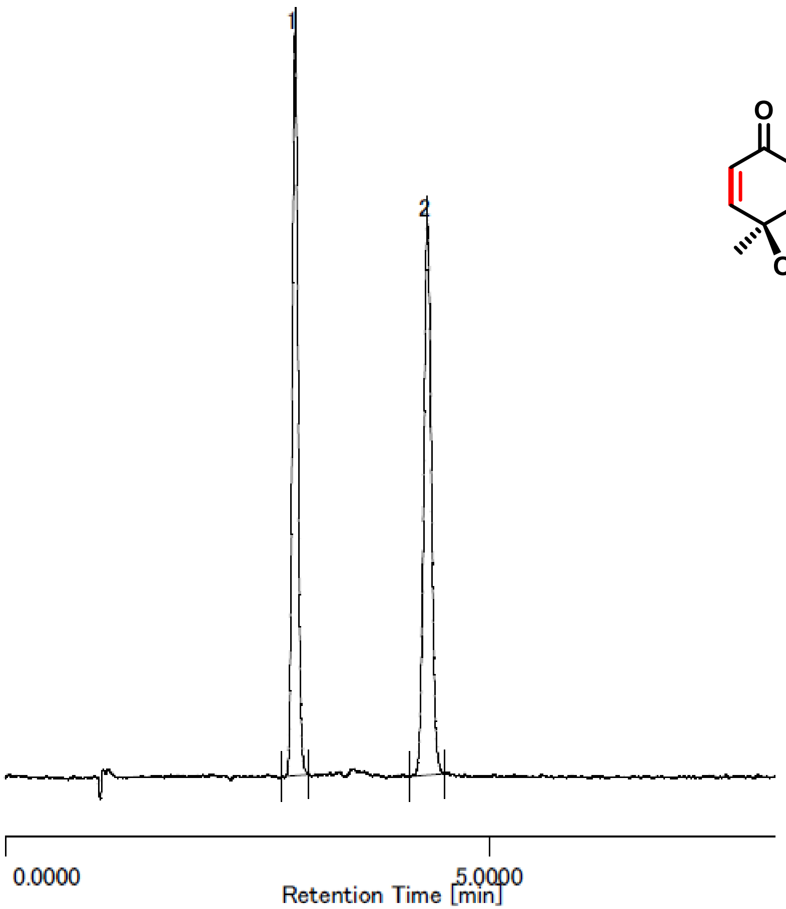

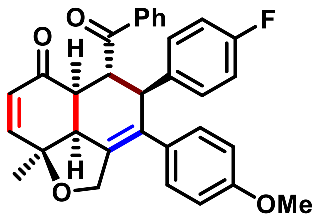

3ec

98% ee

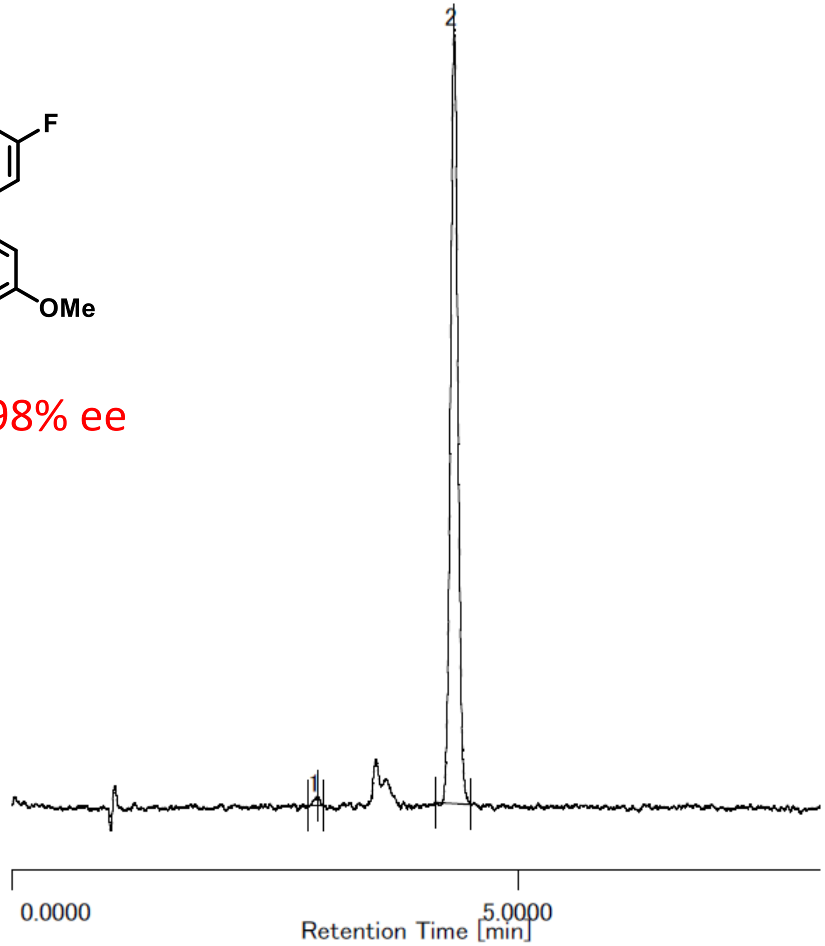

ChromatogramName R-723-rac.dienone-p-OMe-aryl-yne+p-F-chalcone

#	CH	tR	Area	Height	Area%
1	9	2.9917	2826067	717767	49.073
2	9	4.3583	2932789	536083	50.927

ChromatogramName R-726-Chiral-dienone-p-OMe-aryl-yne+p-F-chalcone

#	CH	tR	Area	Height	Area%
1	10	3.0200	20171	5329	0.946
2	10	4.3683	2111457	412793	99.054

Supplementary Figure 21. ^{1}H, ^{13}C-NMR and SFC spectra of product 3ek

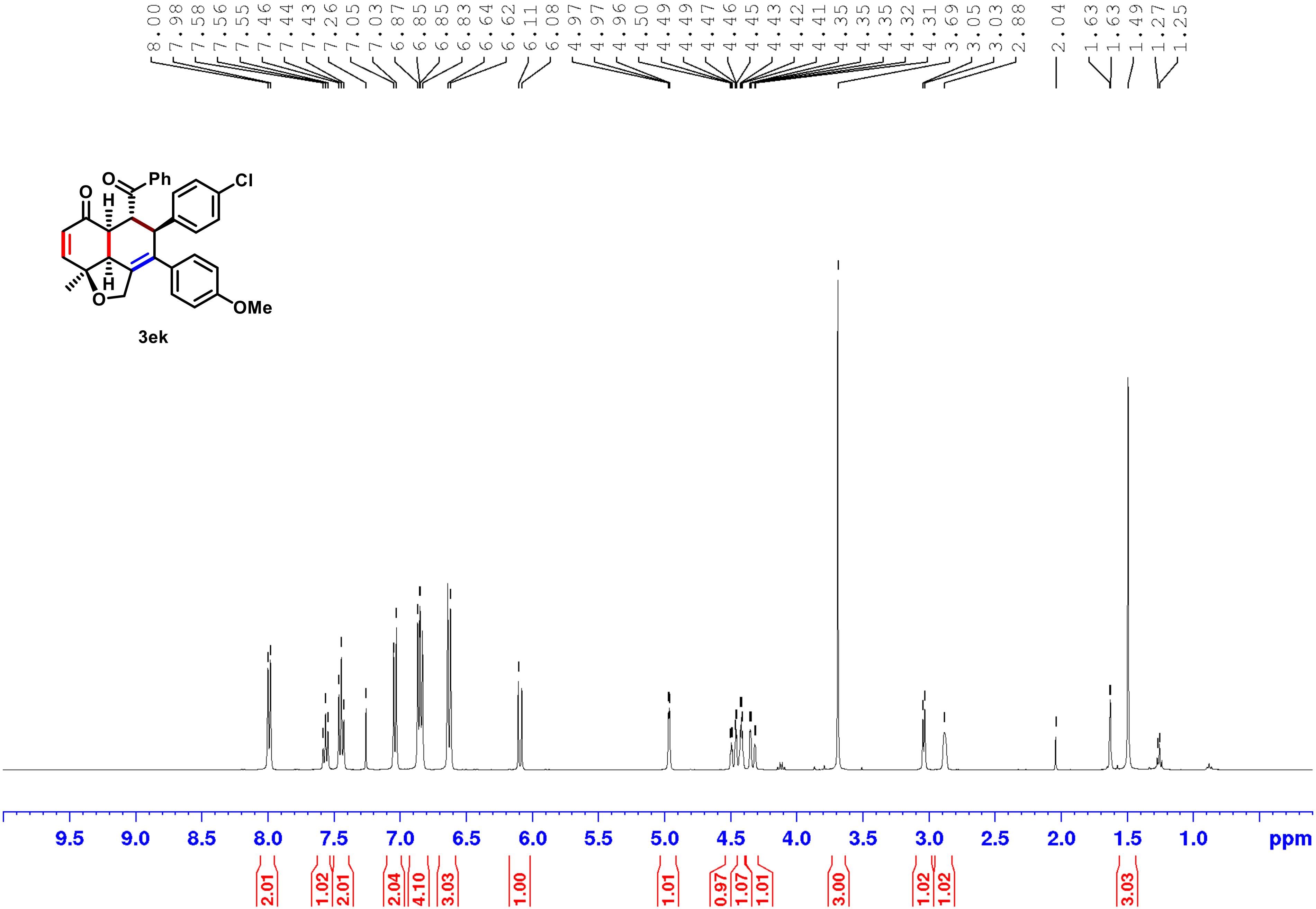

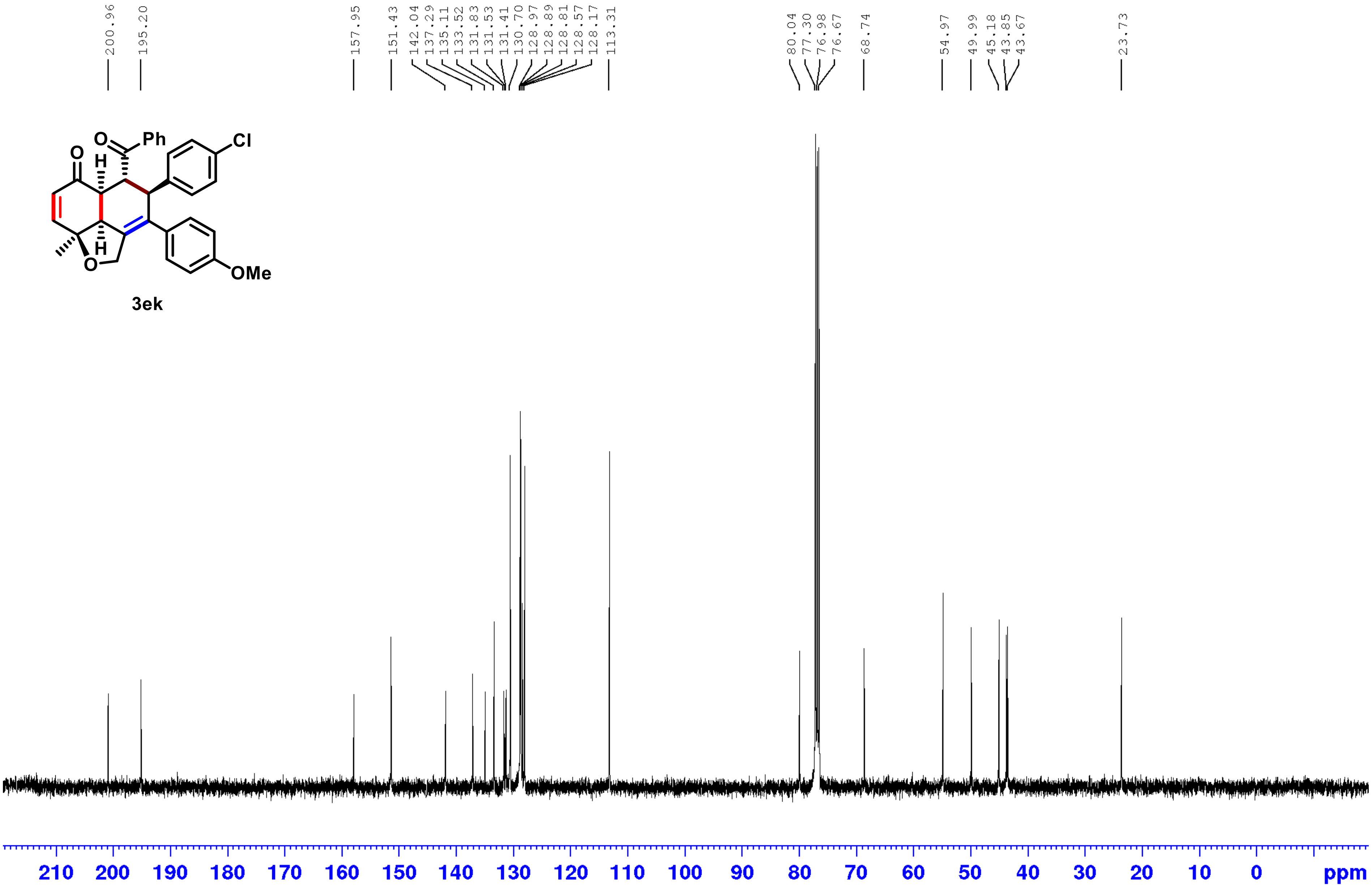
200.96
195.20
157.95
151.43
142.04
137.29
135.11
133.52
131.83
131.53
131.41
130.70
128.97
128.89
128.81
128.57
128.17
113.31
80.04
77.30
76.98
76.67
68.74
54.97
49.99
45.18
43.85
43.67
23.73
O
Ph
Cl
O
H
H
O
OMe
3ek
210 200 190 180 170 160 150 140 130 120 110 100 90 80 70 60 50 40 30 20 10 0 ppm

Chiral Separation (SFC): Chiralpak IA; Flow (CO_2) = 2.4 mL/min; Flow (isopropanol) = 0.6 mL/min; T = 40 ºC; λ = 250 nm; Back pressure = 15 Mpa

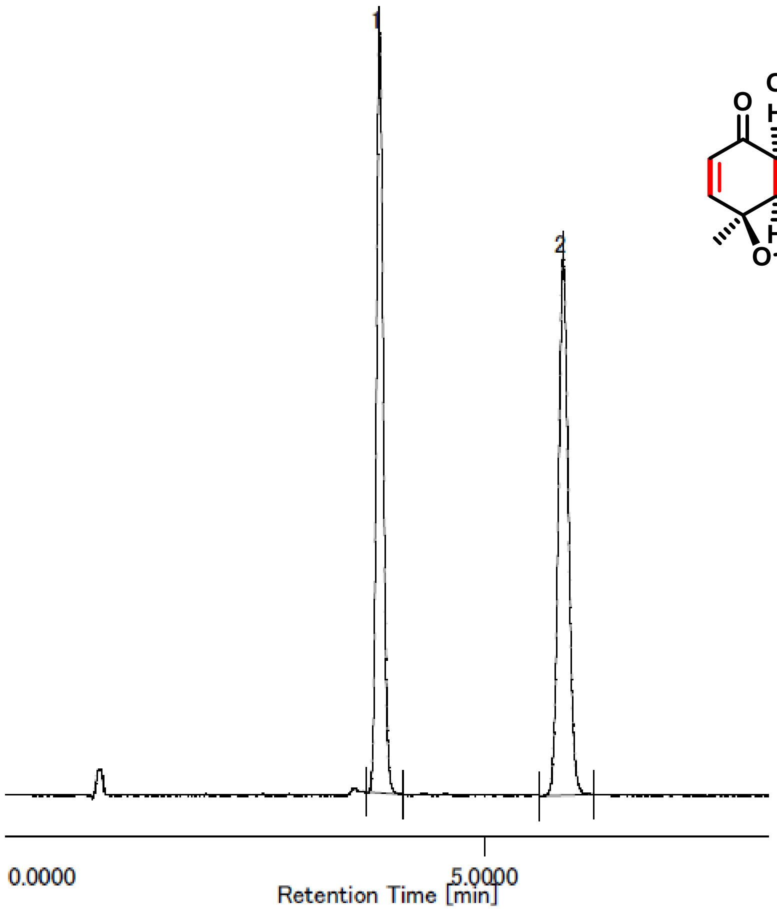

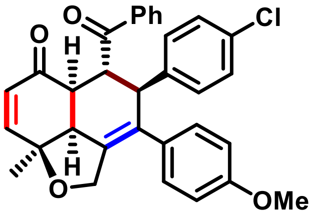

3ek

99% ee

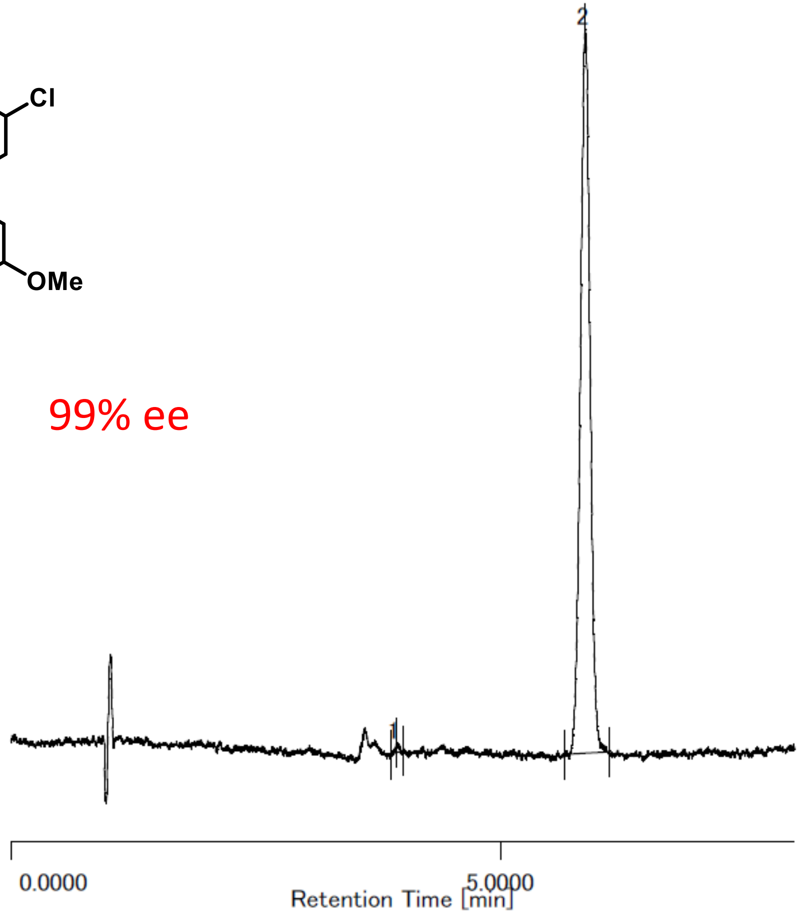

ChromatogramName R-733-rac.dienone-p-OMe-aryl-yne+p-Cl-chalcone

#	CH	tR	Area	Height	Area%
1	9	3.9150	4509356	925913	48.951
2	9	5.8083	4702578	656560	51.049

ChromatogramName R-734-chiral-dienone-p-OMe-aryl-yne+p-Cl-chalcone

#	CH	tR	Area	Height	Area%
1	9	3.9383	1628	698	0.430
2	9	5.8667	376918	55122	99.570

Supplementary Figure 22. ^{1}H, ^{13}C-NMR and SFC spectra of product 3fc

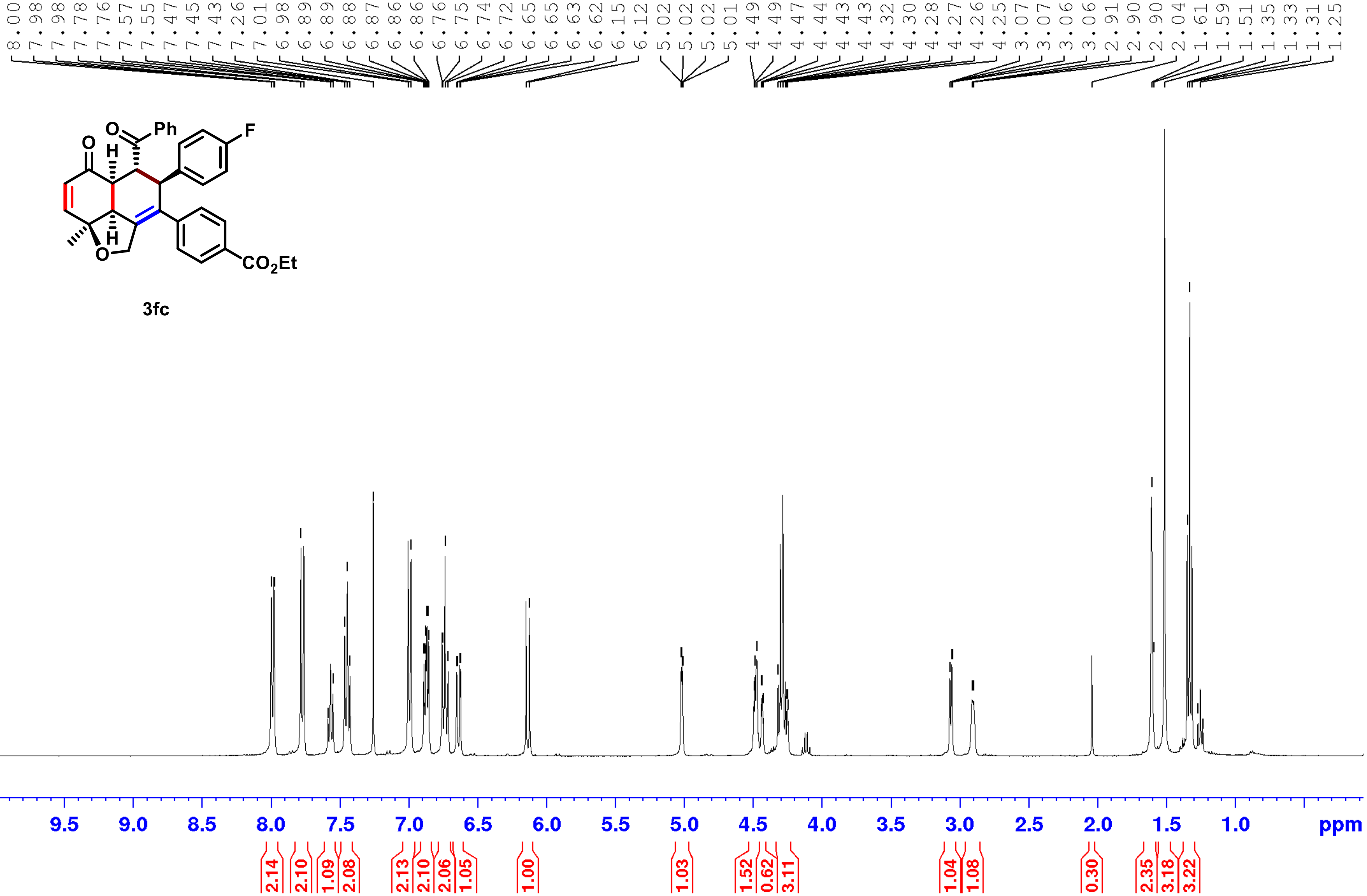

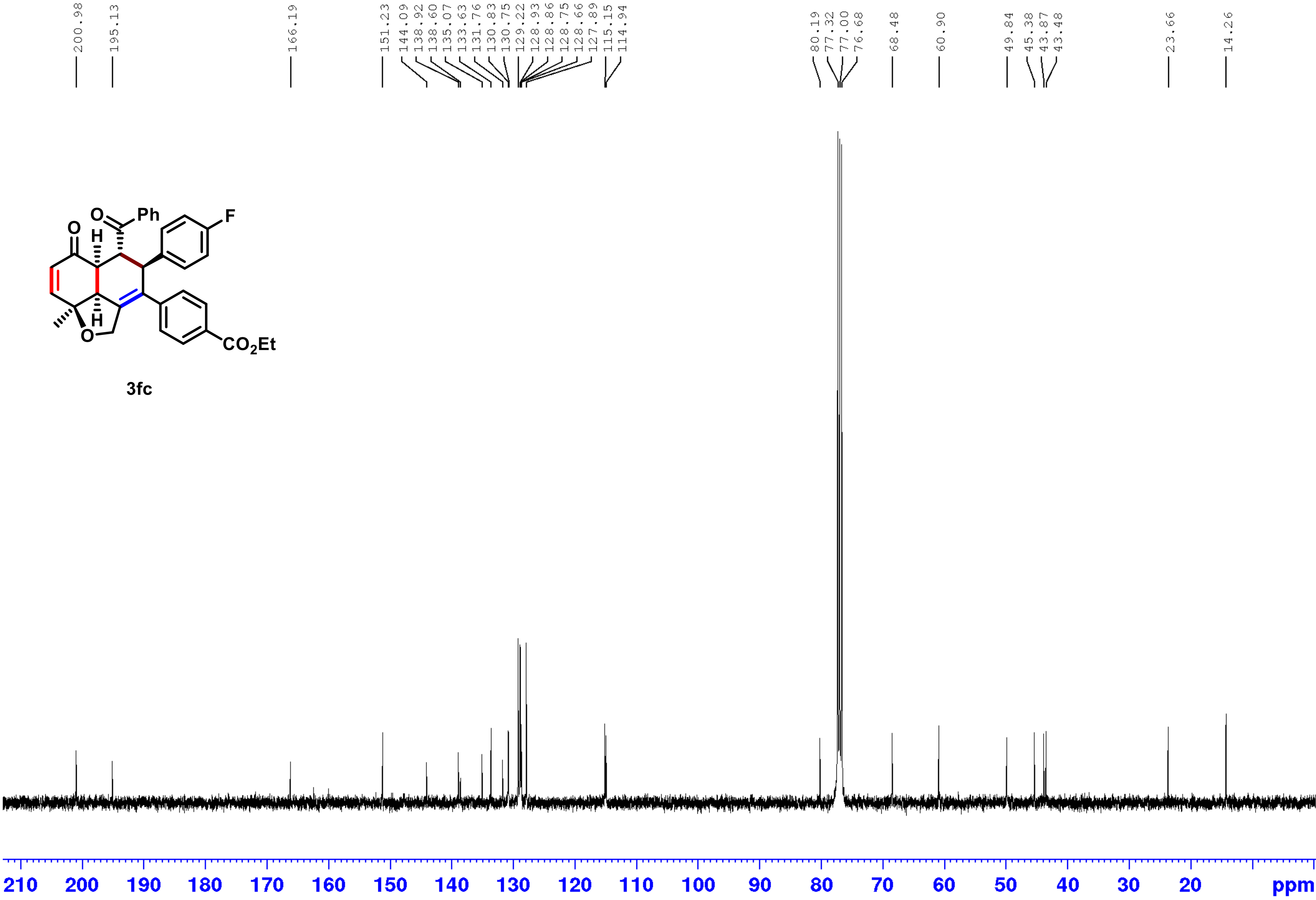
200.98
195.13
166.19
151.23
144.09
138.92
138.60
135.07
133.63
131.76
130.83
130.75
129.22
128.93
128.86
128.75
128.66
127.89
115.15
114.94
80.19
77.32
77.00
76.68
68.48
60.90
49.84
45.38
43.87
43.48
23.66
14.26
O
Ph
F
O
H
H
O
CO2Et
3fc
210
200
190
180
170
160
150
140
130
120
110
100
90
80
70
60
50
40
30
20
ppm

Chiral Separation (SFC): Chiralpak IA; Flow (CO_2) = 2.4 mL/min; Flow (isopropanol) = 0.6 mL/min; T = 40 ºC; λ = 250 nm; Back pressure = 15 Mpa

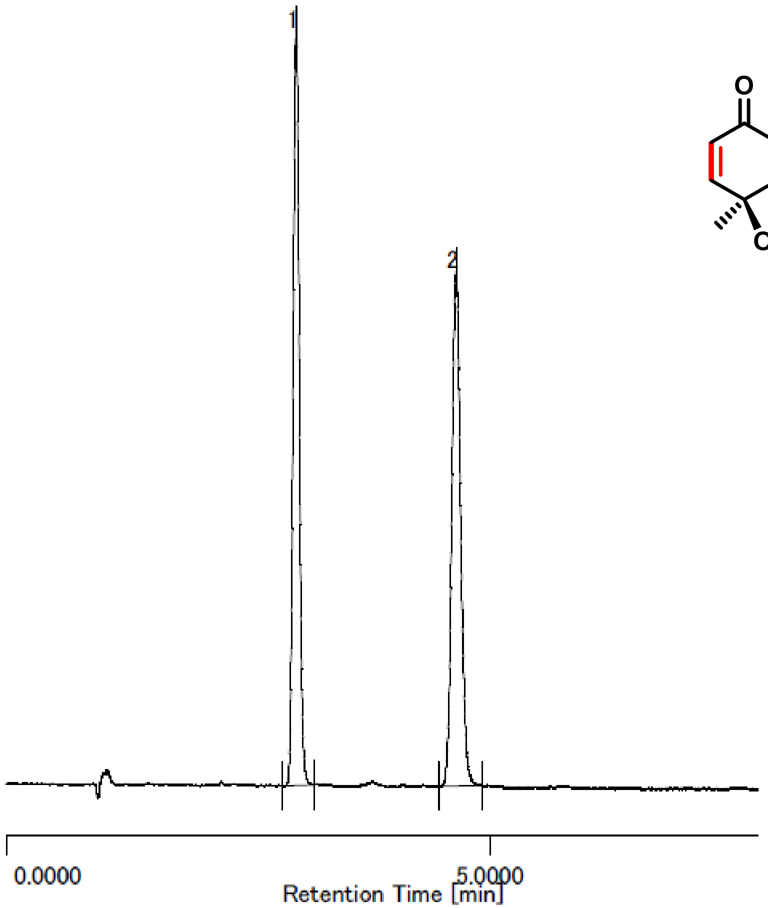

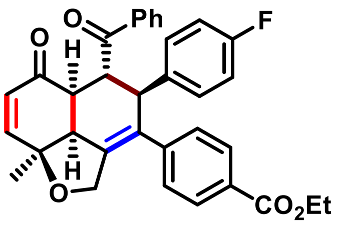

3fc

98% ee

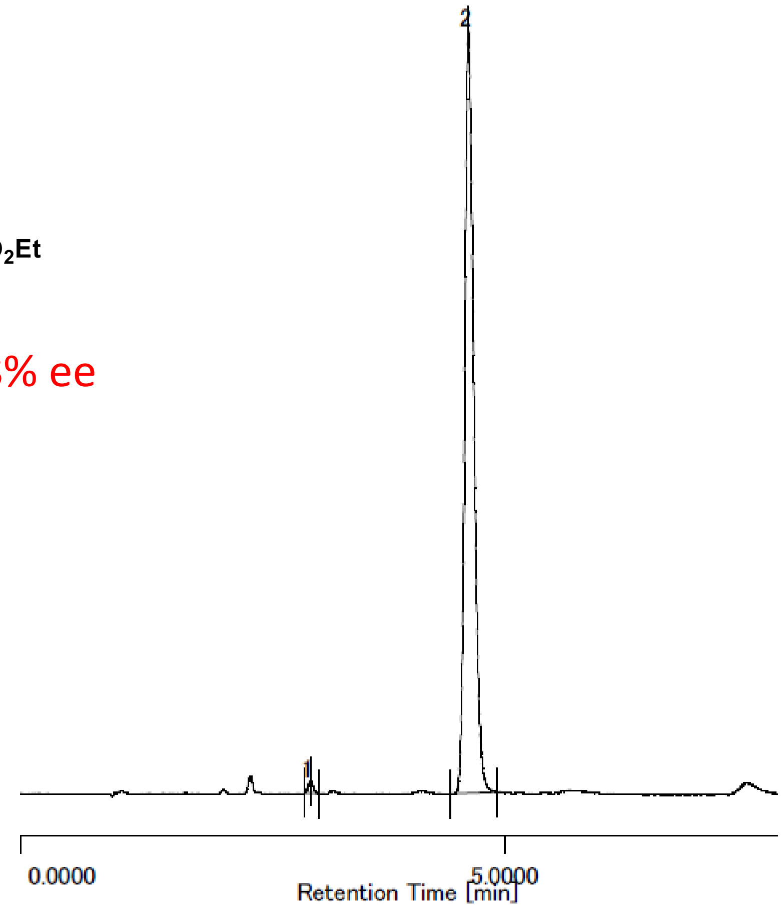

ChromatogramName R-737-rac.dienone-p-Ester-aryl-yne+p-F-chalcone

#	CH	tR	Area	Height	Area%
1	9	2.9950	1020592	235813	49.980
2	9	4.6467	1021394	160901	50.020

ChromatogramName R-739-Chiral-dienone-p-Ester-aryl-yne+p-F-chalcone

#	CH	tR	Area	Height	Area%
1	9	2.9967	83254	21734	0.991
2	9	4.6233	8315369	1292170	99.009

Supplementary Figure 23. ^{1}H, ^{13}C-NMR and SFC spectra of product 3ge

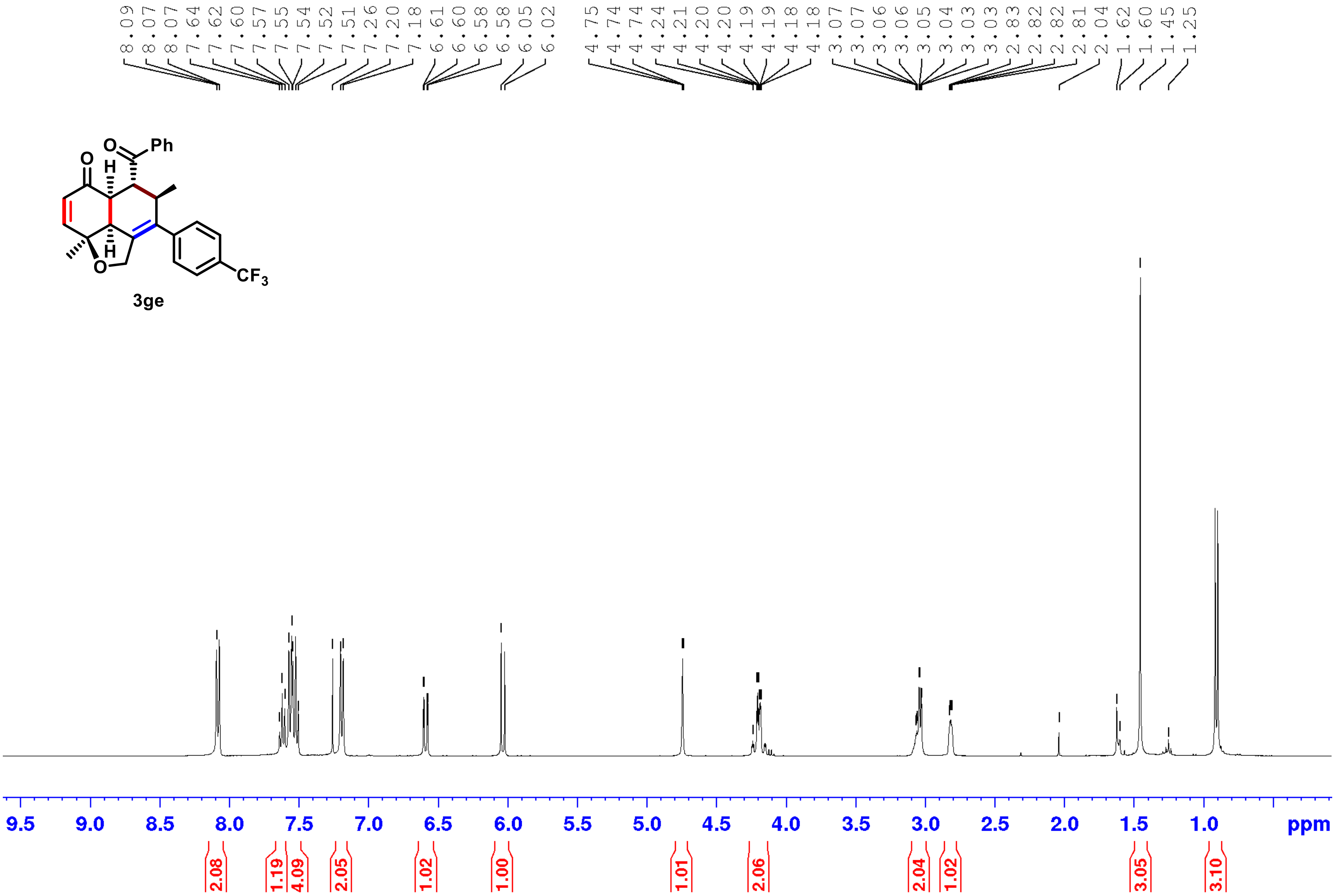

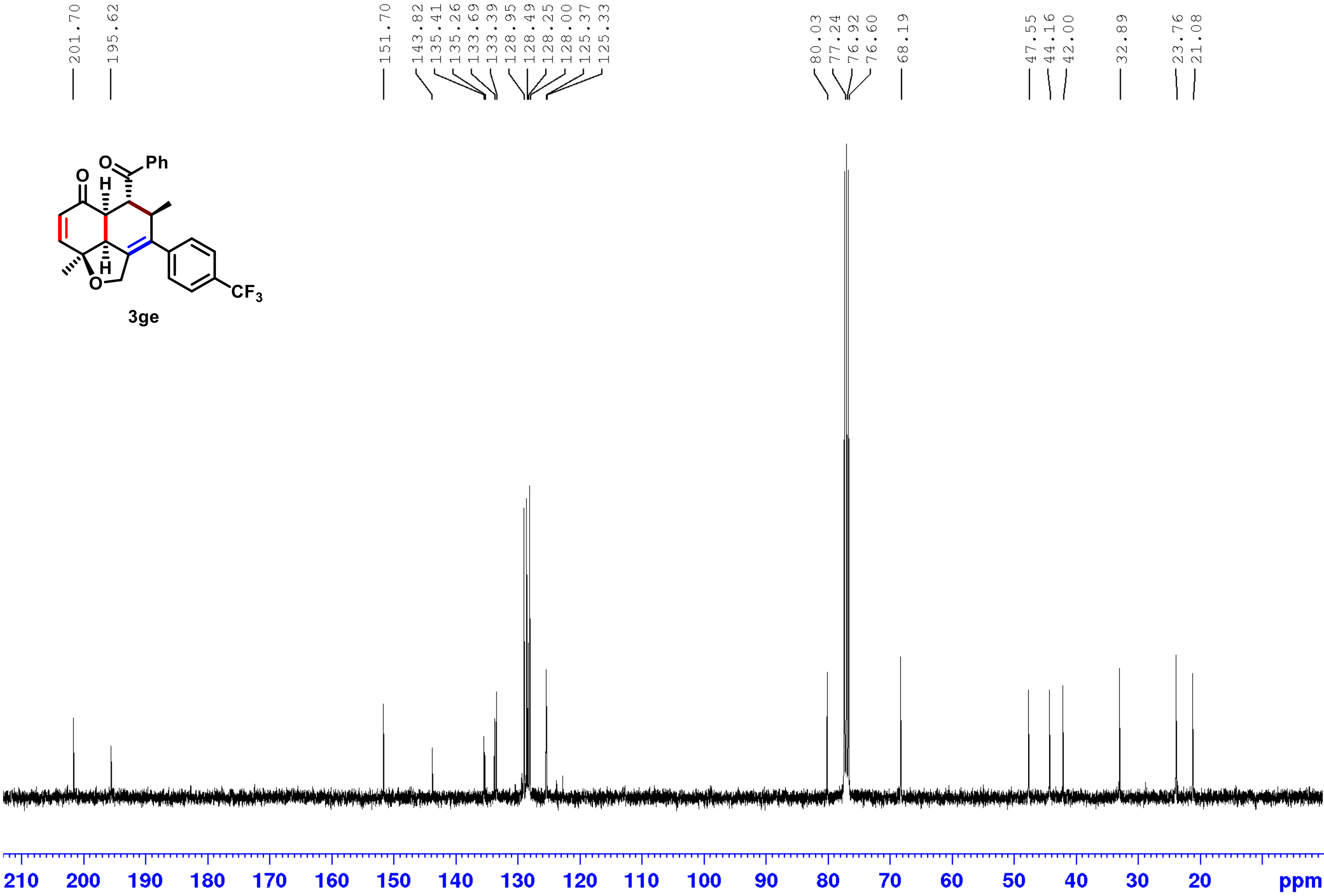
201.70
195.62
151.70
143.82
135.41
135.26
133.69
133.39
128.95
128.49
128.25
128.00
125.37
125.33
80.03
77.24
76.92
76.60
68.19
47.55
44.16
42.00
32.89
23.76
21.08
O
Ph
O
H
H
O
CF3
3ge
210
200
190
180
170
160
150
140
130
120
110
100
90
80
70
60
50
40
30
20
ppm

Chiral Separation (SFC): Chiralpak IA; Flow (CO_2) = 3.0 mL/min; Flow (isopropanol) = 0.3 mL/min; T = 40 ^{o}C; λ = 250 nm; Back pressure = 15 Mpa

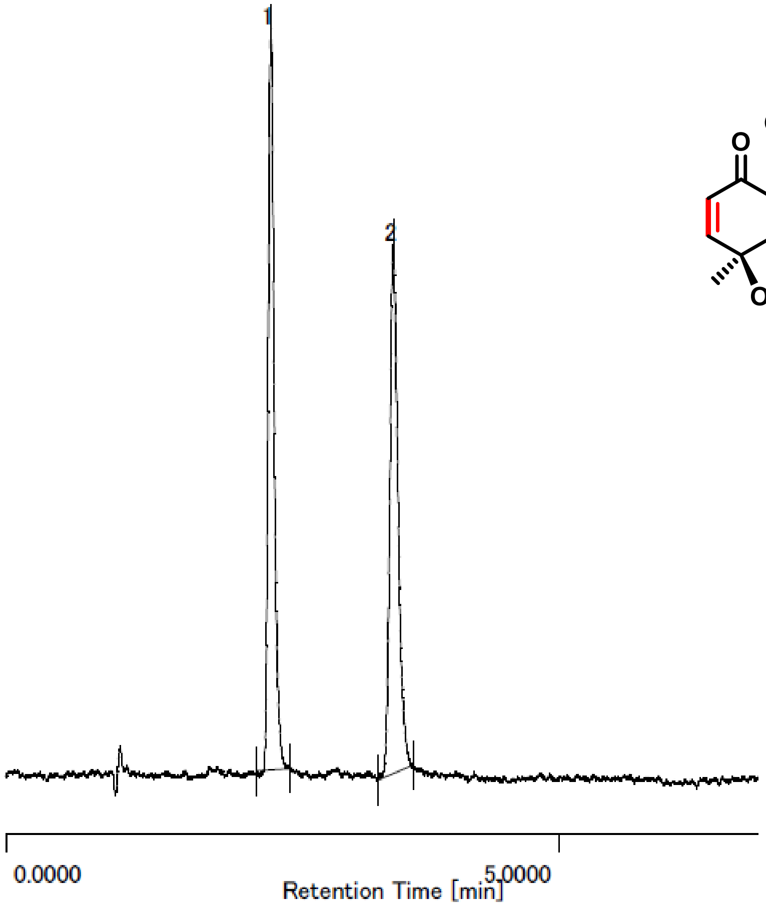

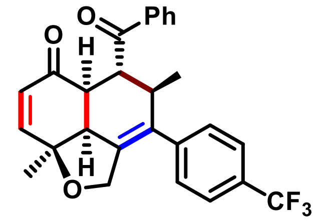

3ge

96% ee

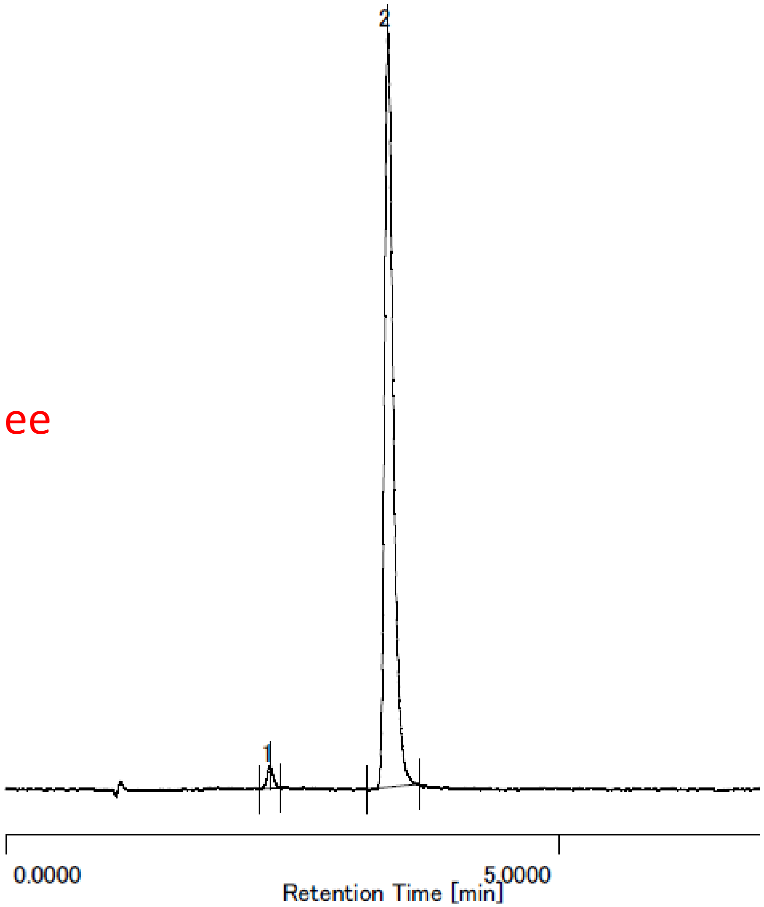

ChromatogramName R-751-rac.p-CF3-Ph-dienone+PPK

#	CH	tR	Area	Height	Area%
1	9	2.3933	373619	101107	49.853
2	9	3.5000	375816	72261	50.147

ChromatogramName R-754-chir-p-CF3-Ph-dienone+PPK

#	CH	tR	Area	Height	Area%
1	9	2.3900	40185	10505	2.073
2	9	3.4517	1898002	345869	97.927

Supplementary Figure 24. ^{1}H, ^{13}C-NMR and SFC spectra of product 3he

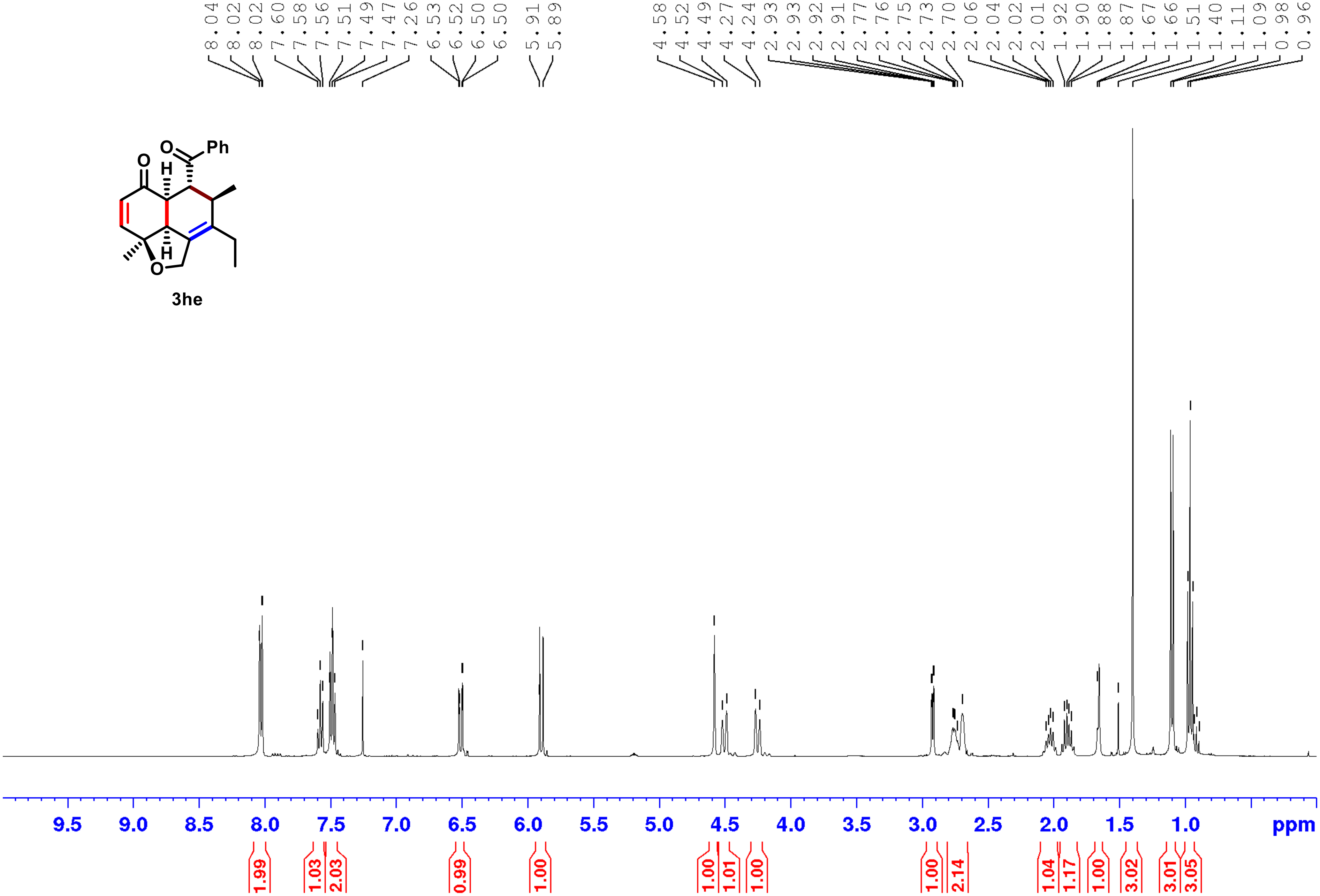

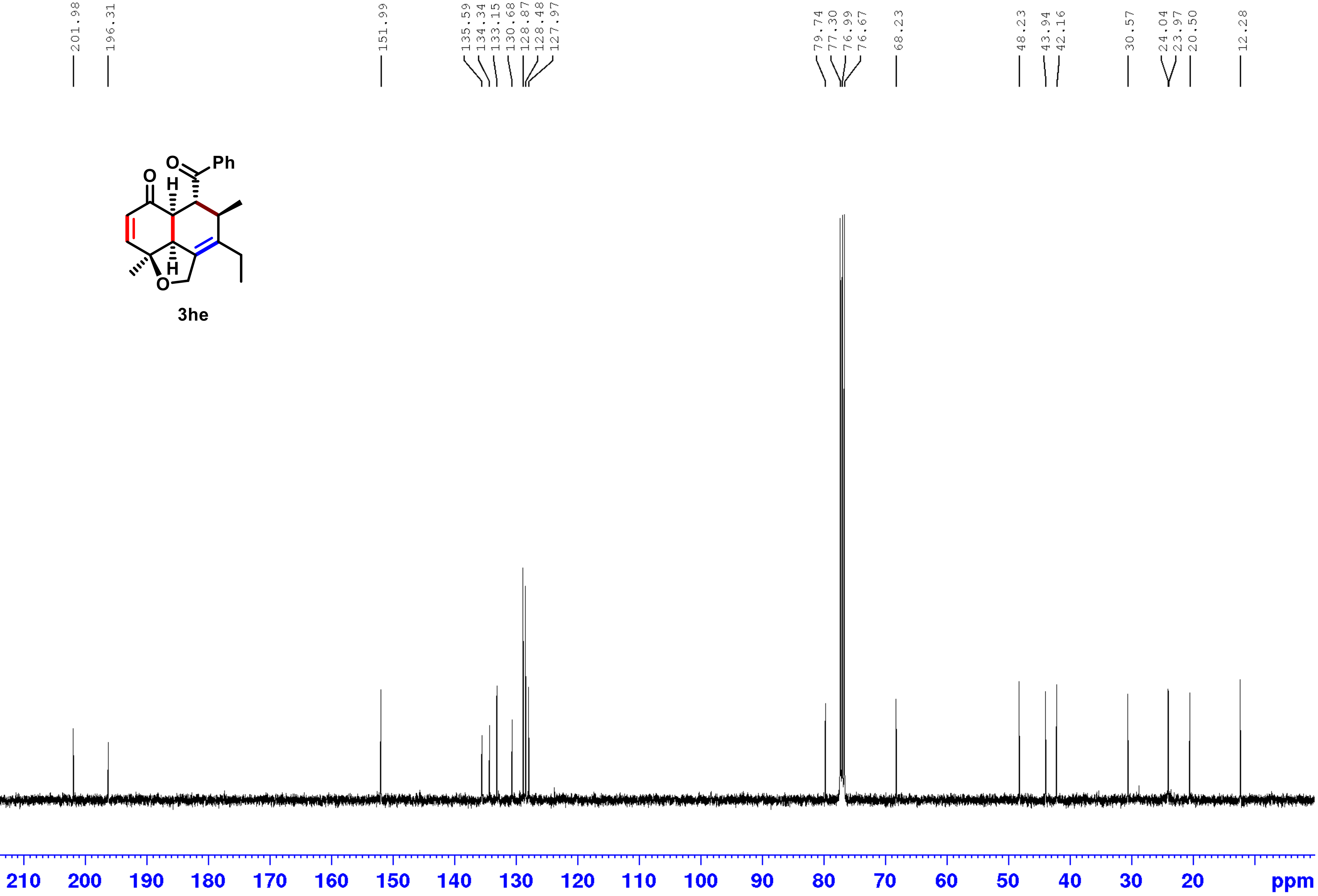
201.98
196.31
151.99
135.59
134.34
133.15
130.68
128.87
128.48
127.97
79.74
77.30
76.99
76.67
68.23
48.23
43.94
42.16
30.57
24.04
23.97
20.50
12.28
O
Ph
O
H
H
O
3he
210
200
190
180
170
160
150
140
130
120
110
100
90
80
70
60
50
40
30
20
ppm

Chiral Separation (SFC): Chiralpak IA; Flow (CO_2) = 3.0 mL/min; Flow (isopropanol) = 0.3 mL/min; T = 25 ºC; λ = 250 nm; Back pressure = 15 Mpa

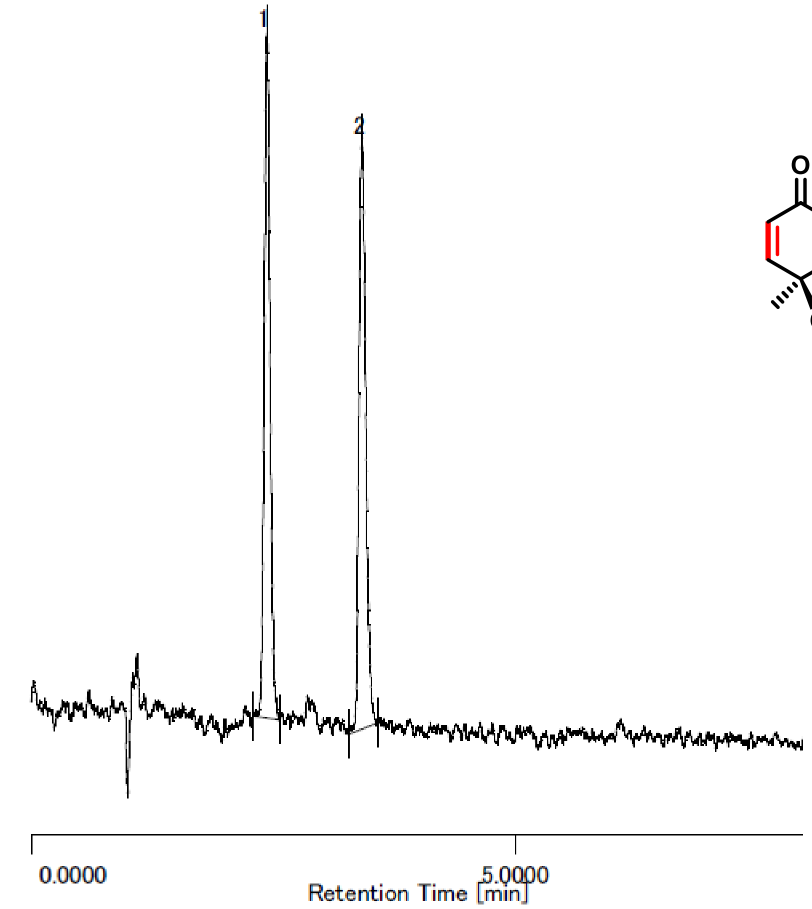

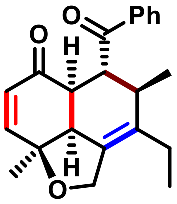

3he

98% ee

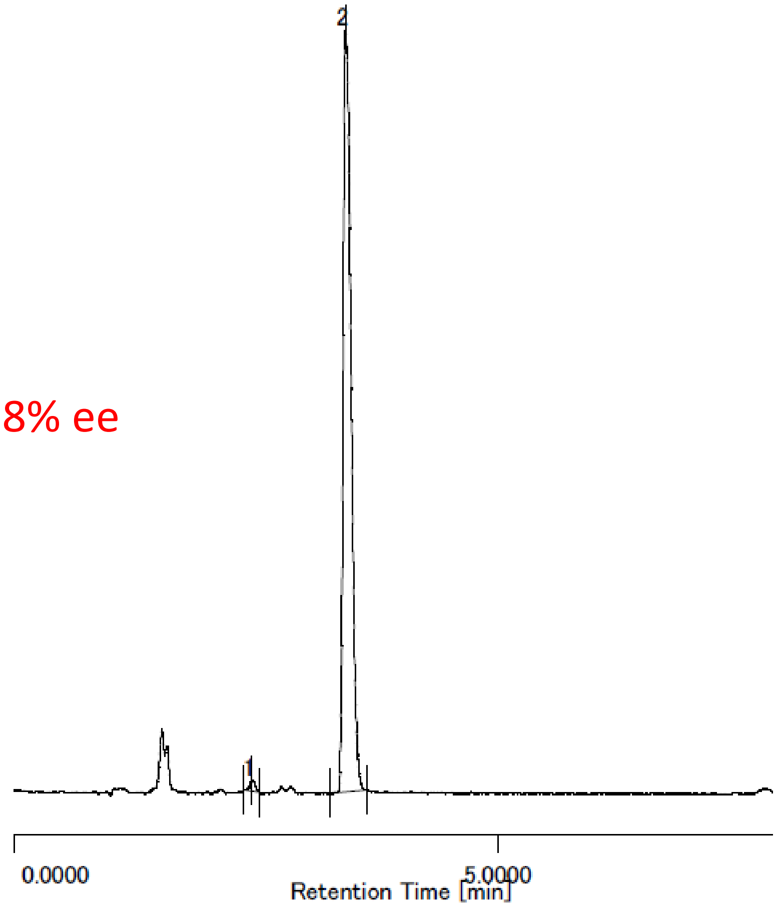

ChromatogramName R-718-rac.dienone-ethyl-yne+PPK

#	CH	tR	Area	Height	Area%
1	10	2.4333	464991	109950	50.386
2	10	3.4150	457871	94592	49.614

ChromatogramName R-720-chiral-dienone-ethyl-yne+PPK

#	CH	tR	Area	Height	Area%
1	10	2.4600	90646	23505	0.998
2	10	3.4233	8992726	1594106	99.002

Supplementary Figure 25. ^{1}H, ^{13}C-NMR and SFC spectra of product 3ic

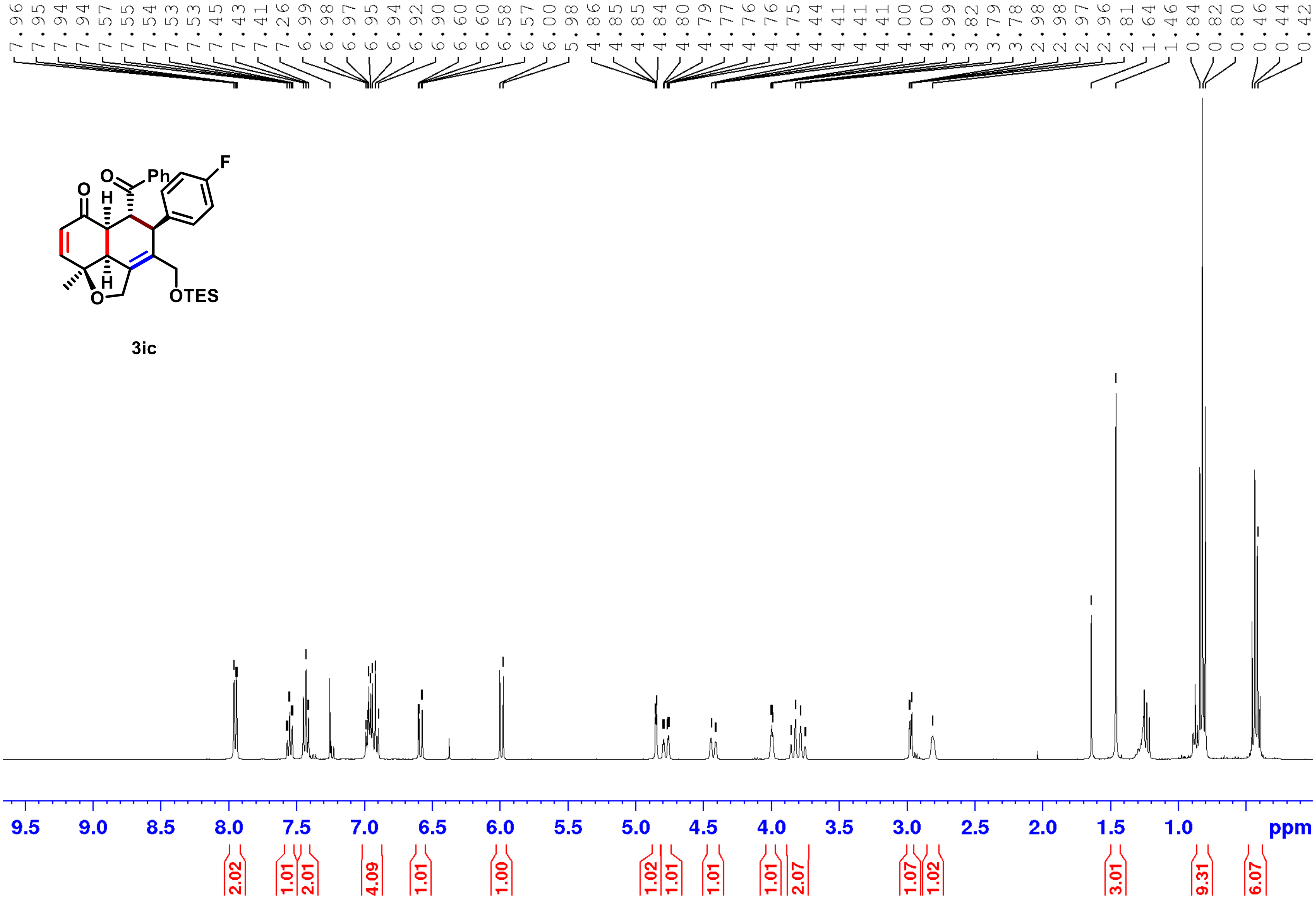

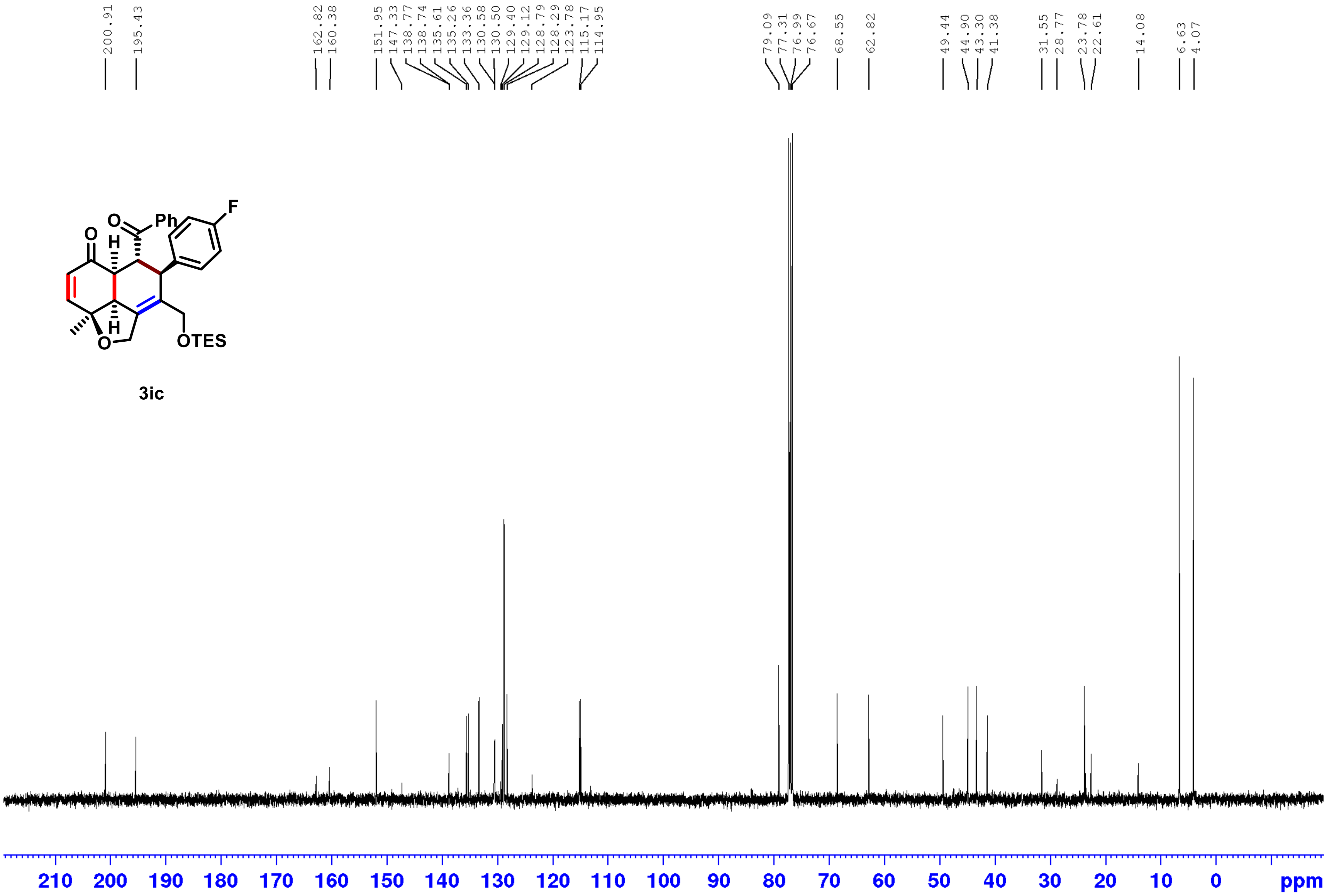
200.91
195.43
162.82
160.38
151.95
147.33
138.77
138.74
135.61
135.26
133.36
130.58
130.50
129.40
129.12
128.79
128.29
123.78
115.17
114.95
79.09
77.31
76.99
76.67
68.55
62.82
49.44
44.90
43.30
41.38
31.55
28.77
23.78
22.61
14.08
6.63
4.07
O
Ph
F
O
H
H
O
OTES
3ic
210 200 190 180 170 160 150 140 130 120 110 100 90 80 70 60 50 40 30 20 10 0 ppm

Chiral Separation (SFC): Chiralpak IA; Flow (CO_2) = 3.0 mL/min; Flow (isopropanol) = 0.3 mL/min; T = 25 ºC; λ = 250 nm; Back pressure = 15 Mpa

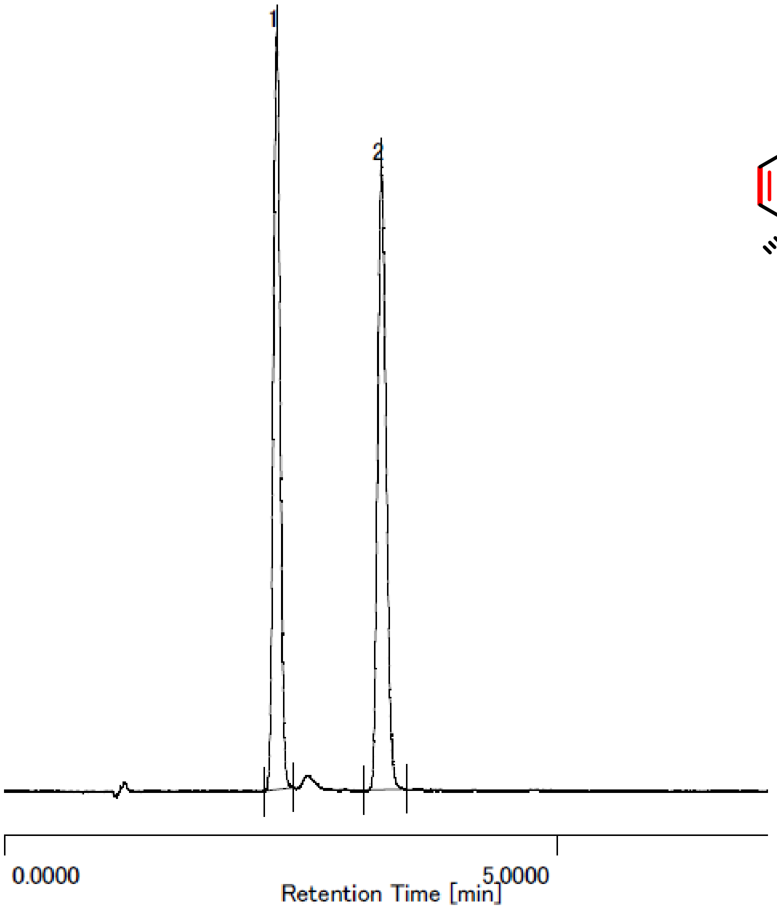

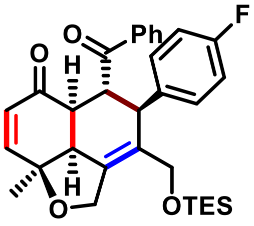

3ic

95% ee

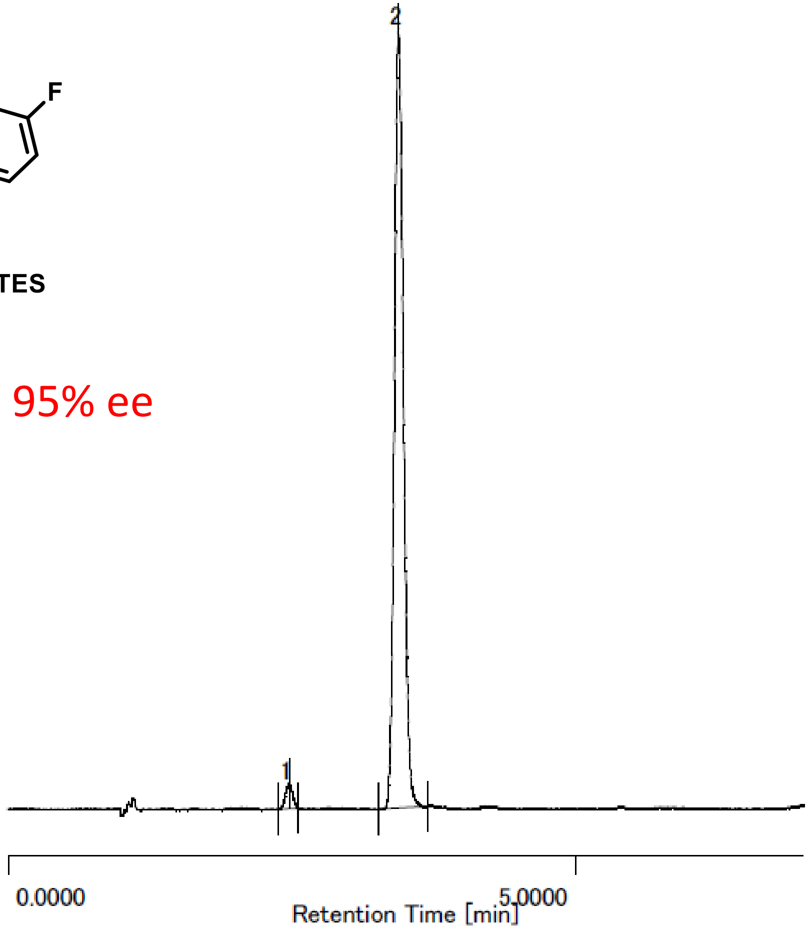

ChromatogramName R-707-rac1

#	CH	tR	Area	Height	Area%
1	9	2.4600	2224319	519973	49.565
2	9	3.4067	2263399	429511	50.435

ChromatogramName R-710-chir-OTES-F-chal.

#	CH	tR	Area	Height	Area%
1	9	2.4767	58021	13359	2.495
2	9	3.4367	2267722	412149	97.505

Supplementary Figure 26. ^{1}H, ^{13}C-NMR and SFC spectra of product 3ik

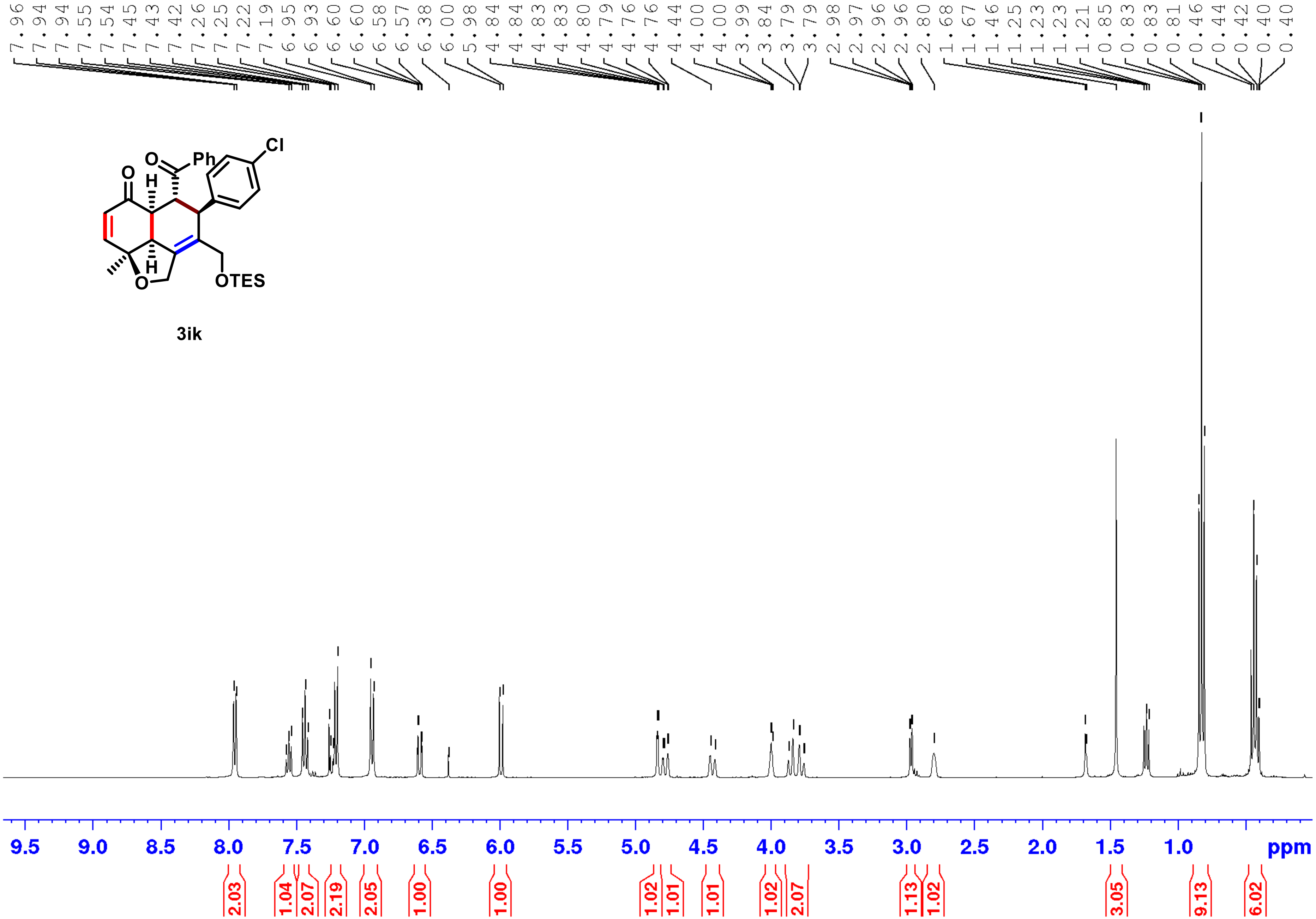

3ik

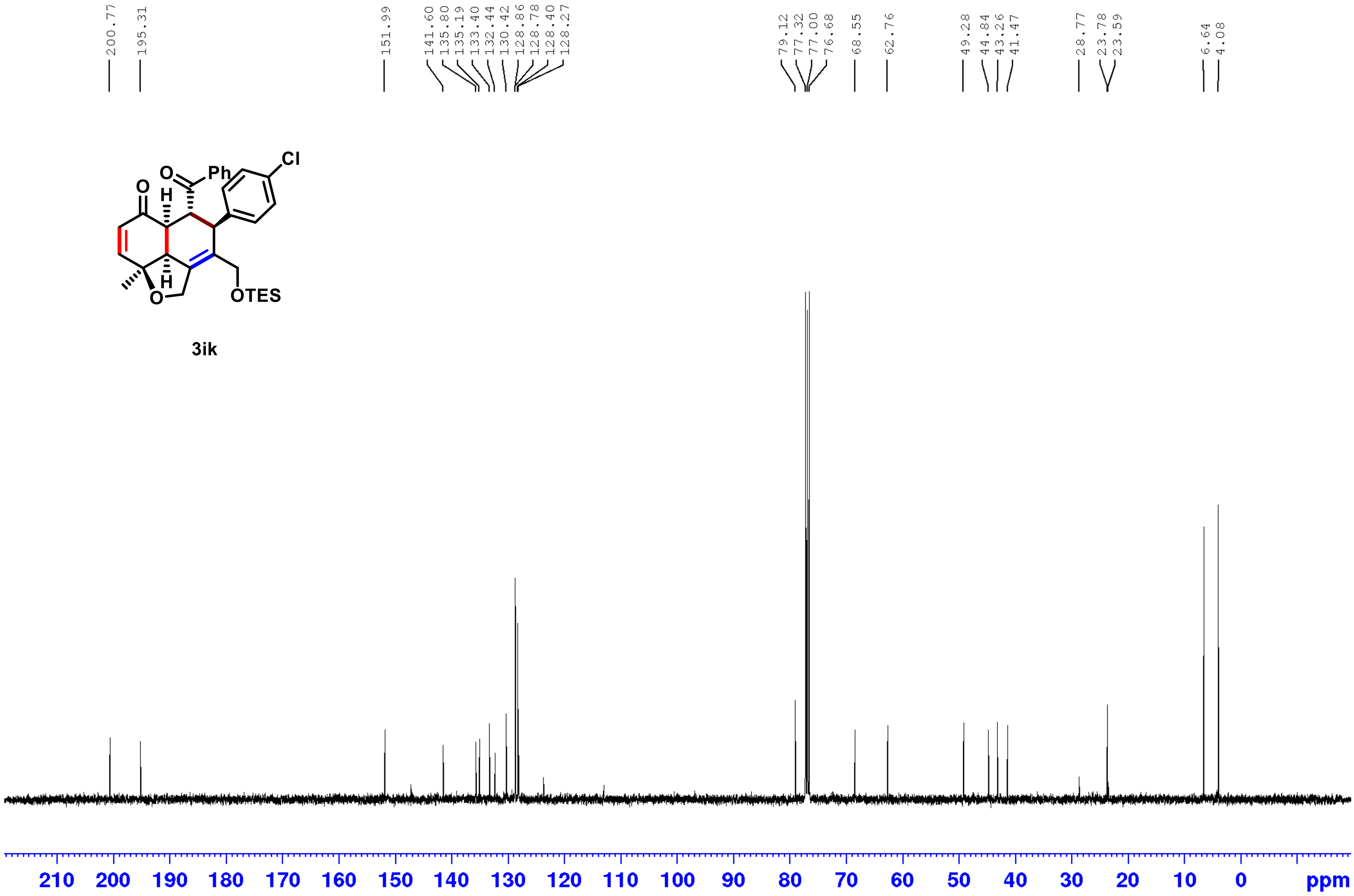
200.77
195.31
151.99
141.60
135.80
135.19
133.40
132.44
130.42
128.86
128.78
128.40
128.27
79.12
77.32
77.00
76.68
68.55
62.76
49.28
44.84
43.26
41.47
28.77
23.78
23.59
6.64
4.08
O
Ph
Cl
O
H
H
O
OTES
3ik
210 200 190 180 170 160 150 140 130 120 110 100 90 80 70 60 50 40 30 20 10 0 ppm

Chiral Separation (SFC): Chiralpak IA; Flow (CO_2) = 3.0 mL/min; Flow (isopropanol) = 0.3 mL/min; T = 25 °C; λ = 250 nm; Back pressure = 15 Mpa

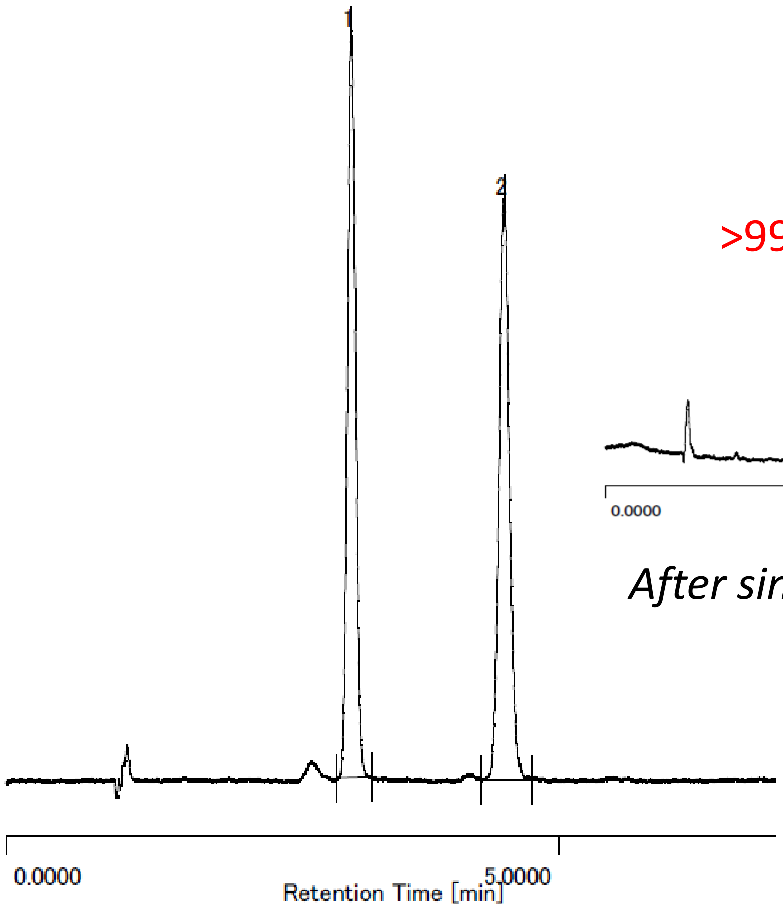

ChromatogramName R-712-rac1

#	CH	tR	Area	Height	Area%
1	9	3.1217	713916	139962	49.615
2	9	4.4983	724992	108951	50.385

>99.9% ee

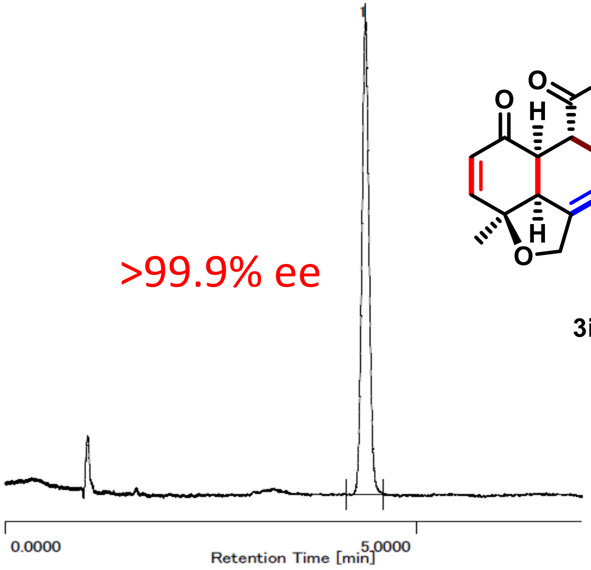

After single recrystallization

3ik

97% ee

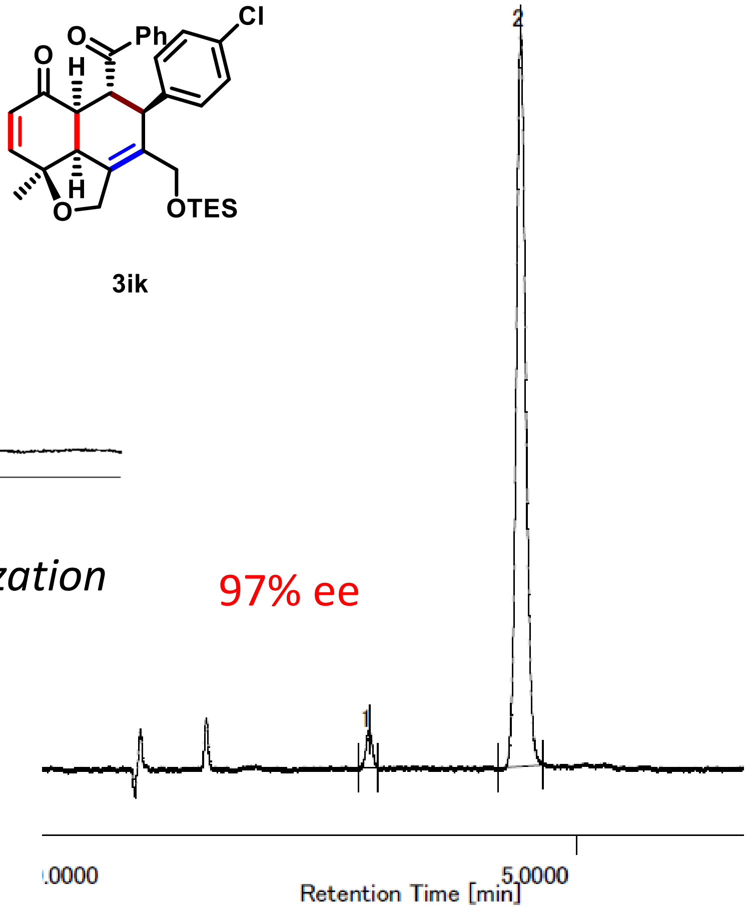

ChromatogramName R-714--chir-OTES-Cl-chal.

#	CH	tR	Area	Height	Area%
1	9	3.1250	26451	6936	3.161
2	9	4.4933	810292	135802	96.839

Supplementary Figure 27. ¹H and ¹³C-NMR spectra of product 4

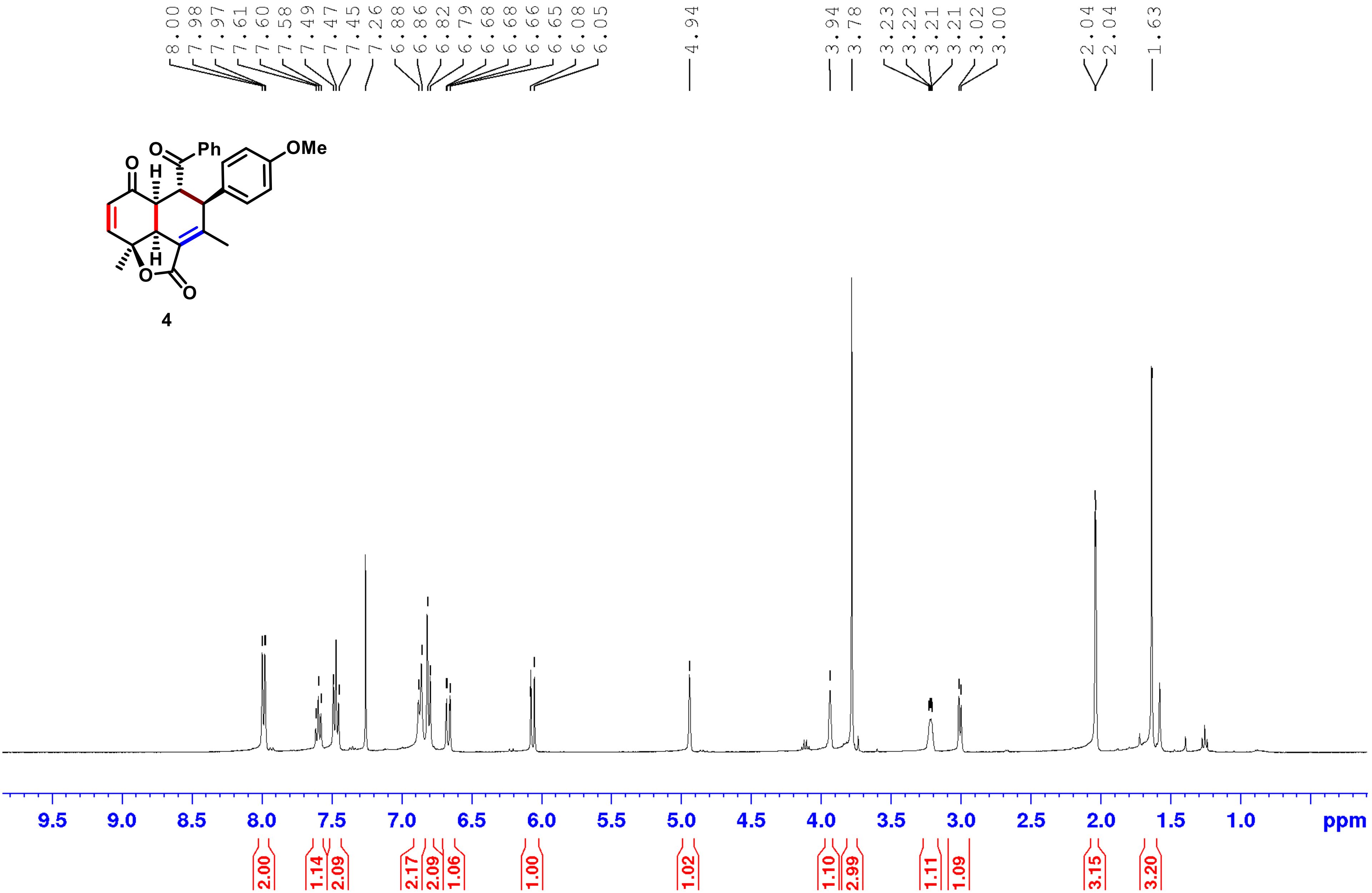

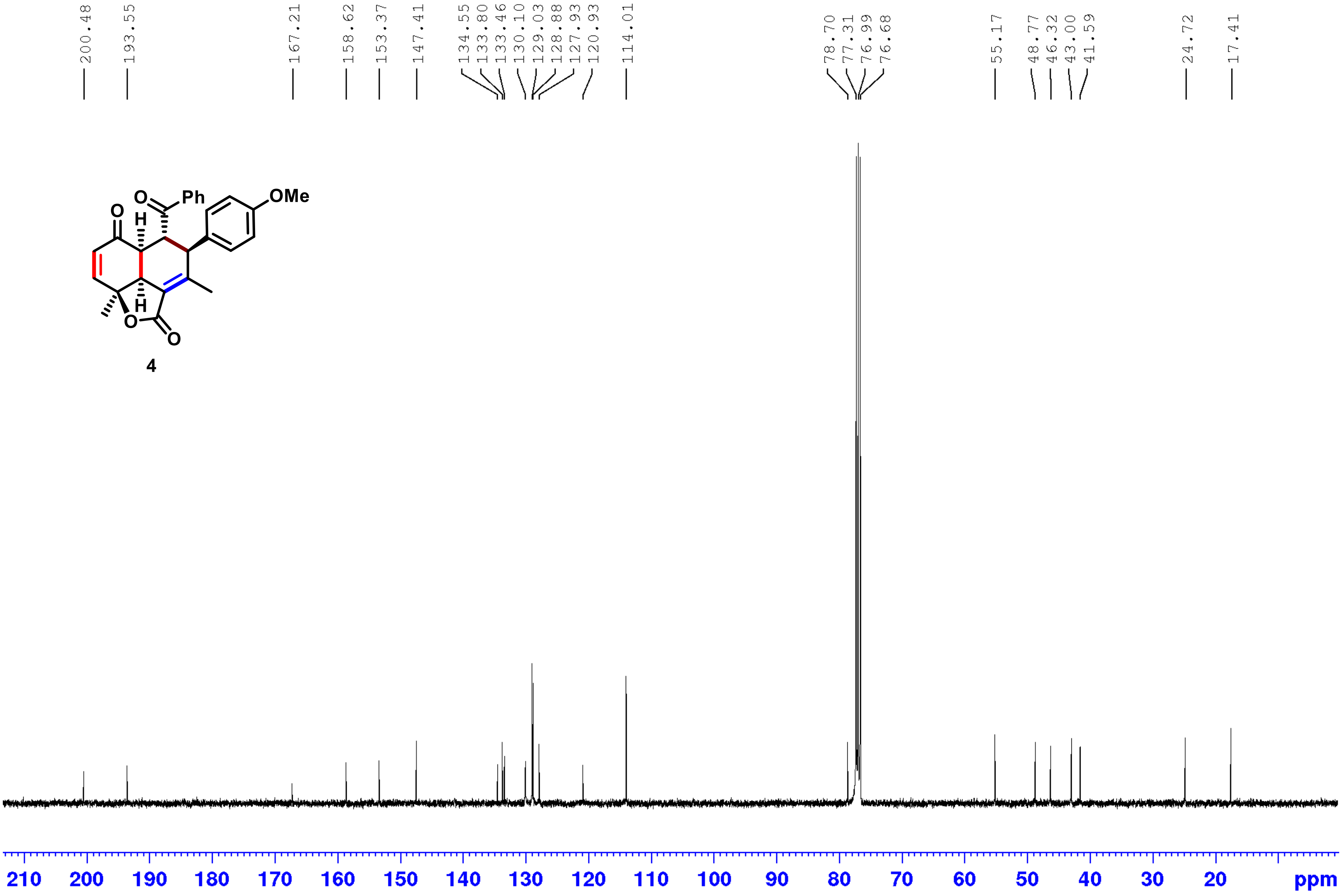
200.48
193.55
167.21
158.62
153.37
147.41
134.55
133.80
133.46
130.10
129.03
128.88
127.93
120.93
114.01
78.70
77.31
76.99
76.68
55.17
48.77
46.32
43.00
41.59
24.72
17.41
O
Ph
OMe
O
H
H
O
O
4
210 200 190 180 170 160 150 140 130 120 110 100 90 80 70 60 50 40 30 20 ppm

Supplementary Figure 28. ^{1}H and ^{13}C-NMR of product 5

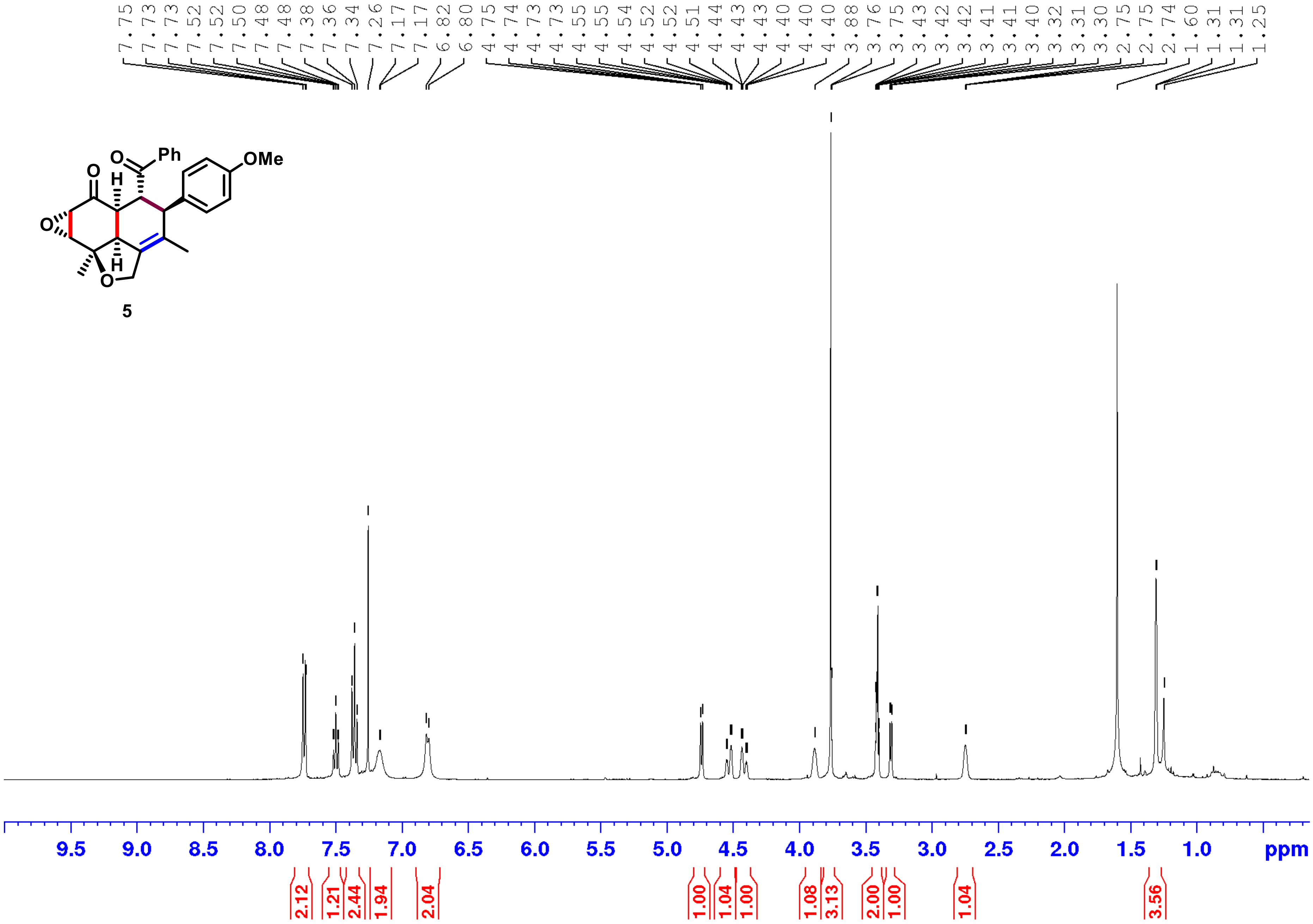

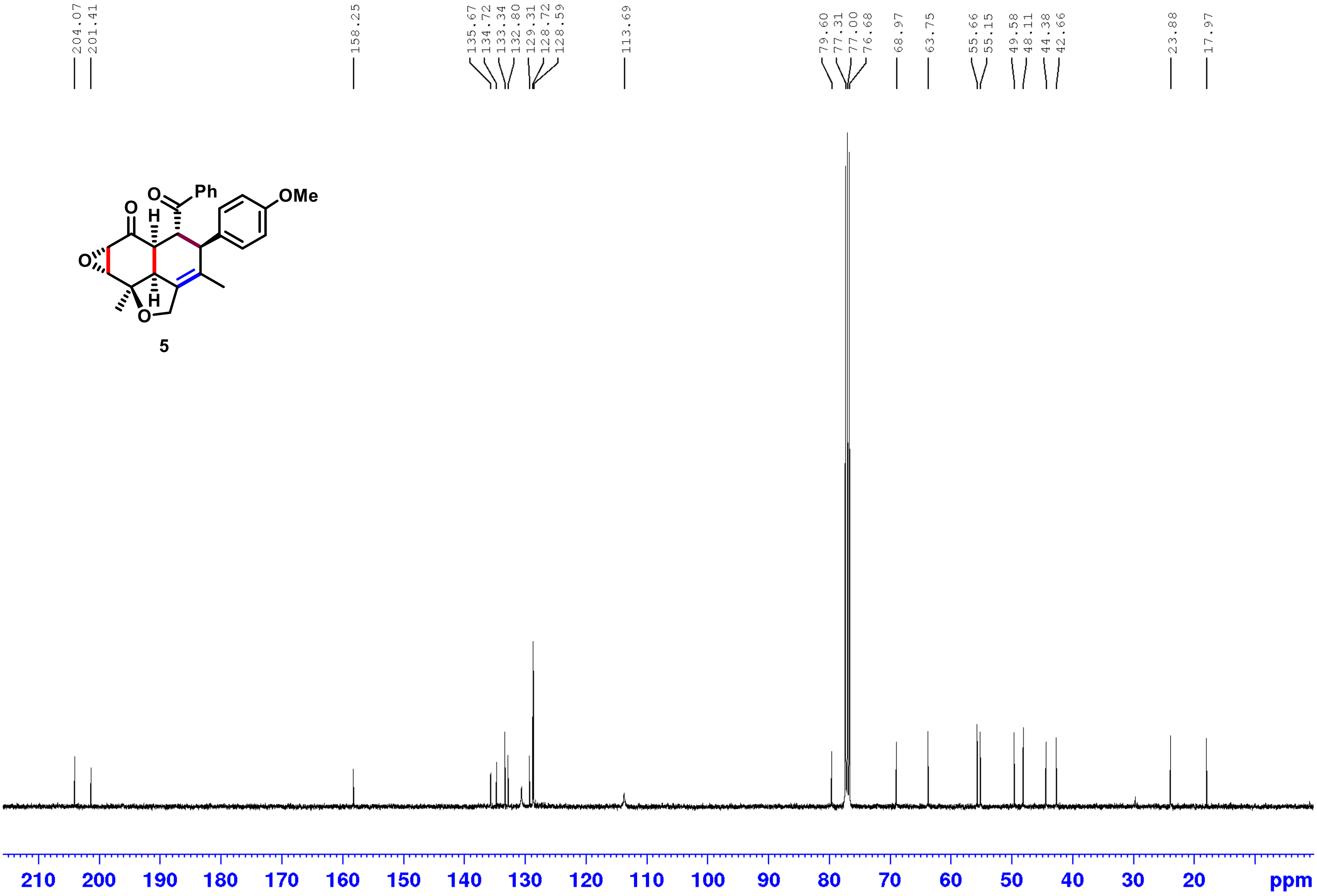
5
204.07
201.41
158.25
135.67
134.72
133.34
132.80
129.31
128.72
128.59
113.69
79.60
77.31
77.00
76.68
68.97
63.75
55.66
55.15
49.58
48.11
44.38
42.66
23.88
17.97
210
200
190
180
170
160
150
140
130
120
110
100
90
80
70
60
50
40
30
20
ppm

Supplementary Figure 29. ^{1}H and ^{13}C-NMR of product 6

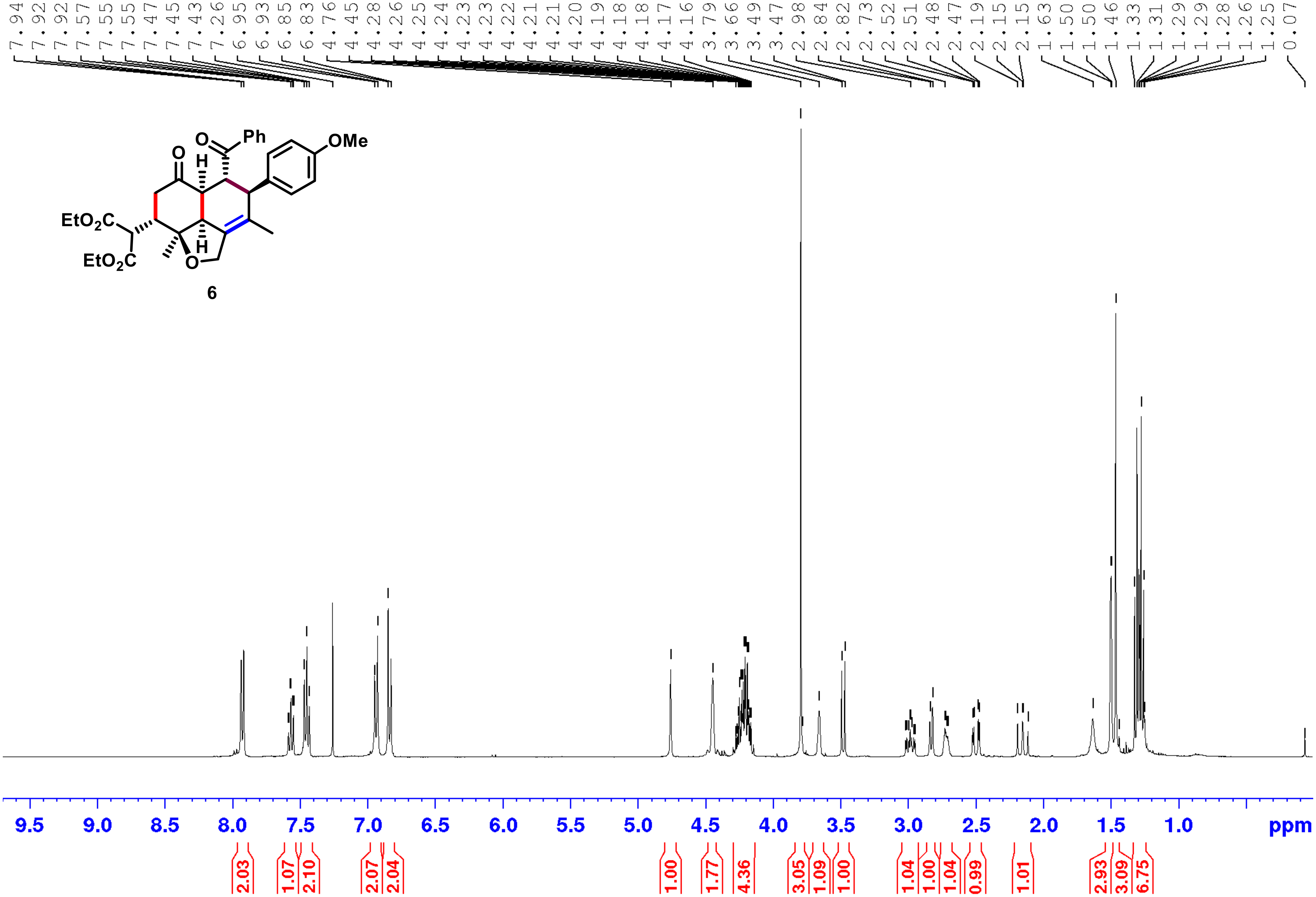

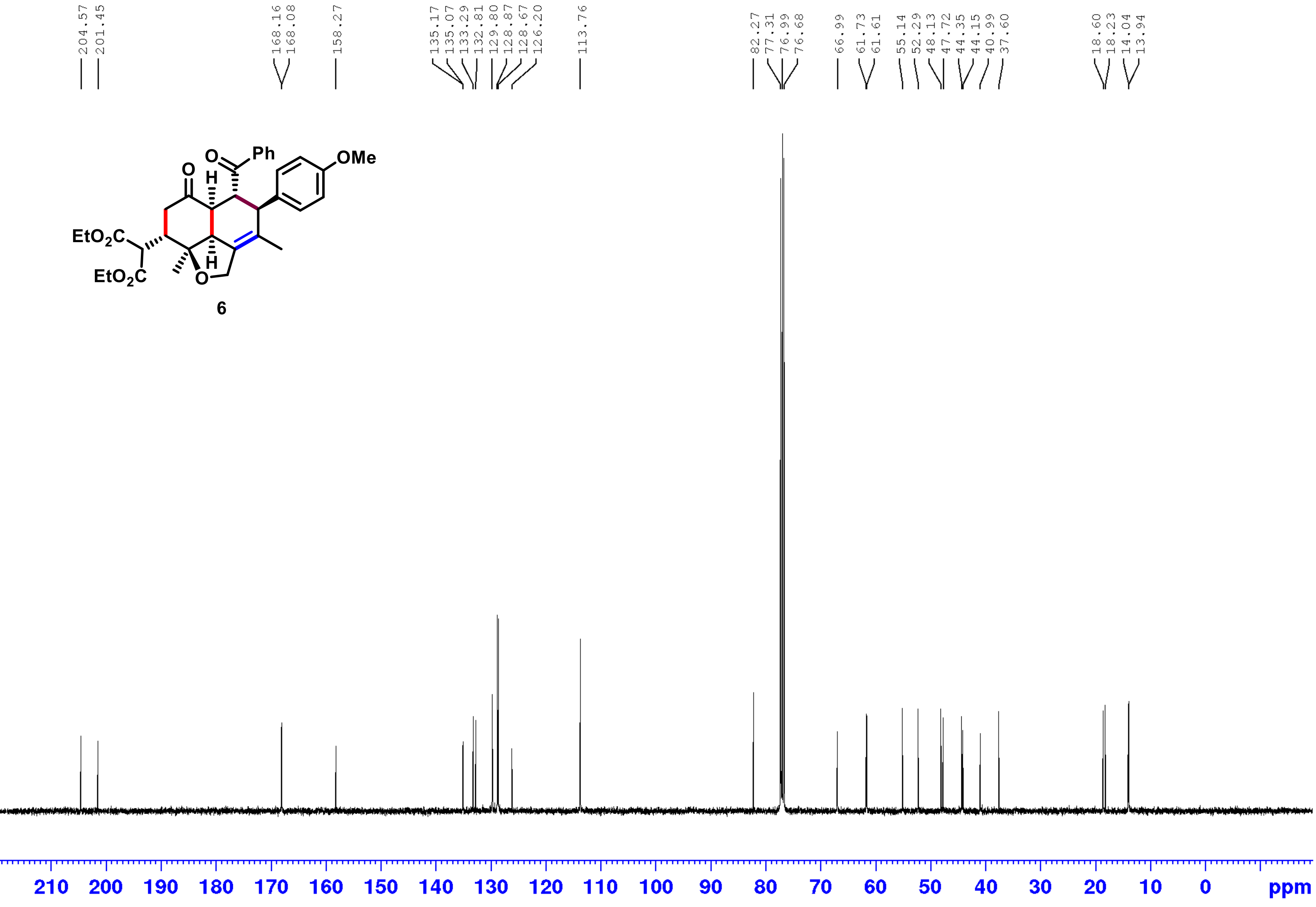
204.57
201.45
168.16
168.08
158.27
135.17
135.07
133.29
132.81
129.80
128.87
128.67
126.20
113.76
82.27
77.31
76.99
76.68
66.99
61.73
61.61
55.14
52.29
48.13
47.72
44.35
44.15
40.99
37.60
18.60
18.23
14.04
13.94
O
Ph
OMe
O
H
EtO_2C
EtO_2C
H
O
6
210
200
190
180
170
160
150
140
130
120
110
100
90
80
70
60
50
40
30
20
10
0
ppm

Supplementary Figure 30. ^{1}H and ^{13}C-NMR of product 7

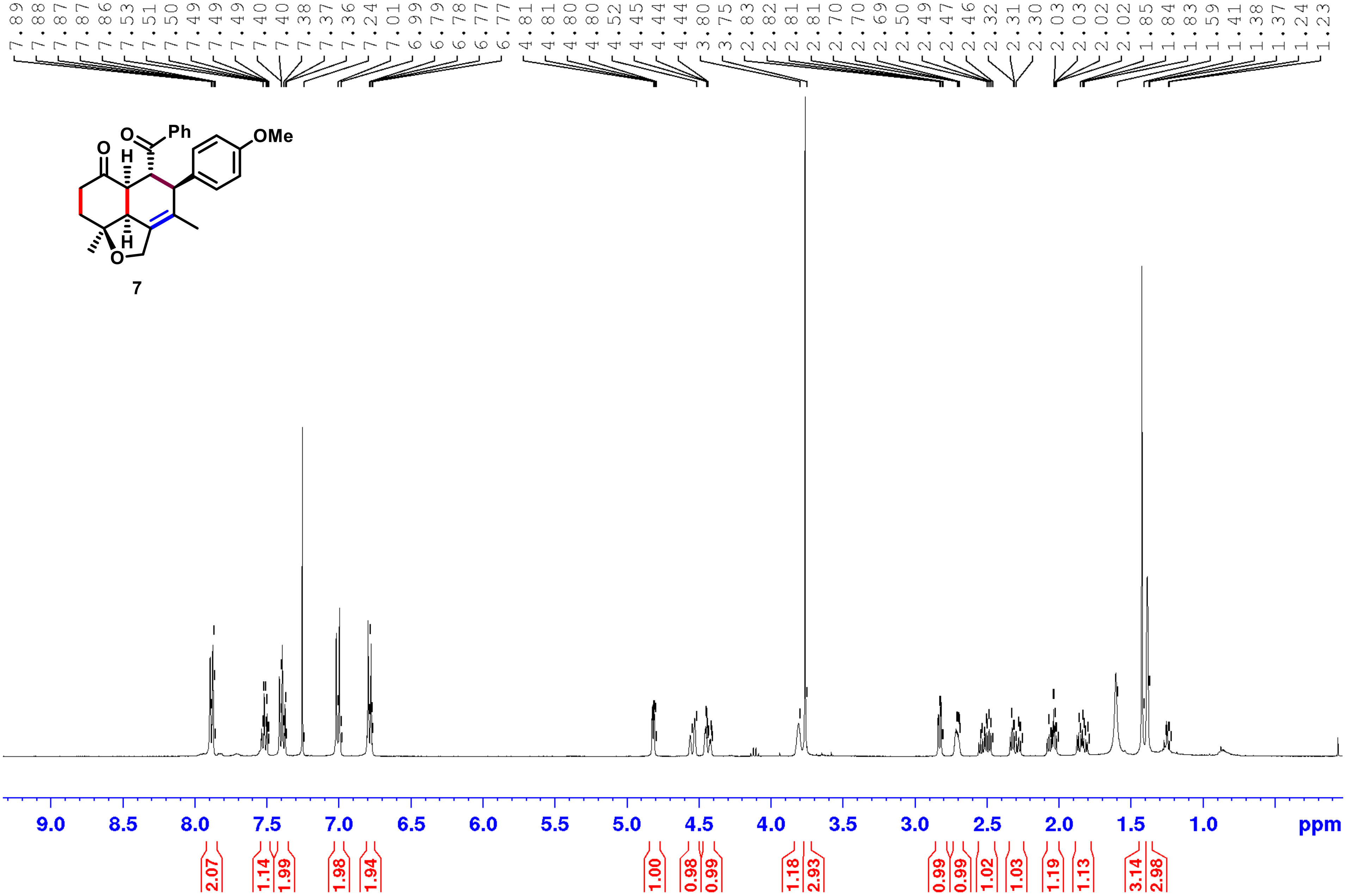

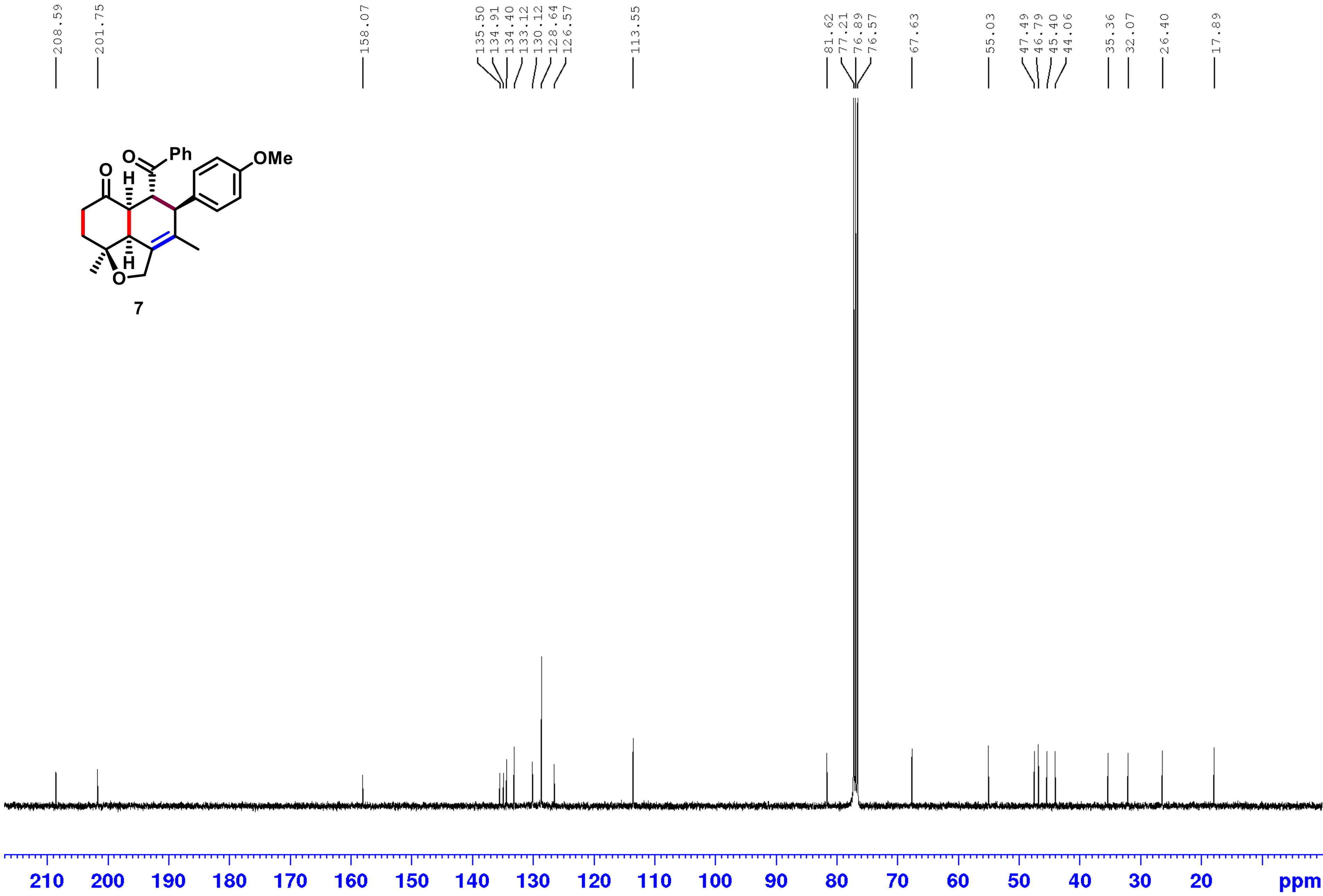

208.59
201.75
158.07
135.50
134.91
134.40
133.12
130.12
128.64
126.57
113.55
81.62
77.21
76.89
76.57
67.63
55.03
47.49
46.79
45.40
44.06
35.36
32.07
26.40
17.89
O
Ph
OMe
O
H
H
O
7
210
200
190
180
170
160
150
140
130
120
110
100
90
80
70
60
50
40
30
20
ppm

Supplementary Figure 31. ^{1}H and ^{13}C-NMR of product 8

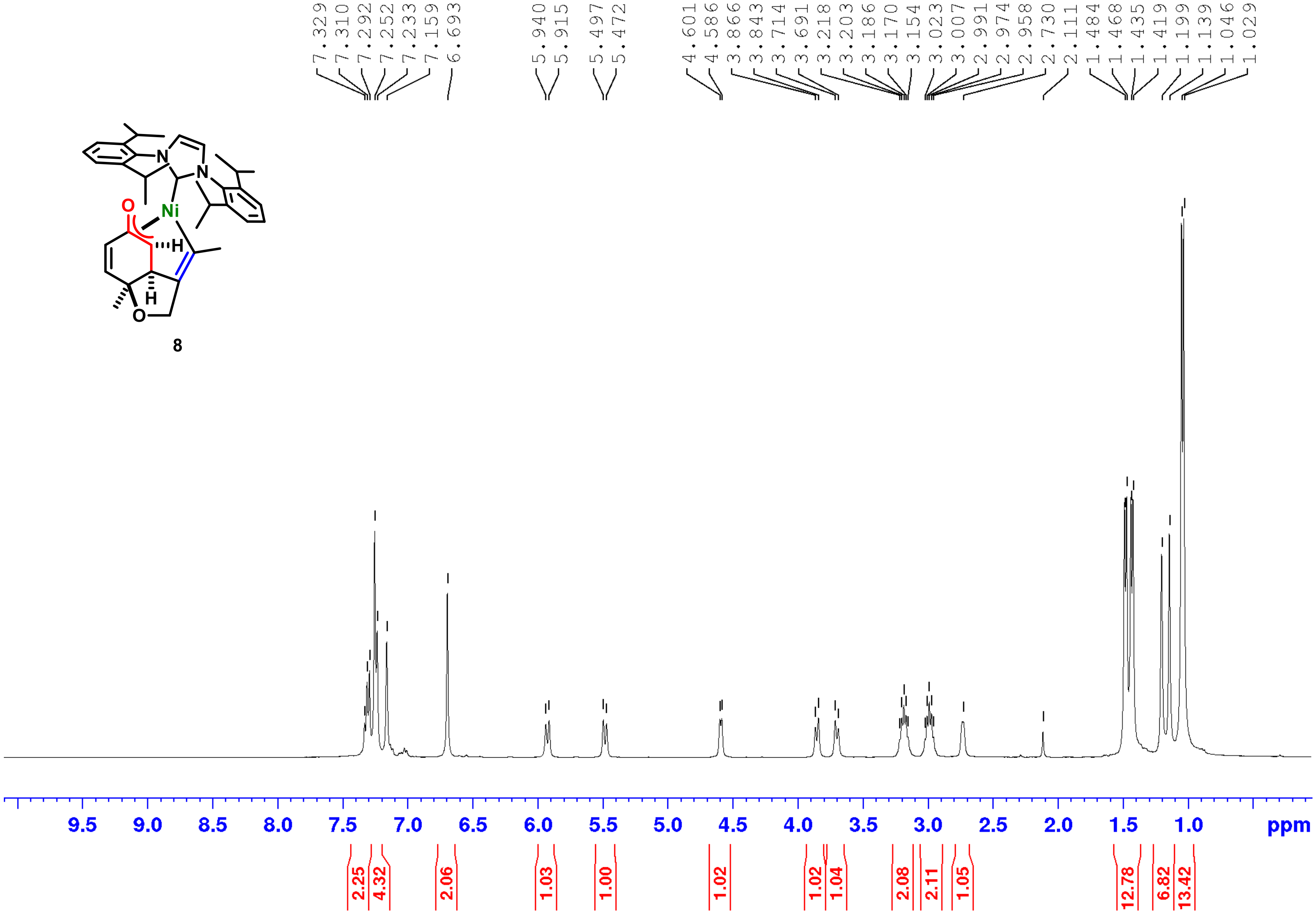

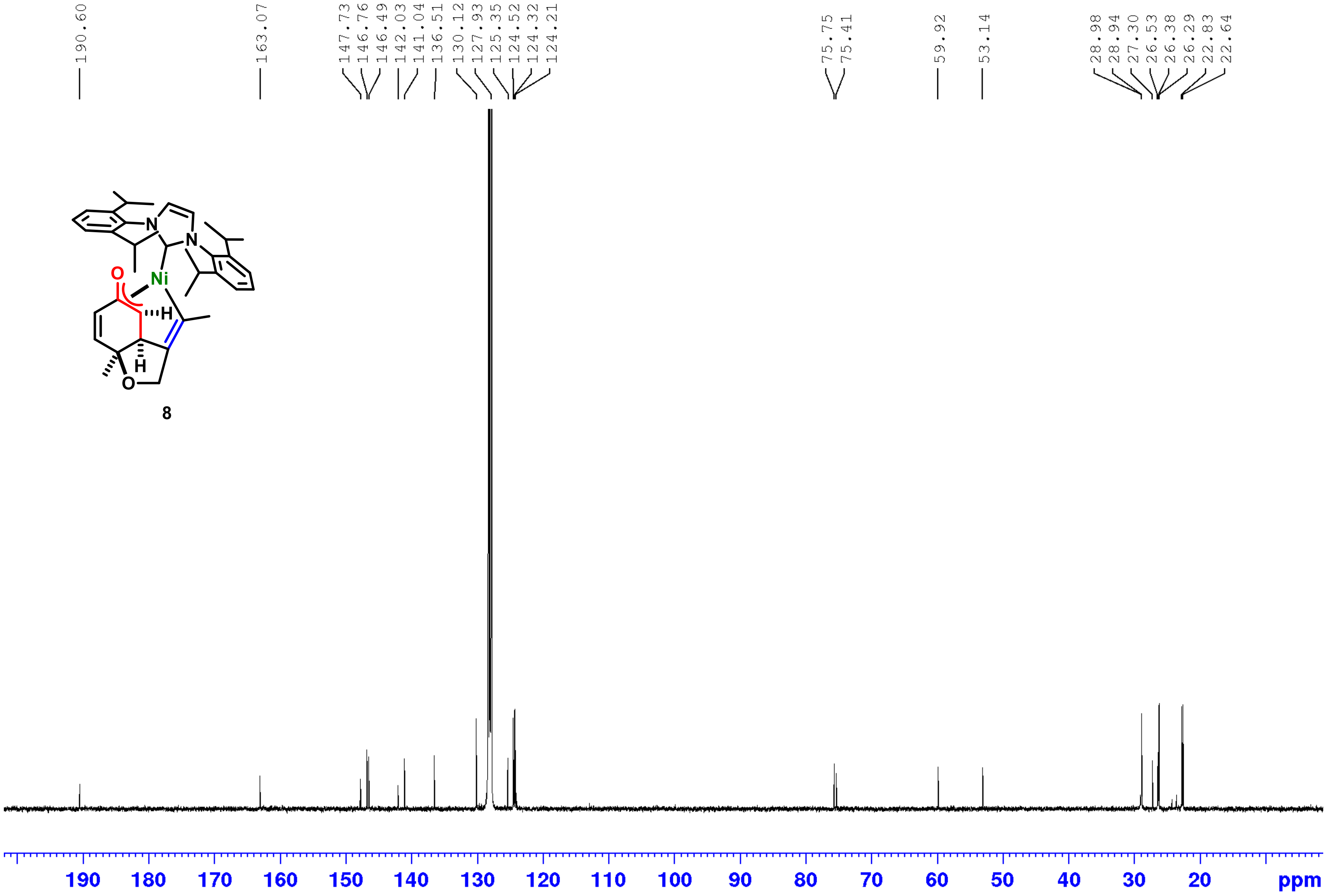
190.60
163.07
147.73
146.76
146.49
142.03
141.04
136.51
130.12
127.93
125.35
124.52
124.32
124.21
75.75
75.41
59.92
53.14
28.98
28.94
27.30
26.53
26.38
26.29
22.83
22.64
N
N
Ni
O
H
H
O
8
190
180
170
160
150
140
130
120
110
100
90
80
70
60
50
40
30
20
ppm

Supplementary References

1. Seiders, T. J., Ward, D. W. & Grubbs, R. H. Enantioselective ruthenium-catalyzed ring-closing metathesis. *Org. Lett.* **3**, 3225–3228 (2001).
2. Luan, X., Mariz, R., Robert, C., Gatti, M., Blumentritt, S., Linden, A. & Dorta, R. Matching the chirality of monodentate *N*-heterocyclic carbene ligands: a case study on well-defined palladium complexes for the asymmetric a-arylation of amides. *Org. Lett.* **10**, 5569–5572 (2008).
3. Matsumoto, Y., Yamada, K. & Tomioka, K. C_2 symmetric chiral NHC ligand for asymmetric quaternary carbon constructing copper-catalyzed conjugate addition of Grignard reagents to 3-substituted cyclohexenones. *J. Org. Chem.* **73**, 4578–4581 (2008).
4. Chaulagain, M. R., Sormunen, G. J. & Montgomery, J. New *N*-heterocyclic carbene ligand and its application in asymmetric nickel-catalyzed aldehyde/alkyne reductive couplings. *J. Am. Chem. Soc.* **129**, 9568–9569 (2007).
5. Tello-Aburto, R., Kalstabakken, K. A. & Harned, A. M. Ligand and substrate effects during Pd-catalyzed cyclizations of alkyne-tethered cyclohexadienones. *Org. Biomol. Chem.* **11**, 5596–5604, (2013).
6. Takenaka, K., Mohanta, S. C. & Sasai, H. Palladium enolate umpolung: cyclative diacetoxylation of alkynyl cyclohexadienones promoted by a Pd/SPRIX catalyst. *Angew. Chem. Int. Ed.* **53**, 4675–4679, (2014).
7. He, Z.-T., Tang, X.-Q., Xie, L.-B., Cheng, M., Tian, P. & Lin, G. Q. Efficient access to bicyclo[4.3.0]nonanes: copper-catalyzed asymmetric silylative cyclization of cyclohexadienone-tethered allenes. *Angew. Chem. Int. Ed.* **54**, 14815–14818 (2015).
8. Hexum, J. K., Tello-Aburto, R., Struntz, N. B., Harned, A. M. & Harki, D. A. Bicyclic cyclohexenones asinhibitors of NF-κB signaling. *ACS Medicinal Chemistry Letters* **3**, 459–464, (2012).
